# Phase 1 randomized trials to assess safety, pharmacokinetics, and vaginal bleeding associated with use of extended duration dapivirine and levonorgestrel vaginal rings

**DOI:** 10.1371/journal.pone.0304552

**Published:** 2024-06-05

**Authors:** Sharon L. Achilles, Clifton W. Kelly, Craig J. Hoesley, Diana L. Blithe, Jill Brown, Barbra A. Richardson, Brid Devlin, Craig W. Hendrix, Samuel M. Poloyac, Mark A. Marzinke, Holly Gundacker, Devika Singh, Jeanna M. Piper, Sherri Johnson, John Steytler, Beatrice A. Chen, on behalf of the MTN-030/IPM 041 and MTN-044/IPM 053/CCN019 Protocol Teams for the Microbicide Trials Network and the Contraceptive Clinical Trials Network

**Affiliations:** 1 Department of Obstetrics, Gynecology, and Reproductive Sciences, University of Pittsburgh, Pittsburgh, Pennsylvania, United States of America; 2 Magee-Womens Research Institute, Pittsburgh, Pennsylvania, United States of America; 3 Statistical Center for HIV/AIDS Research & Prevention, Fred Hutchinson Cancer Center, Seattle, Washington, United States of America; 4 University of Alabama at Birmingham Heersink School of Medicine, Birmingham, Alabama, United States of America; 5 National Institute of Child Health and Human Development, Contraceptive Development Program, DIPHR, NIH, Bethesda, Maryland, United States of America; 6 Department of Biostatistics, University of Washington, Seattle, Washington, United States of America; 7 International Partnership for Microbicides, Silver Spring, Maryland, United States of America; 8 Department of Medicine, Division of Clinical Pharmacology, Johns Hopkins University School of Medicine, Baltimore, Maryland, United States of America; 9 Department of Pharmaceutical Sciences, School of Pharmacy, University of Pittsburgh, Pittsburgh, Pennsylvania, United States of America; 10 National Institutes of Allergy and Infectious Disease, NIH, Bethesda, Maryland; 11 FHI 360, Durham, North Carolina, United States of America; National Institute of Allergy and Infectious Diseases, UNITED STATES

## Abstract

**Background:**

Vaginal rings formulated to deliver two drugs simultaneously have potential as user-controlled, long-acting methods for dual prevention of HIV and pregnancy.

**Methods:**

Two phase 1 randomized trials (MTN-030/IPM 041 and MTN-044/IPM 053/CCN019) respectively enrolled 24 and 25 healthy, HIV-negative participants to evaluate safety, pharmacokinetics, and vaginal bleeding associated with use of a vaginal ring containing 200mg dapivirine (DPV) and 320mg levonorgestrel (LNG) designed for 90-day use. MTN-030/IPM 041 compared the DPV/LNG ring to a DPV-only ring (200mg) over 14 days of use. MTN-044/IPM 053/CCN019 compared continuous or cyclic use of the DPV/LNG ring over 90 days of use. Safety was assessed by recording adverse events (AEs). DPV and LNG concentrations were quantified in plasma, cervicovaginal fluid, and cervical tissue. Vaginal bleeding was self-reported.

**Results:**

There were no differences in the proportion of participants with grade ≥2 genitourinary AEs or grade ≥3 AEs with DPV/LNG ring vs. DPV ring use (p = .22), or with DPV/LNG ring continuous vs. cyclic use (p = .67). Higher plasma DPV concentrations were observed in users of DPV/LNG compared to DPV-only rings (C_max_ p = 0.049; AUC p = 0.091). Plasma DPV and LNG concentrations were comparable with continuous and cyclic use (C_max_ p = 0.74; AUC p = 0.25). With cyclic use, median nadir plasma DPV concentration was approximately 300 pg/mL two days after removal and median t_1/2_ for cervicovaginal fluid DPV concentration was 5.76 hours (n = 3). Overall bleeding experiences did not differ between continuous and cyclic users (p = 0.12).

**Conclusions:**

The extended duration DPV/ LNG rings were well tolerated and the observed DPV concentrations in plasma and cervicovaginal fluid when used continuously exceeded concentrations observed in previous DPV ring efficacy studies. LNG concentrations in plasma were comparable with other efficacious LNG-based contraceptives. Genital DPV concentrations had a short half-life and were thus not well sustained following ring removal.

## Introduction

Multipurpose prevention technologies (MPTs) offer potential for combined protection against overlapping sexual and reproductive health risks, including unintended pregnancy, HIV, and other sexually transmitted infections. Unprotected heterosexual intercourse is the leading mode of HIV acquisition among women. Despite a significantly greater use of modern contraceptives, HIV pre-exposure prophylaxis (PrEP), and condoms over the past 20 years [1,2], nearly 121 million unintended pregnancies [3], two million new HIV infections [4], and 376 million other sexually transmitted infections [5] are reported globally each year. Oral PrEP was approved for use in 2012, however, PrEP uptake and continued daily use by those at risk of HIV acquisition remains low [6–8]. Recent advances have been made in both extended duration HIV prevention methods [9–11] and new MPT methods [12–16] that simultaneously address multiple health concerns, advancements that may improve uptake and use of PrEP [17–19].

Rings developed for vaginal delivery of contraceptives and hormone replacement therapy solved some of the challenges associated with conventional vaginal products (gels, suppositories, and inserts), which are often messy and coitally-dependent. Rings that are replaced monthly or less frequently also have convenience benefits over dosage forms that need to be used more frequently and this may be linked to continuation and effectiveness. Rings are user-controlled, discreet, and can be loaded with ample drug to provide protection over several months [16,20]. Rings have high user acceptability [21,22] and are currently under development for a wider range of reproductive health indications, most notably HIV prevention [23–31]. A ring containing 25mg of the antiretroviral dapivirine (DPV) and designed for continuous HIV prevention with vaginal wear for one month, received prequalification and recommendation by the WHO for HIV prevention [32] following a positive review of this ring by the European Medicines Agency for use by adult cisgender women when oral pre-exposure HIV prophylaxis is not or cannot be used, or is unavailable [33,34]. In the phase 3 MTN-020 DPV efficacy trial, increased protection against HIV acquisition was observed with increased product adherence; plasma DPV concentrations >95 pg/mL were associated with more consistent product use [25]. More recently, a DPV vaginal ring containing 200mg DPV designed for extended use (3 month) has been evaluated for pharmacokinetics and safety; geometric mean concentrations of 411 pg/mL and 315 pg/mL were observed after 28 days and 91 days of product use, respectively [35].

The development of a ring containing DPV for HIV prevention and levonorgestrel (LNG) for contraception, both with adequate loading doses to support extended release for 3-months continuous use, allows for use of a single ring with less frequent ring replacements compared to simultaneous use of monthly contraceptive and HIV preventative rings. Similar to contraceptive rings designed for use over multiple cycles [36,37], extended duration MPT rings replaced quarterly may further reduce user and provider burden, thereby increasing accessibility to important preventative sexual and reproductive health options and improving adherence. The Microbicide Trials Network (MTN)-030/International Partnership for Microbicides (IPM) 041 and the MTN-044/IPM 053/Contraceptive Clinical Trials Network (CCN)019 studies evaluated the safety, pharmacokinetics (PK), and vaginal bleeding associated with up to 90-days use of an extended duration vaginal ring loaded with 200mg DPV and 320mg LNG.

## Materials and methods

### Study design

We conducted two phase 1 randomized trials to assess safety, PK, and vaginal bleeding of the DPV/LNG MPT ring. MTN-030/IPM 041 (ClinicalTrials.gov Identifier: NCT02855346) compared a DPV-only ring to a combination DPV/LNG ring over 14 days of continuous use in a trial conducted at the University of Pittsburgh (Pittsburgh, PA) and the University of Alabama at Birmingham (Birmingham, AL). The full trial protocol can be accessed here: https://www.mtnstopshiv.org/research/studies/mtn-030ipm-041. MTN-044/IPM 053/CCN019 (ClinicalTrials.gov Identifier: NCT03467347) compared continuous to cyclic use of DPV/LNG rings over 90 days in a trial conducted at the University of Pittsburgh (Pittsburgh, PA). The full trial protocol can be accessed here: https://www.mtnstopshiv.org/research/studies/mtn-044ipm-053ccn019. IRBs: University of Pittsburgh IRB, Pittsburgh, PA; University of Alabama at Birmingham IRB, Birmingham, AL; Adverra, Columbia, MD. IRB approval numbers: MTN-030 clinical trial: University of Alabama–F160622004; University of Pittsburgh–PRO16040579. MTN044 Clinical trial: University of Pittsburgh: PRO18020450 and MOD18020450- 01/PRO18020450; Adverra: MOD00272363.Clinical Trial Protocols and Informed Consent Forms were approved by the IRBs listed above and written consent was obtained from each participant prior to initiating any study procedures. A list of protocol modifications and modification to the statistical analysis plans for both trials can be found in S1 Appendix.

The DPV and DPV/LNG rings have identical appearance and are flexible, smooth, off-white, and have an outer diameter of 56mm and a cross-sectional diameter of 7.7mm with DPV (200mg) with or without LNG (320mg) dispersed in a platinum-cured silicone matrix. These rings are designed to provide sustained release over a minimum of three months.

Safety was a primary objective in MTN-030/IPM 041 and was a secondary objective in MTN-044/IPM 053/CCN019. Quantification of local and systemic DPV and LNG concentrations with use of the DPV/LNG rings was a primary study objective of both trials. Vaginal bleeding was a secondary objective in MTN-030/IPM 041 and was an exploratory objective in MTN-044/IPM 053/CCN019. Methods for assessing safety, local and systemic drug concentrations, and vaginal bleeding were the same in both trials as follows unless otherwise noted: Safety was evaluated as the proportion of participants with grade ≥2 genitourinary adverse events (AEs) and grade ≥3 AEs, using the Division of AIDS Table for Grading the Severity of Adult and Pediatric Adverse Events and Female Genital Grading Table for Use in Microbicide Studies [38,39]. Primary PK endpoints included DPV and LNG concentrations in plasma, cervicovaginal fluid (CVF), and cervical tissue (tissue only evaluated in MTN-044/IPM 053/CCN019). Participants answered a daily questionnaire by short message service (SMS) that included reporting of vaginal bleeding as follows: none, light (used no protection, toilet paper, or panty liner only), moderate (used pad or tampon), or heavy (leaked through pad or tampon).

In both trials, eligible participants were born female, aged 18–45, HIV-negative, using effective non hormonal method of contraception, having regular menstrual cycles, and generally healthy. Major exclusion criteria included: pregnant/breastfeeding; use of pre-/post-exposure HIV prophylaxis in the past 3 months; medical contraindication to use of a progestin-only contraceptive; use of hormonal contraception in the past 28 days or use of depot medroxyprogesterone acetate in the past 6 months; current use of medications with potential for study drug interactions (strong CYP3A inhibitors/inducers, antibiotics, corticosteroids); unresolved urinary or reproductive tract infection; current sexually transmitted infection requiring treatment; chronic or recurrent vaginal candidiasis; significant hematologic or liver function test abnormalities; clinically apparent grade ≥2 gynecologic abnormalities; or gynecologic or urogenital procedure in the past 45 days (MTN-044/IPM 053/CCN019) or 60 days (MTN-030/IPM 041). MTN-030/IPM 041 excluded participants with BMI >35 kg/m^2^ and MTN-044/IPM 053/CCN019 excluded participants with BMI >40 kg/m^2^. In both trials, the enrollment visit was scheduled with consideration of the menstrual cycle with ideally no anticipated bleeding during the 14 days of study product use in MTN-030/IPM 041 and no anticipated bleeding during the first 3 days of study product use in MTN-044/IPM 053/CCN019.

### MTN-030/IPM 041–14-day DPV only vs. DPV/LNG ring

After providing written consent and completing screening, eligible participants were randomized to continuous 14-day use of either a ring containing 200mg DPV or 200mg DPV and 320mg LNG between May 2017 and August 2017. The ring was vaginally self-inserted at enrollment, followed by a clinician-performed exam to confirm placement. Blood and CVF were collected at enrollment prior to ring insertion and after 1-, 2-, 4-, and 6-hours, and on day 14 blood and CVF were collected immediately prior to and 6-hours following ring removal. At all other study visits (days 1, 2, 3, 7, 15, and 16), blood and CVF were obtained at a single timepoint for drug measurements (notably samples collected on days 15 and 16 assessed the elimination phase following ring removal on day 14). CVF was clinician-collected via a vaginal swab within 30 minutes of blood collection, and net weight of CVF was determined. Used rings were collected at day 14. Participants, all study team members, and pharmacists were blinded to randomized intervention and blinding was maintained until all final data were locked into the trial database and ready for analysis. All vaginal rings were individually packaged and labeled and multiple codes were utilized to conceal and protect randomization assignments and the identity of the content of the ring.

### MTN-044/IPM 053/CCN019–90 day continuous vs. cyclic DPV/LNG ring

After providing written consent and completing screening, eligible participants were randomized to a 200mg DPV and 320mg LNG ring used continuously (90 consecutive days) or cyclically (28 days ring in and 2 days ring out each cycle) for 90 days between July 2018 and May 2019. The ring was vaginally self-inserted at enrollment, followed by a clinician-performed exam to confirm placement. At all study visits (days 0, 2, 14, 28, 30, 44, 58, 60, 74, 90, 91, 92, and final visit on day 93–94), blood and CVF were obtained at a single timepoint for drug measurements (notably samples collected on days 91, 92, and 93–94 assessed the elimination phase following ring removal on day 90). Blood and CVF were collected prior to ring insertion at enrollment (all participants) and days 30 and 60 (cyclic use participants). Blood and CVF were collected prior to VR removal on day 90 (all participants) and on days 28 and 58 (cyclic use participants). CVF was clinician-collected via a vaginal swab within 30 minutes of blood collection and the CVF net weight was determined. Ectocervical tissue biopsies were collected at day 14 (ring in place for both study arms), day 30 (two days after day 28 ring removal for cyclic use participants), and day 90 (day of, but prior to, ring removal for all participants); net weights were immediately recorded and biopsies were then flash frozen in a dry ice/ethanol bath. For cyclic use participants, used rings were collected on days 28 and 58, rinsed with tap water and stored at the study site, and the same ring was reinserted on days 30 and 60. Used rings were collected from all participants at day 90 visits. This was an open-label trail with randomization to use pattern, which was not blinded.

### Sample and randomization

Both MTN-030/IPM 041 and MTN-044/IPM 053/CCN019 were designed to enroll approximately 24 participants randomized 1:1 using permuted block randomization. The randomization scheme was generated and maintained by the MTN Statistical Data Management Center and study investigators enrolled participants and assigned interventions dictated by the randomization scheme. Twelve participants in each study regimen would provide 89% power to yield detectable PK concentrations in at least 83% of participants if the true rate of detectable PK concentration was 90%, and if all 12 participants are observed to have detectable PK, the 95% exact 2-sided lower confidence bound for the true rate of PK detection is 74%. A total of 24 participants were randomized in MTN-030/IPM 041 and 25 participants were randomized in MTN-044/IPM 053/CCN019.

### Laboratory methods

DPV and LNG were quantified in plasma and CVF and DPV was quantified in cervical tissue using liquid chromatography-tandem mass spectrometry by the Clinical Pharmacology Analytical Laboratory at the Johns Hopkins University School of Medicine and the Small Molecule Biomarker Core laboratory at the University of Pittsburgh respectively as previously described [31,40,41]. A modified version of a previously published liquid chromatography-tandem mass spectrometry method that employed CVF extraction from Dacron swabs via a 1:1 solution of methanol:water, was used for DPV quantitation in CVF [42]. All assays were validated in accordance with FDA bioanalytical guidelines. The lower limits of quantification (LLOQ) for DPV in plasma, CVF, and cervical tissue were 20 pg/mL, 0.25 ng/swab, and 0.05 ng/sample respectively. When normalized to fluid or biopsy weights, median LLOQs were 0.005 ng/mg (interquartile range (IQR): 0.004–0.007 ng/mg) and 0.004 ng/mg (IQR: 0.003–0.006 ng/mg), respectively. The lower limits of quantification (LLOQ) for LNG in plasma and CVF were 0.25 ng/mL and 0.25 ng/swab respectively. When normalized to fluid weights the median LLOQ was 0.00034 ng/mg (interquartile range (IQR): 0.00027–0.00044 ng/mg).

### Pharmacokinetic and statistical analysis

For both trials, participant characteristics were summarized using descriptive statistics. For the safety objective, which included all evaluable participants who inserted the ring, we evaluated the proportion of participants with AE endpoints comparing the two study arms within each study using Fisher’s exact tests and 95% exact binomial confidence intervals for the event rates.

Plasma, CVF and cervical tissue (collected in MTN-044/IPM 053/CCN019 only) PK data are reported for all participants who inserted the ring even if product use was discontinued early. PK summary statistics including the peak concentration (C_max_) and time to peak concentration (T_max_) were determined, and the area under the concentration-time curves (AUC) in plasma and CVF were calculated from 0–14 days (AUC_0-14d_) for MTN-030/IPM 041 and 0–90 days (AUC_0-90d_) for MTN-044/IPM 053/CCN019 using the trapezoidal method [43]. In each trial, C_max_ and AUC for DPV and LNG in plasma and cervicovaginal fluid were each compared between study arms using a 2-sided exact Mann-Whitney U Test. Terminal half-life t_1/2_ was calculated for participants with at least 3 valid concentration time points in the elimination phase (after ring removal). Post-dose concentrations below the LLOQ of an assay were imputed with a value of zero for calculation of PK summary statistics and were imputed with a value of LLOQ/2 in the figures.

For MTN-044/IPM 053/CCN019, vaginal bleeding patterns were compared between study arms using a Chi-square Test (number of days with no bleeding, spotting/light, moderate, and heavy bleeding) and Fisher’s Exact Tests (any bleeding vs. no bleeding).

All statistical analyses were conducted using SAS (version 9.4, Cary, NC).

## Results

### MTN-030/IPM 041

**Fig 1:**
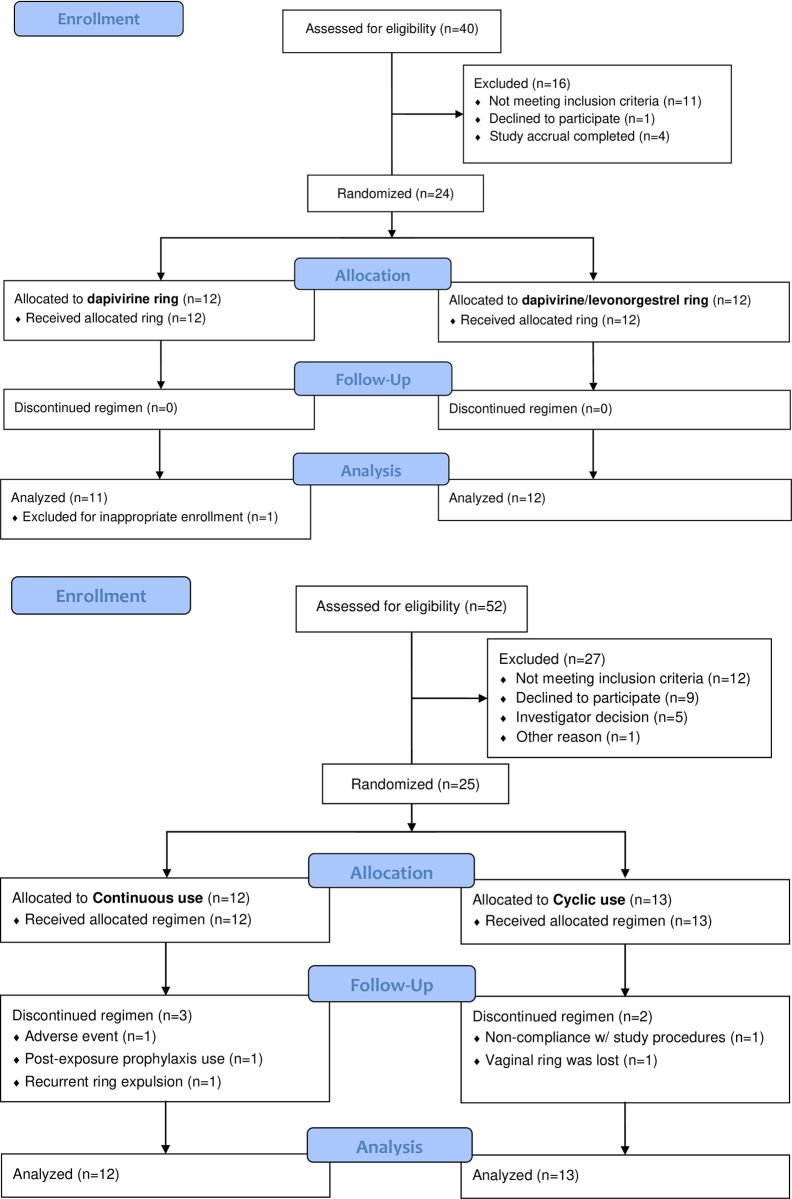
a. MTN-030/IPM 041 Study Flow Diagram. MTN-030/IPM 041 Study Flow Diagram. b. MTN-044/IPM 053/CCN019 Study Flow Diagram. MTN-044/IPM 053/CCN019 Study Flow Diagram.

Twenty-four participants enrolled at the Birmingham (n = 12) and Pittsburgh (n = 12) sites. Study flow and participant demographics are outlined in Fig 1A and Table 1, respectively. All enrolled participants completed the study. After enrollment, one participant, assigned to the DPV ring study product arm, was found to be inappropriately enrolled due to a positive chlamydia test at screening that would have made the participant ineligible, therefore all data associated with this participant were excluded from the analyses.

**Table 1:** Demographics of evaluable participants.

	MTN-030/IPM 041		MTN-044/IPM 053/CCN019	
	14-day DPV (n = 11)	14-day DPV/LNG (n = 12)	90-day DPV/LNG continuous (n = 12)	90-day DPV/LNG cyclic (n = 13)
Age, years	31 (22–41)	33 (20–43)	32 (22–43)	37 (21–43)
Body mass index (kg/m^2^)*	26 (19–35)	27 (19–34)	26 (20–39)	29 (24–39)
Race Black White Asian Asian and White American Indian or Alaska Native and White	2 (18%) 8 (73%) 0 1 (9%) 0	5 (42%) 6 (50%) 0 0 1 (8%)	2 (17%) 8 (67%) 2 (17%) 0 0	1(8%) 12 (92%) 0 0 0
Marital Status Never Married Married Divorced	8 (73%) 3 (27%) 0	9 (75%) 3 (25%) 0	10 (83%) 1 (8%) 1 (8%)	8 (62%) 5 (38%) 0
No Primary Sex Partner Primary Sex Partner^§^ Man Woman Other	2 (18%) 9 (82%) 7 (64%) 2 (18%) 0	6 (50%) 6 (50%) 4 (33%) 1 (8%) 1 (8%)	6 (50%) 6 (50%) 5 (42%) 1 (8%) 0	4 (31%) 9 (69%) 8 (62%) 0 1 (8%)

Median (range) or n (%). DPV = dapivirine. LNG = levonorgestrel.

*Body Mass Index >35 kg/m^2^ was exclusionary for enrollment in MTN-030/IPM 041 and >40 kg/m^2^ was exclusionary for enrollment in MTN-044/IPM 053/CCN019.

^§^Primary sex partner gender as reported by participants.

### MTN-044/IPM 053/CCN019

Twenty-five participants enrolled at the Pittsburgh site. Study flow and participant demographics are outlined in Fig 1B and Table 1, respectively. Four participants did not complete the study. In the continuous use arm, one participant withdrew after day 14 due to recurrent ring expulsion, one participant withdrew after day 30 due to sexual assault necessitating use of prohibited medication (HIV post-exposure prophylaxis), and one participant withdrew after day 44 due to vulvar contact dermatitis unrelated to study product. In the cyclic use arm, one participant withdrew at day 28 due to refusal of cervical biopsy. One participant in the cyclic arm at day 90 was found to have unknowingly expelled the ring. The vaginal fluid samples collected for DPV and LNG PK for two contemporaneously occurring participant visits (one day 0 and one day 28) were excluded from analyses due to a protocol deviation in handling these specimens. All other data for enrolled participants were included in the analyses.

### Safety assessment

#### MTN-030/IPM 041

A total of 43 AEs were reported in 16/23 (70%) participants, including 24 AEs in 9/12 (75%) participants in the DPV/LNG ring arm and 19 AEs in 7/11 (64%) participants in the DPV ring arm. Overall, 27/43 (63%) AEs were assessed as related to study product. All AEs were grade 1 (36/43 [84%]) or grade 2 (7/43 [16%]). We found no statistically significant difference in the proportion of participants with grade ≥2 genitourinary AEs in the DPV/LNG ring (0/12 [0%]) arm compared with the DPV ring arm (2/11 [18%]), p = .22.

#### MTN-044/IPM 053/CCN019

A total of 84 AEs were reported in 23/25 (92%) participants, including 43 AEs in 11/12 (92%) participants in the continuous use arm and 41 AEs in 12/13 (92%) participants in the cyclic use arm. Overall, 42/84 (50%) AEs were assessed as related to study product. Most AEs were grade 1 (59/84 [70%]) or grade 2 (24/84 [29%]). There were no grade 3 AEs. One grade 4 AE was reported in the cyclic use arm: symptomatic anemia, related to study product in a participant with a history of bariatric surgery and baseline anemia at enrollment, reporting heavy vaginal bleeding at day 90 and found to have had an unrecognized ring expulsion. We found no difference in the proportion of participants with grade ≥2 genitourinary AEs in the continuous use (3/12 [25%]) compared with the cyclic use arm (5/13 [38%]), p = .67, and no difference in the proportion of participants with grade ≥3 AEs in the continuous use (0/12 [0%]) compared with the cyclic use (1/13 [8%]) arm, p = 1.00.

### Pharmacokinetic analysis

**Fig 2:**
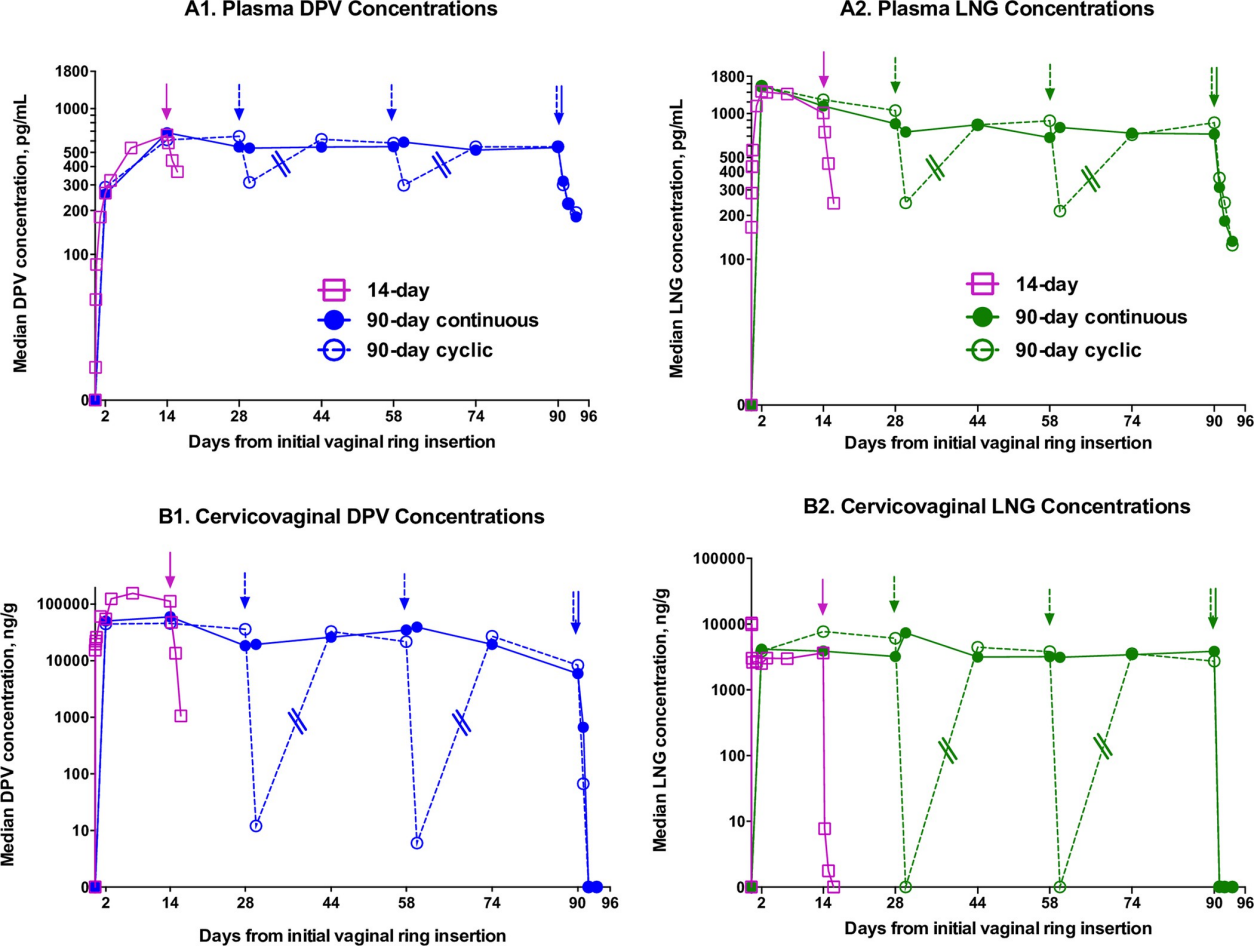
Concentration-time profiles for DPV and LNG in plasma and cervicovaginal fluid. Median Dapivirine (DPV) and Levonorgestrel (LNG) concentrations in (A) blood plasma (pg/ml) and (B) cervicovaginal fluid (ng/mg). MTN-030/IPM 041 study data are represented by pink squares and labeled as ‘14-day’ in the legend. MTN-044/IPM 053/CCN019 study data are represented by blue (DPV) and green (LNG) circles and labeled as ‘90-day continuous’ (solid circles with solid connecting lines) and ‘90-day cyclic’ (open circles with dashed connecting lines) in the legend. Given the sparse sampling protocol not designed to evaluate the pharmacokinetics of return to steady-state drug concentrations following reinsertion of rings in cyclic users after days 30 and 60, double slash symbols to represent line breaks were drawn on the connecting lines of the curves between sampling on days 30 and 44 in the cyclic group. Arrows depict timing of ring removals for each group.

Concentration-time profiles for DPV and LNG in plasma and CVF associated with use of the DPV/LNG ring in both trials (MTN-030/IPM 041 and MTN-044/IPM 053/CCN019) are shown in Fig 2. Median DPV and LNG concentrations in plasma and CVF associated with use of DPV-only and DPV/LNG rings in both trials, and median DPV and LNG concentrations in cervical tissue associated with use of DPV/LNG rings in the MTN-030/IPM 041 trial are summarized in Table 2. Pharmacokinetic parameters associated with ring use in both trials are further summarized in Table 3.

**Table 2:** Median DPV and LNG concentrations in plasma, cervicovaginal fluid (CVF), and cervical tissue.

	14-day DPV (n = 11) ^¶^^1^	14-day DPV/LNG (n = 12)^¶^		90-day DPV/LNG continuous (n = 12) ^§^^2^		90-day DPV/LNG cyclic (n = 13)^§^^2^	
Plasma Time Point^3^	DPV (pg/mL)	DPV (pg/mL)	LNG (ng/mL)	DPV (pg/mL)	LNG (ng/mL)	DPV (pg/mL)	LNG (ng/mL)
1 hr 2 hr 3 hr 4 hr 24 hr 48 hr 72 hr Day 7 Day 14 Day14+6hr Day15 Day16 Day 28 Day 30 Day 44 Day 58 Day 60 Day 74 Day 90 Day91 Day92 Day93‐94	BLQ (BLQ, BLQ) 25 (BLQ, 36) 56 (48, 96) 90 (75, 117) 210 (170, 257) 246 (217, 286) 327 (254, 394) 378 (281, 564) 499 (346, 656) 425(322,536) 258(176,458) 196(118,386)	BLQ (BLQ, BLQ) 17 (BLQ, 33) 49 (36, 81) 85 (59, 109) 181 (157, 254) 264 (192, 323) 322 (261, 630) 537 (396, 673) 661 (588, 804) 581(504,805) 441(315,530) 369(256,436)	0.17 (0.11, 0.19) 0.29 (0.20, 0.40) 0.43 (0.30, 0.64) 0.56 (0.43, 0.84) 1.13 (0.79, 1.82) 1.42 (1.03, 2.13) 1.40 (1.11, 2.61) 1.36 (0.95, 2.34) 1.01 (0.90, 1.61) 0.75(0.54,1.03) 0.45(0.40,0.50) 0.24(0.20,0.33)	260 (191, 345) 684 (530, 777) 546 (476, 744) 536 (428, 735) 544 (459, 680) 548 (435, 764) 589 (456, 705) 519 (475, 654) 541 (420, 572) 318(260,428) 220(206,287) 181(145,192)	1.56 (1.30, 2.33) 1.13 (0.97, 1.51) 0.85 (0.60, 1.29) 0.75 (0.70, 1.14) 0.84 (0.72, 1.10) 0.69 (0.63, 1.04) 0.80 (0.59, 1.05) 0.73 (0.60, 0.88) 0.72 (0.59, 0.87) 0.31(0.25,0.38) 0.18(0.14,0.23) 0.13(0.07,0.17)	289 (237, 325) 610 (584, 667) 645 (469, 741) 312 (214, 383) 616 (449, 695) 580 (446, 638) 298 (174, 394) 547 (436, 594) 549 (341, 571) 300(183,386) 223(136,328) 194(90,288)	1.54 (1.10, 2.50) 1.24 (0.88, 1.37) 1.05 (0.83, 1.20) 0.24 (0.23, 0.36) 0.84 (0.71, 1.21) 0.89 (0.70, 1.31) 0.21 (0.17, 0.31) 0.71 (0.63, 1.28) 0.86 (0.54, 1.16) 0.36(0.32,0.45) 0.25(0.17,0.30) 0.13(0.07,0.22)
CVF Time Point^3^	DPV (ng/mg)	DPV (ng/mg)	LNG (ng/mg)	DPV (ng/mg)	LNG (ng/mg)	DPV (ng/mg)	LNG (ng/mg)
1 hr 2 hr 4 hr 6 hr 24 hr 48 hr 72 hr Day 7 Day 14 Day14+6hr Day15 Day16 Day 28 Day 30 Day 44 Day 58 Day 60 Day 74 Day 90 Day91 Day92 Day93‐94	42.2 (29.0, 61.9) 57.6 (39.2, 75.6) 54.9 (47.4, 83.0) 50.1 (39.4, 65.6) 43.0 (38.7, 105) 38.8 (23.0, 74.9) 43.8 (26.5, 113) 31.6 (21.2, 82.2) 60.2 (38.0, 108) 30.4(7.3,74.7) 3.6(0.2,7.7) 0.4(<0.1,1.4)	15.4 (7.5, 22.8) 19.6 (5.8, 45.2) 22.3 (9.8, 42.9) 26.0 (13.0, 45.1) 60.3 (22.3, 65.6) 54.6 (28.7, 140) 124 (61.7, 172) 156 (111, 218) 113 (72.8, 167) 47.7(15.2,88.4) 13.7(0.4,18.1) 1.1(<0.1,5.7)	10.3 (2.5, 25.7) 9.8 (2.0, 19.8) 3.0 (0.6, 16.7) 2.6 (1.5, 3.9) 2.7 (1.6, 4.8) 2.5 (1.5, 4.0) 3.0 (1.2, 3.8) 3.0 (2.2, 3.9) 3.6 (2.7, 5.6) <0.1(<0.1,0.1) <0.1(<0.1,<0.1) BLQ(BLQ,<0.1)	50.3 (29.9, 63.0) 59.8 (32.6, 86.4) 18.4 (6,2, 74.4) 19.5 (9.4, 74.5) 26.1 (5.8, 76.8) 34.8 (4.5, 44.2) 39.2 (3.4, 59.7) 19.4 (4.2, 58.2) 5.9 (4.9, 41.3) 0.7(BLQ,4.0) BLQ(BLQ,0.4) BLQ(BLQ,BLQ)	4.1 (2.7, 5.4) 3.9 (2.0, 5.8) 3.2 (2.6, 7.2) 7.3 (2.4, 10.9) 3.2 (1.9, 6.4) 3.2 (1.1, 4.1) 3.1 (2.6, 4.4) 3.4 (2.6, 3.8) 3.9 (2.6, 5.1) <0.1(<0.1,0.1) BLQ(BLQ,BLQ) BLQ(BLQ,BLQ)	44.8 (34.5, 56.5) 45.6 (19.0, 66.0) 36.0 (7.9, 55.0) 0.01 (BLQ, 0.3) 32.9 (8.4, 41.7) 21.7 (7.3, 60.9) 0.01 (BLQ, 0.1) 27.0 (1.8, 44.4) 8.3 (2.9, 32.0) 0.1(<0.1,0.5) BLQ(BLQ,<0.1) BLQ(BLQ,BLQ)	3.9 (2.6, 5.2) 7.7 (3.9, 12.3) 6.1 (3.5, 16.5) BLQ (BLQ, BLQ) 4.4 (2.8, 7.5) 3.8 (2.1, 7.1) BLQ (BLQ, BLQ) 3.5 (2.3, 6.6) 2.7 (2.2, 4.5) <0.1(<0.1,<0.1) BLQ(BLQ,<0.1) BLQ(BLQ,BLQ)
Cervical Tissue Time Point				DPV (ng/mg)		DPV (ng/mg)	
Day 14 Day 30 Day 90				1.9 (0.9, 3.0) 1.6 (0.7, 7.9) 2.2 (BLQ, 3.3)		1.9 (0.9, 3.7) BLQ (BLQ, BLQ) 0.6 (BLQ, 2.6)	

Concentrations presented as median and IQR (1^st^ quartile, 3^rd^ quartile). CVF = cervicovaginal fluid. BLQ = below the limit of quantification. DPV = dapivirine. LNG = levonorgestrel.

^¶^ MTN-030/IPM 041 trial with participants randomized to 14-day DPV and 14-day DPV/LNG rings.

^§^ MTN-044/IPM 053/CCN019 trial with participants randomized to continuous and cyclic use of 90-day DPV/LNG rings.

^1^ Number of available CVF concentrations per time point vary for 14-day DPV (n = 11 for all time points except Day 14 and Day 15 (n = 10)).

^2^ Number of available plasma and CVF concentrations per time point varies. For 90-day DPV/LNG continuous cohort: n = 12 for 48hr & Day 14; n = 11 for Days 28 through 44; and n = 9 for Days 58 through 93–94, except for Day 91 (n = 8). For 90-day DPV/LNG cyclic cohort: n = 13 for 48hr & Day 14; n = 12 for Days 28 through 90, except for Day 30 (n = 11) and Day 28 for CVF only (n = 11); and n = 11 for Days 91 through 93–94. For cervical tissue, 90-day DPV/LNG continuous cohort has n = 12, 11, and 9 for Days 14, 30, and 90, respectively; 90-day DPV/LNG cyclic has n = 13, 11, 11 for Days 14, 30, and 90, respectively. All Day 0 pre-insertion plasma and CVF specimen results were BLQ for DPV and LNG after excluding a Day 0 CVF specimen result for one continuous arm participant and a Day 28 CVF specimen result for one cyclic arm participant due to a protocol deviation in handling of the two contemporaneous samples.

^3^ Grayed time points indicate the elimination phase after ring removal.

**Table 3:** PK Parameters for DPV and LNG concentrations in plasma and cervicovaginal fluid (CVF).

	MTN-030/IPM 041^1^			MTN-044/IPM 053/CCN019			
	14-day DPV (n = 11)	14-day DPV/LNG (n = 12)		90-day DPV/LNG continuous (n = 12)^2^		90-day DPV/LNG cyclic (n = 13)^2^	
PK Parameters	DPV	DPV	LNG	DPV	LNG	DPV	LNG
Plasma t_max_ (d) C_max_ (pg or ng/mL) AUC (ng*d/mL)^3^ t_1/2_ (d)	13.9 (7.2, 14.0) 499 (346, 698) 5.0 (3.8, 7.0) 1.1 (1.0, 2.1)	14.0 (13.9, 14.1) 661 (588, 845) 6.4 (5.3, 8.4) 2.1 (1.5, 2.2)	3.0 (2.1, 5.2) 1.64 (1.12, 2.61) 17.9 (13.6, 28.6) 1.1 (1.0, 1.2)	21.0 (14.0, 66.0) 750 (551, 813) 50.5 (44.7, 56.3) 2.1 (1.8, 2.5)	2.0 (2.0, 8.0) 1.68 (1.34, 2.33) 78.3 (71.3, 92.7) 1.2 (1.1, 1.3)	28.0 (14.0, 44.0) 673 (603, 796) 47.2 (36.9, 50.4) 2.4 (1.9, 2.7)	2.0 (2.0, 2.0) 1.54 (1.10, 2.50) 79.8 (61.1, 107) 1.3 (1.0, 1.5)
CVF t_max_ (d) C_max_ (ng/mg) AUC (μg*d/mg)^3^ t_1/2_ (d)	0.3 (0.1, 3.0) 107 (71.4, 113) 0.53 (0.44, 1.05) 0.3 (0.3, 1.0)	7.2 (7.0, 14.0) 183 (130, 233) 1.58 (0.87, 1.98) 0.3 (0.3, 1.1)	0.1 (0.1, 0.2) 19.8 (6.3, 31.3) 0.05 (0.02, 0.06) 0.3 (0.3, 0.3)	14.0 (8.0, 29.0) 78.9 (57.5, 95.0) 3.41 (1.78, 5.02) 0.4 (0.3, 0.9)	30.0 (21.0, 51.5) 9.0 (6.6, 20.5) 0.39 (0.35, 0.59) 0.2 (0.2, 0.2)	14.0 (2.0, 28.0) 66.0 (45.9, 74.4) 1.75 (1.30, 4.19) 0.2 (0.2, 0.3)	14.0 (14.0, 28.0) 8.0 (6.9, 15.5) 0.37 (0.18, 0.56) 0.2 (0.2, 0.2)

PK parameters presented as median and IQR (1^st^ quartile, 3^rd^ quartile). CVF = cervicovaginal fluid. AUC = area under the curve. d = day. ng = nanogram. μg = microgram. mL = milliliter. mg = milligram. Plasma C_max_ units: pg/mL for DPV; ng/mL for LNG.

^1^ Statistical significance and trends toward statistical significance are noted for comparisons of the DPV-only ring vs. DPV/LNG ring study arms for C_max_ of DPV in plasma (p = 0.049), C_max_ of DPV in CVF (p = 0.088), AUC of DPV in plasma (p = 0.091), and AUC of DPV in CVF (p = 0.118) by 2-sided exact test for Mann-Whitney U Test.

^2^ Number of participants with plasma and CVF PK summary parameters AUC and t_1/2_ varies. For 90-day DPV/LNG continuous cohort: n = 9 for AUC for both DPV and LNG in plasma and CVF; for t_1/2_, n = 9 for DPV and LNG in plasma, n = 3 for DPV in CVF, and n = 2 for LNG in CVF. For 90-day DPV/LNG cyclic cohort: n = 12 for AUC for both DPV and LNG in plasma and n = 11 for AUC for both DPV and LNG in CVF; for t_1/2_, n = 11 for DPV and LNG in plasma, n = 3 for DPV in CVF, and n = 4 for LNG in CVF.

^3^ For plasma and CVF, the period for AUC is AUC_0-14_ for the 14-day cohorts and AUC_0-90_ for the 90-day cohorts.

### DPV in plasma

After vaginal insertion of DPV-containing rings, plasma DPV concentrations were quantifiable by 2 hours after ring insertion in MTN-030/IPM 041 and progressively increased to a steady state concentration >200pg/mL by 48 hours. Median plasma DPV concentrations were similar 48 hours after ring insertion in both trials, including users of DPV-only and DPV/LNG rings (Table 2). Median maximum DPV concentrations (C_max_) were 499pg/mL and 661pg/mL in the DPV and DPV/LNG rings, respectively (p = 0.049).

In MTN-044/IPM 053/CCN019, the median t_max_ was 21 days and 28 days for plasma DPV concentrations in continuous and cyclic users, respectively. Median DPV C_max_ were 750pg/mL and 673pg/mL with continuous and cyclic ring use, respectively (p = 0.74). In participants randomized to cyclic ring use, wearing rings for 28 days followed by 2 ring-free days for each 30-day cycle, nadir median plasma DPV concentrations were 312pg/mL (IQR 214-383pg/mL) and 298pg/mL (IQR 174-394pg/mL) on days 30 and 60, respectively, representing a ~50% decrease in plasma concentration over 48 hours. The time-course of DPV return to steady state following reinsertion of rings in cyclic users was not captured with the sparsely sampled timepoints; DPV concentrations had returned to steady state at the next follow-up visit, 14 days after reinsertion. With the exception of days 30 and 60, plasma DPV concentrations were similar at all timepoints in continuous and cyclic users in MTN-044/IPM 053/CCN019, and AUCs were similar (50.5 vs. 47.2(ng*d/mL)^3^, respectively, p = 0.25). After terminal ring removal in all participants and sufficient data in the elimination phase (at day 14 in MTN-030/IPM 041, n = 11 & 12 for DPV and DPV/LNG arms respectively, and at day 90 in MTN-044/IPM 053/CCN019, n = 9 & 11 for continuous and cyclic arms respectively), plasma DPV concentrations decreased with a median t_1/2_ of 1.1–2.4 days.

### DPV in cervicovaginal fluid

After vaginal insertion of DPV-containing rings, CVF DPV concentrations were quantifiable at the earliest timepoint (1 hour) after ring insertion in MTN-030/IPM 041 and rapidly increased to a steady state concentration by 24 hours. Median CVF DPV concentrations trended higher in users of DPV/LNG rings compared to users of DPV-only rings in MTN-030/IPM 041 (median C_max_ 183 vs. 107ng/mg, p = 0.088; and median AUC 1.58 vs. 0.53(μg*d/mg)^3^, p = 0.118) and were similar in continuous compared to cyclic users of DPV/LNG rings in MTN-044/IPM 053/CCN019 at every time point except days 30 and 60 (median C_max_ 78.9 vs. 66.0ng/mg, respectively, p = 0.14; and median AUC 3.41 vs. 1.75(μg*d/mg)^3^, p = 0.37) (Table 2). In 90-day users of DPV/LNG rings (MTN-044/IPM 053/CCN019), the median t_max_ was 14 days for CVF DPV. In participants randomized to cyclic ring use, nadir median CVF DPV concentrations were 0.1ng/mg (IQR BLQ-0.3ng/mg) and 0.1ng/mg (IQR BLQ-0.1ng/mg) on days 30 and 60 respectively, representing a >99% decrease in CVF concentration over 48 hours. The concentration-time profile following reinsertion of rings in cyclic users was not captured due to sparse sampling. At day 90, median CVF DPV concentrations had decreased by 88% and 81% in continuous and cyclic users, respectively, compared to median CVF concentrations at day 2. After terminal ring removal in all participants and sufficient data in the elimination phase (at day 14 in MTN-030/IPM 041, n = 11 & 12 for DPV and DPV/LNG arms respectively, and at day 90 in MTN-044/IPM 053/CCN019, n = 3 & 3 for continuous and cyclic arms respectively), CVF DPV concentrations decreased with a median t_1/2_ of 0.2–0.4 days.

### DPV in cervical tissue (see Table 2)

Tissue concentrations were only captured in MTN-044/IPM 053/CCN019 at Days 14, 30 and 90. Median cervical tissue concentrations ranged from 1.6–2.2ng/mg with continuous use. While tissue concentrations were comparable at Day 14 between continuous and cyclic ring use, tissue concentrations at Days 30 and 90 (each two days after ring removal in the cyclic group) were at least 97% lower in the cyclic use group (Fig 2, Table 2). At Day 30, 1/11 (9%) cyclic ring use participants had a quantifiable concentration two days after ring removal, and at Day 90, quantifiable concentrations were observed in 5/9 (56%) continuous ring users and 8/11 (73%) cyclic ring users.

### LNG in plasma

After vaginal insertion of LNG-containing rings, plasma LNG concentrations were quantifiable at the earliest timepoint (1 hour) after ring insertion in MTN-030/IPM 041 and progressively increased to a steady state concentration >1ng/mL by 24hours. Plasma LNG concentrations were similar 48 hours after ring insertion in both trials (Table 2). The median t_max_ was 3 days and 2 days for plasma LNG concentrations in MTN-030/IPM 041 and MTN-044/IPM 053/CCN019 (both continuous and cyclic users) respectively. In participants randomized to cyclic ring use, wearing rings for 28 days followed by 2 ring-free days for each 30-day cycle, nadir plasma LNG concentrations were 0.24ng/mL (IQR 0.23–0.36ng/mL) and 0.21ng/mL (IQR 0.17–0.31ng/mL) on days 30 and 60 respectively, representing a ~75% decrease in plasma concentration over 48 hours. The time-course of LNG return to steady state following reinsertion of rings in cyclic users was not captured by the sampling timepoints; LNG concentrations had returned to steady state at the next follow-up visit, 14 days after reinsertion. With the exception of days 30 and 60, plasma LNG concentrations were similar at all timepoints in continuous and cyclic users in MTN-044/IPM 053/CCN019, and AUCs were similar (78.3 vs. 79.8(ng*d/mL)^3^, respectively, p = 1.0). After terminal ring removal in all participants and sufficient data in the elimination phase (at day 14 in MTN-030/IPM 041, n = 12 for DPV/LNG arm, and at day 90 in MTN-044/IPM 053/CCN019, n = 9 & 11 for continuous and cyclic arms respectively), plasma LNG concentrations decreased with a median t_1/2_ of 1.1–1.3 days.

### LNG in cervicovaginal fluid

After vaginal insertion of LNG-containing rings, CVF LNG concentrations had attained steady state concentrations at the earliest timepoint (1 hour) after ring insertion in MTN-030/IPM 041. Median CVF LNG concentrations were similar in continuous compared to cyclic users of DPV/LNG rings in MTN-044/IPM 053/CCN019 at every time point except days 30 and 60 (median C_max_ 9.0 vs. 8.0ng/mg, respectively, p = 0.97; and median AUC 0.39 vs. 0.37(μg*d/mL)^3^, p = 0.71) (Table 2). In 90-day users of DPV/LNG rings (MTN-044/IPM 053/CCN019), the median t_max_ was 30 days and 14 days for CVF LNG in continuous and cyclic users, respectively. In participants randomized to cyclic ring use, nadir median CVF LNG concentrations were BLQ 2 days following ring removal, representing a >99% decrease in CVF concentration over 48 hours. The time-course of LNG return to steady state following reinsertion of rings in cyclic users was not captured by the sampling timepoints; LNG concentrations had returned to steady state at the next follow-up visit, 14 days after reinsertion. At day 90, median CVF LNG concentrations had decreased by 5% and 30% in continuous and cyclic users respectively compared to median CVF concentrations at day 2. After terminal ring removal in all participants and sufficient data in the elimination phase (at day 14 in MTN-030/IPM 041, n = 12 for DPV/LNG arm, and at day 90 in MTN-044/IPM 053/CCN019, n = 2 & 4 for continuous and cyclic arms respectively), CVF LNG concentrations decreased with a median t_1/2_ of 0.2–0.3 days.

### Vaginal bleeding

In MTN-044/IPM 053/CCN019, the number of days with no bleeding, spotting/light, moderate, and heavy bleeding did not differ by use pattern as shown in Table 4 (p = 0.12). In both continuous and cyclic users, 89% experienced no bleeding to light bleeding (Table 4). Although there were no significant differences in the number of bleeding episodes or bleeding incidence rates for continuous compared to cyclic use of the ring, cyclic users experienced less or a trend toward less bleeding in the days following each ring reinsertion compared to continuous users: On days 34–37, 9/11 (82%) of continuous users and 4/12 (33%) of cyclic users reported any bleeding (p = 0.04) and on days 64–67, 6/9 (67%) of continuous users and 5/12 (42%) of cyclic users reported any bleeding (p = 0.39). In MTN-030/IPM 041, participants randomized to DPV/LNG rings had bleeding incidence of 3.05 per person-time of follow-up (95% CI 1.81–4.82) compared to 0.92 per person-time of follow-up (95% CI 0.30–2.16) in those randomized to DPV-only rings. There were no participant discontinuations in either trial due to bleeding.

**Table 4:** Bleeding experiences with continuous compared to cyclic dapivirine/levonorgestrel vaginal ring use.

	Continuous (n = 12)	Cyclic (n = 13)
Total days on study	967	1155
No bleeding	557 (58%)	670 (58%)
Spotting/light bleeding	303 (31%)	354 (31%)
Moderate bleeding	101 (10%)	111 (10%)
Heavy bleeding	6 (1%)	20 (2%)
Total bleeding episodes (n)	67	62
Bleeding incidence rate during product use (days/person-month)	12.6	12.3
Bleeding incidence rate after product use (days/person-month)	20.8	27.5

Data presented as number of days (%) unless otherwise indicated.

## Discussion

Cyclic use of hormonal contraceptives is common and is intended to optimize user bleeding experiences without compromising contraceptive efficacy. Further, since user-controlled products are commonly used with incomplete adherence [36,44,45], it is important to understand the tolerance that any user-controlled prevention product has for periodic removals. We hypothesized that cyclic use of DPV/LNG rings, designed for dual prevention of pregnancy and HIV over 90-days, would be associated with an improved bleeding profile compared to continuous use. Given that vaginal rings containing both DPV and LNG had not previously been used in humans, the initial, first in human study (MTN-030/IPM 041) was conservatively designed to evaluate a short exposure (14 days) comparing use of DPV-only rings to DPV/LNG rings as a demonstration of safety. The MTN-044/IPM 053/CCN019 trial was subsequently designed to test our hypothesis that cyclic use of the 90-day DPV/LNG ring may be associated with more favorable bleeding experiences compared to continuous use. Here we present the safety, pharmacokinetic, and user-reported bleeding results from both trials.

DPV/LNG rings designed for dual prevention of pregnancy and HIV were found to be well-tolerated without safety concerns when used continuously for 14 days compared to DPV-only rings and when used continuously compared to cyclically over 90 days. We found that observed local and systemic drug concentrations were relatively comparable to reported concentrations with use of other DPV and LNG-containing products, particularly understanding that these products have not been compared directly and thus variation due to laboratory analytics and sampling methods are expected. For DPV, with continuous use of DPV/LNG rings containing 200mg DPV, we found a maximal DPV plasma concentration (C_max_) of 750pg/mL and median plasma concentrations during steady state of 519-589pg/mL. Despite statistical differences in plasma DPV C_max_ between the DPV and DPV/LNG rings, the efficacy, and thus clinical significance, is believed to be associated not with peak, or time to peak concentrations, but rather by achieving a minimum effective concentration within 48 hours after ring placement; all of the rings studied in MTN-030/IPM 041 and MTN-044/IPM 053/CCN019 exceed such a minimum. Specifically, with cyclic use of DPV/LNG rings, we found a median nadir plasma DPV concentration two days following ring removal of 312pg/mL and 298pg/mL at day 30 and 60 respectively. These results are consistent with concentrations observed in the MTN-036/IPM 047 phase 1 safety and pharmacokinetics study evaluating the 200mg DPV vaginal ring over a 90-day period [35]. Further, the concentrations observed in both MTN-030/IPM 041 and MTN-044/IPM 053/CCN019 exceed the concentrations observed in 25mg DPV vaginal ring efficacy trials. In ASPIRE, plasma DPV concentrations >95pg/mL were highly correlated with both high adherence and residual ring DPV concentrations <23.5mg, itself correlated with reduction of HIV acquisition [23–25,46].

For LNG, there is a wide acceptable dosing range with respect to contraceptive efficacy and safety/tolerability. The minimal serum threshold for contraceptive efficacy depends on a variety of factors including formulation, dose, delivery route, and user characteristics, including body weight and sex hormone binding globulin levels[47]. Thus, the minimum serum threshold for contraceptive efficacy is imprecise, or ‘elusive’[47], however it can be conservatively surmised to be above approximately 225pg/mL as measured using mass spectrometry-based methods[48,49]. This compares to continuous use of DPV/LNG rings in this study in which we found a median maximal LNG serum concentration of 1680pg/mL and median serum concentrations during steady state of 690-850pg/mL. Contraceptive efficacy associated with progestin-based methods is generally primarily achieved by inhibition of ovulation, which is mediated by systemic (serum) progestin concentrations. Contraceptive efficacy may also be achieved to some degree by changes in cervical mucus characteristics that impede sperm from accessing the upper reproductive tract [50–52], which may be mediated by either systemic or local progestin concentrations. Hence, for contraceptive efficacy, the relative contributions of systemic and local progestin concentrations likely depend on dose and type of progestin in each compartment. Given the t_1/2_, two days following ring removal in cyclic users, we observed median serum concentrations of 240pg/mL and 210pg/mL at day 30 and 60 respectively, that may be near the minimal serum concentration threshold for contraceptive efficacy. Thus, careful investigation to determine maximal time possible for ring outage while maintaining contraceptive efficacy would be needed if a vaginally delivered LNG-based contraceptive were to move forward in product development.

This contrasts with use of DPV to prevent sexual transmission of HIV since the critical compartment (systemic vs. local) within which threshold drug concentrations must be maintained are not yet established. The observed short half-life of DPV in genital tract fluid suggest that cyclic use of the DPV/LNG ring allowing for two days of ring removal each month, may not be ideal for HIV prevention if efficacy depends on local drug concentrations given the unquantifiable/low concentrations of tissue DPV observed at Day 30. Although plasma DPV concentrations with continuous and cyclic 90-day use of DPV/LNG rings matched or exceeded those observed in efficacy trials, because the DPV plasma half-life appears much longer than the half-life in tissue, plasma is not a useful surrogate for tissue DPV concentrations or degree of protection during a dynamic period after ring removal. If the DPV concentration in tissue, at the point of HIV virion contact with CD4+ T-cells, is critical for prevention, then ring removals lasting two days may not be possible without impacting efficacy given median DPV tissue concentration below the LLOQ, a level well below concentrations measured with the ring in place. Shorter periods of ring removal may be acceptable, however shorter periods were not part of the sampling strategy in our study. Alternatively, the concentration-time curve in MTN-030/IPM 041 demonstrated a rapid rise in plasma and genital tract DPV concentration after ring insertion, which suggests DPV-mediated HIV prevention may be induced or restored relatively rapidly (within hours), and thus ring removals concurrent with sexual abstinence and insertion in anticipation of sexual activity may be an option. Nevertheless, for development of dual-use MPT products, the use-case needs to be aligned for both active ingredients, and an ‘on-demand’ use pattern is unlikely to work well for LNG, or other progestins, given the mechanism of action and the likely unfavorable implications on bleeding induced by irregular use.

Bleeding profiles with use of progestin-only contraceptives are critical to product acceptability and the bleeding profile and acceptability associated with use of this product will need to be carefully investigated in a larger phase trial. Contrary to our hypothesis, we found no differences in vaginal bleeding experiences associated with use pattern over 90 days, although the majority of participants experienced none or only light bleeding, and no participants discontinued due to bleeding. Notably, one participant in the cyclic use arm experienced a grade 4 anemia AE associated with heavy vaginal bleeding and who was found to have had an unrecognized ring expulsion. Given that the timing of ring expulsion in this participant is unknown, we cannot assess if the heavy bleeding was the result of ring expulsion or if ring expulsion was the result of heavy bleeding. No other participants experienced similar heavy bleeding following planned ring removals.

These studies have several limitations. First, we performed sparse sampling to create approximate concentration-time curves; more intensive sampling, particularly in the hours surrounding ring insertions and removals, would be required to accurately depict the PK parameters. This phenomenon is demonstrated by the more intensive sampling after initial ring insertion that was performed in MTN-030/IPM 041 compared to MTN-044/IPM 053/CCN019. Indeed, it is likely that the return to steady-state drug concentrations following ring reinsertion in cyclic users is similar to the concentration changes when the ring is first placed—far more rapid than that depicted in the sparse sampling curves shown in Fig 2 and is the reason for addition of line-break symbols to these curves. Secondly, tissue sampling was very limited and therefore only provides cross-sectional-like information on tissue DPV concentration without ability to evaluate changes in tissue concentration relative to ring insertions and removals. Specifically, there are no tissue samples to indicate rate of DPV decline after ring removal, greatly limiting our ability to estimate the length of time a ring may be removed and still maintain concentrations above those in previous studies associated with high levels of adherence, and which were associated with high levels of protection from HIV acquisition. Thirdly, we did not distinguish between spotting (defined as not requiring sanitary protection) and light bleeding in these studies. Therefore, it is possible that we may have missed an impact associated with cyclic use if the cyclic users experiencing spotting/light bleeding were mostly spotting and the continuous users experiencing spotting/light bleeding were mostly light bleeding. These studies also had several key strengths including assessment of periodic removals in a user-controlled prevention product, extended follow up for 13 weeks, and inclusion of participants with BMI >30, the latter being particularly important for LNG-based contraceptives [53,54].

In summary, in these Phase I trials, the extended duration DPV/LNG MPT vaginal rings were found to be well-tolerated. The DPV and LNG plasma PK findings associated with continuous and cyclic ring use were consistent with respective plasma drug concentrations associated with use of effective LNG-based contraceptive and DPV-based HIV prevention products. Genital tract DPV concentrations associated with continuous use were similarly consistent, however, genital tract DPV concentrations were not sustained following ring removal and may be fundamental to HIV prevention as has been demonstrated with continuous use of the monthly 25mg DPV vaginal ring[23,24]. Gaining clear understandings of both the critical compartment and threshold DPV concentration within that compartment for sexual HIV prevention efficacy are needed to better assess the potential tolerance or intolerance of this product for periodic use. These MPTs have potential as a novel contraceptive option for women globally since they are user-controlled, long-acting, and have the addition of discrete HIV prevention, which can increase capacity and equity to achieve reproductive health goals without stigma.

## Supporting information

S1 Checklist (DOCX)

S1 Appendix Protocol amendments. Detailed listing of changes to the original protocols and planned statistical analyses for MTN-030/IPM 041 and MTN-044/IPM 053/CCN019.
(DOCX)

S1 File (DOCX)

S2 File (PDF)
