# Supplementary material for: Phase 1 randomized trials to assess safety, pharmacokinetics, and vaginal bleeding associated with use of extended duration dapivirine and levonorgestrel vaginal rings
Source: PLoS One. 2024 Jun 5;19(6):e0304552. doi: 10.1371/journal.pone.0304552 (PMC11152307; doi:10.1371/journal.pone.0304552)
Supplement: S1 Appendix — Detailed listing of changes to the original protocols and planned statistical analyses for MTN-030/IPM 041 and MTN-044/IPM 053/CCN019. (DOCX) [file pone.0304552.s002.docx]

**S1 Appendix. Protocol Amendments**

**Changes to the Original MTN-030/IPM 041 Protocol**

Changes from Version 1.0 dated 06 April 2016, are listed below:

• A planned investigational product, a vaginal ring containing dapivirine 200

mg/levonorgestrel 32 mg (Dapivirine-Levonorgestrel Vaginal Ring-101) was

removed from the protocol, resulting in several changes in the document, including a

change in treatment assignment ratio from 1:1:1 to 1:1, a reduction in sample size

from 36 to 24, and a reduction in trial duration from 8 to 10 months to 6 to 8 months.

• New, clarified or updated data regarding dapivirine and dapivirine/levonorgestrel

rings, films, and gels, as well as over-the-counter levonorgestrel products were added.

• Use of CYP3A inhibitors and inducers, antibiotics, and corticosteroids was no longer

allowed.

• Other information was added/updated for clarity:

− Information on the risks of toxic shock syndrome was added;

− Co-enrollment guidelines were updated to allow participation in observational

and/or interventional studies for pregnant and HIV-infected participants;

− For participants who became pregnant and did not enroll in MTN-016, language

was added to indicate that the research centers would have made every reasonable

effort to contact participants and collect infant outcome at approximately 1 year

after delivery for those pregnancies that resulted in live birth;

− Language was clarified regarding PK sample collection for participants who

permanently discontinued IP use for reasons other than pregnancy or HIV

infection;

− Additional guidance was incorporated on use and handling of leftover samples;

− Language was modified to clarify that behavioral procedures at some visits would

include a data convergence interview;

− Language was incorporated regarding asymptomatic BV, asymptomatic

candidiasis, and fetal losses;

− Language was incorporated stating that the data management system utilized was

compliant with US-EU Safe Harbor, the EU Data Protection Directive 95/46/EC,

ICH GCP, and CFR requirements.

**Changes in the Planned Statistical Analysis**

The original (Statistical Analysis Plan (SAP), Version 1.0 dated 11 January 2018, was updated to Version 2.0 dated 15 October 2018, and later to Version 3.0 dated 08 April 2019.

Changes from the SAP Version 1.0 dated 11 January 2018 to Version 2.0 dated 15 October 2018,

are summarized below:

• A tabulation of the number of participants enrolled by group was not needed and was

removed (SAP Version 2.0, Section 2.1.1 Study Enrollment).

• Person-months (calculated as number of days divided by 30.5) would be reported

instead of person-years due to the short trial duration and the monthly nature of

vaginal bleeding (SAP Version 2.0, Section 2.3.1 Secondary Analysis of Vaginal

Bleeding and Section 2.5 Exploratory Evaluation of Vaginal Bleeding Following

Vaginal Ring Removal).

Changes from the SAP Version 2.0 dated 15 October 2018 to Version 3.0 dated

08 April 2019 included clarifications added to the SAP, and are summarized below:

• Percentages for each discontinuation reason were included in the tables (SAP

Version 3.0, Sections 2.1.3 Treatment Discontinuation and Section 2.1.4 Study

Discontinuation).

• Analysis of Variance (ANOVA) was added to the PK primary analysis (SAP Version

3.0, Section 2.2.1 Primary Analysis of Pharmacokinetics).

• The MedDRA AE tables would be summarized overall, as well as per trial group,

including percentages of participants with AEs (SAP Version 3.0, Section 2.6

Additional Adverse Event [AE] Analysis).

**Changes to the Original MTN-044/IPM 053/CCN019 Protocol:**

The original Clinical Trial Protocol, Version 1.0, dated 20 February 2018, was submitted to the FDA on 05 March 2018. Clinical Trial Protocol Version 2.0, dated 07 June 2018, was developed

to clarify two inclusion criteria, to add information on condom compatibility studies, and to update the HIV testing algorithm. The protocol was submitted to the FDA on 28 June 2018. Note that Clinical Trial Protocol Version 2.0, dated 07 June 2018 was implemented prior to opening the trial for enrollment and no further protocol modifications were made once enrollment commenced.

Changes from Version 1.0, dated 20 February 2018, are listed below:

• Inclusion criteria 7 and 13 were revised to clarify the use of sex toys.

• The use of SMS text messages as the method for collecting vaginal bleeding and product adherence data during the trial was clarified.

• Results of condom compatibility tests, in which male and female condoms were exposed to gels containing dapivirine and levonorgestrel, were included in the Introduction section.

• The section on SAE reporting was updated.

• The summary of AEs considered possibly related to the dapivirine-levonorgestrel vaginal ring was updated based on preliminary results from MTN-030/IPM 041.

• The section on participant compensation was updated to make provision for medical treatment or compensation for medical expenses for treatment of any illness or injury resulting from participation in the trial.

• The symbol and footnote used for cervical biopsy collection at Visit 11 (Day 90, PUEV/Early termination) were corrected in Appendix I.

• The HIV testing algorithm in Appendix II was updated.

Two clarification memoranda were developed for Clinical Trial Protocol Version 2.0, dated 07 June 2018:

• Clarification Memorandum #01, dated 03 July 2018, was written to clarify the SAE reporting process described in Clinical Trial Protocol Version 2.0, Section 8.4.1; to update the text in the sample ICF about the provision of trial results on the ClinicalTrials.gov website; and to clarify the signing parties for the Clinical Trial Agreement for the trial in Sections 13.3 and 14 of the protocol.

• Clarification Memorandum #02, dated 20 November 2018, was written to clarify the guidelines for ring removal at the time of cervical biopsy sample collection at Visit 11 (Day 90, PUEV/Early termination).

**Changes in the Planned Statistical Analysis**

No changes were made to the original SAP Version 1.0, dated 13 March 2020.
