# Supplementary material for: Phase 1 randomized trials to assess safety, pharmacokinetics, and vaginal bleeding associated with use of extended duration dapivirine and levonorgestrel vaginal rings
Source: PLoS One. 2024 Jun 5;19(6):e0304552. doi: 10.1371/journal.pone.0304552 (PMC11152307; doi:10.1371/journal.pone.0304552)
Supplement: S1 File — (DOCX) [file pone.0304552.s003.docx]

**MTN-044/IPM 053/CCN019**

**A Randomized, Phase 1, Open-Label Study in Healthy HIV-Negative Women to Evaluate the Pharmacokinetics, Safety and Bleeding Patterns Associated with 90-Day Use of Matrix Vaginal Rings Containing 200 mg Dapivirine and 320 mg Levonorgestrel**

**Microbicide Trials Network**

**Funding Agencies:**

**US Eunice Kennedy Shriver National Institute of**

**Child Health and Human Development**

**Division of AIDS, US National Institute of Allergy and Infectious Diseases**

**US National Institute of Mental Health**

**US National Institutes of Health**

**Grant Number:**

**5UM1 AI068633**

**IND Sponsor:**

**International Partnership for Microbicides**

**IND #: 126907**

**Protocol Chair:
Sharon L. Achilles, MD, PhD**

**Protocol Co-Chair:**

**Beatrice A. Chen, MD, MPH**

**Version 2.0**

**June 7, 2018**

**MTN-044/IPM 053/CCN019**

**A Randomized, Phase 1, Open-Label Study in Healthy HIV-Negative Women to Evaluate the Pharmacokinetics, Safety and Bleeding Patterns Associated with 90-Day Use of Matrix Vaginal Rings Containing 200 mg Dapivirine and 320 mg Levonorgestrel**

**IND #: 126907**

**PROTOCOL SIGNATURE PAGE**

**Funding Agencies:**________________________________________________ Diana Blithe, PhD Date NICHD Program Chief, Contraceptive Development Program

________________________________________________ Jill Long, MD, MPH, MHS Date NICHD CCTN Medical Monitor, Contraceptive Development Program

________________________________________________ Jeanna Piper, MD Date DAIDS Senior Medical Officer

________________________________________________ Sharon Hillier, PhD Date MTN Principal Investigator

**IND Holder:** ____________________________________________________

Annaléne Nel, PhD, MD Date IPM Executive Vice President, Chief Medical Officer

**Protocol Chair:**________________________________________________ Sharon L. Achilles, MD, PhD Date Magee-Womens Hospital of UPMC

**Protocol Co-Chair:**________________________________________________ Beatrice A. Chen, MD, MPH Date Magee-Womens Hospital of UPMC

**Faculty Statistician:**________________________________________________ Barbra Richardson, PhD Date Faculty Statistician, FHCRC-SCHARP

**MTN-044/IPM 053/CCN019**

**A Randomized, Phase 1, Open-Label Study in Healthy HIV-Negative Women to Evaluate the Pharmacokinetics, Safety and Bleeding Patterns Associated with 90-Day Use of Matrix Vaginal Rings Containing 200 mg Dapivirine and 320 mg Levonorgestrel**

TABLE OF CONTENTS

[LIST OF ABBREVIATIONS AND ACRONYMS 7](#_Toc516220986)

[PROTOCOL TEAM ROSTER 10](#_Toc516220987)

[PROTOCOL SUMMARY 22](#_Toc516220988)

[1 KEY ROLES 25](#_Toc516220989)

[1.1 Protocol Identification 25](#_Toc516220990)

[1.2 Funding Agencies, Sponsor and Monitor Identification 25](#_Toc516220991)

[1.3 Laboratory Centers 26](#_Toc516220992)

[1.4 Data Center 26](#_Toc516220993)

[1.5 Study Implementation 26](#_Toc516220994)

[2 INTRODUCTION 27](#_Toc516220995)

[2.1 Microbicides, HIV Prevention and Contraception 27](#_Toc516220996)

[2.2 Dapivirine 29](#_Toc516220997)

[2.3 Nonclinical Studies of Dapivirine 30](#_Toc516220998)

[2.4 Clinical Studies of Dapivirine 32](#_Toc516220999)

[2.5 Levonorgestrel 42](#_Toc516221000)

[2.6 Nonclinical Studies of Levonorgestrel 43](#_Toc516221001)

[2.7 Clinical Studies of Levonorgestrel 44](#_Toc516221002)

[2.8 Studies of Levonorgestrel in Combination with Dapivirine 47](#_Toc516221003)

[2.9 Study Hypotheses and Rationale for Study Design 49](#_Toc516221004)

[3 OBJECTIVES 50](#_Toc516221005)

[3.1 Primary Objective 50](#_Toc516221006)

[3.2 Secondary Objectives 50](#_Toc516221007)

[3.3 Exploratory Objectives: 50](#_Toc516221008)

[4 STUDY DESIGN 51](#_Toc516221009)

[4.1 Identification of Study Design 51](#_Toc516221010)

[4.2 Primary Endpoints 51](#_Toc516221011)

[4.3 Secondary Endpoints 51](#_Toc516221012)

[4.4 Exploratory Endpoints: 51](#_Toc516221013)

[4.5 Description of Study Population 52](#_Toc516221014)

[4.6 Time to Complete Accrual 52](#_Toc516221015)

[4.7 Study Groups 52](#_Toc516221016)

[4.8 Expected Duration of Participation 52](#_Toc516221017)

[4.9 Site 52](#_Toc516221018)

[5 STUDY POPULATION 52](#_Toc516221019)

[5.1 Selection of the Study Population 52](#_Toc516221020)

[5.2 Inclusion Criteria 53](#_Toc516221021)

[5.3 Exclusion Criteria 55](#_Toc516221022)

[5.4 Co-enrollment Guidelines 57](#_Toc516221023)

[6 STUDY PRODUCT 57](#_Toc516221024)

[6.1 Regimen 57](#_Toc516221025)

[6.2 Administration 58](#_Toc516221026)

[6.3 Study Product Formulation 58](#_Toc516221027)

[6.4 VR Storage and Dispensing 58](#_Toc516221028)

[6.5 Supply and Accountability 58](#_Toc516221029)

[6.6 VR Use Instructions 59](#_Toc516221030)

[6.7 Concomitant Medications 59](#_Toc516221031)

[6.8 Prohibited Medications 60](#_Toc516221032)

[6.9 Use of Intravaginal Medications/Products and Practices 60](#_Toc516221033)

[7 STUDY PROCEDURES 60](#_Toc516221034)

[7.1 Pre-screening 61](#_Toc516221035)

[7.2 Screening Visit - Visit 1 61](#_Toc516221036)

[7.3 Enrollment Visit - Visit 2 (Day 0) 62](#_Toc516221037)

[7.4 Follow-up Visits 64](#_Toc516221038)

[7.5 Follow-up Procedures for Participants Who Permanently Discontinue Study Product 71](#_Toc516221039)

[7.6 Interim Visits 73](#_Toc516221040)

[7.7 Protocol Counseling: Adherence and Contraception Counseling 73](#_Toc516221041)

[7.8 Pharmacokinetics 73](#_Toc516221042)

[7.9 Behavioral Measures 74](#_Toc516221043)

[7.10 Clinical Evaluations and Procedures 75](#_Toc516221044)

[7.11 Laboratory Evaluations 76](#_Toc516221045)

[7.12 Specimen Management 77](#_Toc516221046)

[7.13 Laboratory Oversight 77](#_Toc516221047)

[7.14 Biohazard Containment 77](#_Toc516221048)

[8 ASSESSMENT OF SAFETY 78](#_Toc516221049)

[8.1 Safety Monitoring 78](#_Toc516221050)

[8.2 Clinical Data and Safety Review 78](#_Toc516221051)

[8.3 Adverse Events Definitions and Reporting Requirements 79](#_Toc516221052)

[8.4 Adverse Event Reporting Requirements 81](#_Toc516221053)

[8.5 Pregnancy and Pregnancy Outcomes 83](#_Toc516221054)

[8.6 Regulatory Requirements 84](#_Toc516221055)

[8.7 Social Harms Reporting 84](#_Toc516221056)

[9 CLINICAL MANAGEMENT 84](#_Toc516221057)

[9.1 Grading System 84](#_Toc516221058)

[9.2 Dose Modification Instructions 84](#_Toc516221059)

[9.3 General Criteria for Temporary Hold and Permanent Discontinuation of Study Product 85](#_Toc516221060)

[9.4 Response to Adverse Events 85](#_Toc516221061)

[9.5 Sexually Transmitted Infection/Reproductive Tract Infection 86](#_Toc516221062)

[9.6 Management of Specific Genital Events 86](#_Toc516221063)

[9.7 HIV Infection 87](#_Toc516221064)

[9.8 Pregnancy 87](#_Toc516221065)

[9.9 Criteria for Early Termination of Study Participation 87](#_Toc516221066)

[10 STATISTICAL CONSIDERATIONS 88](#_Toc516221067)

[10.1 Overview and Summary of Design 88](#_Toc516221068)

[10.2 Primary Endpoints 88](#_Toc516221069)

[10.3 Study Hypotheses 89](#_Toc516221070)

[10.4 Sample Size and Power Calculations 89](#_Toc516221071)

[10.5 Participant Accrual, Follow-up and Retention 91](#_Toc516221072)

[10.6 Randomization 91](#_Toc516221073)

[10.7 Blinding 91](#_Toc516221074)

[10.8 Data and Safety Monitoring and Analysis 91](#_Toc516221075)

[11 DATA HANDLING AND RECORDKEEPING 93](#_Toc516221076)

[11.1 Data Management Responsibilities 93](#_Toc516221077)

[11.2 Source Documents and Access to Source Data/Documents 93](#_Toc516221078)

[11.3 Quality Control and Quality Assurance 94](#_Toc516221079)

[12 CLINICAL SITE MONITORING 94](#_Toc516221080)

[13 HUMAN SUBJECTS PROTECTIONS 95](#_Toc516221081)

[13.1 Institutional Review Boards/Ethics Committees 95](#_Toc516221082)

[13.2 Protocol Registration 95](#_Toc516221083)

[13.3 Study Coordination 96](#_Toc516221084)

[13.4 Risk Benefit Statement 97](#_Toc516221085)

[13.5 Informed Consent Process 101](#_Toc516221086)

[13.6 Participant Confidentiality 101](#_Toc516221087)

[13.7 Special Populations 102](#_Toc516221088)

[13.8 Compensation 103](#_Toc516221089)

[13.9 Communicable Disease Reporting 103](#_Toc516221090)

[13.10 Access to HIV-related Care 103](#_Toc516221091)

[13.11 Study Discontinuation 103](#_Toc516221092)

[14 PUBLICATION POLICY 104](#_Toc516221093)

[15 APPENDICES 105](#_Toc516221094)

[APPENDIX I: SCHEDULE OF STUDY VISITS AND EVALUATIONS 106](#_Toc516221095)

[APPENDIX II: ALGORITHM FOR HIV TESTING FOR SCREENING AND ENROLLED PARTICIPANTS 109](#_Toc516221096)

[REFERENCE LIST 110](#_Toc516221097)

**Table of Figures**

[Figure 1: Study Visit Schedule 23](#_Toc516221098)

[Figure 2: Mean Vaginal Fluid Concentration-Time Curves of Dapivirine (ng/g) after Insertion of the Dapivirine Vaginal Ring-004 for 7, 14, 28, 56, or 84 Days (Groups A, B, C, D, and E): Mean ± SD Values 35](#_Toc516221099)

[Figure 3: Mean Plasma Concentration-Time Curves of Dapivirine (pg/mL) after Insertion of the Dapivirine Vaginal Ring-004 for 7, 14, 28, 56, or 84 Days (Groups A, B, C, D, and E): Mean ± SD Values 35](#_Toc516221100)

[Figure 4: Study Visit Schedule 61](#_Toc516221101)

**Table of Tables**

[Table 1: Phase 1-3 Clinical Trials of Dapivirine Vaginal Rings 33](#_Toc516221102)

[Table 2: Pharmacokinetics of Dapivirine in Plasma and Vaginal Fluids (collected at the cervix) after Insertion of the Dapivirine Vaginal Ring-004 for 7, 14, 28, 56, or 84 Days (Groups A, B, C, D, and E) 36](#_Toc516221103)

[Table 3: Incidence of Treatment-Emergent Adverse Events Reported Most Frequently (Incidence ≥ 10% in Participants Using DPV VRs), Regardless of Causality in IPM 027 40](#_Toc516221104)

[Table 4: Adverse Events in MTN-020 (ASPIRE) 41](#_Toc516221105)

[Table 5: Pharmacokinetics of Dapivirine and Levonorgestrel in Plasma and Vaginal Fluid Following Vaginal Administration to Sheep 47](#_Toc516221106)

[Table 6: Study Regimen 57](#_Toc516221107)

[Table 7: Retrieval of VR 59](#_Toc516221108)

[Table 8: Screening Visit - Visit 1 62](#_Toc516221109)

[Table 9: Enrollment Visit - Visit 2 (Day 0) 63](#_Toc516221110)

[Table 10: Day 2 and Day 14 - Visits 3 and 4 64](#_Toc516221111)

[Table 11: Day 28 and Day 58 - Visits 5 and 8 65](#_Toc516221112)

[Table 12: Day 30 and Day 60 - Visits 6 and 9 66](#_Toc516221113)

[Table 13: Day 44 and Day 74 – Visits 7 and 10 67](#_Toc516221114)

[Table 14: PUEV/Early Study Termination Visit - Day 90 – Visit 11 68](#_Toc516221115)

[Table 15: Day 91, Day 92 and Day 93 or 94 – Visits 12, 13 and 14 69](#_Toc516221116)

[Table 16: Final Phone Calls/Contacts – 1, 4, 8, and 12 Weeks After Ring Removal – Visits 15, 16, 17 and 18 71](#_Toc516221117)

[Table 17: Specimen Collection Schedule 73](#_Toc516221118)

[Table 18: Analysis of PK Event Frequency 89](#_Toc516221119)

[Table 19: Precision of Exact 2-sided 95% CIs for Observed Event Rates 89](#_Toc516221120)

[Table 20: Power for Minimum Detectable Differences in Drug Concentrations for Parallel Group Comparisons of Continuous Ring Users and Two Days Following Ring Removal for Cyclic Ring Users 90](#_Toc516221121)

[Table 21: Analysis of Safety Event Frequency 91](#_Toc516221122)

**MTN-044/IPM 053/CCN019**

**A Randomized, Phase 1, Open-Label Study in Healthy HIV-Negative Women to Evaluate the Pharmacokinetics, Safety and Bleeding Patterns Associated with 90-Day Use of Matrix Vaginal Rings Containing 200 mg Dapivirine and 320 mg Levonorgestrel**

# LIST OF ABBREVIATIONS AND ACRONYMS

ADR adverse drug reaction

AE adverse event

AIDS Acquired Immunodeficiency Syndrome

ALT alanine aminotransferase

ARR after ring removal

ARV antiretroviral

ASPIRE A Study to Prevent Infection with a Ring for Extended Use

AST aspartate aminotransferase

AUC area under the curve

b.i.d. *bis in die* (twice daily)

BRWG Behavioral Research Working Group

BSWG Biomedical Science Working Group

BV bacterial vaginosis

CASI computer assisted self-interview

CBC complete blood count

CCR5 C-C chemokine receptor type 5

CCTN Contraceptive Clinical Trials Network

CDC Centers for Disease Control and Prevention

CDP Contraceptive Development Program

CFR Code of Federal Regulations

C_max_ maximum concentration

CMRB Clinical Microbicide Research Branch

CONRAD Contraception Research And Development

CRF case report form

CRMS Clinical Research Management System

CROI Conference on Retroviruses and Opportunistic Infections

CRS clinical research site

CT *Chlamydia trachomatis,* chlamydia

CTA Clinical Trial Agreement

CV coefficient of variation

CVF cervicovaginal fluid

CVL cervicovaginal lavage

CWG Community Working Group

CXCR4 C-X-C chemokine receptor type 4

DAIDS Division of AIDS

DLV delavirdine

DNA deoxyribonucleic acid

DPV dapivirine

EC Ethics Committee

EC_50_ median effective concentration

EDC electronic data capture

EFV efavirenz

EMBRACE Evaluation of Maternal and Baby Outcome Registry After Chemoprophylactic Exposure

ENR Enrollment

FAME Film Antiretroviral Microbicide Evaluation

FDA (US) Food and Drug Administration

FHCRC Fred Hutchinson Cancer Research Center

g grams

GC *Neisseria gonorrhoeae,* gonorrhea

GCP Good Clinical Practices

GMP Good Manufacturing Practices

HD Health Decisions

HEENT Head, Eye, Ear, Nose and Throat

HHS (US) Department of Health and Human Services

HIV Human Immunodeficiency Virus

HPV human papillomavirus

HSV herpes simplex virus

hu-PBL human peripheral blood lymphocytes

hu-SCID humanized severe combined immunodeficiency

IATA International Air Transport Association

IB Investigator’s Brochure

ICF informed consent forms

ICH International Council for Harmonization

ICRC International Committee of the Red Cross

IDI in-depth interview

IND Investigational New Drug

IoR Investigator of Record

IP Investigational Product

IPM International Partnership for Microbicides

IRB Institutional Review Board

IUD intrauterine device

KOH potassium hydroxide

LC (MTN) Laboratory Center

LDMS Laboratory Data Management System

LLOQ lower limit of quantification

LNG levonorgestrel

LOC (MTN) Leadership and Operations Center

μg microgram

μM micromole

m meter

mg milligram

MIV-160 (-)-cis-1-(5-Cyanopyridin-2-yl)-3-(4,7-diflouro-1,1a,2,7b-tetrahydrocyclopropa[c]chromen-

1-yl)-urea, MSR-216; an NNRTI

mL milliliter

mm millimeter

MO Medical Officer

MPT Multipurpose Prevention Technologies

MTD maximum tolerated dose

MTN Microbicide Trials Network

NAAT nucleic acid amplification test

ng nanogram

NIAID National Institute of Allergy and Infectious Diseases

NICHD *Eunice Kennedy Shriver* National Institute of Child Health and Human Development

NIH National Institutes of Health

NIMH National Institute of Mental Health

nM nanomole

NNRTI non-nucleoside reverse transcriptase inhibitor

NOAEL no observed adverse effect level

NOEL no observed effect level

NVP nevirapine

OHRP Office for Human Research Protections

PD pharmacodynamics

PEP post-exposure prophylaxis

pg picogram

PID pelvic inflammatory disease

PK pharmacokinetics

PoR Pharmacist of Record

PrEP pre-exposure prophylaxis

PSP Prevention Sciences Program

PSRT Protocol Safety Review Team

PTID participant identification

PUEV product use end visit

PVI penile-vaginal intercourse

RBA relative binding affinity

RE Regulatory Entity

RF rectal fluid

RNA ribonucleic acid

RSC Regulatory Support Center

RT reverse transcriptase

RTI reproductive tract infection

SAE serious adverse event

SCHARP Statistical Center for HIV/AIDS Research & Prevention

SCR Screening

SDMC Statistical Data Management Center

SHBG sex hormone binding globulin

SRH sexual and reproductive health

SMC Study Monitoring Committee

SOC System Organ Class

SOP standard operating procedure

SSP study specific procedures

STI sexually transmitted infection

SUSARs suspected, unexpected serious adverse reactions

TFV tenofovir

TEAE treatment-emergent adverse events

TMC-120 dapivirine

UNAIDS United Nations Programme on HIV/AIDS

UPMC University of Pittsburgh Medical Center

USA United States of America

UTI urinary tract infection

VR vaginal ring

WHO World Health Organization

**MTN-044/IPM 053/CCN019**

**A Randomized, Phase 1, Open-Label Study in Healthy HIV-Negative Women to Evaluate the Pharmacokinetics, Safety and Bleeding Patterns Associated with 90-Day Use of Matrix Vaginal Rings Containing 200 mg Dapivirine and 320 mg Levonorgestrel**

# PROTOCOL TEAM ROSTER

**Protocol Chair**

**Sharon L. Achilles, MD, PhD, FACOG**

Magee-Womens Hospital of UPMC

300 Halket Street

Pittsburgh, PA 15213 USA

Phone: 412-641-1403

Fax: 412-641-1133

Email: achisx@upmc.edu

**Protocol Co-Chair**

**Beatrice A. Chen, MD, MPH**

Magee-Womens Hospital of UPMC

300 Halket Street

Pittsburgh, PA 15213 USA

Phone: 412-641-1403

Fax: 412-641-1133

Email: chenba@upmc.edu

**Site Investigator**

**Beatrice A. Chen, MD, MPH**

**Protocol Co-Chair/Site Investigator**

Magee-Womens Hospital of UPMC

300 Halket Street

Pittsburgh, PA 15213 USA

Phone: 412-641-1403

Fax: 412-641-1133

Email: chenba@upmc.edu

**US National Institutes of Health (NIH)**

**Roberta Black, PhD**

**Chief, Microbicide Research Branch**

National Institute of Allergy and Infectious Diseases (NIAID), Division of AIDS (DAIDS)
5601 Fishers Lane, Room 8B62, MSC 9831
Rockville, MD 20852 USA

Phone: 301-496-8199

Fax: 301-402-3684

Email: rblack@niaid.nih.gov

**Diana Blithe, PhD**

**Chief, Contraceptive Development Program (CDP)**

**Contraceptive Clinical Trials Network (CCTN)**

Eunice Kennedy Shriver National Institute of Child Health and Human Development (NICHD)

National Institutes of Health (NIH)

6710B ROCKLEDGE DRIVE Room 3303, MSC 7002
Bethesda, MD 20817

Phone: 301-435-6990

Email: blithed@mail.nih.gov

**Jill Long, MD, MPH, MHS**

**Medical Monitor, Contraceptive Development Program**

**Contraceptive Clinical Trials Network (CCTN)**

Eunice Kennedy Shriver National Institute of Child Health and Human Development (NICHD)

National Institutes of Health (NIH)

6710B Rockledge Drive, Room 3243, MSC 7002
Bethesda, MD 20817

Phone: 301-496-1662

Email: jill.long@nih.gov

**Jeanna Piper, MD**

**DAIDS Senior Medical Officer**

NIAID, DAIDS

5601 Fishers Lane, Room 8B68, MSC 9831

Rockville, MD 20852 USA

Phone: 240-292-4798

Fax: 301-402-3684

Email: piperj@niaid.nih.gov

**Dianne M. Rausch, PhD**

**Director**

DAIDS Research, NIMH
5601 Fishers Lane, Room 8D20, MSC 9831

Rockville, MD 20852 USA

Phone: 240-627-3874

Fax: 240-627-3467

Email: drausch@mail.nih.gov

**MTN Leadership and Operations Center (LOC) - Pitt**

| **Jared Baeten, MD, PhD**  **MTN Co-Principal Investigator**  University of Washington  ICRC, Dept of Global Health  325 Ninth Avenue, Box 359927  Seattle, WA 98104  Phone: 206-520-3808  Fax: 206-520-3831  Email: jbaeten@uw.edu | **Sharon A. Riddler, MD, MPH**  **Protocol Physician**  UPMC, Keystone Building, Suite 510  3520 Fifth Avenue Pittsburgh, PA 15213 USA Phone: 412-383-1741 or 412-383-1675 Fax: 412-383-2900 Email: riddler@pitt.edu |
| --- | --- |
| **Katherine Bunge, MD**  **Protocol Safety Physician**  Magee-Womens Hospital of UPMC  300 Halket Street  Pittsburgh, PA 15213 USA  Phone: 412-641-3464  Fax: 412-641-1133  Email: kbunge@mail.magee.edu | **Devika Singh, MD, MPH**  **Protocol Safety Physician**  University of Vermont Health Network  CVMC  130 Fisher Rd  Berlin, VT 05602 USA  Phone: 206-920-0975  Email: devika@mtnstopshiv.org |
| **Sharon Hillier, PhD**  **MTN Principal Investigator**  Microbicide Trials Network  204 Craft Avenue  Pittsburgh, PA 15213 USA  Phone: 412-641-6435  Fax: 412-641-6170  Email: shillier@mail.magee.edu | **Mei Song, PhD**  **Protocol Specialist**  Microbicide Trials Network  204 Craft Avenue  Pittsburgh, PA 15213 USA  Phone: 412-641-2282  Fax: 412-641-6170  Email: songm4@mail.magee.edu |
| **Cindy Jacobson, PharmD**  **Director of Pharmacy Affairs**  Microbicide Trials Network  204 Craft Avenue  Pittsburgh, PA 15213 USA  Phone: 412-641-8913  Fax: 412-641-6170  Email: cjacobson@mail.magee.edu | **Jennifer Thomas, MSc**  **Protocol Development Manager**  Microbicide Trials Network  204 Craft Avenue  Pittsburgh, PA 15213 USA  Phone: 412-641-5579  Fax: 412-641-6170  Email: thomasj15@mwri.magee.edu |

**International Partnership for Microbicides (IPM)**

| **Annal**é**ne Nel, PhD, MD**  **Executive Vice President, Chief Medical Officer**  International Partnership for Microbicides  63 Main Road, Paarl 7646, South Africa  Phone: +27.21.8602300 ext 320  Email: anel@ipmglobal.org.za  **Bríd Devlin, PhD**  **Executive Vice President, Product Development**  International Partnership for Microbicides  8405 Colesville Rd., Suite 600, Silver Spring, MD 20910, USA  Phone: +301.608.2221 ext.249  Email: bdevlin@ipmglobal.org |
| --- |

**MTN Laboratory Center (LC)**

| **May Beamer, BS**  **Laboratory Manager/Supervisor**  Microbicide Trials Network  204 Craft Avenue  Pittsburgh, PA 15213 USA  **Phone:**412-641-6026  **Fax:** 412-641-6170  **Email:** mbeamer@mwri.magee.edu |
| --- |
| **Craig Hendrix, MD**  **MTN LC Pharmacology Core Director**  Johns Hopkins University  600 North Wolfe Street, Harvey 502  Baltimore, MD 21287 USA  Phone: 410-955-9707  Fax: 410-955-9708  Email: cwhendrix@jhmi.edu |
| **Mark Marzinke, PhD, DABCC**  Johns Hopkins at Bayview  Clinical Pharmacology Analytical Laboratory (CPAL) Director/Marzinke Lab  4940 Eastern Avenue  Mason F. Lord (MFL) Center Tower Suite 6000, Room 621  Baltimore, Maryland 21224  Office: 443-287-7516  CPAL Office: 410-550-9703  Fax: 410-955-0767  Email: mmarzin1@jhmi.edu |
| **John Mellors, MD**  **MTN LC Co-Principal Investigator**  University of Pittsburgh 3550 Terrace Street, Suite 818 Pittsburgh, PA 15261 USA  **Phone:**412-624-8512  **Fax:** 412-383-7982  **Email:** jwm1@pitt.edu |
| **Samuel Poloyac, PharmD, PhD Small Molecular Biomarker Core Director**  University of Pittsburgh, School of Pharmacy  807 Salk Hall  3501 Terrace Street  Pittsburgh, PA 15261 USA Phone: 412-624-4595  Email: poloyac@pitt.edu |
| **Lisa C. Rohan, PhD**  **MTN LC Co-Principal Investigator**  University of Pittsburgh  Magee-Womens Research Institute & Foundation  204 Craft Avenue, Room B509  Pittsburgh, PA 15213 USA  **Phone:**412-641-6108  **Fax:** 412-641-6170  **Email:** lrohan@mwri.magee.edu |

**Health Decisions (HD)**

| **Clint Dart, MS**  **CCTN Statistical & Clinical Coordinating Center Principal Investigator/Head of Data Services**  Health Decisions  2510 Meridian Parkway  Durham, NC 27713-2260 USA  Phone: 919-967-1111  Fax: 919-967-1145  Email: cdart@healthdec.com |  |
| --- | --- |
| **Sarah Godfrey, BS**  **CCTN Program Oversight/Senior Clinical Project Manager**  Health Decisions  2510 Meridian Parkway  Durham, NC 27713-2260 USA  Phone: 919-967-1111  Fax: 919-967-1145  Email: sgodfrey@healthdec.com |  |

**MTN Statistical Data Management Center (SDMC)**

| **Tanya Harrell, BS**  **Clinical Data Manager**  FHCRC - SCHARP  1100 Fairview Ave. North, E3- 129  PO Box 19024  Seattle, WA 98109-1024 USA  Phone: 206-667-6568  Fax: 206-667 -4812  Email: tharrell@scharp.org |  |
| --- | --- |
| **Cliff Kelly, MS**  **SDMC Statistician**  FHCRC-SCHARP  1100 Fairview Avenue North, M2-C200  PO Box 19024  Seattle, WA 98109-1024 USA  Phone: 206-667-2502  Fax: 206-667-4378  E-mail: cwkelly@scharp.org |  |
| **Karen Patterson, MPH**  **SDMC Program and Portfolio Manager**  FHCRC - SCHARP  1100 Fairview Avenue North, E3-129  PO Box 19024  Seattle, WA 98109-1024 USA  Phone: 206-667-7052  Fax: 206-667-4812  Email: karenp@scharp.org |  |
| **Barbra Richardson, PhD**  **Faculty Statistician**  FHCRC-SCHARP  1100 Fairview Ave. North, M2-C200  PO Box 19024  Seattle, WA 98109-1024 USA  Phone: 206-667-7788  Fax: 206-667-4812  Email: barbrar@uw.edu |  |

**MTN Working Group Representatives**

**Jeanne Marrazzo, MD, MPH, FACP, FIDSA**

**Biomedical Science Working Group (BSWG) Representative**

University of Alabama at Birmingham School of Medicine
Tinsley Harrison Tower 215
1900 University Blvd.
Birmingham, AL 35294-0006
Phone: 205-975-5500 or 205-934-5191
Fax: 205-996-2416

Email: jmm2@uab.edu

**Ariane van der Straten, PhD, MPH**

**Behavioral Research Working Group (BRWG) Representative**

RTI International

351 California Street, Suite 500

San Francisco, CA 94104 USA

Phone: 415-848-1324

Fax: 415-848-1330

Email: ariane@rti.org

**MTN-044/IPM 053/CCN019**

**A Randomized, Phase 1, Open-Label Study in Healthy HIV-Negative Women to Evaluate the Pharmacokinetics, Safety and Bleeding Patterns Associated with 90-Day Use of Matrix Vaginal Rings Containing 200 mg Dapivirine and 320 mg Levonorgestrel**

**INVESTIGATOR SIGNATURE FORM**

**Version 2.0; June 7, 2018**

**Funded by:**

US *Eunice Kennedy Shriver* National Institute of Child Health and Human Development

Division of AIDS (DAIDS), US National Institute of Allergy and Infectious Diseases

US National Institute of Mental Health

US National Institutes of Health (NIH)

**PROTOCOL AGREEMENT**

I, the Investigator of Record, agree to conduct this study in full accordance with the provisions of this protocol. I agree to conduct this study in compliance with United States (US) Health and Human Service regulations (45 CFR 46); applicable U.S. Food and Drug Administration regulations; standards of the International Conference for Harmonization Guideline for Good Clinical Practice (E6); The Declaration of Helsinki; Institutional Review Board/Ethics Committee determinations; and all applicable in-country, state, and local laws and regulations.

I agree to maintain all study documentation for at least two years after the last approval of a marketing application in an ICH region and until there are no pending or contemplated marketing applications in an ICH region or at least two years have elapsed since the formal discontinuation of clinical development of the investigational product. These documents should be retained for a longer period, however, if required by the applicable regulatory requirements or by an agreement with the sponsor. NICHD will inform the investigator/institution as to when these documents no longer need to be retained.

I have read and understand the information in the Investigator's Brochure(s), including the potential risks and side effects of the products under investigation, and will ensure that all associates, colleagues, and employees assisting in the conduct of the study are informed about the obligations incurred by their contribution to the study.

The signature of the investigator below indicates acceptance of the protocol and a complete understanding of the investigator obligations as outlined in Investigator Obligations.

______________________________

Name of Investigator of Record (print)

____________________________ ______________________________

Signature of Investigator of Record Date

Site Number __________________

Site Name ______________________________________

Address _______________________________________

_______________________________________

_______________________________________

Phone Number __________________________

**MTN-044/IPM 053/CCN019**

**A Randomized, Phase 1, Open-Label Study in Healthy HIV-Negative Women to Evaluate the Pharmacokinetics, Safety and Bleeding Patterns Associated with 90-Day Use of Matrix Vaginal Rings Containing 200 mg Dapivirine and 320 mg Levonorgestrel**

# PROTOCOL SUMMARY

**Short Title:** PK Study of 90-Day Use of Vaginal Rings Containing Dapivirine

and Levonorgestrel

**Clinical Phase:** Phase 1

**IND Sponsor:** International Partnership for Microbicides (IPM)

**Protocol Chair:** Sharon L. Achilles, MD, PhD, FACOG

**Protocol Co-Chair:** Beatrice A. Chen, MD, MPH

**Sample Size:** Approximately 24 participants

**Study Population:** Healthy, HIV-uninfected females, 18-45 (inclusive) years old

**Study Sites:** One US site

**Study Design:** Phase 1, two-arm, open-label, single-site, randomized trial (1:1)

**Study Duration:** Accrual will require approximately 6 months. Participants will be

followed for approximately 26 weeks.

**Study Products:** One silicone elastomer vaginal ring (VR) containing the active

ingredients dapivirine (DPV) and levonorgestrel (LNG), formulated

as IPM VR: 200 mg DPV + 320 mg LNG (Ring-102)

**Study Regimen:** Participants will insert one VR and will be randomized to the study product in a 1:1 ratio to one of the following use regimens:

1. Regimen A: VR used continuously for approximately 90 days
2. Regimen B: VR used cyclically for approximately 90 days as

follows: used for ~28 days, then removed, washed

and stored for 2 days. The same ring will be used

for 2 additional cycles in a similar fashion.

Figure 1: Study Visit Schedule


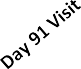

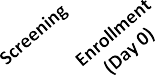

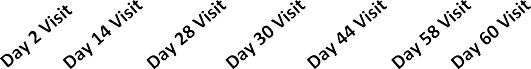

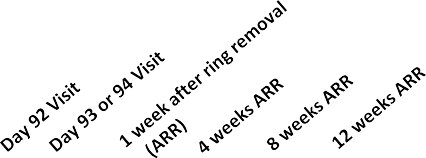

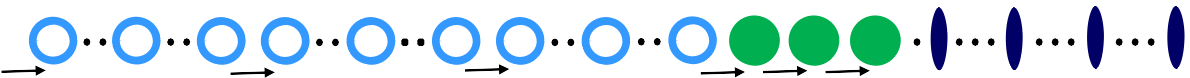

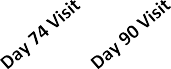


***≤*60**

**days**

2 days

2 days

2 days

1 day 1 day 1 or 2 days

**Visit 1**

**V2 V3**

**V4**

**V5 V6**

**V7**

**V8 V9**

**V10 V11 V12 V13 V14 V15 V16 V17 V18**

**(up to 4 phone calls/contacts)**

**Primary Objective:**

**Pharmacokinetics**

- To characterize the local and systemic pharmacokinetics (PK) of one DPV-LNG vaginal ring formulation used either continuously or cyclically (~28/2) for approximately 90 days

**Primary Endpoint:**

**Pharmacokinetics**

- DPV and LNG concentrations in plasma
- DPV and LNG concentrations in cervicovaginal fluid
- DPV concentration in cervical tissue

**Secondary Objectives:**

**Safety**

- To evaluate the safety of one DPV-LNG vaginal ring formulation used either continuously or cyclically (~28/2) for approximately 90 days

**Secondary Endpoints:**

**Safety**

- Grade 2 or higher genitourinary adverse events as defined by the Division of AIDS (DAIDS) Table for Grading the Severity of Adult and Pediatric Adverse Events, Corrected Version 2.1, July 2017, and/or Addendum 1 (Female Genital Grading Table for Use in Microbicide Studies [Dated November 2007])
- Grade 3 or higher adverse events as defined by the Division of AIDS (DAIDS) Table for Grading the Severity of Adult and Pediatric Adverse Events, Corrected Version 2.1, July 2017

**Exploratory Objectives:**

**Bleeding**

- To assess vaginal bleeding patterns associated with one DPV-LNG vaginal ring formulation used either continuously or cyclically (~28/2) for approximately 90 days

**Acceptability**

- To explore women’s experience with continuous or cyclic use of one DPV-LNG vaginal ring, including bleeding, tolerability and other measures of ring acceptability

**Adherence**

- To evaluate participant adherence to one DPV-LNG vaginal ring formulation used either continuously or cyclically (~28/2) for approximately 90 days

**Vaginal Microenvironment**

- To describe the genital microenvironment in HIV-uninfected women during approximately 90 days of continuous or cyclic use of one DPV-LNG vaginal ring

**Pharmacodynamics**

- To evaluate the HIV inhibitory activity in cervical tissue over approximately 90 days during continuous or cyclic use of one DPV-LNG vaginal ring

**Exploratory Endpoints:**

**Bleeding**

- The total number of days of vaginal bleeding
- The total number of bleeding episodes

**Acceptability**

- Self-reported attitudes about ring attributes, use regimens and tolerability

**Adherence**

- Frequency and duration of study vaginal ring removal (voluntary and involuntary)
- Drug pharmacokinetic levels
- Residual drug levels (DPV and LNG) in returned vaginal rings

**Vaginal Microenvironment**

- Changes in microenvironment (microbiota and biomarkers)

**Pharmacodynamics**

- Anti-HIV-1 activity in cervical tissue

# KEY ROLES

## Protocol Identification

Protocol Title: A Randomized, Phase 1, Open-Label Study in Healthy HIV-Negative Women to Evaluate the Pharmacokinetics, Safety and Bleeding Patterns Associated with 90-Day Use of Matrix Vaginal Rings Containing 200 mg Dapivirine and 320 mg Levonorgestrel

Protocol Number: MTN-044/IPM 053/CCN019

Short Title: PK Study of 90-Day Use of Vaginal Rings Containing

Dapivirine and Levonorgestrel

Date: June 7, 2018

## Funding Agencies, Sponsor and Monitor Identification

Funding Agencies: US *Eunice Kennedy Shriver* National Institute of Child Health and Human Development (NICHD)

Contraceptive Development Program

6710B Rockledge Drive

Bethesda, MD 20817 USA

US Division of AIDS (DAIDS)/US National Institute of Allergy and Infectious Diseases (NIAID)

National Institutes of Health (NIH)

6700 B Rockledge Drive

Rockville, MD 20852 USA

US National Institute of Mental Health (NIMH)

6001 Executive Boulevard
Rockville, MD 20852 USA

IND Sponsor: International Partnership for Microbicides (IPM)

8405 Colesville Rd., Suite 600
Silver Spring, MD 20910 USA

Monitor:  Health Decisions Inc. (HD)

2510 Meridian Parkway

Durham, NC 27713-2260 USA

Medical Officers: Jill Long, MD, MPH, MHS

6710B Rockledge Drive, Room 3243, MSC 7002

Bethesda, MD 20817

Jeanna Piper, MD
5601 Fishers Lane, Room 8B68, MSC 9831
Rockville, MD 20852 USA

## Laboratory Centers

MTN Laboratory Center (LC)

204 Craft Avenue

Pittsburgh, PA 15213 USA

MTN LC Pharmacology Core

600 N. Wolfe Street, Osler 527

Johns Hopkins University

Baltimore, MD 21287 USA

MTN LC Small Molecular Biomarker Core

807 Salk Hall

3501 Terrace Street

Pittsburgh, PA 15261 USA

## Data Center

Data Center: MTN Statistical Data and Management Center (SDMC)

Statistical Center for HIV/AIDS Research & Prevention

(SCHARP)/Fred Hutchinson Cancer Research Center

(FHCRC)

1100 Fairview Avenue N., LE-400

PO Box 19024

Seattle, WA 98109-1024 USA

Qualitative Data Center: Research Triangle Institute (RTI) International
351 California Street, Suite 500
San Francisco, CA 94104 USA

## Study Implementation

Study Implementation: Health Decisions Inc.

2510 Meridian Parkway

Durham, NC 27713-2260 USA

# INTRODUCTION

## Microbicides, HIV Prevention and Contraception

In 2016, 36.7 million people globally were living with HIV. In the same year, 1.8 million people were newly infected and 1 million lost their lives to human immunodeficiency virus (HIV)-related illnesses.^1^ The burden of the HIV/AIDS epidemic is particularly high in sub-Saharan Africa where 70% (approximately 25.5 million) of all HIV-infected people live and where 1.4 million new infections occurred in 2015.^2^ Heterosexual vaginal intercourse remains the major mode of HIV transmission in sub-Saharan Africa, with women bearing the greatest impact of the epidemic. Every 60 seconds, a young woman is infected with HIV^3^ and women account for approximately 51% of people living with HIV worldwide.^1^ In the US, the proportion of AIDS diagnoses in women has more than tripled since the early days of the epidemic, from 7% in 1985 to 24% in 2015. Among the women living with diagnosed HIV in 2015, 61% were African American, 15% were Hispanic/Latina and 19% were white.^4^ African Americans and Hispanics only represent 31% of people in the US, but accounted for 77% of women living with HIV.^5^ Worldwide, HIV/AIDS remains the leading cause of death among women of reproductive age (15 to 44 years of age), and among adolescent girls in Africa.^6^ The ongoing development of safe and effective HIV prevention technologies easily accessible to under-resourced communities and countries remains a public health priority.

Unprotected heterosexual intercourse is the leading mode of HIV acquisition among women. Correct and consistent use of latex condoms is one proven method of preventing HIV acquisition. However, since many women may be unable to negotiate condom use with their partners, condom use is regarded as an inadequate prevention option for women. Thus, developing HIV prevention options that women can use independent of male partner consent remains a global concern.

In addition to HIV acquisition, unintended pregnancies also exert a health and economic burden in the developed and developing world. Globally, nearly half of pregnancies (100+ million per year) are unintended.^7^ On average, women in developing countries have many more pregnancies than women in developed countries, and their lifetime risk of death due to pregnancy is higher.^8^ Developing regions accounted for approximately 99% of the global maternal deaths in 2015, with sub-Saharan Africa alone accounting for roughly 66% and almost one third occurring in South Asia.^9^ Many highly effective contraceptives have been available for decades. Low utilization rates and high discontinuation rates remain problems due to factors including inconvenience, cost, inaccessibility, and a constrained ability for women in many developing nations to fully participate in sexual and reproductive decision-making. For women, similar factors fuel both unintended pregnancies and the acquisition of HIV: lack of education, malnutrition, poverty and oppression of women.^10^ As a result, the need for highly acceptable, effective, affordable, dual-purpose contraceptive and HIV prevention options seems more urgent than ever.

Multipurpose prevention technologies (MPTs), often referred to as “combination” or “dual” technologies, are innovative products currently under development that are designed for at least two sexual and reproductive health (SRH) prevention indications. These products are intended to simultaneously prevent unintended pregnancy and sexually transmitted infections (STIs), including HIV, and/or reproductive tract infections (RTIs).^11^ MPTs include vaccines, contraceptives, microbicides and devices such as intravaginal rings and diaphragms. Barrier devices like male and female condoms and diaphragms are already available, and research is underway to develop new and more innovative biomedical interventions that may allow women and young girls to address multiple SRH issues with one product. The majority of new MPT candidates in development focus on the development or improvement of physical barriers, chemical barriers, and physical/chemical barrier combinations.^11^ One product advanced in clinical testing is a 1% tenofovir (TFV) gel that has been shown to fight against HIV and herpes simplex virus type 2 (HSV-2) infections from one trial and a confirmatory Phase 3 trial is currently underway in South Africa.^12^ MPTs potentially offer a cost-effective approach to addressing an important public health need, which could result in social and economic benefits to women and their families worldwide.

For a MPT to be effective, it is essential that it is used correctly and consistently, and is also acceptable to the user. In addition, a product used independently of sex could be more convenient for women and provide long-term protection during anticipated and unanticipated sexual intercourse. Higher adherence to a product may translate into higher effectiveness of the product to help prevent HIV acquisition. It is likely that products that can be applied less frequently or products that can remain in situ for an extended duration will be more acceptable and will achieve better adherence. Vaginal rings (VRs) that are replaced monthly or less frequently may have benefits over dosage forms that need to be used more frequently.

Multiple clinical trials have evaluated the safety of dapivirine (DPV) in VRs,^13^ aqueous gels,^14-17^ quick-dissolve vaginal films,^14-16^ and in an oral formulation.^18,19^ These clinical trials support the favorable safety profile and tolerability of DPV in general and specifically in vaginal delivery formulations. The safety and efficacy of the VR containing DPV 25 mg (Ring-004) replaced monthly were recently tested in two randomized, double-blind, placebo-controlled Phase 3 trials: MTN-020 (ASPIRE) and IPM 027 (The Ring Study). Both trials demonstrated that the VR was well tolerated and reduced the risk of HIV-1 infection by approximately 27% and 30%, respectively.^20-22^

The development of a VR with a higher DPV loading dose may allow less frequent VR replacements (e.g., quarterly basis instead of monthly basis), which may further reduce user and provider burden, streamline follow-up, and improve adherence. Ring formulations containing DPV and levonorgestrel (LNG), designed for multipurpose prevention, are also being developed, including the Dapivirine-Levonorgestrel VR (Ring-102), containing 200 mg of DPV and 320 mg LNG. It has been designed to provide sustained release of DPV and LNG for a minimum of 3 months, and has recently been evaluated in MTN-030/IPM 041, a first-in-human Phase I clinical trial, to determine the safety and pharmacokinetic (PK) profile when used continuously for 14 days by healthy, HIV-uninfected women.

MTN-044/IPM 053/CCN019 is a collaborative study between the Microbicide Trials Network (MTN), the International Partnership for Microbicides (IPM), and the Contraceptive Clinical Trials Network (CCTN) to evaluate the pharmacokinetics (PK) and safety of this DPV-LNG VR when used for 3 months. This will be the first study to assess local and systemic PK, safety and tolerability of the VR when used by women continuously for approximately 90 days compared with cyclic use (worn for approximately 28 days and taken out for 2 days) for approximately 90 days.

## Dapivirine

### Description

DPV (also known as TMC-120), a non-nucleoside reverse-transcriptase inhibitor (NNRTI), is a substituted di-amino-pyrimidine derivative with potent antiviral activity against HIV-1. DPV is chemically described as 4-[[4-[(2,4,6-trimethylphenyl)amino]-2-pyrimidinyl]amino]benzonitrile.^22^

DPV was originally developed by Janssen Sciences Ireland UC (formerly Tibotec Pharmaceuticals Ltd.), a subsidiary of Johnson & Johnson, as an oral ARV compound for treatment of HIV/AIDS and was tested in Phase 1 and 2 clinical trials in more than 200 participants.^23^ DPV is a promising topical microbicide candidate due to its proven *in vitro* and *in vivo* efficacy, favorable safety profile, and its physical and chemical properties. DPV has potent activity against wild-type HIV-1 strains and strains harboring different resistance-inducing mutations.^13,17,22,24^ The ARV profile of DPV is superior to that of several other NNRTI drugs, including nevirapine (NVP), delavirdine (DLV), and efavirenz (EFV). Like other NNRTIs, *in vitro* tests have also shown that DPV is not active against HIV-2 and has little or no activity against common STIs; therefore, it is not intended for use against HIV-2 or other STIs. DPV does not have any contraceptive properties.^25^ The DPV VR is intended to be used as complementary prevention technology to other HIV prevention strategies.^13^ Detailed information on DPV is available in the DPV VR Investigator’s Brochure (IB).^13^

Systemic exposure to DPV observed in women using the DPV ring (Ring-004) was very low (C_max_ = 462 pg/mL in IPM 028) and the area under the curve (AUC)_0-24h_ = 3.121 ng.h/mL in IPM 028 and these findings suggest that the DPV dose will be unlikely to alter drug metabolism of the co-administered drugs.^13^ MTN-044/IPM 053/CCN019 will obtain PK data for both DPV and LNG to describe *in vivo* exposure to this combination of drugs and the potential for other drug-drug interactions to inform appropriate dosing.

IPM has investigated a wide range of dosage forms for the development of topical microbicide products, including vaginal gels, VRs, films, tablets and soft gel capsules. Vaginal gel was the initial dosage form chosen for a DPV-based microbicide because the majority of previous microbicides evaluated in clinical trials were vaginal gels. Therefore, a wealth of information was available on this dosage form. However, the silicone elastomer matrix VR has been prioritized over all other dosage forms for the following reasons:

- Clinical trials have demonstrated sustained delivery of high levels of DPV throughout the cervicovaginal vault for up to 1 month;
- Since the VR can deliver drug for at least 1 month, the burden of user-dependent adherence is lower than for once-daily products;
- Product acceptability studies and the experience gained from marketed VR products have established a high level of acceptance and adherence in women using VRs with similar physical characteristics;
- The overall cost for the VR is relatively low;
- The VR requires minimal storage space when compared to once daily products;
- VR use may be extended beyond 28 days with this inert study delivery device.

Summaries of the safety and tolerability of DPV administered orally and vaginally as evaluated in clinical studies by IPM and Janssen Sciences Ireland UC can be found in [Section 2.4.3](#_2.5.3_Safety_of).

### Mechanism of Action

DPV is an NNRTI. NNRTIs bind to the HIV reverse transcriptase (RT) enzyme thereby preventing viral replication and therefore the production of an infectious virus.

## Nonclinical Studies of Dapivirine

### Nonclinical Virology and Pharmacology

**Anti-HIV-1 Activity**

The antiviral activity of DPV against wild-type HIV-1, clinical isolates of HIV-1 (including subtype C virus), and a panel of NNRTI-resistant viruses has been established using *in vitro* models. The median effective concentration (EC_50_) values ranged from 0.3 ng/mL (0.9 nM) against laboratory isolates to <33 ng/mL (<100 nM) against 80% of HIV-1 isolates encoding one or more known NNRTI resistance mutations.^13^ The anti-HIV activity of DPV as a microbicide was confirmed in an *ex vivo* model of human cervical and colonic explant cultures.^22^ DPV potently inhibited HIV-1_BaL_ (R5) infection of human ectocervical tissue explants in a dose-dependent manner (IC_50_ = 1.5 nM [0.49 ng/mL]). Although complete inhibition of integrated provirus was not observed at all concentrations, greater than 99% inhibition was observed at concentrations down to 10 nM (3.3 ng/mL).^13,26^ In a humanized severe combined immunodeficient (hu-SCID) mouse model, DPV blocked vaginal transmission of HIV-1 following challenge with HIV‑1-infected human peripheral blood lymphocytes after a single vaginal application of gel containing DPV at concentrations of 2.25 µM (0.7 µg/mL) and higher. The efficacy rate ranged from approximately 70 to 100%, depending on the vaginal gel formulation.^13^

**Resistance**

In vitro studies showed that development of reduced susceptibility to DPV typically required more than one substitution in reverse transcriptase. The observed substitutions included V90I/V, A98S, L100I, K101E, K103N, V106I/V, V108I, E138A/E/G/K/Q/R, T165I, V179E/F/I/M/V, Y181C, Y188H, G190A/E, L214F, F227Y, M230I.^13,27^

**Cross-resistance**

In comparison with NVP, DLV, EFV and emivirine, DPV showed significantly better *in vitro* activity against HIV strains resistant to one or more NNRTIs.^13^ In further experiments, DPV and MIV-160 were able to fully inhibit at sub-micromolar concentrations most NNRTI-resistant viruses selected in the presence of increasing concentrations of DPV, UC781, MIV-160, EFV, and NVP, whereas UC781 was not. In comparison, DLV and NVP lost activity against all resistant viruses, while EFV retained some level of potency against some resistant viruses.^13^

**Secondary and Safety Pharmacology**

In a series of nonclinical safety pharmacology studies, DPV was generally devoid of adverse effects on overt behavior, reflexes, and other body functions. Although these studies revealed no cardiovascular effects, there was evidence of an increase in QT interval during the 4-week and 6-month oral toxicity studies in dogs. However, this was only seen at ≥ 30 mg/kg/day. DPV demonstrated the potential to bind the progesterone receptor *in vitro*. The relative binding affinity (RBA) compared to progesterone was approximately 0.1% to 0.2% and 3230-fold lower than that of LNG (RBA = 323%), therefore DPV is unlikely to impede the progestogenic activity of LNG.^28^

### Nonclinical Pharmacokinetics

Systemic exposure to DPV was relatively low following vaginal administration of DPV gels in rabbits. Much higher systemic exposures were obtained in single dose oral and subcutaneous toxicity studies in mice and rats, and in repeat dose oral toxicity studies in rats, dogs, and monkeys. In vitro, DPV was highly bound to proteins in plasma (> 99%), whereas in vaginal fluid < 16% was protein bound. In rats, tissue to plasma AUC_0-24h_ ratios following a single oral dose were eleven in liver, seven to eight in lung, kidney and adrenals, approximately four in spleen and lymph nodes, and two to three in brain, heart, and muscle. Plasma/tissue equilibrium was rapid, and there was no undue retention of DPV in tissues. Following a single oral or vaginal dose of ^14^C-DPV, absorption and distribution of drug-related material to the tissues was moderate in non-pregnant and slow in pregnant female rats. Vaginal dosing did not result in greater distribution to the reproductive tissues (except the vaginal wall) than oral dosing. For virtually all tissues, maximal concentrations after vaginal dosing were < 1% of those after oral dosing. Drug-related material was shown to freely cross the placenta to the fetus. In dogs, DPV concentrations following oral administration for 14 days were about nine times higher in liver and muscle, and about five times higher in lymph nodes and brain than in plasma. Preliminary metabolism studies demonstrated the presence of free and conjugated metabolites in rats, dogs, monkeys, and humans, but the molecular structures have not been elucidated. In vitro it was shown that DPV is metabolized by cytochrome P450 (CYP450; primarily CYP3A and, to a lesser extent, by the CYP2C family), followed by glucuronidation by UGT1A and -2B isoenzymes.^28^

### Nonclinical Toxicology

DPV has been investigated in single and repeat dose toxicity studies, in a range of reproductive toxicity and mutagenicity studies, and in a carcinogenicity study. In vaginal studies in rabbits, there were no significant local or systemic findings following repeat administration at up to 20 mg/mL for 14 days, up to 5 mg/mL for 13 weeks, or up to 2 mg/mL for 39 weeks, or in reproductive toxicity studies in rats and rabbits performed at concentrations up to 2 mg/mL. In studies of oral administration in rats, a no observed effect level (NOEL) could not be established, however, the observed effects on liver, thyroid, and pituitary were considered adaptive rather than adverse responses, and therefore the no observed adverse effect level (NOAEL) was considered to be 20 mg/kg/day. This dosage was also the NOAEL in dog studies. At higher dose levels, hepatotoxicity and adrenal cortical fatty changes were observed in dogs, and slight hematological and clinical chemistry changes were observed in rats. The NOAEL in oral reproductive toxicity studies in the rat was also 20 mg/kg/day, whereas in the rabbit no effects were seen at dosages up to 90 mg/kg/day. DPV was considered to be non-genotoxic. In the guinea pig, DPV (2 mg/mL) did not demonstrate any potential to cause contact sensitization. No treatment-related neoplastic or non-neoplastic findings were seen in a vaginal carcinogenicity study in rats at concentrations up to 5 mg/mL.^28^

## Clinical Studies of Dapivirine

### Clinical Studies of Dapivirine Vaginal Rings

To date, 37 Phase 1 to Phase 3 clinical trials of DPV with various dosage forms have been completed. These include 18 trials of DPV VRs in 5399 participants (DPV rings, n=3189; placebo rings, n=2210); 8 trials of DPV vaginal gel in 774 participants (DPV gel, n=491; placebo gel, n=283); and 11 trials of oral DPV (oral DPV, n=211 participants).^13^

Among them, two pivotal Phase 3 trials (IPM 027 and MTN-020) evaluated the long-term safety and efficacy of the 25 mg DPV VR (Ring-004) for the prevention of HIV-1 acquisition in 4588 healthy, sexually active, HIV-uninfected women 18 to 45 years of age. The VR used in the two studies was replaced monthly.^20,29^

In the 18 DPV VR trials completed to date (summarized in Table 1 below), two were conducted with reservoir configuration VRs (Rings-001 and Ring-002), and one included both a reservoir VR (Ring-002) and a matrix ring with tin-catalyzed silicone curing (Ring-003).^13^ Fourteen trials, including the two efficacy studies, were conducted using a matrix ring with platinum–catalyzed silicone curing (Ring-004).^30^ One trial (MTN-030/IPM 041) used Ring-102, a combination VR containing 200 mg DPV and 320 mg LNG, which will be the ring used in this trial. PK data from the trials with Ring-004, along with safety data from the oral studies, are summarized below.

Table 1: Phase 1-3 Clinical Trials of Dapivirine Vaginal Rings

| **Trial Number** | **Description** | **Country** | **Dapivirine Ring** | **Number of Participants** | |
| --- | --- | --- | --- | --- | --- |
|  |  |  |  | **Dapivirine** | **Placebo** |
| IPM 001 | Safety and PK in women; 7 days | Belgium | Ring-001 | 12 | 12 crossover |
| IPM 008 | Safety and PK in women; 7 days | Belgium | Ring-002 | 10 | 3 |
| IPM 013 | Safety and PK in women; 56/57 days | Belgium | Ring-004 | 36 | 12 |
| IPM 015 | Safety and PK in women; 84 days | Kenya, Malawi, South Africa, Tanzania | Ring-004 | 140 | 140 |
| IPM 018 | Safety and PK in women; 28 days | Belgium | Ring-002  Ring-003 | 8  8 | 8 |
| IPM 024 | Safety and PK in women; 28 days | Belgium | Ring-004 | 8 | 8 |
| IPM 027 | Safety and efficacy; 2 years | South Africa, Uganda | Ring-004 | 1307 | 652 |
| IPM 028 | Safety and PK in women, Drug-drug interaction; 112 days | Belgium | Ring-004 | 36 | -- |
| IPM 034 | Safety and PK in women; 7 to 84 days | Belgium | Ring-004 | 40 | -- |
| IPM 035 | Effects of menses and tampon on PK in women | Belgium | Ring-004 | 38 | -- |
| IPM 036 | Potential drug-drug interactions and safety in women; 28 days | Belgium | Ring-004 | 36 | -- |
| MTN-013/IPM 026 | Safety and PK in women; 52 days | United States | # | 12 | 12 |
| MTN-020 | Safety and efficacy in women; median 1.6 years | Malawi, South Africa, Uganda, Zimbabwe | Ring-004 | 1313 | 1316 |
| MTN-023/IPM 030 | Safety in adolescent females; 24 weeks | United States | Ring-004 | 73 | 23 |
| MTN-024/IPM 031 | Safety in postmenopausal women; 12 weeks | United States | Ring-004 | 72 | 24 |
| MTN-025 | Safety and adherence in women; 1 year | Malawi, South Africa, Uganda, Zimbabwe | Ring-004 | Former MTN-020 participants | -- |
| MTN-029/IPM 039 | PK in lactating women; 14 days | United States | Ring-004 | 16 | -- |
| MTN-030/IPM 041 | Safety and PK in women; 14 days | United States | Ring-102 | 12  12 | -- |
| **TOTAL participants** | | | | **3189** | **2210** |

Ring-001 - Tin-catalyzed silicone reservoir ring (200 mg DPV)

Ring-002 - Tin-catalyzed silicone reservoir ring (25 mg DPV)

Ring-003 - Tin-catalyzed silicone matrix ring (25 mg DPV)

Ring-004 - Platinum-catalyzed silicone matrix ring (25 mg DPV)

Ring-102 - Silicone elastomer matrix ring (200 mg DPV and 320 mg LNG)

# Investigational platinum-catalyzed silicone matrix ring (25 mg DPV)

### Pharmacokinetics of Dapivirine

**Dapivirine VRs: DPV 25 mg VR (Ring-004)**

The PK profile of the DPV 25 mg VR (Ring-004) was evaluated in several Phase 1/2 trials. DPV concentrations have been assessed in vaginal fluid and plasma. In most Phase I trials, vaginal fluid samples were collected at three locations: near the cervix, near to where the ring was placed and near the introitus; in the Phase III trial IPM 027, vaginal fluid samples were collected at the cervix. The concentration of DPV in cervical tissue biopsies was also assessed in two trials, although there is uncertainty as to where measured drug concentrations were located (e.g., on the tissue surface, in the dead keratinized cell layers, or in interstitial fluid and living target cells).

In general, the shape of the pharmacokinetic profiles for DPV concentrations in vaginal fluids at the different vaginal sampling locations were similar across trials, with DPV concentrations generally highest near the ring, followed by the cervix, with the lowest concentrations near the introitus.^13^ At all three sampling locations, DPV vaginal fluid concentrations that exceeded the in vitro IC_99_ for HIV-1BaL in cervical tissue by more than 1000-fold, were observed within hours after insertion of Ring-004, increasing to over 3000-fold the IC_99_ within the first day after ring insertion. Maximum DPV concentrations were generally observed within 14 days post ring insertion and then decreased steadily throughout the remainder of the ring use period, although concentrations were still > 3000-fold (at the introitus) and > 6000-fold (near the ring and cervix) above the IC_99_ after 28 days of continuous ring use. The PK of DPV when Ring-004 was used over various periods, including prolonged use for up to 84 days, was evaluated in trial IPM 034. Data from this trial are presented in Figure 2, Figure 3 and Table 2. Extending ring use beyond 1 month resulted in a gradual decline in vaginal fluid concentration, but these remained well above the IC_99_ throughout all use periods. Upon removal of the ring, vaginal fluid concentrations declined rapidly with a half-life of approximately 21 hours (based on population pharmacokinetic modeling analyses incorporating the data from multiple trials).^13^

In trial IPM 035, temporary ring removal (5 days removal during menses) resulted in a marked drop in DPV vaginal fluid concentrations. After re-insertion of the same ring, vaginal fluid concentrations started to increase again immediately and returned to levels similar to those in the control group (continued ring use, no menses).^13^

Figure 2: Mean Vaginal Fluid Concentration-Time Curves of Dapivirine (ng/g) after Insertion of the Dapivirine Vaginal Ring-004 for 7, 14, 28, 56, or 84 Days (Groups A, B, C, D, and E): Mean ± SD Values


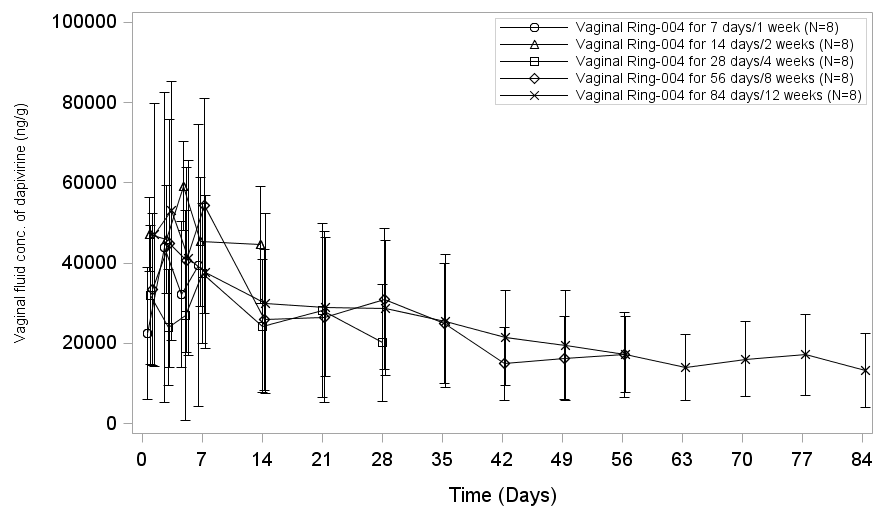


Figure 3: Mean Plasma Concentration-Time Curves of Dapivirine (pg/mL) after Insertion of the Dapivirine Vaginal Ring-004 for 7, 14, 28, 56, or 84 Days (Groups A, B, C, D, and E): Mean ± SD Values


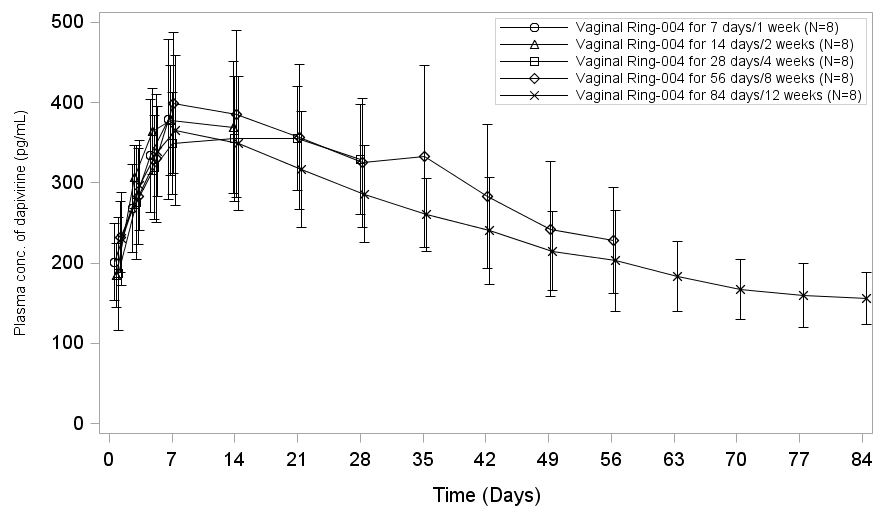


Table 2: Pharmacokinetics of Dapivirine in Plasma and Vaginal Fluids (collected at the cervix) after Insertion of the Dapivirine Vaginal Ring-004 for 7, 14, 28, 56, or 84 Days (Groups A, B, C, D, and E)

| ***Fluid Type***  PK Parameter (mean [± SD], tmax: median [range]) | **Group A (7 days/**  **1 week ring insertion)** | **Group B (14 days/**  **2 weeks ring insertion)** | **Group C (28 days/**  **4 weeks ring insertion)** | **Group D (56 days/**  **8 weeks ring insertion)** | **Group E (84 days/**  **12 weeks ring insertion)** |
| --- | --- | --- | --- | --- | --- |
| ***Plasma*** |  |  |  |  |  |
| n* | 8 | 8 | 8* | 8 | 8 |
| Cmax (pg/mL) | 385 (93.9) | 408 (67.1) | 389 (76.1) | 449 (83.7) | 393 (88.6) |
| tmax (h) | 167.84  (119.45 - 168.13) | 169.29  (119.33 - 336.80) | 419.70  (71.73 - 672.47) | 252.86  (168.82 - 841.17) | 251.54  (71.75 - 503.77) |
| Cprior to ring removal  (pg/mL) | 379 (99.5) | 369 (82.0) | 329 (68.7) | 228 (66.5) | 156 (32.0) |
| AUC0-7days (pg.h/mL) | 45871 (9626) | 48698 (6375) | 43966 (9747) | 48384 (10032) | 48316 (8307) |
| AUC0-14days (pg.h/mL) | - | 111360 (17043) | 103233 (18544) | 114310 (24872) | 108291 (21136) |
| AUC0-28days (pg.h/mL) | - | - | 219747 (38717) | 234045 (53357) | 214841 (40726) |
| AUC0-35days (pg.h/mL) | - | - | - | 289359 (61233) | 260716 (48646) |
| AUC0-42days (pg.h/mL) | - | - | - | 341066 (71353) | 302719 (57481) |
| AUC0-56days (pg.h/mL) | - | - | - | 424686 (96618) | 376425 (75342) |
| AUC0-84days (pg.h/mL) | - | - | - | - | 491981 (95700) |
| ***Vaginal Fluid (Cervix)*** |  |  |  |  |  |
| n* | 8 | 8 | 8* | 8 | 8 |
| Cmax (µg/g) | 53.1 (35.1) | 61.3 (10.4) | 48.2 (13.7) | 58.8 (22.7) | 59.2 (30.6) |
| tmax (h) | 95.94  (24.02 - 168.17) | 119.86  (4.10 - 337.07) | 120.12  (4.00 - 503.82) | 168.87  (4.07 - 169.00) | 71.76  (3.95 - 672.13) |
| Cprior to ring removal  (µg/g) | 39.5 (35.1) | 44.6 (14.6) | 20.1 (14.7) | 17.2 (10.7) | 13.3 (9.23) |
| AUC0-7days (µg.h/g) | 5569 (3472) | 8168 (1610) | 4663 (2192) | 6943 (3685) | 7448 (4277) |
| AUC0-14days (µg.h/g) | - | 15709 (3945) | 9838 (4302) | 13671 (7039) | 13137 (7364) |
| AUC0-28days (µg.h/g) | - | - | 18376 (9662) | 22921 (12155) | 22957 (12377) |
| AUC0-35days (µg.h/g) | - | - | - | 27626 (14553) | 27511 (14767) |
| AUC0-42days (µg.h/g) | - | - | - | 30972 (16270) | 31455 (16913) |
| AUC0-56days (µg.h/g) | - | - | - | 36414 (18999) | 38011 (20642) |
| AUC0-84days (µg.h/g) | - | - | - | - | 48489 (25527) |
| * n = 7 for Cprior to ring removal and AUC0-28days | | | | | |

DPV concentrations in plasma were typically observed within one to four hours after ring insertion, indicating that DPV is readily absorbed into vaginal/cervical tissue and subsequently into the systemic circulation. Similar to the DPV vaginal fluid concentrations, DPV plasma concentrations at 24 hours after ring insertion were similar to those after 28 days of continuous use, suggesting that this may also occur in vaginal/cervical tissue. Maximum plasma concentrations were generally observed within 7 to 10 days and then tended to decrease steadily throughout the remainder of the ring use period (Figure 3). The highest DPV plasma concentration observed across all trials did not exceed 2 ng/mL. Since the mean maximum and average steady state plasma concentrations following the highest well-tolerated oral dose (300 mg twice daily) were 2,286 and 1,521 ng/mL, respectively, based on simulations using the developed population pharmacokinetic model, the 90% percentile of predicted maximum DPV ring plasma concentrations is approximately 3000 times (95% confidence interval [CI]: 2880 to 3100) below the abovementioned average oral plasma concentration. In the Phase III trials, where the ring was used continuously for up to approximately 2.5 years, with replacements every 4 weeks, DPV plasma concentrations were similar at all visits, indicating there is no systemic accumulation during continued use.^13^

Cervical tissue biopsies were collected in two trials, either after removal of the first DPV VR (Ring-004) used for 28 to 35 days (IPM 013) or after removal of the third DPV VR (Ring-004) (each used for 28 days consecutively [MTN-024/IPM 031]). In IPM 013, DPV concentrations were quantifiable in all participants and the median DPV concentration after 28-day use was 850 ng/g.^31^ In MTN-024/IPM 031, DPV concentrations were only detectable in five of the 10 participants (undetectable concentrations probably due to small biopsy samples). The median concentration in the samples with detectable concentrations was 600 ng/g.^13^

### Safety of Dapivirine

**Oral Dapivirine**

In a series of 11 oral DPV clinical trials, 211 participants were exposed to oral doses of DPV ranging from 50 mg to 1000 mg daily. The maximum tolerated dose (MTD) for a single dose was 350 mg and for multiple doses 300 mg b.i.d. No trials were stopped for safety reasons, and no deaths occurred during these trials. Treatment emergent adverse events (TEAE) reported in more than 2% of participants included headache, dizziness, nausea, diarrhea, fatigue, tremor, somnolence, flatulence and vomiting. Most (≥ 80%) of these TEAEs were Grade 1 or 2 (mild or moderate) and most (≥ 80%) were considered related to DPV. Grade 3 (severe) TEAEs included headache, dizziness, injury, nausea, tremor, paresthesia, disturbance in attention, abrasion, elevated liver enzymes AST and ALT, polyuria, fever, diarrhea, and vomiting. Elevated liver enzymes were transient and did not result in any liver impairment. Grade 3 increases in AST and ALT occurred in a single participant who received 50 mg b.i.d. oral DPV. One HIV-infected participant was withdrawn from the trial due to elevated AST and ALT, related to an acute concomitant hepatitis C infection. This event was the only serious adverse event (SAE) across all oral DPV trials, which led to a participant’s withdrawal from a trial.^13^

**Vaginally administered Dapivirine**

Considering the lack of significant local and systemic toxicities observed in ongoing and completed trials with vaginally administered DPV and comparing systemic exposure from orally administered to vaginally administered DPV, a wide safety margin exists for daily dosing of vaginally administered DPV.^32^

***Gel Studies***

In a series of eight Phase 1/2 DPV vaginal gel clinical trials that assessed PK, safety and acceptability endpoints, a total of 774 participants were enrolled of whom 491 participants used DPV vaginal gels of various concentrations. The trials were conducted in the United States, Belgium, Malawi, Kenya, Rwanda, South Africa, and Tanzania. Maximum exposure (days) to the gels ranged from 7 days to 84 days during these trials. The results of clinical trials with DPV gels showed that they were generally safe and well tolerated. Six participants required permanent discontinuation of the IP due to non-serious adverse events (AEs) that were regarded by the Investigator as at least possibly related to product use. The events included in the DPV treatment arm, a Grade 1 hypersensitivity (reported as allergic response with symptoms and signs that included vaginal burning, itching and erythema), Grade 1 worsening of a cervicovaginal human papilloma virus (HPV) infection, Grade 2 vulvar irritation along with vaginal pruritus in the same participant), and Grade 1 intermenstrual bleeding.^17^

Across all completed clinical gel trials, the most commonly reported TEAEs (documented in at least 5% of women who used DPV gel) were metrorrhagia, headache, bacterial vaginitis, and vaginal candidiasis.^17^

MTN-012/IPM 010 was a Phase 1 male tolerance trial that studied the safety of DPV gel (0.05%) among 48 (24 circumcised and 24 uncircumcised) healthy HIV-negative male participants aged 18 years or older. Participants were randomized 2:1:1 to apply DPV 0.05% gel, matched placebo gel, or universal placebo gel to their penis once daily for 7 sequential days. The safety of DPV 0.05% gel was assessed by the presence of Grade 2 or higher genitourinary adverse events (AEs) and systemic AEs. There were no Grade 2 genitourinary AEs in 47 participants completing the final clinical visit. There were 13 AEs reported; all were Grade 1 except one Grade 2 corneal laceration unrelated to study product. In summary, topical seven-day penile application of DPV 0.05% gel was locally and systemically safe, was acceptable to male participants, and resulted in systemic exposure to the drug.^33^

***DPV VRs (Phase 1/2 studies)***

Across all completed clinical trials, the DPV VRs (Rings 001, 002, 003 and 004) were generally safe and well tolerated. One participant assigned to DPV Ring-004 required permanent discontinuation of the IP due to a non-serious AE (Grade 2 generalized pruritus) that was classified by the investigator as possibly related to product use. Three Grade 3 SAEs (thoracic vertebral fractures, fracture of the right acetabulum and tonsillitis) were reported for three participants who used the DPV VR-004; all three events were classified by the investigator as unrelated to ring use.^13^

Six SAEs have been reported in participants using the placebo ring, including one participant who experienced two SAEs: Grade 3 (severe) bacterial gastroenteritis and Grade 4 (life-threatening) pyrexia and who was subsequently discontinued from the trial (IPM 024). Other reported SAEs in participants using placebo rings included a Grade 4 hemopneumothorax (following physical assault), which ultimately led to the participant’s death, a Grade 3 event of bronchiectasis, a Grade 3 peritonsillar abscess, and a Grade 3 psychiatric disorder (suicide attempt). None of the reported SAEs in placebo ring users were classified by the investigators as related to ring use.^13^

Across all completed DPV Ring-004 clinical trials (IPM 013, IPM 015, IPM 024, IPM 028 and IPM 034), the cumulative incidence of TEAEs was generally similar in the DPV ring and placebo ring groups. TEAEs that occurred in ≥ 5% of DPV Ring-004 users were metrorrhagia, headache, gynecological chlamydia (CT) infection, vaginal candidiasis, urinary tract infection (UTI), vaginal discharge, upper respiratory tract infection, lower abdominal pain, nasopharyngitis, and nausea. Metrorrhagia (29.7% vs. 24.4%), headache (15.1% vs. 11.9%) and vulvovaginal discomfort (2.7 vs. 1.3%) were reported more frequently in users of the DPV VR than the placebo ring respectively.^13^

Extended use of a single 25 mg DPV Ring-004 (IPM 034) for up to 84 days was considered generally safe and well tolerated. Forty HIV-negative female participants (eight per group) were instructed to use the ring continuously for 7, 14, 28, 56, or 84 days. There was no placebo arm. The majority of AEs were of mild or moderate intensity. Metrorrhagia was reported most frequently (2-5 participants [25-50%] per group), and all cases were reported by the investigator as “breakthrough bleeding.” Apart from metrorrhagia, the TEAEs reported most frequently (by at least 5.0% of participants using the DPV rings) were nasopharyngitis, lower abdominal pain, headache, vaginal discharge, oropharyngeal pain, nausea, and procedural pain. The only AEs reported in the Reproductive and Breast Disorders System Order Class were grade 1 (mild) vaginal discharge and metrorrhagia.^13^

***DPV VRs: Phase 3 studies***

**IPM 027 (The Ring Study):** The rate of AEs, including product-related AEs, urogenital AEs and deaths, was similar between the DPV and placebo groups. Although the rate of SAEs was statistically significantly higher in the DPV VR group than the placebo group (2.9% vs. 0.9%, p=0.008), no patterns were identified to indicate clinical relevance. The cumulative incidence of TEAEs was similar in DPV VR users (1142/1306; 87.4%) and placebo VR users (559/652; 85.7%) (Table 3). Gynecological CT infection was reported most frequently, with a similar incidence observed in the DPV and placebo VR groups. All cases were moderate (Grade 2) in severity. Apart from gynecological CT infection, the TEAEs reported most frequently (by at least 10% of participants using DPV VRs) were metrorrhagia, female genital infection, genitourinary tract gonococcal infection, upper respiratory tract infection, trichomoniasis, UTI, and vulvovaginal candidiasis.^13,29^ No clinically significant differences in the frequency of TEAEs between the DPV and placebo treatment groups were detected.^13,29^

Table 3: Incidence of Treatment-Emergent Adverse Events Reported Most Frequently (Incidence ≥ 10% in Participants Using DPV VRs), Regardless of Causality in IPM 027

| **MedDRA SOC/Preferred Term (MedDRA v 15.0)** | **Dapivirine (N=1307)** | **Placebo (N=652)** |
| --- | --- | --- |
|  | n (%) | n (%) |
| Participants with at least one TEAE | 1142 (87.4%) | 559 (85.7%) |
| INFECTIONS AND INFESTATIONS | | |
| Gynaecological chlamydia infection | 400 (30.6%) | 205 (31.4%) |
| Genital infection female* | 287 (22.0%) | 115 (17.6%) |
| Genitourinary tract gonococcal infection | 234 (17.9%) | 106 (16.3%) |
| Upper respiratory tract infection | 225 (17.2%) | 109 (16.7%) |
| Trichomoniasis | 217 (16.6%) | 95 (14.6%) |
| Urinary tract infection | 180 (13.8%) | 97 (14.9%) |
| Vulvovaginal candidiasis | 165 (12.6%) | 76 (11.7%) |
| REPRODUCTIVE SYSTEM AND BREAST DISORDERS | | |
| Metrorrhagia | 335 (25.7%) | 182 (27.9%) |
| * This term described events where there was a clinical suspicion of genital infection and syndromic treatment given but no etiology was confirmed. | | |

SAEs were reported by 44 participants (38 [2.9%] participants in the DPV VR group and six [0.9%] participants in the placebo VR group). The events varied in intensity from Grade 1 (mild) to Grade 5 (death); none were considered related to the VRs by the investigator. Two participants in the DPV VR group died due to multiple injuries sustained in a motor vehicle accident and a gunshot wound respectively, and one placebo VR user died as a result of circulatory collapse during an episode of substance abuse.^13^

Product-related events were reported for five (0.4%) participants in the DPV VR group and included metrorrhagia (two participants), pelvic discomfort, pelvic pain, and suprapubic pain (one participant for each). Three placebo VR users experienced a product-related event that included pelvic discomfort, menometrorrhagia and application site pain. All product-related events in both groups were assessed by the investigator as mild in severity.^13^ One participant assigned to the DPV VR group discontinued the trial early due to a Grade 2 non-product related AE (cervical dysplasia) which required further evaluation and treatment.^13^

**MTN-020 (ASPIRE):** No statistically significant differences were identified between the DPV VR and placebo VR arms in frequency of the primary safety endpoint (defined as the incidence of any SAE, any Grade 3 or 4 AE, and any Grade 2 AE that was assessed by the investigator as being related to the IP) or in other AEs commonly detected in the trial population (Table 4). The most commonly occurring AE was metrorrhagia. Other frequently reported AEs (occurring in ≥ 10% of trial participants) included genitourinary CT infection, menorrhagia, UTI, menometrorrhagia, vulvovaginal candidiasis, bacterial vaginosis (BV), vaginal discharge, upper respiratory tract infection, increased AST and ALT, abnormal weight loss, genitourinary tract gonococcal infection, trichomonal vulvovaginitis, vulvovaginal pruritus, pelvic pain, decreased hemoglobin, and decreased neutrophil count.^13,20^

Table 4: Adverse Events in MTN-020 (ASPIRE)

|  | **Placebo VR**  **(N=1316)** | **Dapivirine VR**  **(N=1313)** |
| --- | --- | --- |
|  | **n (%)** | **n (%)** |
| Primary safety endpoint* | 186 (14%) | 180 (14%) |
| Any SAE | 48 (4%) | 52 (4%) |
| Death | 3 (<1%) | 4 (<1%) |
| Any Grade 4 AE | 23 (2%) | 22 (2%) |
| Any Grade 3 AE | 162 (12%) | 151 (12%) |
| Any Grade 2 AE assessed as related | 9 (1%) | 7 (1%) |
| * The primary safety endpoint of the trial was defined as any SAE, any Grade 3 or Grade 4 AEs, and any Grade 2 AE assessed by the investigator as related to the IP. Overall chi-squared P-value = 0.80. | | |

SAEs were reported by 100 participants (52 [4.0%] participants in the DPV VR group and 48 [3.6%] participants in the placebo VR group). The events varied in intensity from Grade 1 (mild) to Grade 5 (death); none were considered related to IP by the investigators. Four deaths were reported in the DPV VR group: two participants died from fatal stab wounds, one participant died from an abdominal injury due to a physical assault, and one participant died from dyspnea considered secondary to a pulmonary embolism. Three participants in the placebo VR group died: one due to a fatal stab wound, one due to gastrointestinal tuberculosis, and one due to pulmonary tuberculosis.^13^

Grade 2 (moderate) product-related events (as assessed by the investigator) were reported for seven (0.5%) participants in the DPV VR group and included pelvic pain (reported for two participants), cervix erythema, cervix edema, cervicitis, UTI, urinary incontinence, dyspareunia, and headache, each reported for one participant. Nine (0.7%) placebo VR users experienced a Grade 2 product-related event that included application site pain (reported for two participants), pelvic inflammatory disease (PID), cervicitis, UTI, decreased neutrophil count, abnormal weight loss, dysmenorrhea, and pelvic pain, each reported for one participant. No Grade 3 (serious) or Grade 4 (potentially life threatening) product-related events were reported in the trial.^13^

### Efficacy of Dapivirine VR for Prevention of HIV

The safety and efficacy of the VR matrix containing DPV 25 mg (Ring-004) replaced monthly were tested in two randomized, double-blind, placebo-controlled Phase 3 trials: MTN-020 (ASPIRE) and IPM 027 (The Ring Study). Both trials demonstrated that the DPV VR was well tolerated and reduced the risk of HIV-1 infection by approximately 27% and 30%, respectively.^20,29^ In IPM 027, a total of 133 post-randomization HIV-1 infections occurred: 77 among women assigned to the DPV VR (incidence 4.08 per 100 person-years) and 56 among women assigned to placebo VR (incidence 6.10 per 100 person-years).^21^ The DPV VR reduced the risk of HIV-1 infection by 30.7% (95% CI: 0.90-51.5%; p=0.0401) relative to placebo VR. A 37.5% (95% CI: 3.5-59.5%) reduction in HIV-1 infection was observed in a subgroup analysis of women older than 21 years.^29^

In MTN-020, a total of 168 HIV-1 infections occurred: 71 among those assigned the DPV VR and 97 among those assigned the placebo VR (incidence 3.3 and 4.5 per 100 person-years, respectively).^20^ The DPV VR resulted in a 27% (95% CI: 1-46%, p=0.05) relative reduction in HIV-1 incidence overall, a 37% (95% CI: 12-56%, p=0.007) reduction in an analysis defined early in the study, excluding data from two study sites with lower retention and adherence, and a 56% (95% CI: 31-71%, p<0.001) reduction in a post-hoc analysis among women older than 21 years of age.^20^ In pre-defined as-randomized subgroup analyses, HIV-1 protection differed significantly by age, with a 61% reduced risk of HIV-1 infection for women ≥ 25 years [CI: 32%, 77%)] p<0.001, and 10% reduced risk for women < 25 years (CI: -41%, 43%) p=0.64. A post-hoc analysis was conducted to further explore this result, which indicated a 56% (95% CI: 31-71%, p<0.001) risk reduction among women older than 21 years of age. HIV-1 protection was not observed for women aged 18-21, and objective markers of adherence (plasma DPV levels and ring residual levels) were lower in this subgroup compared to women older than 21.^20^ Across multiple analyses, there was a statistically significant relationship between VR use and HIV protection. The ASPIRE results suggest VR use is associated with at least 56% and potentially >65% protection when used consistently.^34^ Finally, among those acquiring HIV-1, the detection of NNRTI mutations did not differ by study arm (8/68 [12%] assigned DPV and 10/96 [10%] assigned placebo, p=0.80). The frequency of ARV resistance was also similar between study arms.^20^

**Condom Compatibility**

Condom compatibility tests in which a variety of male and female condoms were exposed to gel containing 0.05% DPV did not identify any deleterious effects on condom integrity. Clinical condom functionality studies using a placebo VR showed an acceptable failure rate when compared to that for condom use without a VR.^13^

## Levonorgestrel

### Description

LNG is a second-generation progestin (synthetic progestogen), with chemical name (17a)-(-)-13-ethyl-17-hydroxy-18,19,dinorpregna-4-en-20-yn-3-one.^35^ LNG is used extensively as an active ingredient in hormonal contraceptives including combined oral contraceptive pills, emergency contraceptive pills, intrauterine systems, and contraceptive subdermal implants. LNG has a well-established safety profile with no significant safety findings reported from post-marketing experience, and demonstrated efficacy in many contraceptive formulations. LNG is being investigated in other multi-purpose prevention technologies under development.

### Mechanism of Action

LNG is a progestin that works primarily through cervical mucus thickening. LNG also decreases ovulation but does not completely inhibit ovulation in all women using currently approved effective LNG-based contraceptives. Other possible mechanisms of action include suppression of mid-cycle gonadotropin peaks and a variety of effects on the endometrium and/or fallopian tubes.

## Nonclinical Studies of Levonorgestrel

### Nonclinical Pharmacology

**Primary Pharmacology of Levonorgestrel**

LNG is a progestin that is widely used in a variety of hormonal contraceptive products. Progestins are synthetic forms of the hormone progesterone, and are classified as compounds that transform proliferative endometrium to secretory endometrium in estrogen-primed uteri. The pharmacologic action of LNG has been thoroughly reviewed in the literature, supporting the progestin activity of LNG with low androgen receptor affinity.^28^

**Secondary Pharmacology of Levonorgestrel**

Levonorgestrel had no effect on the activity of dapivirine against a laboratory adapted strain of HIV-1 in an in vitro model of cellular infectivity, suggesting that it is unlikely to affect the efficacy of dapivirine.^28^

### Nonclinical Pharmacokinetics

The PK, distribution, metabolism, and excretion of LNG were evaluated during the development programs for the marketed LNG-based contraceptive products. The only additional study to support the vaginal administration of LNG was a sheep study that is described in details in [Section 2.8.2](#_Pharmacokinetics)^28^.

### Nonclinical Toxicology

LNG has been approved for use in contraceptive products for more than 3 decades. Extensive programs of nonclinical safety studies supporting the initial registration of oral contraceptives containing norgestrel and LNG and of subdermal implants delivering LNG (Norplant® and Jadelle®) have been conducted and provide important safety information for the fixed-dose combination DPV-LNG VR. Additional safety assessments of the potential for LNG to cause vaginal irritation have been performed in rabbits, including 10 daily doses at concentrations up to 0.375 mg/mL using two different formulations in a Carraguard-based gel. All formulations were well tolerated with minimal signs of vaginal mucosal irritation. Results from all of the previous studies were considered adequate to support the delivery of LNG via a vaginal ring.^28^

## Clinical Studies of Levonorgestrel

### Pharmacokinetics of Levonorgestrel

LNG, a synthetic progestin, has been approved for use in contraceptive products for more than three decades. Clinical data for LNG have been gathered in numerous studies supporting the 67 currently approved LNG-containing products (prescription and over the counter).^35^ Jadelle® and Mirena® are long-acting contraceptives that deliver LNG subdermally or directly to the endometrium respectively and are approved for contraceptive use. Plan-B® is an oral LNG product approved as an emergency contraceptive. In the 1970s, studies on VRs delivering LNG alone or in combination with estrogen assessed the efficacy and safety of lower dose levels of LNG.^35^ The approved total dosage for a complete regimen of Plan B One-Step® is a single oral dose of 1.5 mg LNG, whereas the Mirena® IUS is loaded with 52 mg of LNG that is slowly released into the endometrium for up to 5 years of use. The Jadelle® implant system is comprised of two contraceptive rods, each loaded with 75 mg of LNG that are placed subdermally and release LNG slowly for up to 5 years of use.

**Intrauterine Device (IUD)**

There are four currently approved LNG-IUDs: Mirena®, Skyla®, Kyleena®, and Liletta®. For Mirena®, the 52-mg LNG IUDs, the initial release rate of LNG is 20 μg/day; this rate declines by about 50% after 5 years to approximately 10 μg/day. A stable plasma level, without peaks and troughs, of LNG of 150-200 pg/mL occurs after the first few weeks of use. LNG plasma concentrations after 12, 24 and 60 months were approximately 180 ± 66 pg/mL, 192 ± 140 pg/mL and 159 ± 59 pg/mL, respectively.^36^ For Skyla®, the 13.5mg LNG IUD, the initial release rate of LNG is 14 μg/day; this rate decreases to 5 μg/day after 3 years. In a subset of 7 subjects, maximum observed serum LNG concentration was 192 ± 105 pg/mL, reached after 2 days (median) of Skyla insertion. Thereafter, LNG serum concentration decreased after long-term use of 12, 24, and 36 months to concentrations of 77 ± 21 pg/mL, 62 ± 38 pg/mL, and 72 ± 29 pg/mL, respectively. A population pharmacokinetic evaluation based on a broader database (>1000 patients) showed similar concentration data of 168 ± 46 pg/mL at 7 days after placement. Thereafter, LNG serum concentrations decline slowly to a value 61 ± 19 pg/mL after 3 years.^37^

**Subdermal Implant**

For the LNG implant Jadelle®, the release rate is estimated to be 100 μg/day at 1 month, declining to about 40 μg/day at 12 months, and stabilizing at a rate of approximately 30 μg/day from 24 months onwards. Maximum plasma concentrations are reached within 2-3 days with the mean ± standard deviation being 772 ± 140 pg/mL at 2 days. After the initial phase, LNG concentrations decline to 435 ± 172 pg/mL at one month, 357 ± 155 pg/mL at 6 months and 280 ± 123 pg/mL at 3 years. Concentrations at 4 and 5 years are similar to those at 3 years.^28^

### Safety of Levonorgestrel

The safety profile for these products is well established and post-marketing experience has not identified any significant safety concerns over a wide dosing range. Implantable products such as Norplant® and Jadelle®, transdermal products like Climara Pro® and the intrauterine devices such as Mirena®, Skyla®, Kyleena®, and Liletta® provide support for the safety and tolerability of LNG delivered continuously from extended-release formulations. The extensive nonclinical and clinical data collected during the development of Norplant® and Jadelle® are summarized in NDA No. 19897 NORPLANT® LNG implants and NDA No. 20544 JADELLE® (NORPLANT® II) LNG implants respectively.^32^

In Jadelle® clinical trials, where calculated mean *in vivo* release rates of LNG were 100 ug/day at one month and declined to approximately 40 ug/day at 12 months, discontinuation rates at one year were 4.5 per 100 women for irregular bleeding. For most women, menstrual irregularities tended to diminish with prolonged use and despite changes in menstrual bleeding patterns, mean hemoglobin levels among Jadelle® users remained unchanged or increased. Experience among users of Norplant® (a subdermal LNG contraceptive system consisting of six capsules) has shown that only in rare cases did menstrual bleeding result in marked decreases in hemoglobin concentration.^32^

The only consistent change in liver function in users of Jadelle® has been a small increase in total bilirubin, with all mean values remaining within the normal range. Assessment of kidney function for Jadelle® included an evaluation of blood uric acid, urea nitrogen, sodium, potassium, calcium, and inorganic phosphorous. There were no indications of compromised kidney function. Some evidence was reported of a minor decrease in thyroxin and triiodothyronine levels in Jadelle® users but this was not accompanied by changes in free thyroxin.^32^

The most common adverse reactions (in >5% users) for other LNG-containing contraceptive devices are similar and include uterine/vaginal bleeding alterations (including amenorrhea, menorrhagia and intermenstrual bleeding), abdominal/pelvic pain, headache/migraine, acne, depressed/altered mood, breast tenderness/pain, vaginal discharge and nausea. Other rare and potentially more serious AEs associated with continued LNG use that have been reported are ectopic pregnancy, ovarian cysts, thrombosis, and idiopathic intracranial hypertension (particularly in obese participants).^32^

Some users of progestin-only oral contraceptives experience a slight deterioration in glucose tolerance, with increases in plasma insulin; however, the effect of LNG-containing implants on carbohydrate metabolism appears to be minimal. In a Norplant® post-marketing surveillance study, there was no significant difference in the development of diabetes mellitus among users of Norplant® compared to women who were using IUDs or who had been sterilized.

As a second-generation progestin, LNG favors progestogenic activity resulting in contraceptive efficacy. LNG has relatively low androgenic and glucocorticoid agonist activity, which are typically related to the side effects of acne, oily skin, and hair growth (androgenic), and bloating and weight gain due to salt and water retention (glucocorticoid). The relatively low sex hormone binding globulin (SHBG) binding activity of LNG (compared to testosterone) indicates a low venous thromboembolism (VTE) risk. Epidemiological data also suggest that progestin-only contraceptives, using second-generation progestins, such as LNG, have significantly lower risk of VTE risk compared to contraceptives containing estrogen and/or the newer third-generation progestins.^28^

A two-year longitudinal study undertaken by the WHO (1999) compared 177 users of Norplant® with a similar number of copper IUD users. Lipid changes were greatest three months after implant insertion, with a slow reversal of these trends during the next 19 months. The report concluded that lipid changes induced by Norplant® would probably not affect the risk of atherosclerotic disease in women who use this contraceptive method. In the WHO trial with a LNG vaginal ring (20 μg daily), no significant differences were observed in lipid/lipoprotein values or in glucose tolerance between the baseline and post removal assessment.^32^

In general, there have been no significant findings from laboratory safety evaluations with LNG products. Although the safety profile for vaginally administered LNG is less established compared to oral, subdermal, or intrauterine dosing, AEs reported with vaginal delivery of LNG include many AEs also observed during use of LNG subdermal implants.^32^

**Vaginal Rings**

A large WHO-sponsored trial evaluating a Silastic® 382, core design vaginal ring containing 5 mg LNG (20 μg/day release rate) enrolled 1,005 women. The ring was used continuously for 90 days. The most commonly reported AEs were menstrual disturbances (breakthrough bleeding, prolonged or heavy periods), vaginal discharge, vaginal infection, and vaginitis. Approximately 17.2% of women discontinued the trial early due to menstrual disturbances. There appeared to be no significant trend in the bleeding patterns over one year of product use. Users with the worst bleeding patterns tended to discontinue first during the clinical trial and were influenced by their more recent experience of vaginal bleeding irregularities. Although the number of bleeding days was increased in this study, total blood loss decreased after 12 months of use, and hemoglobin levels increased after 6 and 12 months.^38^

CONRAD recently completed the A13-128 trial, A Phase I One-Month Safety, Pharmacokinetic, Pharmacodynamic, and Acceptability Study of Intravaginal Rings Releasing Tenofovir and Levonorgestrel or Tenofovir Alone. A total of 51 participants from 2 sites, Eastern Virginia Medical School, Norfolk, VA, and Profamilia, Santo Domingo, Dominican Republic were randomized 2:2:1 to use tenofovir (TFV) (10 mg/d)-only ring, TFV/LNG ring, or placebo ring. The active LNG-containing VRs were anticipated to release 20 μg of LNG per day. The primary objectives of the trial were genital and systemic safety. The secondary objective of the trial was TFV and LNG pharmacokinetics. Conclusions based on preliminary analysis include: no safety concerns, TFV and LNG PK benchmarks met, LNG levels in cervical mucus similar to LNG 52 mg IUS users, TFV effect showing anti-HIV activity in collected specimens, ring performance within specifications, and high TFV-diphosphate concentrations in target tissues.^39,40^

## Studies of Levonorgestrel in Combination with Dapivirine

### Dapivirine Activity

The presence of LNG had no effect on DPV activity against a laboratory adapted strain of HIV-1 in an *in vitro* model of cellular infectivity, suggesting that it is unlikely to affect the efficacy of DPV.^32^

### Pharmacokinetics

A pharmacokinetic study in sheep was performed in which DPV-only and DPV-LNG combination rings were inserted vaginally for up to 15 days.^41^ Vaginal fluid and plasma concentrations were evaluated. Systemic exposure to DPV showed little change with increasing ring load of DPV (Table 5).^32^ For rings containing both DPV and LNG, C_max_ was generally similar to values for rings containing DPV alone and AUC values were higher, although the increase in AUC showed no correlation to the LNG load. In vaginal fluid, C_max_ and AUC values for DPV were higher for rings containing 200 or 530 mg DPV alone compared to the ring containing 75 mg DPV alone, and these values were even higher for rings that contained LNG and DPV, although again, the increase showed no correlation to LNG load. C_max_ and AUC values for LNG in plasma and vaginal fluid increased with increasing ring load of LNG, but the increase was less than proportional to the increase in load.^32^ LNG is reported to be ~55% bound to plasma proteins, a substrate for CYP3A4 metabolism and an inhibitor of CYP2B6.

Table 5: Pharmacokinetics of Dapivirine and Levonorgestrel in Plasma and Vaginal Fluid Following Vaginal Administration to Sheep

| **Ring Load (mg)** | |  | **Plasma** |  | **Vaginal Fluid** | | |
| --- | --- | --- | --- | --- | --- | --- | --- |
| DPV | LNG | C_max_  (pg/mL) | AUC_0-last_  (pg.h/mL) | T_max_  (h) | C_max_  (ng/mL) | AUC_0-last_  (ng.h/mL) | T_max_  (h) |
| **Dapivirine** | | | | | | | |
| 75 | - | 91.3 | 26201 | 72 | 1470 | 31447 | 6 |
| 200 | - | 99.8 | 23635 | 12 | 2840 | 212896 | 6 |
| 530 | - | 94.2 | 24559 | 12 | 2046 | 108163 | 6 |
| 200 | 32 | 117 | 38252 | 12 | 13800 | 492894 | 1 |
| 200 | 120 | 93.1 | 29878 | 360 | 9070 | 650172 | 1 |
| 200 | 800 | 103 | 30318 | 6 | 9070 | 255673 | 1 |
| **Levonorgestrel** | | | | | | | |
| 200 | 32 | 84.7 | 21009 | 1 | 8770 | 48332 | 1 |
| 200 | 120 | 199 | 46833 | 2 | 25100 | 146858 | 1 |
| 200 | 800 | 421.5 | 102614 | 4 | 61800 | 766028 | 1 |

*In vitro* release data for vaginal Ring-102 (containing 200 mg DPV and 320 mg LNG) and Ring-104 (containing 200 mg DPV) provide a very conservative (i.e. high over-estimate) assessment of the peak daily drug delivery of <8 mg in IPA:water and ~1.5mg in Na-acetate buffer with 2% solutol (Day 1 release). Similar conservative estimates of LNG release from VRs containing 200 mg DPV in combination with 32 mg (Ring-101) and 320 mg (Ring-102) of LNG indicate peak daily drug delivery of ~125 µg and ~400 µg respectively (Day 1 in Na-acetate buffer with 2% solutol).^32^

### Safety and Toxicity

Since neither DPV nor LNG have demonstrated any toxicity via the vaginal route, there is no basis on which to expect an interaction between the drugs in the fixed-dose combination product that would exacerbate the toxicity of either agent. Local levels of DPV in the vaginal vault may exceed the *in vitro* IC_50_ values for DPV. Competition of DPV for type A and B human progesterone receptor is not expected to interfere with progesterone even though DPV was found to bind to the recombinant progesterone receptors with a relative binding affinity (compared to progesterone at 100%) of only ~0.1% - 0.2%. LNG is reported to have a relative binding affinity at progesterone receptors of 323%.^28^ Given that LNG has an approximately 3230-fold higher affinity for progesterone receptors compared to DPV, it seems highly unlikely that the roughly 2-65-fold difference in vaginal concentrations will result in significant inhibition of LNG binding to local progesterone receptors in vaginal tissues. *In vitro,* there was no effect on the anti-HIV activity of DPV in the presence of LNG up to maximal soluble concentrations.^42^

Assessed together, these data suggest that it is unlikely that there will be any pharmacodynamic interactions between the two active ingredients that would compromise their efficacy. Data on the effects of each drug on cytochrome P450 enzymes also suggest that pharmacokinetic interactions are unlikely.

During the conduct of the MTN-030/IPM 041 study, a first-in-human Phase I clinical trial to determine the safety and PK profile of a vaginal ring containing 200 mg DPV alone or a combination of 200 mg DPV and 320 mg LNG (same ring to be used in this study), ongoing review of the study data did not identify any safety concerns or issues for either ring. There were no Grade 3, 4 or 5 AEs. There were 7 total Grade 2 AEs reported, 4 not related to the study VR and 3 related to the study VR (1 bacterial vaginosis, 1 headache, and 1 uterine cramping). Data analysis is still ongoing, however, the lack of concerning safety signals during the conduct of MTN-030/IPM 041 supports the continued development of this DPV-LNG VR.^43^

### Condom Compatibility

Condom compatibility tests in which a variety of male and female condoms were exposed to gels containing 0.05% DPV and 0.08% LNG did not identify any deleterious effects on condom integrity relative to placebo gel.

## Study Hypotheses and Rationale for Study Design

### Study Design

The design of MTN-044/IPM 053/CCN019, a clinical study of DPV-LNG VRs in women, will provide data on the PK profiles of DPV and LNG when the ring is worn continuously or cyclically (one cycle is approximately 28 days of continuous wear followed by 2 ring-free days) for approximately 90 days. Although full PK analysis has not yet been completed, initial results from MTN-030/IPM 041 indicated that peak plasma levels of DPV and LNG during 14 days of using this ring were well within the range deemed to be safe in prior oral dosing studies of each drug. In this study, it is anticipated that the levels of DPV and LNG will not exceed those previously identified as safe; however, close monitoring will be performed over the 90 days of planned product use enabling rapid to participant safety concerns. MTN-044/IPM 053/CCN019 will evaluate DPV and LNG concentrations in blood, vaginal fluid and cervical tissue during approximately 90 days of continuous or cyclical use. PK data will allow for determination of the concentration-time profiles using pooled data across all participants. The study design includes frequent collection of corresponding blood, CVF and cervical samples following monthly removal/reinsertion or continued use of a DPV-LNG VR to assess DPV and LNG levels in both usage patterns. PK parameters of DPV and LNG will be calculated for blood and CVF and of DPV only for cervical tissue. It is important to note that the goal of this study is not to show a comparative difference in safety, but to characterize what AEs are experienced and assess bleeding rates. For this reason, a control, or placebo arm, was not included.

Results from this study may support future, more complex study designs that include assessments of markers of contraceptive efficacy and acceptability of LNG when combined with an antiretroviral such as DPV in a vaginal ring. Specifically, this study will determine the associated bleeding patterns with DPV-LNG VR use over 90 days and the bleeding patterns that optimize acceptability and adherence to this MPT product.

### Study Hypotheses

- DPV and LNG concentrations in blood and CVF, and DPV concentration in cervical tissue will be measurable in all participants at all time points
- DPV concentrations in blood, CVF and cervical tissue will not be significantly lower following 2 days of ring removal in the cyclic ring users compared to the continuous ring users
- Both continuous and cyclic exposure to DPV/LNG VR containing 200 mg DPV + 320 mg LNG VR for approximately 90 days will be safe

# OBJECTIVES

## Primary Objective

**Pharmacokinetics**

- To characterize the local and systemic pharmacokinetics of one DPV-LNG vaginal ring formulation used either continuously or cyclically (~28/2) for approximately 90 days

## Secondary Objectives

**Safety**

- To evaluate the safety of one DPV-LNG vaginal ring formulation used either continuously or cyclically (~28/2) for approximately 90 days

## Exploratory Objectives:

**Bleeding**

- To assess vaginal bleeding patterns associated with one DPV-LNG vaginal ring formulation used either continuously or cyclically (~28/2) for approximately 90 days

**Acceptability**

- To explore women’s experience with continuous or cyclic use of one DPV-LNG vaginal ring, including bleeding, tolerability and other measures of ring acceptability

**Adherence**

- To evaluate participant adherence to one DPV-LNG vaginal ring formulation used either continuously or cyclically (~28/2) for approximately 90 days

**Vaginal Microenvironment**

- To describe the genital microenvironment in HIV-uninfected women during approximately 90 days of continuous or cyclic use of one DPV-LNG vaginal ring

**Pharmacodynamics**

- To evaluate the HIV inhibitory activity in cervical tissue over approximately 90 days during continuous or cyclical use of one DPV-LNG vaginal ring

# STUDY DESIGN

## Identification of Study Design

MTN-044/IPM 053/CCN019 is a Phase 1, two-arm, open-label, single-site, randomized trial of one silicone elastomer matrix VR containing a combination of the active ingredients of 200 mg DPV and 320 mg of LNG. Healthy, HIV-uninfected women aged 18-45 (inclusive) will use the DPV/LNG VR either continuously or cyclically (one cycle is approximately 28 days of continuous wear followed by 2 ring-free days) for approximately 90 days. Randomized participants will know their group assignment to continuous use or cyclic use of the VR.

## Primary Endpoints

**Pharmacokinetics**

- DPV and LNG concentrations in plasma
- DPV and LNG concentrations in cervicovaginal fluid
- DPV concentration in cervical tissue

## Secondary Endpoints

**Safety**

- Grade 2 or higher genitourinary adverse events as defined by the Division of AIDS (DAIDS) Table for Grading the Severity of Adult and Pediatric Adverse Events, Corrected Version 2.1, July 2017, and/or Addendum 1 (Female Genital Grading Table for Use in Microbicide Studies [Dated November 2007])
- Grade 3 or higher adverse events as defined by the Division of AIDS (DAIDS) Table for Grading the Severity of Adult and Pediatric Adverse Events, Corrected Version 2.1, July 2017

## Exploratory Endpoints:

**Bleeding**

- The total number of days of vaginal bleeding
- The total number of bleeding episodes

**Acceptability**

- Self-reported attitudes about ring attributes, use regimens and tolerability

**Adherence**

- Frequency and duration of study vaginal ring removal (voluntary and involuntary)
- Drug pharmacokinetic levels
- Residual drug levels (DPV and LNG) in returned vaginal rings

**Vaginal Microenvironment**

- Changes in microenvironment (microbiota and biomarkers)

**Pharmacodynamics**

- Anti-HIV-1 activity in cervical tissue

## Description of Study Population

The study population will be healthy, HIV-uninfected women who meet the criteria outlined in [Sections 5.2](#_Inclusion_Criteria) and [5.3](#_Exclusion_Criteria).

## Time to Complete Accrual

Accrual is expected to be complete in approximately 6 months.

## Study Groups

Approximately 24 participants will be randomized in a 1:1 ratio to one of the following study regimens, using one VR (200 mg DPV and 320 mg LNG):

- Regimen A: VR used continuously for approximately 90 days
- Regimen B: VR used cyclically for approximately 90 days as follows: used for ~28 days, then removed, washed and stored for 2 days. The same ring will be used for 2 additional similar cycles.

## Expected Duration of Participation

The expected trial duration for each enrolled participant is approximately 26 weeks.

## Site

One US site

# STUDY POPULATION

## Selection of the Study Population

The inclusion and exclusion criteria in [Sections 5.2](#_Inclusion_Criteria) and [5.3](#_Exclusion_Criteria) will be utilized to ensure the appropriate selection of study participants.

### Recruitment

Participants will be recruited from a variety of sources at the site, including family planning and gynecological offices, colleges and universities, online websites, faith communities, as well as community-based locations such as community-based organizations and street-based outreach. In addition, participants may be referred to the study from other local research projects and other health and social service providers. The site Institutional Review Board (IRB) will approve the site recruitment plan and all recruitment materials prior to use. Advice regarding these materials will be sought from site community representatives before they are submitted to the IRB for review.

### Retention

Once a participant is enrolled and randomized in MTN-044/IPM 053/CCN019, the study site will make every effort to retain the participants in follow-up to minimize possible bias associated with loss-to-follow-up. An average retention rate of 95% will be targeted. The study site will be responsible for developing and implementing local standard operating procedure (SOPs) to achieve this. Engaging peer educators/advocates or organizations in retention messaging, etc. may be used to facilitate MTN-044/IPM 053/CCN019 retention.

## Inclusion Criteria

Participants must meet all of the following criteria to be eligible for inclusion in the study:

1. Assigned female sex at birth

*Note: Participants who are female at birth, who now identify as male, will not be excluded so long as they are not currently or have not been on female-to-male transition therapy 90 days prior to Enrollment.*

1. Age 18 through 45 years (inclusive) at Screening, verified per site SOPs
2. Able and willing to provide written informed consent to be screened for and enrolled in MTN-044/IPM 053/CCN019
3. Able and willing to provide adequate locator information, as defined in site SOPs
4. Able to communicate in spoken and written English
5. Available for all visits and able and willing to comply with all study procedural requirements
6. Willing to abstain from receptive intercourse (vaginal, oral, and finger stimulation) and tampon use for 24 hours preceding the Enrollment Visit and clinical visits where samples are taken and for 1 week following each cervical biopsy visit
7. Not at risk for pregnancy, defined as consistently using an effective, non-hormonal method of contraception per participant report at Enrollment, and intending to continue use of an effective, non-hormonal method for the duration of study participation. Effective methods include:
   1. Non-hormonal (e.g. copper) intrauterine device (IUD) inserted at least 28 days prior to Enrollment
   2. Consistent and correct male condom use*
   3. Sterilization (of participant or partner, as defined in site SOPs)
   4. Having sex exclusively with individuals assigned female sex at birth
   5. Sexually abstinent for 90 days prior to Enrollment, and intending to remain abstinent for the duration of study participation

* Details regarding this criterion will be specified in the MTN-044/IPM 053/

CCN019 Study Specific Procedures (SSP) Manual

1. In general good health as determined by the Investigator of Record (IoR)/designee at Screening and Enrollment
2. HIV-uninfected based on testing performed at Screening and Enrollment (per protocol algorithm in [Appendix II](#_APPENDIX_II:__1))

1. Per participant report at Screening, current regular menstrual cycles of approximately 21 to 35 days in duration with no reported intermenstrual bleeding
2. Intact uterus with at least one ovary
3. Per participant report at Screening and Enrollment, states a willingness to refrain from inserting any non-study vaginal products or objects into the vagina including, but not limited to spermicides, female condoms, diaphragms, other intravaginal rings, vaginal medications, menstrual cups, cervical caps or any other vaginally applied barrier contraceptive method, vaginal douches, lubricants, moisturizers and sex toys (e.g., vibrators, dildos, etc.) for the 24 hours preceding the Enrollment Visit through completion of Visit 15.
4. Participants over the age of 21 (inclusive) must have documentation of a satisfactory Pap within the past 3 years prior to Enrollment consistent with Grade 0 according to the Female Genital Grading Table for Use in Microbicide Studies Addendum 1 (Dated November 2007) to the DAIDS Table for Grading Adult and Pediatric Adverse Events, Corrected Version 2.1, July 2017, or satisfactory evaluation with no treatment required of Grade 1 or higher Pap result
5. At Screening and Enrollment, agrees not to participate in other research studies involving drugs, medical devices, vaginal products or vaccines after the Screening Visit through completion of Visit 15

## Exclusion Criteria

Participants who meet any of the following criteria will be excluded from the study:

1. Body mass index greater than 40 kg/m^2^ at Screening
2. Pregnant at Screening or Enrollment or plans to become pregnant during the study period

*Note: A documented negative pregnancy test performed by study staff is required for inclusion; however, a self-reported pregnancy is adequate for exclusion from the study.*

1. Diagnosed with symptomatic urinary tract infection (UTI) or reproductive tract infection (RTI) at Screening or Enrollment

*Otherwise eligible participants diagnosed with symptomatic UTI/RTI during screening will be offered treatment. If treatment is complete and symptoms have resolved within the 60-day screening window, eligible participants may be enrolled.*

1. Diagnosed with an acute STI requiring treatment per current Centers for Disease Control and Prevention (CDC) guidelines (http://www.cdc.gov/std/treatment/) at Screening or Enrollment such as gonorrhea (GC), chlamydia, trichomonas, pelvic inflammatory disease (PID), and/or syphilis

*Note: Genital warts requiring treatment and frequent recurrence of herpes simplex virus (HSV) are considered exclusionary; however, infrequent HSV outbreaks are not. Genital warts requiring treatment are defined as those that cause undue burden or discomfort to the participant, including bulky size, unacceptable appearance, or physical discomfort. See MTN-044/IPM 053/CCN019 SSP Manual for additional information.*

1. Has a clinically apparent Grade 2 or higher pelvic exam finding (observed by study staff) at Screening or Enrollment, as per the DAIDS Table for Grading the Severity of Adult and Pediatric Adverse Events, Corrected Version 2.1, July 2017, and/or Addendum 1 (Female Genital Grading Table for Use in Microbicide Studies [Dated November 2007])

*Note: Cervical bleeding associated with speculum insertion and/or specimen collection judged to be within the range of normal according to the clinical judgment of the IoR/designee is considered expected non-menstrual bleeding and is not exclusionary.*

*Note: Otherwise eligible participants with exclusionary pelvic exam findings may be enrolled/randomized after the findings have improved to a non-exclusionary severity grading or resolved within 60 days of providing informed consent for screening.*

1. Participant report and/or clinical evidence of any of the following:
   1. Known adverse reaction to any component of the study product (ever)
   2. Chronic and/or recurrent vaginal candidiasis
   3. Has a contraindication to a progestin-only contraceptive method as defined by a category 3 or 4 condition according to the CDC *U.S. Medical Eligibility Criteria for Contraceptive Use, 2016^44^*
   4. Use of hormonal contraception, including hormonal IUD and implants within the 28 days prior to Enrollment
   5. Current use or planned use of CYP3A inhibitors and inducers
   6. Current use or planned use of antibiotics and/or corticosteroids that may interact with levonorgestrel
   7. Depot medroxyprogesterone acetate (DMPA) use in the 6 months prior to Enrollment or any prior use without return of regular spontaneous menstrual cycles.
   8. Non-therapeutic injection drug use in the 12 months prior to Enrollment
   9. Post-exposure prophylaxis (PEP) for HIV exposure within the 3 months prior to Enrollment
   10. Pre-exposure prophylaxis (PrEP) for HIV prevention within the 3 months prior to Enrollment
   11. Last pregnancy outcome less than 60 days prior to Enrollment
   12. Gynecologic or genital procedure (e.g., tubal ligation, dilation and curettage, piercing) 45 days or less prior to Enrollment

*Note: Pap test at the Screening Visit, colposcopy and cervical biopsies for evaluation of an abnormal Pap test as well as IUD insertion/removal are not exclusionary.*

- 1. Currently breastfeeding or planning to breastfeed during the study period
  2. Participation in any other research study involving drugs, medical devices, vaginal products or vaccines, in the 60 days prior to Enrollment

1. Has any of the following Grade 1 or higher laboratory abnormalities at Screening Visit:
   1. AST or ALT*
   2. Creatinine*
   3. Hemoglobin*

*Note: Otherwise eligible participants with an exclusionary laboratory result may be re-tested and may be enrolled/randomized after the findings have improved to a non-exclusionary severity grading or resolved within 60 days of providing informed consent for screening. Results of safety laboratory testing performed at the Enrollment Visit are expected to be received after the Enrollment Visit, and thus will not be exclusionary. Abnormal results will be noted as pre-existing conditions, and may result in product discontinuation, per IoR discretion as per Section 9.3 of the protocol.*

*DAIDS Table for Grading the Severity of Adult and Pediatric Adverse Events Corrected Version 2.1, July 2017, and/or Addendum 1 (Female Genital Grading Table for Use in Microbicide Studies [Dated November 2007])

1. Has any other condition that, in the opinion of the IoR/designee, would preclude informed consent, make study participation unsafe, complicate the interpretation of study outcome data, or otherwise interfere with achieving the study objectives including any significant uncontrolled active or chronic medical condition.

## Co-enrollment Guidelines

As indicated in [Sections 5.2](#_Inclusion_Criteria) and [5.3](#_Exclusion_Criteria), participants must not take part in other research studies involving drugs, medical devices, vaginal products or vaccines after the Screening Visit and while taking part in MTN-044/IPM 053/CCN019 unless approved by the Protocol Safety Review Team (PSRT) and Protocol Chair. Participation in the following types of studies may be allowed at the discretion of the IoR/designee after consultation with the Protocol Chair and PSRT:

- Participants may take part in MTN ancillary studies
- Participants who become infected with HIV may take part in observational and/or interventional studies for HIV-positive persons

Should any participant report concurrent participation in contraindicated studies after enrolling in MTN-044/IPM 053/CCN019, the IoR/designee will consult the PSRT regarding ongoing product use and other potential safety considerations associated with co-enrollment.

# STUDY PRODUCT

## Regimen

Each participant will be randomized (1:1) to one of two study regimens:

Table 6: Study Regimen

| **Regimen** | **N** | **Regimen Description** |
| --- | --- | --- |
| A | 12 | VR used continuously for approximately 90 days |
| B | 12 | VR used cyclically for approximately 90 days as follows: inserted for ~28 days, then removed, washed and stored for 2 days. The same ring will be inserted for 2 additional cycles in a similar fashion. |

Each participant will receive a VR containing the active ingredients dapivirine (DPV) and levonorgestrel (LNG), formulated as IPM VR: 200 mg of DPV + 320 mg LNG.

## Administration

At the Enrollment Visit, the VR will be inserted by the participant (or clinician/designee, if preferred or necessary). Participants will be given detailed instructions in the clinic on proper VR insertion and removal procedures. Additional details on administration procedures in the event of expulsion or loss and cleaning will be provided to the participant. Additional details regarding VR administration and storage procedures for Regimen B participants will be provided in the MTN-044/IPM 053/CCN019 SSP Manual.

## Study Product Formulation

The rings are designed to provide sustained release of drug(s) over a 90-day period. The dapivirine-levonorgestrel silicone elastomer vaginal matrix ring is a white flexible ring containing 200 mg of DPV and 320 mg (Ring-102) of LNG dispersed in a platinum-cured DDU-4320 silicone matrix. The dimensions of the ring are 57.1 mm (outer diameter) and 7.9 mm (cross sectional diameter).

## VR Storage and Dispensing

The recommended storage condition for VRs containing LNG is 2-8°C. Study VRs will be dispensed only to clinic staff on behalf of an enrolled participant, upon receipt of a written prescription from an authorized prescriber. Dispensation of one VR will take place on the day of enrollment. Provisions for the dispensation of additional VRs will be at the discretion of the IoR, in consultation with the PSRT.

## Supply and Accountability

### Supply

IPM (Silver Spring, MD) will oversee the manufacture and analysis/release of all study VRs under Good Manufacturing Practices (GMP).

### Accountability

Each Clinical Research Site (CRS) Pharmacist of Record (PoR) is required to maintain a complete record of all study VRs received. The procedures to be followed are provided in the MTN-044/IPM 053/CCN019 Pharmacy Study Product Management Procedures Manual. The clinic staff will document all VRs provided to the participants. The clinic staff will also document when the ring is returned or removed in the clinic. Any VRs not returned must also be documented by the clinic.

### Retrieval of Used Study Product

Study participants will be instructed to return for VR removal or to bring the removed ring at the Product Use End Visit (PUEV)/Early Study Termination Visit if the VR was self-discontinued. If the participant removed the VR and it is not returned at the PUEV/Early Study Termination Visit, site staff members will make every effort to encourage participants to return the VR as soon as possible (optimally within 5 working days). Attempts by study staff to retrieve the VR from the participant must be documented. If the VR is not returned within the time frames outlined below, the MTN-044/IPM 053/CCN019 PSRT must be notified. When product use is permanently discontinued for HIV infection or pregnancy, the VR must be retrieved (optimally within 24 hours) and returned to the clinic (see table below). Additional VR retrieval specifications in response to discontinuations for other reasons, or IoR instruction, can be found in Table 7. Study product retrieval should occur within the specified timeframe. Attempts should be made by study staff to contact the participant and retrieve the VR as soon as possible when not returned as expected.

Table 7: Retrieval of VR

| **Reason for Ring Removal** | **Timeframe for Study Product Retrieval** |
| --- | --- |
| Permanent discontinuation or temporary hold due to potential HIV infection or pregnancy | Within 24 hours |
| Permanent discontinuation for any other reason or IoR discretion | Within 5 working days |
| Temporary hold for any other reason with expected duration of greater than 7 days | Within 7 working days |

If there is a product hold, expulsion (i.e., ring removed and cannot be reinserted) or an extended period of time that the ring is removed, the MTN-044/IPM 053/CCN019 PSRT should be notified. The PSRT will evaluate each reported event to determine if the ring should be reinserted, if a new ring should be dispensed, or if the participant should discontinue product use. See [Section 9](#_General_Criteria_for).3 for additional information related to product holds.

## VR Use Instructions

Participants will receive VR use instructions at the Enrollment Visit and at additional follow-up visits, as needed. Site staff will counsel participants on VR use, including instruction to refrain from removing the ring (except as directed) and instructions for re-insertion in case of accidental ring expulsion, product use Regimen B, etc. Additional details will be provided in the MTN-044/IPM 053/CCN019 SSP Manual. Participants will also be counseled on the use of non-study intravaginal products and other devices as described in [Section 6.9](#_Use_of_Intravaginal).

## Concomitant Medications

Enrolled study participants may use concomitant medications during study participation with the exception of medications and products listed as prohibited. All concomitant medications reported throughout the course of the study (from enrollment until Visit 15) will be recorded in the study database. All prescription medications, over-the-counter preparations, vitamins, nutritional supplements, and herbal preparations will be recorded as concomitant medications.

## Prohibited Medications

Several concomitant medications/practices will not be permitted. Participants are prohibited from using strong CYP3A inhibitors and inducers. These medications are not permitted because DPV and LNG are CYP3A substrates. Use of heparin, including Lovenox® (enoxaparin sodium), Coumadin® (warfarin), Plavix® (clopidogrel bisulfate), hormone-replacement therapy, and chronic use of non-steroidal anti-inflammatory drugs (NSAIDS) is prohibited during study participation. Participants will be counseled to abstain from using aspirin (greater than 81 mg) and any other drugs (not including acute use of NSAIDS) that are associated with increased likelihood of bleeding for 72 hours before and after the collection of the cervical biopsies.

A listing of the specific prohibited agents is provided in the MTN-044/IPM 053/CCN019 SSP Manual available at www.mtnstopshiv.org. This listing is for guidance and may not necessarily be all-inclusive. If drug-drug interaction questions arise during the study that cannot be answered by any of the study-related materials provided (protocol, SSP, SOPs), please contact the MTN-044/IPM 053/CCN019 PSRT by sending a message to mtn044psrt@mtnstopshiv.org. Medications with unknown interactions will be addressed on a case-by-case basis with input from the PSRT, as needed.

## Use of Intravaginal Medications/Products and Practices

All participants will be counseled to avoid the use of non-study intravaginal products and other devices. These include, but are not limited to, spermicides, female condoms, diaphragms, other intravaginal rings, vaginal medications, menstrual cups, cervical caps, douches, lubricants, and sex toys (e.g., vibrators, dildos, etc.) for the 24 hours preceding the Enrollment Visit through completion of Visit 15. Use of these products will be captured in the study database. Participants who report use of these products during study product use periods will be counseled regarding the use of alternative methods and study staff should reference [Section 9.3](#_General_Criteria_for) for permanent discontinuation guidelines. Participants are expected to be sexually abstinent i.e., no receptive intercourse (vaginal, oral and finger stimulation) and no tampon use for the 24 hours preceding the Enrollment Visit and clinical visits where samples are taken and for one week following each cervical biopsy.

# STUDY PROCEDURES

An overview of the study visits and evaluations schedule is provided in [Appendix I](#_APPENDIX_I:_). Presented in this section is additional information on visit-specific study procedures. Detailed instructions to guide and standardize procedures as well as to specify the visit windows are provided in the MTN-044/IPM 053/CCN019 SSP Manual available at www.mtnstopshiv.org.

Figure 4: Study Visit Schedule


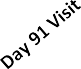

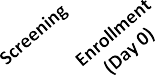

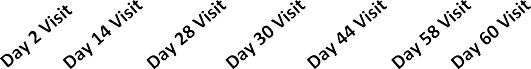

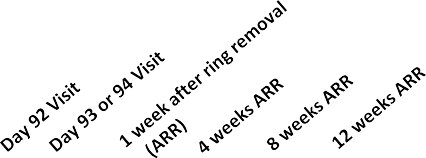

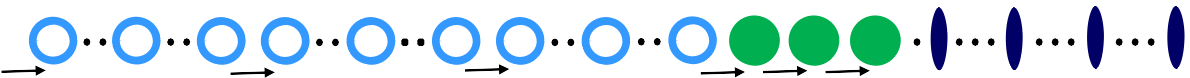

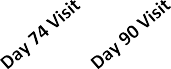


***≤*60**

**days**

2 days

2 days

2 days

1 day 1 day 1 or 2 days

**Visit 1**

**V2 V3**

**V4**

**V5 V6**

**V7**

**V8 V9**

**V10 V11 V12 V13 V14 V15 V16 V17 V18**

**(up to 4 phone calls/contacts)**

## Pre-screening

As part of participant outreach and recruitment strategies, study staff may pre-screen potential study participants at either on-site or off-site locations. During these interactions, study staff may explain the study to potential participants and ascertain elements of presumptive eligibility, to be confirmed at an on-site screening visit. Process information (e.g., number of potential participants contacted, number presumptively eligible) may be recorded and stored at the study site in the absence of written informed consent from potential participants, provided the information is collected in such a manner that it cannot be linked to participant identifiers, unless a waiver is granted from the site IRB. At the study site, procedures and documentation will comply with site IRB requirements.

## Screening Visit - Visit 1

A Screening Visit may take place up to 60 days prior to the Enrollment Visit (Day 0). Multiple visits may be conducted to complete all required screening procedures, if necessary. Written informed participant consent for Screening/Enrollment will be obtained at the Screening Visit before any screening procedures are initiated. For participants who do not meet the eligibility criteria, screening will be discontinued once ineligibility is determined.

*NOTE: Participants who fail their first screening attempt may be re-screened one time.*

Table 8: Screening Visit - Visit 1

| **Screening Visit - Visit 1** | | |
| --- | --- | --- |
| **Component** | | **Procedure/Analysis** |
| **Administrative and Regulatory** | | - - Obtain written informed consent   - Assign a unique Participant Identification (PTID) number   - Assess eligibility   - Collect demographic and background information   - Collect locator information   - Provide reimbursement   - Schedule next visit/contact* |
| **Behavioral/Counseling** | | - - HIV pre- and post-test counseling   - HIV/STI risk reduction counseling |
| **Clinical** | | - - Collect medical eligibility information   - Collect medical and menstrual history   - Collect concomitant medications   - Perform physical examination   - Perform pelvic examination   - Perform breast examination   - Treat or prescribe treatment for RTI/UTI/STIs*   - Provide available test results |
| **Laboratory** | **Urine** | - - Pregnancy test   - Urine dipstick/culture* |
|  | **Blood** | - - HIV testing   - Serum creatinine   - Complete blood count (CBC) with differential and platelets   - AST/ALT   - Syphilis serology |
|  | **Pelvic** | - - NAAT for GC/CT and trichomonas   - Pap test^   - Genital lesion testing for HSV*   - Saline/potassium hydroxide (KOH) wet mount with pH for candidiasis and/or BV* |
| **Study Product Supply** | | - Offer male condoms**☼** |

* If indicated and/or per local standard of care

^ if indicated (if participant over age 21 is unable to provide documentation of a satisfactory Pap test within 3 years

prior to enrollment)

**☼** Details are specified in the SSP

## Enrollment Visit - Visit 2 (Day 0)

All Enrollment procedures must be performed on the same day. The participant’s menstrual cycle must be considered when scheduling Visit 2 - Enrollment (Day 0). Ideally, no bleeding should occur within the first 3 days of product use, e.g., Study Visits 2 and 3 (Days 0 and 2).

Table 9: Enrollment Visit - Visit 2 (Day 0)

| **Enrollment Visit - Visit 2 (Day 0)** | | |
| --- | --- | --- |
| **Component** | | **Procedure/Analysis** |
| **Administrative and Regulatory** | | - - Assess and confirm eligibility   - Review/update locator information   - Randomization   - Provide reimbursement   - Schedule next visit/contact* |
| **Behavioral/Counseling** | | - - HIV pre- and post-test counseling   - HIV/STI risk reduction counseling   - Protocol counseling   - Contraceptive counseling   - Behavioral assessment |
| **Clinical** | | - - Collect medical eligibility information   - Review/update medical and menstrual history   - Review/update concomitant medications   - Perform physical examination   - Perform pelvic examination   - Perform breast examination   - Digital examination by clinician to place or check VR   - Treat or prescribe treatment for RTI/UTI/STIs*   - Provide available test results   - Collect AEs **π** |
| **Laboratory** | **Urine** | - - Pregnancy test   - Urine dipstick/culture* |
|  | **Blood** | - - HIV testing   - Serum creatinine   - CBC with differential and platelets   - AST/ALT   - Plasma for archive   - Syphilis serology*   - DPV concentration▲   - Progestogen concentrations including LNG▲   - Sex hormone-binding globulin (SHBG) and albumin   - Serum progesterone and estradiol |
|  | **Pelvic** | - - NAAT for GC/CT and trichomonas*   - Saline/KOH wet mount with pH for candidiasis and/or BV*   - Genital lesion testing for HSV*   - Vaginal Gram stain   - CVF DPV concentration▲   - CVF LNG concentration▲   - CVF for microbiota▲   - Cervicovaginal lavage (CVL) for biomarkers▲ |
| **Study Product Supply** | | - - Provision of one study VR   - Insertion of the provided study VR by the participant or clinician/designee   - Provision of study VR use instructions   - Offer male condoms**☼** |

* If indicated and/or per local standard of care

▲ To be collected at baseline prior to ring insertion

**π** To be collected following randomization

**☼** Details are specified in the SSP

## Follow-up Visits

The target date for each follow-up visit is listed below. Visit windows will be specified in the MTN-044/IPM 053/CCN019 SSP Manual (www.mtnstopshiv.org). If a participant is unable to complete scheduled study visits within the visit window, any visit procedures missed may be conducted during an interim visit (see [Section 7.6](#_Interim_Visits) for additional details).

### Day 2 and Day 14 - Visits 3 and 4

Table 10: Day 2 and Day 14 - Visits 3 and 4

| **Day 2 and Day 14 - Visits 3 and 4** | | |
| --- | --- | --- |
| **Component** | | **Procedure/Analysis** |
| **Administrative and Regulatory** | | - - Review/update locator information   - Provide reimbursement   - Schedule next visit/contact |
| **Behavioral/Counseling** | | - - HIV pre- and post-test counseling*   - HIV/STI risk reduction counseling*   - Protocol counseling   - Contraceptive counseling |
| **Clinical** | | - - Review/update medical and menstrual history   - Review/update concomitant medications   - Perform targeted physical examination*   - Perform pelvic examination* (Day 14 mandatory)   - Perform breast examination*   - Treat or prescribe treatment for RTI/UTI/STIs*   - Provide available test results   - Collect AEs |
| **Laboratory** | **Urine** | - - Pregnancy test* (Day 14 mandatory)   - Urine dipstick/culture* |
|  | **Blood** | - - HIV testing*   - Serum creatinine*   - CBC with differential and platelets*   - AST/ALT*   - Syphilis serology*   - Serum progesterone and estradiol   - DPV concentration   - Progestogen concentrations including LNG |
|  | **Pelvic** | - - NAAT for GC/CT and trichomonas*   - Saline/KOH wet mount with pH for candidiasis and/or BV*   - Genital lesion testing for HSV*   - CVF DPV concentration   - CVF LNG concentration   - Cervical biopsies for DPV concentration and PD (Day 14 only) |
| **Study Product Supply** | | - - Offer male condoms**☼** |

* If indicated and/or per local standard of care

**☼** Details are specified in the SSP

### Day 28 and Day 58- Visits 5 and 8

Table 11: Day 28 and Day 58 - Visits 5 and 8

| **Day 28 and Day 58 - Visits 5 and 8** | | |
| --- | --- | --- |
| **Component** | | **Procedure/Analysis** |
| **Administrative and Regulatory** | | - - Review/update locator information   - Provide reimbursement   - Schedule next visit/contact |
| **Behavioral/Counseling** | | - - HIV pre- and post-test counseling*   - HIV/STI risk reduction counseling*   - Protocol counseling   - Contraceptive counseling |
| **Clinical** | | - - Review/update medical and menstrual history   - Review/update concomitant medications   - Perform targeted physical examination*   - Perform pelvic examination*   - Perform breast examination*   - Treat or prescribe treatment for RTI/UTI/STIs*   - Provide available test results   - Collect AEs |
| **Laboratory** | **Urine** | - - Pregnancy test*   - Urine dipstick/culture* |
|  | **Blood** | - - HIV testing*   - Serum creatinine* (Day 28 mandatory)   - CBC with differential and platelets* (Day 28 mandatory)   - AST/ALT* (Day 28 mandatory)   - Syphilis serology*   - Serum progesterone and estradiol   - DPV concentration ⌂   - Progestogen concentrations including LNG ⌂ |
|  | **Pelvic** | - - NAAT for GC/CT and trichomonas*   - Saline/KOH wet mount with pH for candidiasis and/or BV*   - Genital lesion testing for HSV*   - CVF DPV concentration ⌂   - CVF LNG concentration ⌂ |
| **Study Product Supply** | | - - Removal and storage of study VR (Group 2 only, record exact time of ring removal)   - Offer male condoms**☼** |

* If indicated and/or per local standard of care

⌂ To be collected prior to or at the time of ring removal for participants in Group 2

**☼** Details are specified in the SSP

### Day 30 and Day 60 - Visits 6 and 9

Table 12: Day 30 and Day 60 - Visits 6 and 9

| **Day 30 and Day 60 - Visits 6 and 9** | | |
| --- | --- | --- |
| **Component** | | **Procedure/Analysis** |
| **Administrative and Regulatory** | | - - Review/update locator information   - Provide reimbursement   - Schedule next visit/contact |
| **Behavioral/Counseling** | | - - HIV pre- and post-test counseling*   - HIV/STI risk reduction counseling*   - Protocol counseling   - Contraceptive counseling   - Collect product use information |
| **Clinical** | | - - Review/update medical and menstrual history   - Review/update concomitant medications   - Perform targeted physical examination*   - Perform pelvic examination   - Perform breast examination*   - Digital examination by clinician to place or check VR   - Treat or prescribe treatment for RTI/UTI/STIs*   - Provide available test results   - Collect AEs |
| **Laboratory** | **Urine** | - - Pregnancy test   - Urine dipstick/culture* |
|  | **Blood** | - - HIV testing*   - Serum creatinine*   - CBC with differential and platelets*   - AST/ALT*   - Syphilis serology*   - Serum progesterone and estradiol   - DPV concentration ♦   - Progestogen concentrations including LNG ♦ |
|  | **Pelvic** | - - NAAT for GC/CT and trichomonas*   - Saline/KOH wet mount with pH for candidiasis and/or BV*   - Genital lesion testing for HSV*   - CVF DPV concentration ♦   - CVF LNG concentration ♦   - Cervical biopsies for DPV concentration and PD ♦ (Day 30 only) |
| **Study Product Supply** | | - - Provision of the stored study VR (Group 2 only)   - Insertion of the stored study VR by the participant or clinician/designee (Group 2 only)   - Review of study VR use instructions   - Offer male condoms**☼** |

* If indicated and/or per local standard of care

♦ To be collected prior to ring insertion for participants in Group 2

**☼** Details are specified in the SSP

### Day 44 and Day 74 - Visits 7 and 10

Table 13: Day 44 and Day 74 – Visits 7 and 10

| **Day 44 and Day 74 - Visits 7 and 10** | | |
| --- | --- | --- |
| **Component** | | **Procedure/Analysis** |
| **Administrative and Regulatory** | | - - Review/update locator information   - Provide reimbursement   - Schedule next visit/contact |
| **Behavioral/Counseling** | | - - HIV pre- and post-test counseling*   - HIV/STI risk reduction counseling*   - Protocol counseling   - Contraceptive counseling |
| **Clinical** | | - - Review/update medical and menstrual history   - Review/update concomitant medications   - Perform targeted physical examination*   - Perform pelvic examination   - Perform breast examination*   - Treat or prescribe treatment for RTI/UTI/STIs*   - Provide available test results   - Collect AEs |
| **Laboratory** | **Urine** | - - Pregnancy test*   - Urine dipstick/culture* |
|  | **Blood** | - - HIV testing*   - Serum creatinine*   - CBC with differential and platelets*   - AST/ALT*   - Syphilis serology*   - Serum progesterone and estradiol   - DPV concentration   - Progestogen concentrations including LNG |
|  | **Pelvic** | - - NAAT for GC/CT and trichomonas*   - Saline/KOH wet mount with pH for candidiasis and/or BV*   - Genital lesion testing for HSV*   - Vaginal Gram stain   - CVF DPV concentration   - CVF LNG concentration   - CVF for microbiota   - CVL for biomarkers |
| **Study Product Supply** | | - - Offer male condoms**☼** |

* If indicated and/or per local standard of care

**☼** Details are specified in the SSP

### PUEV/Early Study Termination Visit Ring Removal- Day 90 - Visit 11

The Product Use End Visit/Visit 11 will be conducted around Day 90. A visit window will be specified in the MTN-044/IPM 053/CCN019 SSP Manual (www.mtnstopshiv.org).

Table 14: PUEV/Early Study Termination Visit - Day 90 – Visit 11

| **PUEV/Early Study Termination Visit - Day 90 – Visit 11** | | |
| --- | --- | --- |
| **Component** | | **Procedure/Analysis** |
| **Administrative and Regulatory** | | - - Review/update locator information   - Provide reimbursement   - Schedule next visit/contact |
| **Behavioral/Counseling** | | - - HIV pre- and post-test counseling   - HIV/STI risk reduction counseling   - Protocol counseling   - Contraceptive counseling   - Collect product use information   - Behavioral assessment   - Behavioral in-depth interview |
| **Clinical** | | - - Review/update medical and menstrual history   - Review/update concomitant medications   - Perform physical examination   - Perform pelvic examination   - Perform breast examination   - Treat or prescribe treatment for RTI/UTI/STIs*   - Provide available test results   - Collect AEs |
| **Laboratory** | **Urine** | - - Pregnancy test   - Urine dipstick/culture* |
|  | **Blood** | - - HIV testing   - Serum creatinine   - CBC with differential and platelets   - AST/ALT   - Syphilis serology*   - Serum progesterone and estradiol   - Sex hormone-binding globulin (SHBG) and albumin   - DPV concentration ∞   - Progestogen concentrations including LNG ∞ |
|  | **Pelvic** | - - NAAT for GC/CT and trichomonas*   - Saline/KOH wet mount with pH for candidiasis and/or BV*   - Genital lesion testing for HSV*   - Vaginal Gram stain   - CVF DPV concentration ∞   - CVF LNG concentration ∞   - Cervical biopsies for DPV concentration and PD ∞ |
| **Study Product Supply** | | - - Removal and collection of study VR   - Offer male condoms**☼** |

* If indicated and/or per local standard of care

∞ To be collected immediately prior to ring removal

**☼** Details are specified in the SSP

### Day 91, Day 92 and Day 93 or 94 - Visits 12, 13 and 14

Table 15: Day 91, Day 92 and Day 93 or 94 – Visits 12, 13 and 14

| **Day 91, Day 92 and Day 93 or 94 - Visits 12, 13, and 14** | | |
| --- | --- | --- |
| **Component** | | **Procedure/Analysis** |
| **Administrative and Regulatory** | | - - Review/update locator information   - Provide reimbursement   - Schedule next visit/contact |
| **Behavioral/Counseling** | | - - HIV pre- and post-test counseling*   - HIV/STI risk reduction counseling*   - Contraceptive counseling (Visit 14 only) |
| **Clinical** | | - - Review/update medical and menstrual history   - Review/update concomitant medications   - Perform targeted physical examination*   - Perform pelvic examination*   - Perform breast examination*   - Treat or prescribe treatment for RTI/UTI/STIs*   - Provide available test results   - Collect AEs |
| **Laboratory** | **Urine** | - - Urine dipstick/culture* |
|  | **Blood** | - - HIV testing*   - Serum creatinine*   - CBC with differential and platelets*   - Syphilis serology*   - DPV concentration   - Progestogen concentrations including LNG |
|  | **Pelvic** | - - NAAT for GC/CT and trichomonas*   - Saline/KOH wet mount with pH for candidiasis and/or BV*   - Genital lesion testing for HSV*   - CVF DPV concentration   - CVF LNG concentration |
| **Study Product Supply** | | - - Provide home pregnancy test kits (Visit 14 only)   - Offer male condoms**☼** |

* If indicated and/or per local standard of care

**☼** Details are specified in the SSP

### Final Phone Calls/Contacts – 1, 4, 8 and 12 Weeks After Ring Removal – Visits 15, 16, 17 and 18

At one week (Day 7) after the ring removal, participants will be contacted by phone to provide updates on bleeding/menses, AEs, concomitant medications and other study measures. The 1-week phone call after ring removal (Visit 14) is required for all participants. Study staff will inform participants at Visit 14 that continued phone/contact at 4, 8, and 12 Weeks is indicated to ensure that:

- Any ongoing study product-related AE at study discontinuation will be followed monthly until the AE has resolved or stabilized.
- Any participant electing to use a non-hormonal contraceptive or no contraception (including those desiring pregnancy) will be followed monthly until return of menses for up to 3 months after stopping study product use. Any woman who does not spontaneously menstruate within 3 months following study product discontinuation will be referred for appropriate evaluation. The study site will follow up with the participant and/or her medical provider for a diagnosis. Notably, phone calls at 4, 8, and 12 weeks after ring removal will not be conducted for participants who reported return of menses or initiation of a hormonal contraceptive method on any preceding phone call. The end of study/final visit will be defined by the last phone call conducted for each participant (Visits 15, 16, 17 or 18) and is dependent on initiation of hormonal contraception, return to spontaneous menses or referral for evaluation of secondary amenorrhea.

Table 16: Final Phone Calls/Contacts – 1, 4, 8, and 12 Weeks After Ring Removal – Visits 15, 16, 17 and 18

| **Final Phone Calls/Contacts – 1, 4, 8, and 12 Weeks After Ring Removal – Visits 15, 16, 17 and 18** | |
| --- | --- |
| **Component** | **Procedure/Analysis** |
| **Administrative and Regulatory** | - - Review/update locator information   - Provide reimbursement¥   - Schedule next visit/contact* |
| **Behavioral/Counseling** | - - HIV pre- and post-test counseling*   - HIV/STI risk reduction counseling* |
| **Clinical** | - - Review/update medical and menstrual history   - Review/update concomitant medications (Visit 15 only)   - Refer for evaluation of secondary amenorrhea* (Visit 18 only)   - Provide available test results (Visit 15 only)   - Collect AEs (Visit 15 only)   - Review result of self-administered home pregnancy test performed on the day of phone call/contact |

¥ Per site policy specified in the SSP

* If indicated and/or per local standard of care

## Follow-up Procedures for Participants Who Permanently Discontinue Study Product

### Participants Who Become Infected with HIV

If a participant tests positive for HIV at the Enrollment Visit or at any time during study participation, she will be referred to local care and treatment services and may return to the research clinic for additional counseling and other support services, as needed. Continued study participation would be of no added benefit; thus follow-up visits will be discontinued, study product use will cease, and the participant will be considered terminated from the study. Participant may be asked to complete the procedures scheduled to occur at the PUEV/Early Study Termination Visit (Visit 11). Participants who become infected after randomization may be offered additional laboratory testing (such as HIV RNA and HIV drug resistance testing), as clinically indicated per discussions between IoR and LC. Please reference the MTN-044/IPM 053/CCN019 SSP Manual for additional details (www.mtnstopshiv.org).

### Participants Who Become Pregnant

If a participant becomes pregnant, she will be referred to local health care services and may return to the research clinic for additional counseling, as needed. Continued study participation would be of no added benefit to the participant, thus follow-up visits and procedures will be discontinued and the participant will be considered terminated from the study. The participant may be asked to complete procedures scheduled to occur at the PUEV/Early Study Termination Visit (Visit 11). A participant who is pregnant at study termination will continue to be followed until the pregnancy outcome is ascertained, see [Section 9.8](#_Pregnancy) for additional details. The study site will make every reasonable effort to contact participants and collect infant outcome information up to approximately one year after delivery for those pregnancies that result in live birth. For additional details regarding obtaining pregnancy outcome, please reference the MTN-044/IPM 053/CCN019 SSP Manual (www.mtnstopshiv.org).

### Participants Who Permanently Discontinue Study Product for Other Reasons

For participants who permanently discontinue study product use for any other clinician initiated reasons other than HIV seroconversion or pregnancy, site investigators may, after consultation with the PSRT and MTN-044/IPM 053/CCN019 Management Team, decide to discontinue study follow-up visits and procedures. Participants will, however, be asked to complete all the procedures scheduled to occur at the PUEV/Early Study Termination Visit (Visit 11), if willing.

Participants who permanently discontinue study product use due to an AE must continue to be followed in the study, if willing, until resolution (return to baseline) or stabilization of the AE is documented.

In the event study follow-up is continued, participants will have the protocol-specified visits through Final Contact. Protocol-specified procedures will continue except the following:

- - Pelvic exams*
  - Collection of blood for safety assessments*
  - Behavioral assessments related to product adherence
  - Sample collections for DPV and LNG concentrations
  - Product use data collection
  - Protocol counseling will be modified

**Unless required for AE follow-up*

The above procedures should be collected/conducted at the visit in which study product is discontinued and omitted thereafter, unless the participant was already on a temporary hold.

*Note: The MTN-044/IPM 053/CCN019 Management Team, in consultation with the MTN Pharmacology Core, may provide guidance to the site regarding a modified study visit schedule for participants who permanently discontinue study to ensure PK samples are collected at the appropriate time points and/or omitted if the collection of samples would not be anticipated to yield analyzable data. Participants’ duration of use and timing of study product permanent discontinuation will be factored into a modified schedule. Refer to the MTN-044/IPM 053/CCN019 SSP Manual for additional details.*

## Interim Visits

Interim visits may be performed at any time during the study, and any visit procedures may be conducted as indicated. All interim contacts and visits will be documented in participants' study records. If a participant misses a visit (e.g., presents to the clinic outside of the visit window), she can return for an interim visit to make up certain missed visit procedures and specimen collections. Refer to the MTN-044/IPM 053/CCN019 SSP Manual for additional details.

## Protocol Counseling: Adherence and Contraception Counseling

Study product and protocol adherence counseling will be provided to all participants upon enrollment into the study. Contraception counseling will be provided to all participants beginning at the Enrollment Visit. Counseling will be provided in accordance with standard methods, and will include reminders regarding concomitant medications and behavioral restrictions prior to and following collection of biopsies.

## Pharmacokinetics

The entire MTN-044/IPM 053/CCN019 cohort will provide plasma and CVF for PK at Visits 2-14. Cervical biopsies will be collected for DPV concentration and PD at Visits 4, 6 and 11. Specimens will be collected for vaginal microbiota and biomarkers at Visits 2, 7 and 10. Please see Table 17 for details. Detailed instructions are provided in the MTN-044/IPM 053/CCN019 SSP Manual available at http://www.mtnstopshiv.org.

Table 17: Specimen Collection Schedule

| **Study Visit** | **Specimens collected for PK & PD** | | | **Specimens collected for vaginal environment** |
| --- | --- | --- | --- | --- |
| Visit 2 - Enrollment  (Day 0) ▲ | - Blood for DPV - Blood for LNG | - CVF for DPV - CVF for LNG |  | - CVF - CVL |
| Interval #1  Visit 3 - Day 2  Visit 4 - Day 14  Visit 5 - Day 28 ⌂ | - Blood for DPV - Blood for LNG | - CVF for DPV - CVF for LNG | - Cervical tissue for DPV   and PD (Day 14 only) |  |
| Interval #2  Visit 6 - Day 30 ♦  Visit 7 - Day 44  Visit 8 - Day 58 ⌂ | - Blood for DPV - Blood for LNG | - CVF for DPV - CVF for LNG | - Cervical tissue for DPV   and PD (Day 30 only) | - CVF   (Day 44 only)   - CVL   (Day 44 only) |
| Interval #3  Visit 9 - Day 60 ♦  Visit 10 - Day 74 | - Blood for DPV - Blood for LNG | - CVF for DPV - CVF for LNG |  | - CVF   (Day 74 only)   - CVL   (Day 74 only) |
| Visit 11 - PUEV/Early Study Termination Visit (Day 90) ∞ | - Blood for DPV - Blood for LNG | - CVF for DPV - CVF for LNG | - Cervical tissue for DPV   and PD |  |
| Visit 12 - Day 91  Visit 13 - Day 92  Visit 14 - Day 93 or 94 | - Blood for DPV - Blood for LNG | - CVF for DPV - CVF for LNG |  |  |

▲ To be collected at baseline prior to ring insertion

⌂ To be collected prior to or at the time of ring removal for participants in Group 2 (Day 28 and 58)

♦ To be collected prior to ring insertion for participants in Group 2 (Day 30 and 60)

∞ To be collected immediately prior to ring removal

## Behavioral Measures

**Vaginal Bleeding and Adherence Assessment**

We will assess vaginal bleeding and product adherence via participant report. Participants will be asked to answer questions regarding their ring use and any vaginal bleeding they may experience during the study product use period. Short message service (SMS) will be employed as a measure to monitor vaginal bleeding or spotting and product adherence. Text messages will be sent daily to collect vaginal bleeding or spotting data and weekly to collect product adherence data.

**Behavioral Assessment**

The behavioral measures used in this protocol will focus on assessing participant experiences with inserting, wearing and removing the ring. At baseline, initial

acceptability of the VRs will be assessed. The baseline behavioral assessment will have questions on prior contraceptive use, use of vaginal products and practices, experience of vaginal ring use and sexual activity. Participant experiences with the trial, including acceptability of and adherence to the ring and product preference, will be assessed through a follow-up behavioral assessment at Visit 11.

**In-Depth Interview**

All participants will be invited to complete an in-depth interview (IDI) before exiting from the study. Depending on participant availability and visit length, it may be necessary to conduct this assessment as a separate visit. The IDI will address bleeding experience with study VR use and acceptability during the trial. Data on bleeding, other components of acceptability and factors affecting adherence will be collected during the IDI. These IDIs will be conducted by a trained qualitative interviewer and will follow a semi-structured questionnaire guide and are anticipated to last approximately 30-45 minutes. These IDIs may be conducted over the computer. The audio from the IDI will be recorded, summarized and transcribed for analysis. The interview notes, audio recordings and transcripts will be considered as source documentation.

## Clinical Evaluations and Procedures

**Physical examinations**

Physical exams will include the following assessments:

General appearance

Weight*

Height**

Vital signs

- - Temperature*
  - Pulse
  - Blood pressure
  - Respirations*

Neck*

Lymph nodes*

Heart*

Lungs*

Breast*

Abdomen*

Extremities*

Skin*

Neurological*

**may be omitted during targeted physical examinations*

***may be omitted after the Screening Visit*

Additional clinical assessments may be performed at the discretion of the examining clinician in response to symptoms or illnesses present at the time of the examination.

**Pelvic Examination and Specimen Collection**

Pelvic examinations will be conducted per guidelines for naked eye inspection described in the WHO/CONRAD Manual for Standardization of Colposcopy for the Evaluation of Vaginal Products, Update 2004, available at http://www.conrad.org/publications-13.html.

The required sequence of procedures and specimen collection performed during pelvic exams will be specified in the MTN-044/IPM 053/CCN019 SSP Manual.

## Laboratory Evaluations

Local Laboratory

- Urine
  - Pregnancy test
  - Dipstick/culture
- Blood
  - Serum creatinine
  - AST/ALT
  - Complete blood count with platelets and differential
  - HIV testing
  - Syphilis serology
  - Sex hormone-binding globulin (SHBG) and albumin
  - Serum progesterone and estradiol
- Pelvic
  - Pap test
  - Saline/KOH wet mount with pH for candidiasis and/or BV
  - NAAT for GC/CT and trichomonas
  - Herpes testing of genital lesions

Network LC

- Blood
  - DPV and Progestogen concentrations including LNG
  - Confirmation HIV testing for seroconversion
  - HIV resistance testing for those with confirmed seroconversion
  - Plasma for archive
- Pelvic
  - CVF for DPV and LNG concentrations
  - Cervical tissue for DPV concentration
  - Cervical tissue for PD
  - Gram stain of vaginal smear
  - CVL for biomarkers
  - CVF for microbiota

Self-Evaluation

- Urine
  - Home pregnancy test

IPM or MTN Designated Laboratory

- Study Product
  - Used study VR residual drug level assessment (DPV and LNG)

Once all required study analyses of collected specimens are complete, any remaining samples may be shipped to the MTN LC for use in study-related quality assurance and quality control testing. If study samples will be used for assay validation or proficiency testing that is not study related, all participant identifiers will be removed from the samples prior to use. Specimens obtained from participants who do not consent to long- term storage will not be used for assay validation or proficiency testing purposes.

## Specimen Management

The study site will adhere to the standards of good clinical laboratory practice in accordance with MTN-044/IPM 053/CCN019 SSP Manual (http://www.mtnstopshiv.org) and site SOPs for proper collection, processing, labeling, transport, and storage of specimens to standardize procedures. Specimen collection, testing, and storage at the site laboratories will be documented when applicable using the Laboratory Data Management System (LDMS). In cases where laboratory results are not available due to administrative or laboratory error, sites are permitted to re-draw specimens. Further, as part of quality control, researchers may need to look at short pieces of non-coding repetitive DNA sequence (3-7 base pairs) from blood in the event of sample mix-up. This test will only let researchers know the number of times this short segment is repeated and not specific genes or specific sequences of base pairs. This sequence element does not contain any information about genes, and therefore researchers will not be able to identify if participants are predisposed to specific diseases or any other genetic information based on this information. This test will be an important tool for distinguishing whether two samples collected at the same or different time points are likely from the same person. The test will only be used as part of a sample investigation with the knowledge of the site in situations where a known or suspected sample mix-up has occurred. No genetic testing (limited or genome-wide) is planned on leftover samples that are stored for the purposes of future research.

## Laboratory Oversight

All laboratories participating in this clinical trial will adhere to the DAIDS Laboratory Policy.

(https://www.niaid.nih.gov/research/daids-clinical-research-policies-us-labs)

## Biohazard Containment

As the acquisition of HIV and other blood-borne pathogens can occur through contact with contaminated needles, blood, and blood products, appropriate blood and secretion precautions will be employed by all personnel in the drawing of blood and shipping and handling of all specimens for this study as recommended by the U.S. CDC and NIH. All biological specimens will be transported using packaging mandated by US Code of Federal Regulations (CFR) 42 Part 72. All dangerous goods materials, including diagnostic specimens and infectious substances, must be transported according to instructions detailed in the International Air Transport Association (IATA) Dangerous Goods Regulations. Biohazardous waste will be contained according to institutional, transportation/carrier, and all other applicable regulations.

# ASSESSMENT OF SAFETY

## Safety Monitoring

Site IoRs/designees are responsible for continuous close safety monitoring of all study participants, and for alerting the Protocol Team if unexpected concerns arise. A sub-group of the Protocol Team, including the Protocol Co-Chairs, NICHD and DAIDS Medical Officers (MOs), Protocol Safety Physician(s), and IPM Safety Physician(s) will serve as the PSRT. The MTN SDMC prepares routine AE and clinical data reports for review by the PSRT, which meets via conference call approximately once per month or as needed throughout the period of study implementation to review safety data, discuss product use management, and address any potential safety concerns.

## Clinical Data and Safety Review

A multi-tiered safety review process will be followed for the duration of this study. The site IoR is responsible for the initial evaluation and reporting of safety information at the participant level and for alerting the PSRT if unexpected concerns arise. Participant safety is also monitored at the Network level through a series of routine reviews conducted by the SDMC, the PSRT and study sponsors. Additional reviews may be conducted at each of these levels as dictated by the occurrence of certain events.

MTN SDMC staff will review incoming safety data on an ongoing basis. Events identified as questionable, inconsistent, or unexplained will be queried for verification. AE reports submitted that meet the definition of an SAE will be reported in an expedited manner to the HD Safety Review Team. SAEs will also be included in safety summaries for the PSRT to review (see [Section 8.4.1](#_Serious_Adverse_Event) for more information).

The PSRT will meet approximately every month, or as needed, via conference call to review clinical data reports generated by the MTN SDMC. The content, format and frequency of the clinical data reports will be agreed upon by the PSRT and the SDMC in advance of study implementation. In addition to the routine safety data reviews, the PSRT will convene on an ad hoc basis to make decisions regarding the handling of any significant safety concerns. If necessary, experts external to the MTN representing expertise in the fields of microbicides, contraception, biostatistics, HIV acquisition and medical ethics may be invited to join the PSRT safety review. A recommendation to pause or stop the trial may be made by the PSRT at this time or at any such time that the team agrees that an unacceptable type and/or frequency of AEs has been observed.

The Study Monitoring Committee (SMC) will review participant safety data as part of their regular reviews (see [Section 10.8.1](#_Data_and_Safety)), since no Data and Safety Monitoring Board oversight is planned for MTN-044/IPM 053/CCN019. The SMC may recommend that the study proceed as designed, proceed with design modifications, or be discontinued. Members of the SMC will be independent investigators with no interest (financial or otherwise) in the outcomes of this study. If at any time a decision is made to discontinue enrollment and/or study product use in all participants, IPM will notify the US Food and Drug Administration (FDA) and the Site IoR will notify the responsible IRB expeditiously.

The MTN SMC will also conduct interim reviews of study progress, including rates of participant accrual, retention, completion of primary and main secondary endpoint assessments, and study or laboratory issues. These reviews will take place approximately every 4-6 months, or as needed. At the time of these reviews, or at any other time, the SMC may recommend that the study proceed as designed, proceed with design modifications, or be discontinued.

## Adverse Events Definitions and Reporting Requirements

### Adverse Events

An AE is defined as any untoward medical occurrence in a clinical research participant administered an IP and which does not necessarily have a causal relationship with the IP. As such, an AE can be an unfavorable or unintended sign (including an abnormal laboratory finding, for example), symptom or disease temporally associated with the use of an IP, whether or not considered related to the product. This definition is applied to all study groups beginning at the time of enrollment (i.e., once a participant is randomized) through Visit 15. The IP for this study refers to the matrix vaginal rings containing 200 mg DPV and 320 mg LNG.

Study participants will be provided instructions for contacting the study site to report any untoward medical occurrences they may experience. In cases of potentially life-threatening events, participants will be instructed to seek immediate emergency care. Where feasible and medically appropriate, participants will be encouraged to seek evaluation where a study clinician is based, and to request that the clinician be contacted upon their arrival. With appropriate permission of the participant, whenever possible, records from all non-study medical providers related to untoward medical occurrences will be obtained and required data elements will be captured in the study database. All participants reporting an untoward medical occurrence will be followed clinically until the occurrence resolves (returns to baseline) or stabilizes. Study site staff will document in source documents and in the study database all AEs reported by or observed in enrolled study participants regardless of severity and presumed relationship to study product. AEs will be graded per the DAIDS Table for Grading the Severity of Adult and Pediatric Adverse Events, Corrected Version 2.1, July 2017, and Addendum 1 (Female Genital Grading Table for Use in Microbicide Studies [Dated November 2007]).

In cases where a genital AE is covered in multiple tables, the Female Genital Grading Table for Use in Microbicide Studies will be the grading scale utilized.

Please note:

- Asymptomatic BV and asymptomatic candidiasis will not be reportable AEs;

*Note:* Asymptomatic BV and asymptomatic candidiasis will be captured on the STI CRF.

- Fetal losses (e.g., spontaneous abortions, ectopic pregnancy, spontaneous fetal deaths, stillbirths) will not be reported as AEs;

*Note:* Fetal loss data will be captured on the Pregnancy Outcome CRF.

- Untoward maternal conditions that either result in or result from fetal losses are reported as reproductive system AEs;
- Changes in vaginal bleeding will be collected as data, but will not be considered an AE unless it results in early discontinuation from the study or is deemed to be an SAE.

### Serious Adverse Events

SAEs will be defined as AEs occurring at any dose that:

- Result in death
- Are life-threatening
- Result in persistent or significant disability/incapacity
- Are congenital anomalies/birth defects
- Require inpatient hospitalization or prolongation of existing hospitalization

*Note:* Per International Conference on Harmonization (ICH) SAE definition, hospitalization itself is not an AE, but is an outcome of the event. Thus, hospitalization in the absence of an AE is not regarded as an AE, and is not subject to expedited reporting. The following are examples of hospitalization that are not considered to be AEs:

- - Protocol-specified admission (e.g., for procedure required by study protocol)
  - Admission for treatment of target disease of the study, or for pre-existing condition (unless it is a worsening or increase in frequency of hospital admissions as judged by the clinical investigator)
  - Diagnostic admission (e.g., for a work-up of an existing condition such as persistent pretreatment laboratory abnormality)
  - Administrative admission (e.g., for annual physical)
  - Social admission (e.g., placement for lack of place to sleep)
  - Elective admission (e.g., for elective surgery)

Important medical events that may not result in death, be life-threatening, or require hospitalization may be considered a serious adverse drug experience when, based upon appropriate medical judgment, they may jeopardize the patient or subject and may require medical or surgical intervention to prevent one of the outcomes listed above. Examples of these events would include cancer or an overdose for which an SAE would be expected to be submitted.

### Adverse Event Relationship to Study Product

Relatedness is an assessment made by a study clinician of whether or not the event is related to the study agent. The relationship categories that will be used for this study are:

- *Related:* There is a reasonable possibility that the AE may be related to the study agent(s)
- *Not Related:* There is not a reasonable possibility that the AE is related to the study agent(s)

## Adverse Event Reporting Requirements

### Serious Adverse Event Reporting

All Serious Adverse Events (SAEs) will be reported promptly in accordance with the Food and Drug Administration (FDA) policy and recorded on the appropriate forms. IPM, in conjunction with NICHD, DAIDS and MTN, is responsible for complying with the reporting requirements of SAEs to the FDA in accordance with 21 CFR 312.50. Health Decisions will serve as the third-party monitoring body of SAEs.

When any SAE, regardless of causality or relationship to the study product, is encountered during this clinical trial at an investigator’s site, the investigator, in accordance with 21 CFR 312.64(b), will notify HD’s Safety Review Team within 24 hours of identification/awareness of the SAE by reporting the event via the electronic data capture system. The investigator should complete the SAE section of the Adverse Event Log eCRF within the study Electronic Data Capture (EDC) system which will send the appropriate notifications immediately to the HD Safety Review Team, PSRT and appropriate funding agencies staff.

In the event of a system outage or other complication, notification may be made to HD at +1 919-967-1111. Immediately following phone communication, the clinical investigator is to follow up with the SAE Report Form submission by fax or email to HD at 1-919-967-1145 or nichd@healthdec.com to ensure information on the event is received within 24 hours. HD will then notify the PSRT accordingly and appropriate funding agencies staff. The SAE form is available on the HD Study Home page at <https://livetrial.healthdec.com/>. Once the system outage is resolved, submission through the EDC system will be required. Please note that site queries may also be released to ensure that any SAE Report Form information is consistent with the information submitted through the study EDC system should any SAE Report Form be submitted due to a system outage. Investigators should not wait to collect the additional information needed to fully document the event before initial submission of an SAE.

Any additional SAE supporting documentation should be submitted whenever possible (with subject identification information redacted) to verify the medical diagnosis. This includes hospital discharge summaries, laboratory report, death certificates/autopsy reports (where applicable), surgical procedure summaries, histology reports, and imaging reports. These supporting documents should be uploaded to the EDC system within 3 days of receipt at the site as an attachment to the Adverse Event Log eCRF.

IPM, in conjunction with NICHD, DAIDS and MTN will be responsible for the appropriate recording, review and compliance with regulatory reporting requirements to the FDA of the SAE, while HD will work with the site to ensure the SAE is properly recorded. The site may be queried for clarification, as needed, to ensure that all SAE information is consistent and accurate. If warranted, the PSRT will recommend if action is required to convene the Study Monitoring Committee (SMC) or if the trial should be stopped prematurely, undergo modification, or be placed on hold. HD may also recommend to the PSRT if any of these actions are warranted based on the SAE reports.

Please refer to the MTN-044/IPM 053/CCN019 SSP Manual for further details related to AE and SAE reporting. For further questions about expedited reporting, please contact your HD study project team.

### Reporting Requirements for this Study

- The SAE Reporting Category, as defined in the MTN-044/IPM 053/CCN019 SSP Manual will be used in this study
- The study agent for which expedited reporting is required is the DPV-LNG VR

### Grading Severity of Events

The grading of severity of events and the reporting period will be the same as for all AEs, as described in [Section 8.3.1](#_Adverse_Events). The most current DAIDS Table for Grading Adult and Pediatric Adverse Events, Corrected Version 2.1, July 2017 and the Female Genital Grading Table for Use in Microbicide Studies (Addendum 1 to the DAIDS Table for Grading the Severity of Adult and Pediatric Adverse Events, Version 1.0, November 2007), will be used and are available on the RSC website at http://rsc.tech-res.com/clinical-research-sites/safety-reporting/daids-grading-tables.

### Serious AE Reporting Period

The SAE reporting period for this study begins at enrollment (after randomization) and continues through completion of Visit 15.

After the protocol-defined AE reporting period, unless otherwise noted, only suspected, unexpected serious adverse reactions (SUSARs) as defined in 21 CFR 312.32(c)(1)(i) and further clarified in the FDA Guidance for Industry and Investigators Safety Reporting Requirements for INDs and BA/BE Studies (https://www.fda.gov/downloads/Drugs/Guidances/UCM227351.pdf) will be reported to the HD Safety Review Team if the study staff becomes aware of the events on a passive basis (from publicly available information).

## Pregnancy and Pregnancy Outcomes

Pregnant women are excluded from this study.

The incidence of pregnancy will be assessed. Information on pregnancy will be recorded on all subjects becoming pregnant during their study participation after enrollment through their PUEV/Early Study Termination Visit. If a subject becomes pregnant during the trial, the investigator must inform the HD Safety Review Team of the pregnancy within 24 hours of such determination via the Pregnancy Report CRF within the study EDC system, which will also send the appropriate notifications to the PSRT and appropriate funding agencies staff. Additional pregnancy data will be submitted as it becomes available.

In the event of a system outage or other complication, notification may be made to HD at +1 919-967-1111. Immediately following phone communication, the clinical investigator is to follow up with submission of the Pregnancy Report Form following the same submission guidelines outlined in [Section 8.4.1](#_Serious_Adverse_Event) for SAE submissions by fax or email to HD at 1-919-967-1145 or nichd@healthdec.com to ensure information on the event is received within 24 hours. HD will then notify the PSRT accordingly and appropriate funding agencies staff. The Pregnancy Report Form is available on the HD Study Home page at https://livetrial.healthdec.com/. Once the system outage is resolved, submission through the EDC system will be required.

The estimated date of conception will be made by the investigator based upon results of the following pregnancy determination criteria once a positive pregnancy result is received, listed in order from most accurate to least accurate:

- - Transvaginal ultrasound.
  - Quantitative β-hCG determination.
  - Estimate based on pelvic and/or abdominal examination or pregnancy outcome.
  - Clinical history and other information collected in the study (e.g., last menstrual period).
  - Investigator estimation in the absence of above criteria.

A participant who is pregnant after enrollment will continue to be followed until the pregnancy outcome is ascertained. A participant who becomes pregnant during the course of the study will have study product discontinued and will be terminated from the study (see [Section 9.8](#_Pregnancy) for additional details). Pregnancy outcomes will be reported to the HD Safety Review Team and the PSRT. The study site will make every reasonable effort to contact participants and collect infant outcome up to approximately one year after delivery for those pregnancies that result in live birth. Any outstanding data at the end of the study that is collected will be submitted to the FDA as part of an annual IND update. A clinical summary of the prenatal and postnatal events will be reported separately from the case report form in narrative format in the clinical summary report.

## Regulatory Requirements

Information on all reported AEs will be included in reports to the FDA and other applicable government and regulatory authorities. Site IoR/designee will submit AEs and any relevant safety information in accordance with local regulatory requirements.

## Social Harms Reporting

Although study sites make every effort to protect participant privacy and confidentiality, it is possible that participants’ involvement in the study could become known to others and that social harms may result. Social harms that are determined by the IoR/designee to be serious or unexpected will be reported to the PSRT and responsible site IRBs according to their individual requirements.

# CLINICAL MANAGEMENT

Guidelines for clinical management and temporary product hold/permanent discontinuation of study product are outlined in this section. In general, the IoR/designee has the discretion to hold study product use at any time if s/he feels that continued product use would be harmful to the participant or interfere with treatment deemed clinically necessary. Unless otherwise specified below, the IoR/designee must immediately consult the PSRT for further guidance on resuming study product, continuing the hold temporarily, or progressing to permanent discontinuation of study product. The IoR/designee will document all temporary product holds and permanent discontinuations on applicable CRFs.

Participants reporting any unresolved AEs at the time of study termination will be followed clinically until the occurrence resolves (returns to baseline) or stabilizes, and this information will be documented.

## Grading System

AE severity grading is described in [Section 8.4.3](#_Grading_Severity_of).

## Dose Modification Instructions

No dose modifications will be undertaken in this study.

## General Criteria for Temporary Hold and Permanent Discontinuation of Study Product

A participant will be permanently discontinued from the DPV-LNG VR product use by the IoR/designee for any of the following reasons:

- Acquisition of HIV infection; such participants will not resume product use at any time. The study VR must be discontinued immediately upon recognition of the first reactive rapid HIV test.
- Allergic reaction to the VR
- Pregnancy
- Breastfeeding
- Non-therapeutic injection drug use
- Reported use of PEP for HIV exposure
- Reported use of PrEP for HIV prevention
- Participant is unable or unwilling to comply with required study procedures, or otherwise might be put at undue risk to their safety and well-being by continuing product use (e.g., changes in safety laboratories between Screening and Enrollment) according to the judgment of the IoR/designee.

A participant will be put on temporary hold from DPV-LNG VR product use by the IoR/designee for any of the following reasons:

- Participant reports current or expected continued use of prohibited medications during study participation as listed in [Section 6.8](#_Prohibited_Medications) and further clarified in the MTN-044/IPM 053/CCN019 SSP Manual.

The loR/designee must consult the PSRT once the temporary hold is initiated. Together,

the loR/designee and the PSRT will discuss resuming product use, continuing the temporary hold, or progressing to permanent discontinuation. Please refer to the MTN-044/IPM 053/CCN019 SSP Manual for further details related to temporary product hold, resuming product use, and permanent product discontinuation.

## Response to Adverse Events

**Grade 1 or 2**

In general, a participant who develops a Grade 1 or 2 AE not specifically addressed in Section 9, regardless of relationship to study product, may continue product use.

**Grade 3**

Participants who develop a Grade 3 AE not specifically addressed in Section 9, judged by the IoR/designee to be not related to study product, may continue product use.

If a participant develops a Grade 3 AE not specifically addressed in Section 9 and the AE is judged by the IoR/designee to be related to study product, the IoR/designee must put the participant on temporary hold from product use and consult with the PSRT for further decision on product use.

**Grade 4**

Participants who develop a Grade 4 AE (regardless of relationship to study product) not specifically addressed in Section 9, must have the study product held. The IoR/designee must consult the PSRT and continue the temporary product hold until a recommendation is obtained from the PSRT.

## Sexually Transmitted Infection/Reproductive Tract Infection

The IoR/designee must manage STI/RTI per current CDC guidelines, available at http://www.cdc.gov/std/treatment/.

VR use need not be held in the event of an STI/RTI requiring treatment, unless other temporary product hold/permanent discontinuation guidelines described below apply. Should the IoR/designee determine that a temporary product hold is warranted due to an STI or RTI, consultation with the PSRT is required.

## Management of Specific Genital Events

If a suspected finding is reported by the participant between scheduled visits, an interim visit may be scheduled at the discretion of the site investigator. Management of genital events observed at scheduled or interim visits will be in accordance with the following:

**Superficial epithelial disruption or localized erythema or edema: area of less than 50% of vulvar surface or combined vaginal and cervical surface**

- Continue study VR use (at study clinician’s discretion)
- Perform naked eye evaluation
- Re-evaluate by speculum examination in approximately 3-5 days
- If condition worsens or does not resolve at that time, permanently discontinue study VR use

**Deep epithelial disruption (ulceration) or generalized erythema or severe edema: area of more than 50% of vulvar surface or combined vaginal and cervical surface affected by erythema or severe edema**

- Temporarily hold study IVR
- Perform naked eye evaluation
- Re-evaluate in approximately 3-5 days and resume study IVR use if resolved
- If unresolved at reevaluation, continue temporary product hold, and re-evaluate within approximately 2-3 days. If resolved at that time may resume use. If unresolved at this second re-evaluation, continue temporary product hold, consult with PSRT regarding permanent discontinuation, and provide care per local standard
- If there is reoccurrence with no identified etiology, continue temporary product hold and consult PSRT regarding permanent discontinuation

**Unexpected** **genital bleeding**

- Continue study VR use (at study clinician’s discretion)
- Perform naked eye evaluation
- If determined to be due to deep epithelial disruption, refer to guidelines above; otherwise continue study VR use

**Genital petechia(e) and genital ecchymosis**

- Continue study VR use (at study clinician’s discretion)
- Perform naked eye evaluation
- No further evaluation or treatment is required

## HIV Infection

Participants who test positive for HIV must have study product permanently discontinued by the IoR/designee. A participant who is confirmed to be HIV positive during the course of the study will have study product discontinued, all follow-up visits will be discontinued and the participant will be considered terminated from the study, as per [Section 7.5.1](#_Participants_Who_Become). Guidance regarding management and referral for participants confirmed to be HIV-positive is located in [Section 13.10.1](#_Care_for_Participants).

## Pregnancy

Pregnancy testing will be performed at designated study visits and participants will be encouraged to report all signs or symptoms of pregnancy to study staff. The IoR/designee will counsel any participant who becomes pregnant regarding possible risks to the fetus according to site SOPs. The IoR/designee also will refer the participant to all applicable services; however, sites will not be responsible for paying for pregnancy-related care.

A participant who becomes pregnant during the course of the study will have study product discontinued and will be terminated from the study, as per [Section 7.5.2](#_Participants_Who_Become_1). A participant who is pregnant at study termination will continue to be followed until the pregnancy outcome is ascertained (or, in consultation with the PSRT, it is determined that the pregnancy outcome cannot be ascertained). Pregnancy outcomes will be reported on relevant CRFs; outcomes meeting criteria for SAE reporting also will be reported on the Adverse Event Log eCRF. The study site will make every reasonable effort to contact participants and collect infant outcome up to approximately one year after delivery for those pregnancies that result in live birth.

## Criteria for Early Termination of Study Participation

Participants may voluntarily withdraw from the study for any reason at any time. The IoR/designee also may withdraw participants from the study to protect their safety and/or if they are unwilling or unable to comply with required study procedures, after consultation with the PSRT. Participants also may be withdrawn if IPM, NICHD, MTN, CCTN, government or regulatory authorities, including the FDA and Office for Human Research Protections (OHRP), or site IRBs terminate the study prior to its planned end date. Every reasonable effort is made to complete a final evaluation of participants who withdraw or are withdrawn from the study prior to completing follow-up. Study staff members will record the reason(s) for all withdrawals in participants’ study records.

# STATISTICAL CONSIDERATIONS

## Overview and Summary of Design

MTN-044/IPM 053/CCN019 is a Phase 1, two-arm, open-label, single-site, randomized trial of one silicone elastomer matrix VR containing a combination of the active ingredients of 200 mg DPV and 320 mg of LNG. Healthy, HIV-uninfected women aged 18-45 (inclusive) will use the DPV/LNG VR either continuously or cyclically (worn continuously for approximately 28 days and taken out for 2 days) for approximately 90 days. Randomized participants will know their group assignment to continuous use or cyclic use of the VR. Further details regarding the statistical methodology will be described in the statistical analysis plan.

## Primary Endpoints

Consistent with the primary study objective to characterize the local and systemic pharmacokinetics (PK) of one DPV-LNG vaginal ring formulation used either continuously or cyclically (~28/2) for approximately 90 days, the following endpoints will be assessed:

- DPV and LNG concentrations in plasma
- DPV and LNG concentrations in cervicovaginal fluid
- DPV concentration in cervical tissue

Consistent with the secondary study objective to evaluate the safety of one DPV-LNG vaginal ring formulation used either continuously or cyclically (~28/2) for approximately 90 days, the proportion of women with the following will be assessed:

- Grade 2 or higher genitourinary adverse events as defined by the Division of AIDS (DAIDS) Table for Grading the Severity of Adult and Pediatric Adverse Events, Corrected Version 2.1, July 2017, and/or Addendum 1 (Female Genital Grading Table for Use in Microbicide Studies [Dated November 2007])
- Grade 3 or higher adverse events as defined by the Division of AIDS (DAIDS) Table for Grading the Severity of Adult and Pediatric Adverse Events, Corrected Version 2.1, July 2017

## Study Hypotheses

- DPV and LNG concentrations in blood and CVF, and DPV concentration in cervical tissue will be measurable in all participants at all time points
- DPV concentrations in blood, CVF and cervical tissue will not be significantly lower following 2 days of ring removal in the cyclic ring users compared to the continuous ring users
- Both continuous and cyclic exposure to DPV/LNG VR containing 200 mg DPV + 320 mg LNG VR for approximately 90 days will be safe

## Sample Size and Power Calculations

### Primary Endpoints

The proposed total sample size is approximately N=24 women randomized into 2 arms in a 1:1 ratio giving 12 women per group. This sample size is based upon the size of similar Phase 1 studies of vaginal microbicide products.

As a means to characterize the statistical properties of this study Table 18 below presents the probability of observing ten or more, eleven or more, or twelve women with detectable PK levels among the 12 women in each arm given a true event rate. For example, if the true rate of detection among women using a ring is 99% then the probability we will see 11 or more women with detectable PK levels is 99%.

Table 18: Analysis of PK Event Frequency

| “True” Event Rate (PK Detectable) | P (>10 events \| n=12) | P (>11 events \| n=12) | P (12 events \| n=12) |
| --- | --- | --- | --- |
| 75% | 0.39 | 0.16 | 0.03 |
| 90% | 0.89 | 0.66 | 0.28 |
| 99% | 1.00 | 0.99 | 0.89 |

An alternative way of describing the statistical properties of the study design is in terms of the 95% confidence interval (95% CI) for the true rate based on the observed data. Table 19 below shows the exact 2-sided 95% CIs for the probability of an event based on a particular observed rate. For example, if all of the 12 participants in an arm have detectable PK, the 95% exact 2-sided lower confidence bound for the true rate of PK detection is 74%.

Table 19: Precision of Exact 2-sided 95% CIs for Observed Event Rates

| Observed Event Rate | Exact 2-sided 95% CI (n=12) |
| --- | --- |
| 12/12 (100%) | 74%, 100% |
| 10/12 (83%) | 52%, 98% |
| 8/12 (67%) | 35%, 90% |
| 6/12 (50%) | 21%, 79% |
| 4/12 (33%) | 10%, 65% |
| 2/12 (17%) | 2%, 48% |
| 0/12 (0%) | 0%, 26% |

For the primary endpoint comparing DPV concentrations in blood, CVF and cervical tissue in continuous users and cyclic users two days after removal of the ring, we use data from the MTN-030/IPM 041 study where DPV drug concentrations in blood plasma at Day 14, just prior to ring removal, and at Day 16, two days after ring removal, yielded a coefficient of variation (CV) of approximately 0.4 at each time point. Since comparisons in this study will be made between parallel groups instead of based on within-participant differences at two time points as in MTN-030/IPM 041, departures from the assumption of CV=0.4 are also explored.

Based on a two-sample t-test, α=0.05, multiple possible values for the CV, and a sample size of 12 participants in each group,

Table 20 below summarizes the power available to detect various absolute differences in drug concentration levels between continuous ring users and, following 2 days of ring removal, in cyclic ring users (i.e. at study visit days 30 and 60).

So, assuming each group has a CV of 0.5 for drug concentrations at study visits corresponding to days 30 and 60, this study has approximately 83% power to detect at least a 300 pg/mL difference in drug concentrations between the two use regimens.

Table 20: Power for Minimum Detectable Differences in Drug Concentrations for Parallel Group Comparisons of Continuous Ring Users and Two Days Following Ring Removal for Cyclic Ring Users

| Minimum detectable difference in drug concentrations (pg/mL) | Coefficient of Variation (CV) | | |
| --- | --- | --- | --- |
|  | 0.4 | 0.5 | 0.6 |
| 100 | 0.255 | 0.163 | 0.127 |
| 200 | 0.737 | 0.491 | 0.365 |
| 300 | 0.973 | 0.827 | 0.677 |
| 400 | 0.999 | 0.972 | 0.896 |
| 500 | >0.999 | 0.998 | 0.980 |
| 600 | >0.999 | >0.999 | 0.998 |

### Secondary Endpoint

Table 21 below presents the probability of observing zero, one or more and two or more safety events among the 12 women in each arm given a true event rate. For example, if the true rate of a safety event among women using a ring is 15% then the probability we will see 1 or more women with this event is 86%.

Table 21: Analysis of Safety Event Frequency

| “True” Event Rate (Safety Event) | P (0 events \| n=12) | P (>1 events \| n=12) | P (>2 events \| n=12) |
| --- | --- | --- | --- |
| 1% | 0.89 | 0.11 | <0.01 |
| 5% | 0.54 | 0.46 | 0.12 |
| 15% | 0.14 | 0.86 | 0.56 |

An alternative way of describing the statistical properties of the study design is in terms of the 95% confidence interval (95% CI) for the true rate based on the observed data. Table 19 above shows the exact 2-sided 95% CIs for the probability of an event based on a particular observed rate. If none of the 12 participants in an arm experience a safety event, the 95% exact 2-sided upper confidence bound for the true rate of such events in a particular arm of the study is 26%.

## Participant Accrual, Follow-up and Retention

Based on previous studies of vaginal products with similar eligibility requirements, the accrual of 24 eligible participants will take approximately 6 months. Each participant will be followed for approximately up to 97 consecutive days. The site will target retention of 95% of enrolled participants over the study period.

## Randomization

Participants will be randomized in a 1:1 ratio to the two arms of the study. The randomization scheme will be generated and maintained by the MTN SDMC.

## Blinding

Study staff and participants will be unblinded to the treatment regimen assignments.

## Data and Safety Monitoring and Analysis

### Study Monitoring Committee

Data and Safety Monitoring Board oversight is not planned for this study. The MTN SMC will conduct interim reviews of study progress, including rates of participant accrual, retention, completion of primary and main secondary endpoint assessments, study or laboratory issues, and a closed safety data report to voting SMC members. These reviews will take place approximately every 4-6 months, or as needed. At the time of this review, or at any other time, the SMC may recommend that the study proceed as designed, proceed with design modifications, or be discontinued. For further information regarding the SMC, please reference the MTN Manual of Operational Procedures ([www.mtnstopshiv.org](http://www.mtnstopshiv.org)).

### Primary Analyses

When the use of descriptive statistics to assess group characteristics or differences is required, the following methods will be used: for categorical variables, the number and percent in each category; for continuous variables, the mean, median, standard deviation, quartiles and range (minimum, maximum). Within-treatment group assessment of the change from the baseline measurement to a follow-up measurement will be analyzed using McNemar’s test (for categorical response variables) or the paired t-test or Wilcoxon signed-ranks test (for continuous variables).

To assess the adequacy of the randomization, participants in each of the two treatment regimens will be compared for baseline characteristics including demographics and laboratory measurements using descriptive statistics. Due to the small sample size, formal comparisons will not be done.

The proportion of women with detectable drug levels in each arm and the measured drug concentration levels will be summarized using descriptive statistics and graphics.

A two-sample t-test or Wilcoxon-Mann-Whitney test will be used to compare the mean DPV concentrations in blood, CVF, and cervical tissue among women in the two arms at study visit days 30 and 60 as applicable.

### Secondary Analyses

Safety Endpoints

All visits in which participants have been exposed to the study products will be included in the analysis of safety. Secondary intent to treat analyses may also be performed. To assess genitourinary safety, the number and percentages of participants experiencing each safety endpoint (see [Section 10.2](#_Primary_Endpoints)) will be tabulated by study arm as well as the total number of safety endpoints experienced in each arm. Each participant will contribute once in each category (i.e., only for the highest severity AE for each participant) for the calculation of event rates. Exact binomial confidence intervals will be calculated for each safety endpoint for each arm.

### Missing Data

In any situation with missing data, appropriate secondary analyses will be performed to adjust for variables that may be related to the missingness mechanism. If missing data rates are higher than 10%, covariates that are related to missingness in likelihood-based regression models will be included. Sensitivity analyses to assess the potential impact of the missing data will also be performed. These analyses will include imputing the data under the most extreme scenarios of information missingness, such as assuming everyone missing has an extreme value of the missing variable, and less informative imputation approaches.

# DATA HANDLING AND RECORDKEEPING

## Data Management Responsibilities

Data collection tools will be developed by the MTN SDMC in conjunction with the protocol team. Quality control and data integrity are managed manually and systematically with reports and queries routinely generated and provided by the SDMC to the study sites for verification and resolution. As part of the study activation process, the study site will identify all CRFs to be used as source documents. Study CRF data will be entered into the MTN-044/IPM 053/CCN019 database, transferred in compliance with the US-EU Safe Harbor Requirements and the EU Data Protection Directive 95/46/EC to the MTN SDMC, entered, and cleaned using Medidata Rave, a data management system compliant with the International Council on Harmonization (ICH) Good Clinical Practices (GCP) and US CFR guidelines for electronic data capture.

Transcriptions of interviews will be generated in the field and electronically transferred to RTI using a secure File Transfer Protocol site, where they will be uploaded and managed using a qualitative software package. RTI will act as a hub, and manage all qualitative data for the study. A convention for file naming will be developed, and all data will be labeled according to this process. RTI will save all versions of all files on a secure, password-protected server.

## Source Documents and Access to Source Data/Documents

The study site will maintain source data/documents in accordance with 21 CFR 312.57 (c) and further clarified in FDA Guidance for Industry E6 Good Clinical Practice: Consolidated Guidance located at https://clinicalcenter.nih.gov/ccc/clinicalresearch/guidance.pdf. (Refer to guidance sections 1.51 Source Data, 1.52 Source Documents, and 4.9 Records and Reports)

The IoR/designee will maintain, and store securely, complete, accurate and current study records throughout the study. In accordance with U.S. regulations regarding testing IPs, the IoR/designee will maintain all study documentation for at least two years following the date of marketing approval for the study product being tested for the indication in which they were studied. If no marketing application is filed, or if the application is not approved, the records will be retained for two years after the investigation is discontinued and the US FDA is notified.

Study records must be maintained on site for the entire period of study implementation. Thereafter, instructions for record storage will be provided by NICHD. No study records may be moved to an off-site location or destroyed prior to receiving approval from NICHD. NICHD should be notified before any transfer of oversight of the records if there is PI change at the site.

## Quality Control and Quality Assurance

The study site will conduct quality control and quality assurance procedures in accordance with internally developed SOPs, GCP and all applicable regulatory requirements.

# CLINICAL SITE MONITORING

Study monitoring will be carried out by Health Decisions (HD) (Durham, NC) in accordance with FDA GCP and ICH guidelines. HD is the Statistical and Clinical Coordinating Center for the NICHD CCTN.

The IoR/designee will allow representatives of HD direct access to inspect study facilities, including all eCRFs, source documents, and corresponding portions of medical records for each subject, observe the performance of study procedures at mutually convenient times, visit any clinical pharmacy, and review biological specimen collection and patient evaluation areas for periodic review before, during and after the study has been completed. The monitoring visits should provide HD with the opportunity to:

- Initiate the research center
- Evaluate the progress of the study and ensure timely follow-up of study conduct issues identified during monitoring visits
- Review informed consent forms (ICF), consent procedures, and documentation
- Verify the accuracy and completeness of the eCRFs
- Ensure that all protocol requirements, GCP guidelines, applicable FDA regulations and investigators’ obligations are being fulfilled
- Resolve any inconsistencies in the study records including investigational product reconciliation
- Close out the trial at the research center

In addition to the routine monitoring, the IoR/designee will also allow HD, NICHD, MTN LOC, SDMC, LC, IPM, NIAID, FDA, OHRP, IRB representatives or other local, US or international regulatory authorities at their discretion, to perform site audits. The purpose of such audits will be to evaluate site trial conduct and compliance with the protocol, SOPs, GCP, and the applicable regulatory requirements. If an audit is performed, a site must provide the auditors with direct access to all relevant records and documentation related to the study. A site visit log will be maintained at the study site to document all visits.

# HUMAN SUBJECTS PROTECTIONS

Site investigators will make every effort to minimize risks to participants. Participants and study staff members will take part in a thorough informed consent process. Before beginning the study, the IoR/designee will have obtained IRB approval and the protocol will have been submitted to the FDA. The IoR/designee will permit audits by the NIH, IPM, the FDA, OHRP, MTN LOC, IRBs, SDMC, and other local, US or international regulatory authorities, or any of their appointed agents.

## Institutional Review Boards/Ethics Committees

The participating institution is responsible for assuring that this protocol, the associated site-specific ICFs, and study-related documents (such as participant education and recruitment materials) are reviewed by an IRB responsible for oversight of research conducted at the study sites. For this study, HD will use Chesapeake IRB for oversight. As a single-site study, local IRB approval is also required for oversight of research conducted at the clinical site. Any amendments to the protocol must be approved by the responsible IRBs prior to implementation.

Subsequent to the initial review and approval, the responsible IRBs must review the study at least annually. The IoR/designee will make safety and progress reports to the IRBs at least annually and within three months after study termination or completion. These reports will include the total number of participants enrolled in the study, the number of participants who completed the study, all changes in the research activity, and all unanticipated problems involving risks to human subjects or others. In addition, the results of all SMC reviews of the study will be provided to the IRBs. The study site will submit documentation of continuing review from its local IRB to HD for the study master files.

## Protocol Registration

Prior to implementation of this protocol, and any subsequent full version amendments, the site must have the protocol and the protocol ICF(s) approved, as appropriate, by their local IRB/ethics committee (EC) and any other applicable regulatory entity (RE). The study team will register this trial on ClinicalTrials.gov, a registry database of clinical studies of human participants conducted around the world, and updated at regular, specified intervals.

In addition, HD will submit the final protocol and protocol ICF(s) to Chesapeake IRB which will review and approve the study for safety and scientific review and provide oversight of HD study operations for this single site study.

Any change of the clinical trial must be written and filed as an amendment to this protocol to the HD and local site IRBs. Such amendments will be made jointly by the funding agencies, Protocol Chairs and faculty statistician. The IoR must submit the protocol amendment for review by the local IRB and shall obtain the approval of the local IRB before it is implemented.

In cases of emergency, when the protocol change or deviation is to eliminate or reduce an immediate hazard or risk to human subjects, the amendment may be implemented before review or approval by the local IRB. In such cases, the IoR shall notify the local IRB of the change or deviation in writing within 10 working days after implementation. Any protocol-related issues that pose an immediate or significant hazard to participants must be reported to HD Safety Review Team who will immediately report the issue to the PSRT. If the protocol amendment is an administrative change, it will be sent to IRBs for information (updating of file).

All modifications of the clinical trial will be written and filed with the FDA as an amendment to this protocol, maintaining original section identification. Such modifications will be made jointly by the NICHD, MTN, HD, IPM and all the investigators with the approval of all of the IRBs.

## Study Coordination

IPM holds the Investigational New Drug (IND) application for this study. Assignment of all sponsor responsibilities for this study will be specified in a Clinical Trial Agreement (CTA) executed by NICHD and IPM.

Study implementation will be directed by this protocol, which may not be amended without proper regulatory approvals. Study implementation will also be guided by a common SSP manual that provides further instructions and operational guidance on conducting study visits; data and forms processing; specimen collection, processing, and shipping; AE assessment, management and reporting; dispensing study products and documenting product accountability; and other study operations. Standardized study-specific training will be provided to the study site by the MTN LOC, HD, SDMC, LC and other designated members of the Protocol Team.

Close coordination between protocol team members is necessary to track study progress, respond to queries about proper study implementation, and address other issues in a timely manner. The PSRT will address issues related to study eligibility and AE management and reporting as needed to assure consistent case management, documentation, and information-sharing at the site. Rates of accrual, adherence, follow-up, and AE incidence will be monitored closely by the team as well as the SMC.

## Risk Benefit Statement

### Risks

It is not expected that this trial will expose human subjects to unreasonable risk.

Vaginal Fluid Collection

Collection of vaginal fluid may cause discomfort or pressure in the vagina or genital area.

Pelvic Examination and Procedures

Pelvic examination and procedures may cause mild discomfort, pressure and/or vaginal bleeding or spotting.

Cervical Biopsy Collection

Cervical biopsies carry the risk of discomfort or pain during the procedure and for a few hours afterwards. Participants may have mild vaginal spotting (bleeding) for one or two days. Participants will be instructed not to use aspirin (over 81 mg per day) and/or other drugs that are associated with the increased likelihood of bleeding for 72 hours before and after the collection of the cervical biopsies. If participants engage in sexual intercourse before the biopsy has healed they may experience some temporary discomfort. If participants are sexually active they may also be at increased risk for STIs and HIV acquisition, if exposed. There is a small risk of infection and heavier bleeding. Participants will be instructed to contact the clinic if symptoms are bothersome, if heavy bleeding is noted (soaking through a pad in an hour or less) or if the participant develops any abnormal odor or discharge from the vagina.

Phlebotomy

Phlebotomy may lead to discomfort, feelings of dizziness or faintness, and/or bruising, swelling, having a blood clot and/or infection.

Other Risks

Being tested for HIV and STIs and waiting for your test results may cause worry, sadness or depression. Provision of positive test results has been associated with depression, suicidal ideation, and denial as well as social isolation. Trained counselors will be available to help participants deal with these feelings.

Participation in clinical research includes the risks of loss of confidentiality and discomfort with the personal nature of questions when discussing sexual behaviors and alcohol and drug use.

Participants at sites where local regulatory authorities require partner notification in response to diagnosed STI or HIV infection could have problems in participants’ relationships. Participants also could have problems in their partner relationships associated with use of study product and abstinence requirements.

Participants may be asked questions about their study product use, vaginal and sexual practices, menstrual hygiene, and alcohol and drug use. These questions may make some participants uncomfortable.

Although study sites make every effort to protect participant privacy and confidentiality, it is possible that participants' involvement in the study could become known to others, and that social harms may result (i.e., because others may think participants are HIV-positive or at "high risk" for HIV infection). For example, participants could be treated unfairly or discriminated against, or could have problems being accepted by their families, communities, and/or employer(s).

Risks Associated With Vaginal Ring

Use of the study VR may lead to vaginal symptoms, including irritation, increased discharge, and discomfort (including with vaginal intercourse if it were to occur). It is possible that a participant may have an allergic reaction to the study product. Symptoms of an allergic reaction include rash or other skin irritation, itching, joint pain, or difficulty in breathing.

Risks Associated With DPV VR

Based on pooled Phase III data analysis of the DPV VR (ASPIRE [MTN-020] and The Ring Study [IPM 027]), the most commonly reported TEAEs (occurring in ≥ 10% of participants in the DPV Ring-004 group) {(IPM), 2016 #528;(IPM), 2016 #528}are listed below:^45^

- Metrorrhagia (irregular uterine bleeding)
- Chlamydia (STI)
- Urinary tract infection
- Menorrhagia (heavy or prolonged menstrual bleeding)
- Gonorrhea (STI)
- Upper respiratory tract infection
- Vaginal yeast infection
- Female genital infection
- Bacterial vaginosis (vaginal dysbiosis)

It should be noted that a causal association between the above side effects and the study products has not yet been determined. There were no significant differences between the DPV VR and placebo groups in frequency of safety endpoints.

Based on in vitro data, HIV-infected participants who have prolonged exposure to low concentrations of DPV by continuing to use the ring after infection may have a risk of selecting viruses carrying NNRTI resistance-associated mutations. Clinical relevance has yet to be established.

Risks Associated With LNG

The most common adverse reactions reported in clinical trials of Plan B® were (>10%):

- Heavier menstrual bleeding
- Nausea
- Lower abdominal pain
- Fatigue
- Headache
- Dizziness

The most common adverse reactions reported in clinical trials of an LNG-releasing subcutaneous implant system (Jadelle®) include (>10%):

- Headache
- Nervousness
- Dizziness
- Nausea
- Changes in menstrual bleeding
- Cervicitis
- Vaginal discharge
- Genital pruritus
- Pelvic pain
- Breast pain
- Weight increase
- Vaginal yeast infection
- Acne
- Bleeding at implant insertion site (not applicable to vaginal rings)

The most common adverse reactions reported in clinical trials of another LNG-releasing subcutaneous implant system (Norplant®) include (>10%):

- Prolonged, frequent, or irregular bleeding
- Lack of vaginal bleeding (amenorrhea)
- Infrequent bleeding or spotting

The most common adverse reactions (in >5% users) for Mirena®, Jadelle® and Plan B® are similar and include:

- uterine/vaginal bleeding alterations (including amenorrhea, menorrhagia and intermenstrual bleeding)
- abdominal/pelvic pain
- headache/migraine
- acne
- depressed/altered mood
- breast tenderness/pain
- vaginal discharge
- nausea

Other rare, and potentially more serious, AEs associated with continued LNG use that have been reported are ectopic pregnancy, ovarian cysts, thrombosis, and idiopathic intracranial hypertension (particularly in obese participants).

Toxic shock syndrome has been reported with currently marketed contraceptive VRs, though a causal relationship between the two has not been established.^46^ As with any vaginally retained product, the possibility of toxic shock syndrome, although rare, exists. Detailed information regarding the plan for diagnosis and management of this condition should it arise is provided in the MTN-044/IPM 053/CCN019 SSP Manual.

Risks Associated With DPV-LNG VR

Based on preliminary analysis of MTN-030/IPM 041, reported AEs that are possibly related to the DPV-LNG VR include:

- Abdominal distension
- Nausea
- Increased appetite
- Headache
- Breast tenderness
- Uterine cramping
- Vaginal discharge
- Vaginal odor
- Vulvovaginal discomfort
- Hot flush

It should be noted that a causal association between the above side effects and the study products has not yet been determined. Data analysis is ongoing.

### Benefits

Participants will receive HIV/STI risk reduction counseling, HIV and STI testing, physical examination, pelvic examination, and routine laboratory testing. Participants will be provided with STI treatment in accordance with CDC guidelines. For other medical conditions identified as part of the study screening and/or follow-up procedures, participants will be referred to other sources of care available in their community. Some participants may have the opportunity to access expedient treatment and as a result may have decreased morbidity due to early diagnosis and treatment of abnormalities identified during tests, examinations and referrals.

Participants and others may benefit in the future from information learned from this study. Specifically, information learned in this study may lead to the development of safe and effective interventions to prevent HIV transmission and unplanned pregnancy. Information learned in this study may also help to understand issues important for broader implementation of the DPV ring. Participants may also appreciate the opportunity to contribute to the field of HIV prevention research.

## Informed Consent Process

Written informed consent will be obtained from each study participant prior to screening. Written informed consent also will be obtained for long-term specimen storage and possible future testing. Consent for long-term specimen storage is not required for study participation. In obtaining and documenting informed consent, the IoR and his/her designees will comply with applicable local and US regulatory requirements and will adhere to GCP and to the ethical principles that have their origin in the Declaration of Helsinki. Study staff must document the informed consent process in accordance with the Requirements for Source Documentation in the MTN-044/IPM 053/CCN019 SSP Manual. Participants will be provided with copies of the ICFs.

In addition to ICFs, the Protocol Team will work with study staff and community representatives to develop appropriate materials about the study and a standardized approach to the informed consent process to be implemented at the study site, which will be detailed in the SSP manual.

The informed consent process will cover all elements of informed consent required by research regulations. In addition, the process specifically will address the following topics of importance to this study:

- The unknown safety and unproven efficacy of the DPV-LNG VR
- Randomization and the importance of participants in both study groups to the success of the study
- The importance of adherence to the study visit and procedures schedule
- The potential medical risks of study participation (and what to do if such risks are experienced)
- The potential social harms associated with study participation (and what to do if such harms are experienced)
- The real yet limited benefits of study participation
- The distinction between research and clinical care
- The right to withdraw from the study at any time

## Participant Confidentiality

All study procedures will be conducted in private, and every effort will be made to protect participant privacy and confidentiality to the extent possible. Each study site will implement confidentiality protections that reflect the local study implementation plan and the input of study staff and community representatives to identify potential confidentiality issues and strategies to address them. In addition to local considerations, the protections described below will be implemented at all sites.

All study-related information will be stored securely. All participant information will be stored in locked areas with access limited to study staff. All laboratory specimens, study data collection, and administrative forms will be identified by coded number only to maintain participant confidentiality. All local databases will be secured with password protected access systems. Forms, lists, logbooks, appointment books, and any other listings that link participants’ ID numbers to identifying information will be stored in a separate, locked file in an area with limited access. After receiving appropriate approval, all study documents/data will be properly disposed of, including the proper destruction and/or deletion of paper files, electronic study data, and electronic documents. Participants’ study information will not be released without their written permission, except as necessary for review, monitoring, and/or auditing by the following:

- Representatives of the US Federal Government, including the US FDA, OHRP, NIH, and/or contractors of the NIH, and other local, US, or international regulatory authorities
- Representatives of IPM
- Representatives of the MTN LOC, SDMC, and/or LC, CCTN and HD
- Study staff
- Site IRBs

MTN has obtained a Certificate of Confidentiality from the US Department of Health and Human Services (HHS) that is applicable for this study. This Certificate protects study staff from being compelled to disclose study-related information by any US Federal, State or local civil, criminal, administrative, legislative or other proceedings. It thus serves to protect the identity and privacy of study participants.

## Special Populations

### Pregnant Women

Women who test positive for pregnancy at the Screening or Enrollment Visit will not be eligible to participate in this study. Should a participant test positive for pregnancy after Enrollment, product discontinuation will be implemented. Follow-up will be completed and data collected per [Section 7.5.2](#_Participants_Who_Become_1). During the informed consent process, participants will be informed that the study VR is not proven to be an effective method of contraception and the effects of the study VR on a developing human fetus are unknown.

### Children

The NIH has mandated that children be included in research trials when appropriate. This study meets “Justifications for Exclusion” criteria for younger children as set forth by the NIH. Specifically, “insufficient data are available in adults to judge potential risk in children” and “children should not be the initial group to be involved in research studies.” This study does not plan to enroll children under 18 years old.

## Compensation

Pending IRB approval, participants will be compensated for their time and effort in this

study, and/or be reimbursed for travel to study visits. Site specific compensation amounts will be specified in the study ICFs.

If a participant becomes ill or injured as a result of participation in this trial, medical treatment for the adverse reaction or injury will be provided appropriately. The site staff will refer the participant for ongoing treatment for the injury, if needed. IPM will be responsible for compensation for appropriate medical expenses for treatment of any such illness or injury. An HIV infection that occurs during the course of the trial will not be considered an injury or illness caused by trial participation.

## Communicable Disease Reporting

Study staff will comply with local requirements to report communicable diseases including HIV identified among study participants to health authorities. Participants will be made aware of reporting requirements during the informed consent process.

## Access to HIV-related Care

HIV test-related counseling will be provided to all potential study participants who consent to undergo HIV screening to determine their eligibility for this study, and to all enrolled participants at each follow-up HIV testing time point. Testing will be performed in accordance with the algorithm in [Appendix II](#_APPENDIX_II:__1). Counseling will be provided in accordance with standard HIV counseling policies and methods at each site and additionally will emphasize the unknown efficacy of the study products in preventing HIV infection. In accordance with the policies of the NIH, participants must receive their HIV test results to take part in this study.

### Care for Participants Identified as HIV-Positive

An individual who has been identified as infected with HIV will be managed or referred for management according to the local standard of care. Should a participant test positive for HIV after the Enrollment Visit, follow-up procedures will be performed as per [Section 7.5.1](#_Participants_Who_Become). Please refer to [Section 9.7](#_Toc470016045) for further details.

## Study Discontinuation

This study may be discontinued at any time by NICHD, the MTN, IPM, CCTN, the US FDA, OHRP, other government or regulatory authorities, or site IRBs.

# PUBLICATION POLICY

NICHD and MTN policies and a CTA between NICHD and IPM will govern publication of the results of this study. Any presentation, abstract, or manuscript will be submitted by the investigator to the MTN Manuscript Review Committee, DAIDS, NIAID, NIMH, NICHD and IPM for review prior to submission.

# APPENDICES

# APPENDIX I: SCHEDULE OF STUDY VISITS AND EVALUATIONS

|  | | | **Visit 1** SCR | **Visit 2** ENR (Day 0) | **Visits 3, 4** (Day 2,14) | **Visits 5, 8** (Day 28, 58) | **Visits 6, 9**  (Day 30, 60) | **Visits 7,10** (Day 44, 74) | **Visit 11** PUEV/Early Termination (Day 90) | **Visits 12-14**  (Day 91, 92, 93 or 94) | **Visits 15-18**  Final Phone Calls  (1, 4, 8, 12 wks after ring removal) |
| --- | --- | --- | --- | --- | --- | --- | --- | --- | --- | --- | --- |
| **ADMINISTRATIVE AND REGULATORY** | | |  | | | | | | | | |
| Obtain written informed consent(s) | | | X |  |  |  |  |  |  |  |  |
| Assign a unique Participant Identification (PTID) number | | | X |  |  |  |  |  |  |  |  |
| Assess and/or confirm eligibility | | | X | X |  |  |  |  |  |  |  |
| Collect demographic and background information | | | X |  |  |  |  |  |  |  |  |
| Collect/review/update locator information | | | X | X | X | X | X | X | X | X | X |
| Randomization | | |  | X |  |  |  |  |  |  |  |
| Provide reimbursement | | | X | X | X | X | X | X | X | X | **¥** |
| Schedule next visit/contact | | | * | * | X | X | X | X | X | X | * |
| **BEHAVIORAL**  **/COUNSELING** | | |  |  | | | | | | | |
| HIV pre- and post-test counseling | | | X | X | * | * | * | * | X | * | * |
| HIV/STI risk reduction counseling | | | X | X | * | * | * | * | X | * | * |
| Protocol counseling | | |  | X | X | X | X | X | X |  |  |
| Contraceptive counseling | | |  | X | X | X | X | X | X | X  Visit 14 only |  |
| Collect product use information | | |  |  |  |  | X |  | X |  |  |
| Behavioral assessment | | |  | X |  |  |  |  | X |  |  |
| In-depth Interview | | |  |  |  |  |  |  | X |  |  |
| **CLINICAL** | | |  |  | | | | | | | |
| Collect medical eligibility information | | | X | X |  |  |  |  |  |  |  |
| Collect/review/update medical/menstrual history | | | X | X | X | X | X | X | X | X | X |
| Collect/review/update concomitant medications | | | X | X | X | X | X | X | X | X | X  Visit 15 only |
| Perform physical exam (Targeted at Visits 3-10, 12-14) | | | X | X | * | * | * | * | X | * |  |
| Perform pelvic exam | | | X | X | *  Day 14 mandatory | * | X | X | X | * |  |
| Perform breast exam | | | X | X | * | * | * | * | X | * |  |
| Treat or prescribe treatment for RTI/UTI/STIs | | | * | * | * | * | * | * | * | * |  |
| Refer for evaluation of secondary amenorrhea | | |  |  |  |  |  |  |  |  | *  Visit 18 only |
| Digital exam by clinician to place or check VR | | |  | X |  |  | X |  |  |  |  |
| Provide available test results | | | X | X | X | X | X | X | X | X | X  Visit 15 only |
| Collect AEs | | |  | π | X | X | X | X | X | X | X  Visit 15 only |
| Review result of home pregnancy test | | |  |  |  |  |  |  |  |  | X |
| **LABORATORY** | | |  |  | | | | | | | |
| URINE | Pregnancy test | | X | X | *  Day 14 mandatory | * | X | * | X |  |  |
|  | Urine dipstick/culture | | * | * | * | * | * | * | * | * |  |
| BLOOD | HIV testing | | X | X | * | * | * | * | X | * |  |
|  | Plasma for archive | |  | X |  |  |  |  |  |  |  |
|  | Serum creatinine | | X | X | * | *  Day 28 mandatory | * | * | X | * |  |
|  | AST/ALT | | X | X | * | *  Day 28 mandatory | * | * | X |  |  |
|  | CBC with differential and platelets | | X | X | * | *  Day 28 mandatory | * | * | X | * |  |
|  | Syphilis serology | | X | * | * | * | * | * | * | * |  |
|  | DPV concentration | |  | ▲ | X | X⌂ | X♦ | X | ∞ | X |  |
|  | Progestogen concentrations including LNG | |  | ▲ | X | X⌂ | X♦ | X | ∞ | X |  |
|  | Sex hormone-binding globulin (SHBG) and albumin | |  | X |  |  |  |  | X |  |  |
|  | Serum progesterone and estradiol | |  | X | X | X | X | X | X |  |  |
| PELVIC | NAAT for GC/CT and trichomonas | | X | * | * | * | * | * | * | * |  |
|  | Saline/potassium hydroxide (KOH) wet mount with pH for candidiasis and/or bacterial vaginosis (BV) | | * | * | * | * | * | * | * | * |  |
|  | Pap test | | ^ |  |  |  |  |  |  |  |  |
|  | Genital lesion testing for HSV | | * | * | * | * | * | * | * | * |  |
|  | Vaginal Gram stain | |  | X |  |  |  | X | X |  |  |
|  | CVF DPV concentration | |  | ▲ | X | X⌂ | X♦ | X | ∞ | X |  |
|  | CVF LNG concentration | |  | ▲ | X | X⌂ | X♦ | X | ∞ | X |  |
|  | Cervical biopsies for DPV concentration and PD | |  |  | X  Day 14 only |  | X♦  Day 30 only |  | ∞ |  |  |
|  | CVF for microbiota | |  | ▲ |  |  |  | X |  |  |  |
|  | CVL for biomarkers | |  | ▲ |  |  |  | X |  |  |  |
| **STUDY PRODUCT SUPPLY** | | |  |  | | | | | | | |
| Provision of study VR | | |  | X |  |  | X  Group 2 only |  |  |  |  |
| Insertion of the provided study VR by the participant (or clinician/designee) | | |  | X |  |  | X  Group 2 only |  |  |  |  |
| Provision/review of study VR use instructions | | |  | X |  |  | X |  |  |  |  |
| Removal and collection of study VR | | |  |  |  | X  Group 2 only |  |  | X |  |  |
| Provide home pregnancy test kits | | |  |  |  |  |  |  |  | X  Visit 14 only |  |
| Offer male condoms | | | **☼** | **☼** | **☼** | **☼** | **☼** | **☼** | **☼** | **☼** |  |
|  | | X Required  ¥ Per site policy specified in the SSP  * If indicated and/or per local standard of care  **π** To be collected following randomization  ▲To be collected at baseline prior to ring insertion  ⌂ To be collected prior to or at the time of ring removal for participants in Group 2  ♦ To be collected prior to ring insertion for participants in Group 2  ∞ To be collected immediately prior to ring removal  ^ If indicated (If participant over age 21 is unable to provide documentation of a satisfactory Pap test within 3 years prior to  enrollment)  **☼** Details are specified in the SSP | | | | | | | | | |

# APPENDIX II: ALGORITHM FOR HIV TESTING FOR SCREENING AND ENROLLED PARTICIPANTS


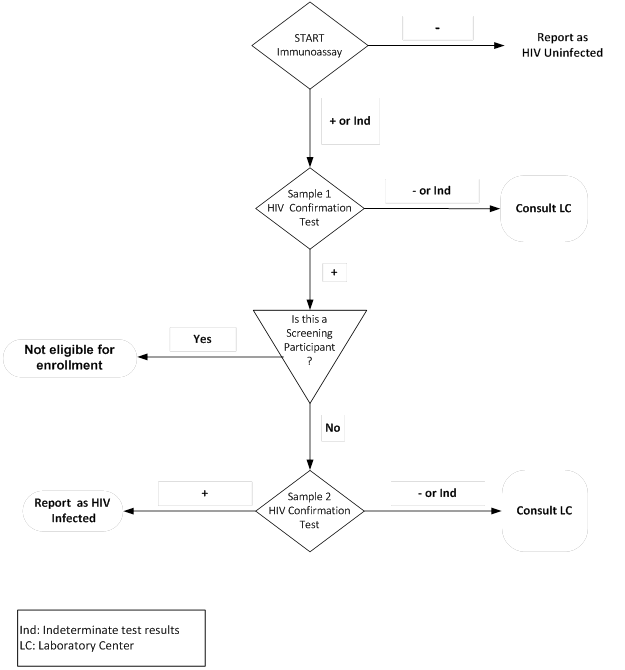


# REFERENCE LIST

1. UNAIDS. Fact sheet - Latest statistics on the status of the AIDS epidemic. 2017. (Accessed 12/12/17, 2017, at http://www.unaids.org/en/resources/fact-sheet.)

2. UNAIDS. AIDS by the numbers. AIDS is not over, but it can be. 2016. (Accessed December 12, 2017, at http://www.unaids.org/sites/default/files/media_asset/AIDS-by-the-numbers-2016_en.pdf.)

3. UNAIDS. Global report: UNAIDS report on the global AIDS epidemic 2013. 2013.

4. Centers for Disease Control and Prevention . HIV among Women. 2017. (Accessed December 12, 2017, at https://www.cdc.gov/hiv/group/gender/women/.)

5. amfAR. Statistics: Women and HIV/AIDS. 2017. (Accessed December 12, 2017, at http://www.amfar.org/About-HIV-and-AIDS/Facts-and-Stats/Statistics--Women-and-HIV-AIDS/.)

6. UNAIDS. 2015 Progress Report on the Global Plan – towards the elimination of new HIV infections among children and keeping their mothers alive. (Accessed December 12, 2017, at http://www.unaids.org/en/resources/documents/2015/JC2774_2015ProgressReport_GlobalPlan.)

7. Kott A. Rates of Unintended Pregnancy Remain High in Developing Regions International Perspectives on Sexual and Reproductive Health 2011;37.

8. WHO. Fact sheet: maternal mortality. November 2016 update. (Accessed December 12, 2017, at Available at: http://www.who.int/mediacentre/factsheets/fs348/en/ )

9. WHO. Trends in maternal mortality 1990 to 2015. (Accessed December 12, 2017, at http://apps.who.int/iris/bitstream/10665/194254/1/9789241565141_eng.pdf?ua=1.)

10. Thurman AR, Clark MR, Hurlburt JA, Doncel GF. Intravaginal rings as delivery systems for microbicides and multipurpose prevention technologies. International journal of women's health 2013;5:695-708.

11. Karim SA, Baxter C, Frohlich J, Karim QA. The need for multipurpose prevention technologies in sub-Saharan Africa. BJOG : an international journal of obstetrics and gynaecology 2014;121 Suppl 5:27-34.

12. Multipurpose Prevention Technologies: Providing women and young girls with more choices for their sexual and reproductive health needs. (Accessed December 20, 2017, at http://www.who.int/reproductivehealth/topics/linkages/mpts/en/.)

13. IPM. Investigator's Brochure: Dapivirine Vaginal Ring (Version 10.0 Final). 2016.

14. Bunge KE, Dezzutti CS, Rohan LC, et al. A Phase 1 trial to assess the safety, acceptability, pharmacokinetics, and pharmacodynamics of a novel dapivirine vaginal film. JAIDS 2016;71:498-505.

15. Robinson JA, Marzinke MA, Bakshi RP, et al. Comparison of dapivirine vaginal gel and film formulation pharmacokinetics and pharmacodynamics (FAME 02B) (Epub ahead of print). AIDS Research and Human Retroviruses 2016.

16. Bunge KE, Dezzutti CS, Macio I, et al. FAME-02: A Phase I trial To assess safety, PK, and PD of gel and film formulations of dapivirine. Conference on Retroviruses and Opportunistic Infections 2014; 2014 3/3/2014; Boston, Massachusetts (USA): International Antiviral Society-USA; 2014. p. 110-1.

17. IPM. Investigator's Brochure: Dapivirine Vaginal Gel, Including Rectal Gel (Version 8.0 Final). 2015.

18. Gruzdev B, Horban A, Boron-Kaczmarska GD, Vant-Klooster G, Pauwels R. TMC120, a new non-nucleoside reverse transcriptase inhibitor, is a potent antiretroviral in treatment naive, HIV-1 infected subjects (Abstract 13). 8th Conference on Retroviruses and Opportunistic Infections (CROI); 2001 2/4/2001; Chicago, IL, USA; 2015.

19. DeBethune MP, Andries K, Ludovici D, et al. TMC-120 (R147681), a next generation NNRTI has potent in vitro activity against NNRTI-resistant HIV variants (Abstract 304). 8th Conference on Retroviruses & Opportunistic Infections (CROI); 2001 2/4/2001; Chicago, IL, USA; 2015.

20. Baeten JM, Palanee-Phillips T, Brown ER, et al. Use of a vaginal ring containing dapivirine for HIV-1 prevention in women. N Engl J Med 2016;375:2121-32.

21. Nel A, Kapiga S, Bekker L-G, et al. Safety and efficacy of dapivirine vaginal ring for HIV-1 prevention in African women. 2016 Conference on Retroviruses and Opportunistic Infections (CROI); 2016 2/22/2016; Boston, MA, USA; 2016.

22. Fletcher P, Harman S, Azijn H, et al. Inhibition of human immunodeficiency virus type 1 infection by the candidate microbicide dapivirine, a nonnucleoside reverse transcriptase inhibitor. Antimicrob Agents Chemother 2009;53:487-95.

23. Nel AM, Coplan P, Smythe SC, et al. Pharmacokinetic assessment of dapivirine vaginal microbicide gel in healthy, HIV-negative women. AIDS Res Hum Retroviruses 2010;26:1181-90.

24. Di FS, Van RJ, Giannini G, et al. Inhibition of vaginal transmission of HIV-1 in hu-SCID mice by the non-nucleoside reverse transcriptase inhibitor TMC120 in a gel formulation. AIDS 2003;17:1597-604.

25. Anderson RM, Swinton J, Garnett GP. Potential impact of low efficacy HIV-1 vaccines in populations with high rates of infection. Proc Biol Sci 1995;261:147-51.

26. Dezzutti CS, Yandura S, Wang L, et al. Pharmacodynamic Activity of Dapivirine and Maraviroc Single Entity and Combination Topical Gels for HIV-1 Prevention. Pharm Res 2015;32:3768-81.

27. Schader SM, Oliveira M, Ibanescu RI, Moisi D, Colby-Germinario SP, Wainberg MA. In vitro resistance profile of the candidate HIV-1 microbicide drug dapivirine. Antimicrob Agents Chemother 2012;56:751-6.

28. IPM. Investigator's Brochure: Dapivirine-Levonorgestrel Vaginal Ring (Version 4.0). 2017.

29. Nel A, van NN, Kapiga S, et al. Safety and Efficacy of a Dapivirine Vaginal Ring for HIV Prevention in Women. N Engl J Med 2016;375:2133-43.

30. Devlin B, Nuttall J, Wilder S, Woodsong C, Rosenberg Z. Development of dapivirine vaginal ring for HIV prevention. Antiviral Res 2013;100 Suppl:S3-S8.

31. Chen BA, Panther L, Marzinke MA, et al. Phase 1 Safety, Pharmacokinetics, and Pharmacodynamics of Dapivirine and Maraviroc Vaginal Rings: A Double-Blind Randomized Trial. J Acquir Immune Defic Syndr 2015;70:242-9.

32. IPM. Investigator's Brochure: Dapivirine-Levonorgestrel Vaginal Ring (Version 3.0 Final). 2016.

33. Cranston RD, Hoesley C, Carballo-Dieguez A, et al. A randomized male tolerance study of dapivirine gel following multiple topical penile exposures (MTN 012/IPM 010). AIDS Res Hum Retroviruses 2014;30:184-9.

34. Brown E, Palanee-Phillips T, Marzinke M, et al. Residual dapivirine ring levels indicate higher adherence to vaginal ring is associated with HIV-1 protection (Abstract). AIDS 2016: 21st International AIDS Conference: International AIDS Society; 2016.

35. Boyd P, Fetherston SM, McCoy CF, et al. Matrix and reservoir-type multipurpose vaginal rings for controlled release of dapivirine and levonorgestrel. Int J Pharm 2016;511:619-29.

36. Bayer. Mirena Package Insert. 2017.

37. Bayer. Skyla Package Insert. 2017.

38. Koetsawang S, Ji G, Krishna U, et al. Microdose intravaginal levonorgestrel contraception: a multicentre clinical trial. IV. Bleeding patterns. World Health Organization. Task Force on Long-Acting Systemic Agents for Fertility Regulation. Contraception 1990;41:151-67.

39. Thurman A. Levonorgestrel/tenofovir intravaginal ring. Microbicide Trials Network (MTN) Annual Meeting 2016; 2016 3/13/2016; North Bethesda, MD, USA; 2016.

40. Thurman A, Schwartz J, Brache V, et al. Pharmacokinetics/pharmacodynamics of tenofovir and tenofovir plus levonorgestrel vaginal rings in women (Poster, P25.02). HIV R4P; 2016 10/18/2016; Chicago, IL, USA; 2016.

41. Holt J, Dias N, Bartlett S, et al. Pharmacokinetics of dapivirine and levonorgestrel in sheep when administered vaginally in a silicone matrix ring. HIV R4P; 2016.

42. Holt J, Evans A, Shattock R, Pessaint L, Attardi B, Nuttall J. Assessment of the potential for pharmacodynamic interaction between dapivirine and levonorgestrel when co-administered in an MPT vaginal ring (Oral abstract; OA06.06)). HIV R4P; 2016 10/18/2016; 2016.

43. Achilles SL. The path to the MPT ring containing DPV and LNG: MTN-030 and MTN-044. Presented at the MTN 2018 Annual Meeting on March 20, 2018. 2018.

44. United States Medical Eligibility Criteria (US MEC) for Contraceptive Use, 2016. Centers for Disease Control and Prevention (US), 2016. (Accessed 8/29/2016, 2016, at http://www.cdc.gov/reproductivehealth/contraception/usmec.htm.)

45. IPM. Investigator's Brochure: Dapivirine Vaginal Ring (Version 11.0 Final). 2017.

46. IPM. Investigator's Brochure: Dapivirine-Levonorgestrel Vaginal Ring (Ver. 3.0 Final): Addendum 1-Ver. 1.0. 2016.

**CONSENT TO ACT AS A PARTICIPANT IN A RESEARCH STUDY**

**(SCREENING, ENROLLMENT, LONG-TERM STORAGE AND FUTURE TESTING)**

| **Sponsor / Study Title:** | **US Eunice Kennedy Shriver National Institute of**  **Child Health and Human Development / “A Randomized, Phase 1, Open-Label Study in Healthy HIV-Negative Women to Evaluate the Pharmacokinetics, Safety and Bleeding Patterns Associated with 90-Day Use of Matrix Vaginal Rings Containing 200 mg Dapivirine and 320 mg Levonorgestrel”** |
| --- | --- |
| **Protocol Number:** | **MTN-044/IPM 053/CCN019** |
| **Principal Investigator:**  **(Study Doctor)** | **«PiFullName»** |
| **Telephone:** | **«IcfPhoneNumber»** |
| **Additional Contact(s):**  **(Study Staff)** | **«AdditionalStaffMemberContacts»** |
| **Address:** | **«PiLocations»** |

**SOURCES OF SUPPORT:** US National Institutes of Health (NIH) and conducted by the Microbicide Trials Network (MTN) and the Contraceptive Clinical Trials Network (CCTN). The study rings are supplied by the International Partnership for Microbicides (IPM), a not-for-profit research organization.

Please take time to read this entire form and ask questions before deciding whether to take part in this research project. If you do decide to take part in the trial, you will sign and date your name on this form. A copy of this form will be offered to you. Signing this consent form does not mean you will be able to join the study. You must first complete the screening tests and exams to see if you are eligible. It is important to know that your participation in this research is your decision and taking part in this study is completely voluntary (see *Is my participation in this research study voluntary?* for more information). Take time to ask the study doctor or study staff as many questions about the study as you would like. The study doctor or study staff can explain words or information that you do not understand. Reading this form and talking to the study doctor or study staff may help you decide whether to take part or not.

**IMPORTANT INFORMATION ABOUT THE RESEARCH STUDY**

You are invited to participate in a research study. In order to participate, you must be a healthy, HIV-negative female between the ages of 18 and 45 years old who has current regular menstrual cycles. Taking part in this research project is voluntary.

Important things you should know:

- The study product in this clinical trial is a vaginal ring (VR) containing a combination of the anti-HIV medication dapivirine (DPV) and an FDA-approved contraceptive hormone called levonorgestrel (LNG)
- The study product is NOT for use to prevent pregnancy in this particular study. You may only participate if you are, and will continue taking steps that prevent pregnancy, or that pose no chance of pregnancy. The study staff will carefully assess this with you and answer your questions to help determine if you may participate.
- You should NOT depend upon the study product to prevent HIV, the virus that causes AIDS. If you are concerned about exposure to HIV you should discuss this with the study staff, who can advise you about your options.
- The purposes of the study are:
  - To find out how the two drugs (DPV and LNG) enter and exit the body when one VR containing the two drugs is inserted into the vagina and worn continuously or cyclically for approximately 90 days
  - To find out if the VR is safe and well-tolerated
  - To look at the effects of the VR on vaginal bleeding
- If you qualify and choose to participate, you will be randomly assigned (like flipping a coin) to one of two groups:
  - Group 1 will use the VR continuously for approximately 90 days
  - Group 2 will use the VR cyclically (use for approximately 28 days, take it out for 2 days and insert the same VR back) for a total of approximately 90 days
- Once enrolled, you will be asked to attend 13 clinic visits at the research clinic over approximately 3 months (94 days) and will be contacted by phone up to 4 times during the 3 months after the ring is removed. The duration of your study participation will be approximately 6 months.
- At the clinic visits, a physical and/or pelvic exam will be performed, blood will be obtained, and vaginal swabs and samples will be collected. At three visits, cervical biopsies will be obtained.
- You may not experience any direct benefit from participation in this study. However, information learned from this study may help in the development of ways to prevent unwanted pregnancy and the spread of HIV in the future.
- Taking part in this research project is voluntary. You don’t have to participate and you can stop at any time.

**STUDY PRODUCTS**

**Dapivirine (DPV)**

DPV VRs were recently tested in two large studies and were shown to be safe and helped to prevent HIV acquisition. HIV is the virus that causes AIDS. DPV works by preventing HIV from making copies of itself, which stops the spread of HIV in the body. Study staff can provide you with additional information about these studies if you are interested in learning more.

**Levonorgestrel (LNG)**

Many contraceptives such as pills and implants contain progestin hormones like LNG. The hormone thickens the mucus of the cervix (the opening to the uterus) to prevent sperm from reaching an egg. It may also keep women from releasing an egg so they do not get pregnant. Currently there is one approved VR available in the United States (called NuvaRing®), which contains a different progestin hormone, etonogestrel, as well as an estrogen hormone, ethinyl estradiol. The new VR being tested in this study contains the progestin hormone LNG.

**Dapivirine/Levonorgestrel combination**

This is the second study of a VR containing both DPV and LNG. The first study included both a VR containing 200 mg DPV without LNG and the combination DPV-LNG VR (200 mg DPV and 320 mg LNG) used for 14 days in healthy women. There were no safety concerns in that study. **Researchers do not know if LNG prevents pregnancy when combined with DPV in a VR. Therefore, it is important that you use another form of contraception that is non-hormonal during your study participation.**

**STUDY GROUPS**

Approximately 24 eligible participants will be randomized equally to one of two VR use study groups:

- Group 1 will use the DPV-LNG VR continuously for approximately 90 days
- Group 2 will use the DPV-LNG VR cyclically for approximately 90 days as follows: use the VR for approximately 28 days, then remove, wash and store for 2 days. The same ring will be used for 2 additional cycles in a similar fashion.

Approximately 12 participants will be assigned to each VR use group. Participants will be assigned to a group by random chance (like flipping a coin), and both you and the study staff will know which group you are in. Both study groups are important. No matter which study group you are in, you must remember that we do not know if the drugs contained within the VR will work to protect you against pregnancy or from getting HIV.

**WHAT WILL HAPPEN DURING THE STUDY VISITS?**

The study includes a total of up to 18 visits or contacts. Fourteen visits will take place at this clinic, including the screening visit, and up to 4 contacts will be conducted with you by phone after ring removal on Day 90.

**The study staff will go over the visit schedule chart, below, with you, provide details, and answer any questions you have.**


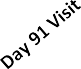

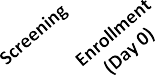

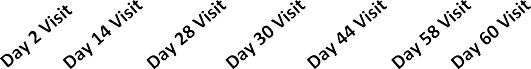

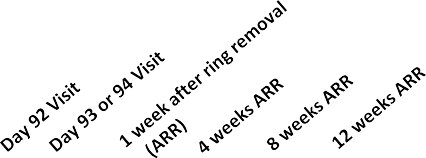

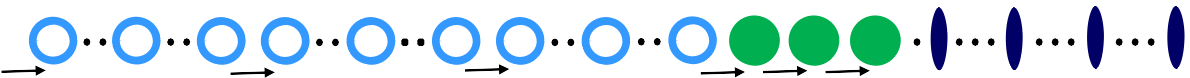

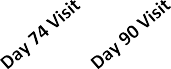


***≤*60**

**days**

2 days

2 days

2 days

1 day 1 day 1 or 2 days

**Visit 1**

**V2 V3**

**V4**

**V5 V6**

**V7**

**V8 V9**

**V10 V11 V12 V13 V14 V15 V16 V17 V18**

**(up to 4 phone calls/contacts)**

You will discuss with study staff the rules of the study and your understanding of the rules, including but not limited to:

- - Keeping the vaginal ring in place and not removing it between study visits.
  - Using another form of contraception that is non-hormonal during your study participation.
  - Being sexually abstinent and avoiding tampon use for the 24 hours prior to your Enrollment Visit and clinical visits where samples are taken and for one week following cervical biopsies. This means no vaginal sexual intercourse with a male partner, no oral sex or no vaginal finger stimulation.
  - Refraining from inserting any non-study vaginal products or objects into the vagina for the 24 hours prior to your Enrollment Visit through completion of Visit 15. These include but are not limited to spermicides, female condoms, diaphragms, other intravaginal rings, vaginal medications, menstrual cups, cervical caps (or any other vaginal barrier method), vaginal douches, non-study lubricants and moisturizers, or sex toys (vibrators, dildos, etc.).
  - Avoiding continued use of certain medications and herbal supplements, such as St. John’s wort, that may interact with the study drugs.

You will also be asked about your study product use and/or vaginal bleeding or spotting for the duration of your participation. Your answers will be kept private.

***What procedures will be performed for research purposes?***

The procedures performed in this study are done for research purposes and will be performed by a study doctor, clinician, or other trained member of the study team. The results will become part of your research record. During the study, the study doctors will review the results of all labs, evaluations, and procedures that monitor your health and safety, and you will be given these results after they are available.

At any time during this study, if tests show you might have medical problems, including infections passed through sex, then you may have a physical or pelvic exam; give blood, urine, or vaginal samples to test for infections; and, if needed, get referrals for medical care and treatment. If HIV, gonorrhea, Chlamydia, or syphilis is identified, we are required to confidentially report this to the local health department with your name and contact information. Someone from the health department may contact you to be sure that you and your partner have been treated.

**Visit 1 Screening**

This visit may continue today or be done within 60 days before Visit 2 Enrollment to determine if you are eligible to take part in this research study. At this visit, which may take ***[SITE TO INSERT TIME***], you will:

- Answer questions to confirm your understanding of the study requirements and that you are able to join the study
- Study staff will:
- Ask you about your contact information (for example, where you live and how we can contact you) and other questions about you, including your education, your behavior including your sexual behavior, your medical history (including what medications you are taking) and menstrual history. They may also ask to view your medical records, with your permission.
- Test your urine for pregnancy and, if needed, for a urinary tract infection.
  - - If you are pregnant you cannot join this study.
    - Study staff will talk with you about ways to avoid becoming pregnant.
    - You will answer questions about whether you are using an effective, non-hormonal method of contraception and intend to use this method for the entire time that you are in this study.
    - If needed, study staff will provide and/or refer you to obtain an acceptable contraceptive method for use during your participation in the study. Acceptable methods include:
      - Non-hormonal (for example, copper) intrauterine devices (IUDs) inserted at least 28 days (4 weeks) prior to enrollment
      - Consistent and correct male condom use
      - Sterilization: You or your partner has been sterilized (tubal ligation, vasectomy, etc.)
      - You engage in sex exclusively with women
      - Sexually abstinent for the past 90 days and planning to remain abstinent for the duration of study participation
- Perform a general physical examination including vital signs (such as blood pressure, heart rate, and temperature), height and weight
- Perform a breast examination
- Perform a pelvic examination:
  - The study doctor will use a speculum, a plastic or metal instrument inserted in the vagina. Study staff will ask if you are experiencing symptoms of an infection. They will check your vagina and cervix for signs of infection and other problems.
  - A small amount of vaginal fluid will be collected via swab(s), like a Q-tip. These swabs will be used to test for sexually transmitted infections (commonly known as STIs or STDs) and other possible problems.
  - If you are 21 years of age or older, samples may be collected from your cervix for a “Pap test” or “Pap smear”. If you have a written report confirming a normal Pap test in the past 3 years, or if you had an abnormal Pap test but had follow-up indicating no treatment was required, then you will not need to have a Pap test. The results of your Pap test may affect whether or not you can continue in the study. You will receive the results of this test when it is available.
- Talk with you about HIV and other sexually transmitted infections (STIs), HIV and STI testing, and ways to avoid HIV and STIs.
- Have ***[SITE TO INSERT AMOUNT]*** of blood drawn from a vein in your arm to test the health of your blood, liver and kidneys and to test for HIV and syphilis. These tests are being done to make sure that the blood test results are normal at the start of the study and to test for infections that are typically passed through sex.
  - You will be told your HIV and other test results as soon as they are available. You will talk with the study staff about the meaning of your results and how you feel about them. Sometimes HIV tests are not clearly positive, but also not clearly negative. In that case, we will do more tests until we know your status for sure. You must receive your HIV test results to be in the study. If the test shows you have HIV, you cannot join the study. We will refer you to available sources of medical care and other services you may need. Medical care for HIV infection will not be part of this study. The study staff will tell you about other studies you may be eligible for, if any.
- If needed, give you treatment or refer you for treatment of STIs or other urinary or reproductive tract infections
- Inform you about other services, if needed
- Provide you with the results of your tests, when available
- Reimburse you for your visit
- Talk with you about the requirements of the study, including the importance of completing clinic visits, and study activities and procedures according to the study schedule
- Give you male condoms, if you need them
- Schedule your next visit to enroll in the study, if you are willing and eligible.

Results of the tests listed above will be available within ***[SITE TO SPECIFY TIMEFRAME]***. The study staff will review your test results with you. If you are not eligible or decide not to participate in this study, no blood collected at Screening will be kept or used for any tests other than those listed above.

**Visit 2 Enrollment (Day 0)**

The Enrollment Visit is when you join the study. This visit will take about ***[SITE TO INSERT TIME***]. In addition to the procedures listed below, it is possible that study staff may need to perform additional tests if medically necessary (for example, you report having symptoms of a urinary, genital, or other infection and/or other issues).

The following procedures are specific to the Enrollment Visit, which will take place up to 60 days (approximately 8 ½ weeks) after your Screening Visit:

- You will answer questions to confirm you are able to join the study.
- You will update study staff with your contact information (for example, where you live and how we can contact you).
- Study staff will:
- Talk with you about the requirements of the study and how to follow them, including restrictions on sexual practices. **If you do not think you can abstain from sex as required by the study, then you should not join this study.** Sex for this study is defined as vaginal sexual intercourse with a male partner, oral sex or vaginal finger stimulation.
- Ask you questions about your vaginal practices that may affect how your body absorbs the study drugs.
- Ask you questions about your thoughts on the study product
  - A staff member may ask you these questions. It is important that you know that you will answer these questions in private and your responses will be kept confidential.
- Talk with you about STIs, HIV, HIV/STI testing, and ways to avoid HIV and other infections passed through sex
- Discuss any health or medical problems you may have had in the past, are currently experiencing or that have occurred since your last visit (including what medications you are taking)
- Ask you about any menstrual periods or spotting you may have had since your last visit
- Give you male condoms, if you need them.
- You will also:
- Have your urine tested for pregnancy and, if needed, for a urinary tract infection
- Have a general physical examination
- Have a breast examination
- Have about ***[SITE TO INSERT AMOUNT]*** of blood collected before you receive the study VR to test the health of your blood, liver and kidneys; to test for HIV; for study purposes including to test for the amount of DPV and LNG present in your blood when you start using the VR; for sex hormone (progesterone, estradiol), a hormone-related protein (sex hormone binding globulin, or SHBG), and albumin; and for storage in the event there is a question about your lab results in the future. After all testing is done, this stored sample will be destroyed.
- Have a pelvic examination using a speculum
  - Your vagina and cervix will be checked for signs of infection or other problems, and you will be asked if you are experiencing symptoms of an infection. Vaginal fluid will be collected with a swab to test for bacteria and organisms and, if needed, for STIs or other possible problems.
  - A small amount of vaginal fluid will be collected via swab before you receive the study VR. A study doctor will insert the swab approximately 3 inches inside your vagina. Swabs will be collected to measure the amount of DPV, LNG, and bacteria present in the vagina when you start using the VR. The same tests will be done when these samples are collected at future visits.
  - A cervicovaginal lavage (CVL) will be performed. For the CVL, a study doctor will rinse your vagina and cervix with a small amount of sterile fluid and collect that fluid in a tube for testing. The CVL fluid collected will be used for research purposes only.
- Receive and insert the study VR. Study staff may help you insert the VR if you cannot do it on your own. All participants will have a vaginal examination by a study doctor to ensure the ring is inserted correctly. You will be asked to keep the VR in place and not remove it between visits. Study staff will show you how to take the ring out in case you need to do so. Study staff will talk with you about what to do if you have any problems or symptoms while using the ring.
- Be told that you will be asked to answer questions regarding your ring use and any vaginal bleeding or spotting you may experience during the study product use period
- Be given instructions about the short message service (SMS) text message system that will be used in this study. At a time that is convenient for you, this confidential system will send you a few short questions by cell phone via daily text messages that will ask about your vaginal bleeding or spotting and via weekly text messages about your use of the study product. If you do not have a cell phone with texting service, the study team will provide you with one.
- Receive treatment or be referred for treatment for any issues that the study staff may find
- Receive test results, if available
- Talk with study staff about any of your questions. Study staff will talk with you if you encounter any problems or symptoms while undergoing any of the procedures listed above.
- Be reimbursed for your visit
- Schedule your next visit, if applicable.

**Follow-Up Visits**

After Visit 2 Enrollment (Day 0), you will have Visits 3-14 on Days 2, 14, 28, 30, 44, 58, 60, 74, 90, 91, 92, and 93 or 94 as listed in the chart above. Most study visits will take about ***[SITE TO INSERT TIME****],* except for Visit 11 (Day 90) which may take about ***[SITE TO INSERT TIME****].* Study staff can answer any questions you may have about the procedures listed below.

At each follow-up visit (except where noted otherwise), you will:

- Update study staff with your contact information
- Provide study staff information about your study product use
- Discuss any health or medical problems you may be currently experiencing or that have occurred since your last visit (including what medications you are taking)
- Talk with study staff about STIs, HIV, HIV/STI testing, and ways to avoid HIV and other infections passed through sex, if needed
- Be asked about any vaginal bleeding or spotting you may have had since your last visit
- Talk with study staff about any problems that you may be experiencing related to using the VR or procedures performed during your last visit
- As needed, study staff will speak with you again and answer your questions about the requirements of the study and using the VR, including keeping the VR in place and not removing it between visits
- Undergo a targeted physical exam, if indicated
- Have about ***[SITE TO INSERT AMOUNT****]* of blood collected for research purposes:
  - to measure the levels of study drugs
  - to test for serum progesterone and estradiol
- Have a pelvic examination, if indicated
- Study staff will ask if you are experiencing symptoms of an infection
- If indicated, the study doctor may use a speculum to check your vagina and cervix for signs of problems due to the ring or infection, and to collect vaginal fluid to test for bacteria and organisms
- Have a breast examination, if indicated
- Have vaginal fluid samples collected via swabs to measure the levels of study drugs
- Receive any available test results
- Be reimbursed for your visit
- Have your next visit scheduled
- Receive male condoms, if you need them.

In addition to the procedures listed above, it is possible that study staff may need to perform additional tests if medically necessary (for example, you report having symptoms of a urinary, genital, or other infection and/or other issues).

**Visit 4 (Day 14)**

At this visit, you will also:

- Have your urine tested for pregnancy
- Have a pelvic examination with a speculum
- Undergo a cervical biopsy. Study doctors will take approximately 3 small tissue samples from your cervix, each about the size of a grain of rice. These samples will be used to see how much of the study drug is in your tissue and for research purposes. It is important that you do not put anything in your vagina for 24 hours before the collection of the biopsies and one week after, including to avoid sexual intercourse, because you may be at higher risk for getting or spreading an infection until the biopsy sites have healed.

**Visits 5 and 8 (Day 28 and 58)**

Visit 5 and Visit 8 will take place after you have worn the VR for approximately 28 and 58 days after your Enrollment visit respectively. At these visits, you will also:

- Remove the VR, wash and store it in the clinic for 2 days (Group 2 participants only)

**Visits 6 and 9 (Days 30 and 60)**

Visit 6 and Visit 9 will take place after you have worn the VR for approximately 30 and 60 days respectively. At these visits, you will also:

- Have your urine tested for pregnancy
- Have a pelvic examination
- Undergo a cervical biopsy (Visit 6 only). Study doctors will take approximately 3 small tissue samples from your cervix, each about the size of a grain of rice
- Re-insert the study VR that had been removed and stored in the clinic for 2 days with a vaginal exam performed by a study clinician after the VR is in place again (Group 2 participants only).

**Visits 7 and 10 (Day 44 and 74)**

Visit 7 and Visit 10 will take place after you have worn the VR for approximately 44 and 74 days after your Enrollment visit respectively. At these visits, you will also:

- Have a pelvic examination
- Have vaginal fluid samples collected with swabs to test for bacteria
- Have vaginal fluid collected by rinsing your vagina with a small amount of sterile salt water.

**Visits 11 – PUEV/Early Study Termination Visit Ring Removal (Day 90)**

Visit 11 will take place after you have worn the VR for approximately 90 days. At this visit, you will also:

- Have your urine tested for pregnancy
- Have about ***[SITE TO INSERT AMOUNT****]* of blood drawn for HIV testing; to test the health of your blood, liver and kidneys; and to test for a hormone-related protein (sex hormone binding globulin, or SHBG) and albumin
- Talk with study staff about STIs, HIV, HIV/STI testing, and ways to avoid HIV and other infections passed through sex
- Have a general physical examination
- Have a breast examination
- Have a pelvic examination
- Have vaginal fluid samples collected to test for bacteria via swabs
- Undergo a cervical biopsy. Study doctors will take approximately 3 small tissue samples from your cervix, each about the size of a grain of rice.
- Remove the study VR
- Be asked to participate in an in-depth interview with a trained staff member to discuss your experiences during study participation. You will be asked questions about your experience with using the ring, your preferences and opinions, any problems you may have had using the ring. The interviews will be audio-recorded to make sure to record your words exactly how you said them. The audio recording, notes, and analyses from these materials will be kept confidential and will only use study numbers or made up names, and the hardware will be physically protected in a locked area. This means that no one other than the study team will have access to your responses. The information that links you to the research materials will be kept in a secure location that will be accessed only by members of the MTN-044/IPM 053/CCN019 study team for the purposes of this research.

**Visit 14 (Day 93 or 94)**

Visit 14 will take place 3 or 4 days after removing the study VR. At this visit, you will also:

- Talk with study staff about the contraceptive method that you would like to use after this visit
- Schedule your first follow-up phone call
- Receive urine pregnancy test kits to perform at home on the morning of your follow-up phone calls

**Visit 15-18/Phone Calls (1, 4, 8 and 12 Weeks After Ring Removal)**

For Visits 15-18, you will be contacted by phone at home during the 12 weeks after you have removed the study VR. The Week 1 phone call is required, but the phone calls at 4, 8, and 12 weeks after ring removal will only be conducted if you do not use birth control with hormones or if your menses has not returned since the previous phone call. You will be asked to perform a pregnancy test at home on the morning of each call and will be followed for up to three months to find out when your first period comes after the VR is removed. Each phone call will take between ***[SITE TO SPECIFY TIMEFRAME]*** to complete.

At each applicable phone call, you will:

- Update study staff with your contact information
- Discuss any health or medical problems you may be currently experiencing or that have occurred since your last visit (including what medications you are taking) (Visit 15 only)
- Talk with study staff about STIs, HIV, HIV/STI testing, and ways to avoid HIV and other infections passed through sex, if needed
- Receive any available test results (Visit 15 only)
- Be asked if you have had your period since your last phone call
- Be asked about the result of the home pregnancy test you perform on the morning of the phone call
- Schedule next visit/contact, if necessary.

If you do not have a period within 3 months after ring removal, you will be referred for medical evaluation.

**Additional Visits and Procedures**

It may be necessary for you to have additional visit(s) and/or provide additional samples if any of the study procedures at any visit need to be repeated due to one or more of the following:

- Issues with sample processing, testing or shipping
- If you are experiencing any symptoms or changes in your physical condition
- If tests or procedures were missed or not conducted.

Additional testing may be performed as part of quality control.

***What are the possible risks, side effects and discomforts of the study?***

As with any research study, there may be adverse events or side effects that are currently unknown and could be permanent, severe, or life threatening. The VRs used in this study may have side effects, some of which are listed below. Please note that this list does not include all the side effects that are possible with these VRs. This list includes the more serious or common side effects with a known or possible relationship. If you have questions concerning VR side effects, please ask the study staff at your site. You should report any new or continuing symptoms to the researchers right away. A study doctor is available 24 hours a day, every day of the week in case you have any questions or need to report any problems.

**Risks of Vaginal Rings**

Use of the study VR may lead to vaginal symptoms, including:

- Irritation
- Increased discharge
- Discomfort (including with vaginal intercourse if it were to occur)

It is possible that a participant may have an allergic reaction to the study product. Symptoms of an allergic reaction include rash or other skin irritation, itching, joint pain, or difficulty in breathing.

**Risks of Dapivirine Vaginal Rings**

Based on data from the two large studies looking at the effectiveness of the DPV VR, the most commonly reported side effects (occurring in greater than or equal to 10% of participants){(IPM), 2016 #528;(IPM), 2016 #528} are listed below; it should be noted that a causal association has not yet been determined.

- Metrorrhagia (irregular uterine bleeding)
- Chlamydia (STI)
- Urinary tract infection
- Menorrhagia (heavy or prolonged menstrual bleeding)
- Gonorrhea (STI)
- Upper respiratory tract infection
- Vaginal yeast infection
- Female genital infection
- Bacterial vaginosis (vaginal infection)

As with any product inserted vaginally, the possibility of toxic shock syndrome exists. Toxic shock syndrome is a rare but serious illness caused by poisons (toxins) released by some types of *Staphylococcus aureus*, a common bacterium. While the likelihood of this occurring is rare, it is important that you alert the study staff if you experience any symptoms associated with toxic shock syndrome, such as sudden high fever, a faint feeling, diarrhea, headache, a rash, and muscle aches.

**Risks of Levonorgestrel**

LNG has been approved for use in contraceptive products for more than three decades including Plan B®, Jadelle®, Norplant®, and Mirena®.

The most common adverse reactions reported in clinical trials of Plan B® were:

- Heavier menstrual bleeding
- Nausea
- Lower abdominal pain
- Fatigue (feeling tired)
- Headache
- Dizziness

The most common adverse reactions reported in clinical trials of an LNG-releasing implant (Jadelle®) were:

- Headache
- Nervousness
- Dizziness
- Nausea
- Changes in menstrual bleeding
- Cervicitis (inflammation of the cervix)
- Vaginal infection
- Vaginal itching
- Vaginal discharge
- Pelvic pain
- Breast pain
- Weight gain
- Acne
- Bleeding at implant insertion site (not applicable to vaginal rings)

The most common adverse reactions reported in clinical trials of a different LNG-releasing implant (Norplant®) were:

- Prolonged, frequent, or irregular bleeding
- Lack of vaginal bleeding (amenorrhea)
- Infrequent bleeding or spotting

The most common adverse reactions in more than 5% of users (more than 5 out of 100 users) for Plan B®, Jadelle®, and the Mirena® IUD are similar and include:

- Uterine/vaginal bleeding alterations including amenorrhea (abnormal absence of menstrual bleeding), menorrhagia (abnormally heavy menstrual bleeding) and intermenstrual bleeding (vaginal bleeding between monthly menstrual periods)
- Abdominal/pelvic pain
- Headache/migraine
- Acne
- Depressed/altered mood
- Breast tenderness/pain
- Vaginal discharge
- Nausea

Other rare and potentially more serious adverse reactions associated with continued LNG use that have been reported are:

- Ectopic pregnancy (a pregnancy outside the uterus)
- Ovarian cysts (fluid-filled sac within the ovary)
- Thrombosis (blood clots)
- Idiopathic intracranial hypertension (increased pressure around the brain, particularly in obese participants)

**Risks of Dapivirine-Levonorgestrel Vaginal Ring**

Based on preliminary results from the first study of the DPV-LNG VR, reported adverse reactions that are possibly related to the VR include:

- Abdominal distension
- Nausea
- Increased appetite
- Headache
- Breast tenderness
- Uterine cramping
- Vaginal discharge
- Vaginal odor
- Vulvovaginal discomfort
- Hot flush

It should be noted that a causal association between the above side effects and the study products has not yet been determined.

**Risks of Discussing HIV and Sexual Behaviors**

You may become embarrassed and/or worried when discussing your sexual practices, ways to protect against HIV and other STIs, and your test results. You may be worried while waiting for your test results. If you have HIV or other infections, learning this could make you worried. Trained study counselors will help you deal with any feelings or questions you have.

**Risks of Blood Draws**

Whenever your blood is drawn, you may have:

- Discomfort
- Feelings of dizziness or faintness
- Bruising, swelling and/or infection

**Risks of Pelvic Exams and Vaginal Fluid Collection**

During pelvic exams and cervical and vaginal fluid collection you may feel discomfort or pressure in your vagina, genital area and/or pelvis. You may also have vaginal bleeding or spotting, which should stop shortly after the examination.

**Risks of Cervical Biopsy Collection**

Cervical biopsies carry the risk of discomfort or pain during the procedure and for a few hours afterwards. You may have mild vaginal spotting (bleeding) for one or two days. Please do not use aspirin (over 81 mg per day) and/or other drugs that are associated with the increased likelihood of bleeding for 72 hours before and after the collection of the cervical biopsies. If you engage in sexual intercourse before the biopsy has healed you may experience some temporary discomfort. If you are sexually active you may also be at increased risk for STIs and HIV acquisition, if exposed. There is a small risk of infection and heavier bleeding.

**Risk of Breach of Confidentiality**

We will make every effort to protect your privacy and confidentiality during the study visits. Your visits will take place in private. However, it is possible that your involvement in the study could become known to others, and that social harms may result (for example, because participants could become known as HIV-positive or at "high risk" for HIV infection). For example, participants could be treated unfairly or discriminated against, or could have problems being accepted by their families, communities, and/or employer(s). Finding out your HIV or STI status could cause depression, suicidal thoughts and/or problems between you and your partner. If you have any problems, study counselors will talk with you and/or your partner to try to help resolve them.

If you are having suicidal thoughts call the study doctor at the telephone number listed on the first page of this form. If you feel in crisis, you can call 911 and/or a Nationwide Suicide Hotline that is answered 24 hours a day with a skilled, trained counselor. One example is the National Suicide Prevention Lifeline at 1-800-273-TALK (8255).

**Sexual Practices, Pregnancy, and Breastfeeding**:

LNG is widely used in different types of hormonal birth control; however, **it is not known whether the study VR containing LNG can prevent pregnancy**. LNG is not recommended for use by women who are pregnant or may be pregnant. We do not know what effect DPV has on pregnancy, including the effect of DPV on the fetuses of women who use the VR when pregnant, or the babies of women who use the VR when breastfeeding. Because of this, anyone who is pregnant or breastfeeding may not join this study. **Participants who join the study must agree to use an acceptable method of contraception** (see Screening Visit for details). Participants who join this study must also agree to be sexually abstinent starting 24 hours prior to the Enrollment Visit and clinical visits where samples are taken and for one week after each cervical biopsy. Participants who join this study will have pregnancy tests while in the study.

If you become pregnant at any time during the study, study staff will refer you to available medical care and other services you may need. The study does not pay for this care. You will not receive (or you will stop using) the study VR and you will exit the study. The outcome of your pregnancy is important to study staff; therefore, your pregnancy will be followed until the results of your pregnancy are known. We may contact you to find out about the health of your pregnancy. If you become pregnant and you deliver a baby from that pregnancy, we will contact you up to approximately one year after your delivery to collect information about the health of your baby.

**If You Become Infected with HIV**

Although a 25 mg DPV VR was recently shown in two large studies to be safe and to help to prevent HIV, **this study is not testing to see if the study VRs prevent HIV infection, and your participation in this study will not cause HIV infection.** The study drugs do not cause HIV. However, there is always a chance that through sexual activity or other activities that you may become HIV-positive. In the unlikely event that you become HIV-positive, study staff will give you counseling and refer you for medical care and other available services. Tests may be performed to see if you have HIV drug resistance. This will allow doctors to know what HIV drugs would be best for the treatment of your type of HIV. If the HIV tests indicate you may be infected with HIV, you will stop using the VR. If HIV infection is confirmed, you will stop your participation in this study. You may be referred to other research studies.

***What are the possible benefits from taking part in this study?***

You will receive HIV/STI risk reduction counseling, HIV and STI testing, physical and pelvic examinations, and routine laboratory testing, including tests to check the overall health of your liver and kidneys. You will be offered free male condoms if you need them. This study cannot provide you with general medical care, but study staff will refer you to other available sources of care as needed. If you test positive for HIV, you will be referred for medical care, counseling, and other services available to you. If you have an STI diagnosed, you will receive medicine or a referral, if needed. Patients in the future may benefit from what we learn in this research study.

***If I agree to take part in this research study, will I be told of any new risks that may be found during the course of the study?***

You will be told of any new information learned during this study that might affect your willingness to stay in the study. For example, if information becomes available that shows that the VR may be causing bad effects, you will be told about this. Additionally, you will be told of any new information about other effective HIV-prevention products as they become available. You will also be told when the study results are available, and how to learn about them.

***May I withdraw, at a future date, my consent for participation in this research study?***

You may withdraw, at any time, your consent for participation in this research study, to include the use and disclosure of your identifiable information for the purposes described above. (Note, however, that if you withdraw your consent for the use and disclosure of your identifiable medical record information for the purposes described above, you will also be withdrawn, in general, from further participation in this research study.) Any identifiable research or medical information recorded for, or resulting from, your participation in this research study prior to the date that you formally withdrew your consent may continue to be used and disclosed by the investigators for the purposes described above.

To formally withdraw your consent for participation in this research study you should provide a written and dated notice of this decision to the principal investigator of this research study at the address listed on the first page of this form.

Your decision to withdraw your consent for participation in this research study will have no effect on your current or future medical care provided by a hospital or affiliated health care provider or your current or future relationship with a health care insurance provider. If you do not want to be in the study or you stop the study at a later time, you will not be penalized or lose any benefits.

***If I agree to take part in this research study, can I be removed from the study without my consent?***

The researchers can ask you to stop participating in the study without your permission for the following reasons:

- The study is cancelled by the US FDA, US NIH, International Partnership for Microbicides (IPM, the nonprofit organization that supplies the VRs), the US Office for Human Research Protections (OHRP), the MTN, the Contraceptive Clinical Trials Network (CCTN), the local or other government or regulatory agency, or the Institutional Review Board. An Institutional Review Board is a committee that watches over the safety and rights of research participants
- The Study Monitoring Committee recommends that the study be stopped early. The Study Monitoring Committee reviews the progress of the study and the kinds of effects that people report while they are participating in the study
- You are found to be infected with HIV
- You become pregnant
- You are found to use recreational injection drugs
- You report the use of PEP for HIV exposure
- You report the use of PrEP for HIV prevention
- Study staff decide that using the VR would be harmful to you, for example, if you have a bad reaction to the study ring
- Other reasons that may prevent you from completing the study successfully, such as inability to consistently keep appointments

If study staff ask you to stop using the VR, you will be asked to complete an interim visit during which time the procedures highlighted to occur at Visit 11 will be completed. Thereafter, you may be asked to continue your regular clinic visit schedule with modified procedures, unless otherwise informed by study staff.

In the event that you are removed from or choose to leave this study, you will be asked to return your VR and complete a final evaluation. If you do not have the VR with you at the time of your contact with staff, staff members will make every effort to assist you in returning the ring as soon as possible. At this visit, you will have the same procedures listed above for Visit 11 (Day 90).

***Will my insurance provider or I be charged for the costs of any procedures performed as part of this research study?***

None of the services and/or procedures (study visits, the study VR, physical and pelvic exams, laboratory tests) you receive during this research study will be billed to you or your health insurance. If you receive a bill or believe that your health insurance has been billed for something that is part of the research study, notify a member of the study staff.

You will be referred for treatment for infections passed through sex, including HIV, for the duration of the study, and this treatment will be billed to you and/or your health insurance. You and/or your health insurance will be charged in the standard manner for services and procedures provided for your routine care. You will need to pay any costs for medical care for problems not related to the use of the study VRs. You will need to pay any costs for any visits to the Emergency Room not authorized by one of the researchers unless immediate medical care is needed.

***Will I be paid if I take part in this research study?***

**[*SITE TO INSERT INFORMATION ABOUT LOCAL REIMBURSEMENT*]:** You will receive ***[SITE TO INSERT AMOUNT $XX]*** for your time, effort, and travel to and from the clinic at each scheduled visit. You will receive ***[SITE TO INSERT AMOUNT $XX]*** for responding to text messages. You will receive ***[SITE TO INSERT AMOUNT $XX]*** for the phone call/contact after ring removal. You may receive ***[SITE TO INSERT AMOUNT $XX]*** for any visits which occur in between your normally scheduled visits. You will be paid at the end of each study visit / monthly for completed study visits / quarterly (every 3 months) for completed study visits. If you do not finish the study, you will only be paid for the visits you completed.

***Who will pay if I am injured as a result of taking part in this study?***

If you believe that the research procedures have resulted in an injury to you, immediately contact the Principal Investigator who is listed on the first page of this form. If you become ill or injured as a result of participation in this study, medical treatment for the adverse reaction or injury will be provided appropriately. The site staff will refer you for ongoing treatment for the injury, if needed. IPM will be responsible for compensation for appropriate medical expenses for treatment of any such illness or injury. An HIV infection that occurs during the course of the trial will not be considered an injury or illness caused by trial participation. The research center or sponsor is not responsible for any loss, injuries and/or damages that are caused by any of the following things:

- Any injury that happens because you used other medicine during the study that you did not tell us about
- Any injury that happens because you did not follow instructions given by the study doctor or nurse
- Any injury that happens because of negligence on your part

To pay these medical expenses, the sponsor will need to know some information about you, like your name, date of birth, and social security number or Medicare Health Insurance Claim Number. This is because the sponsor has to check to see if you receive Medicare and if you do, report the payment it makes to Medicare.

***Who will know about my participation in this research study?***

Any information about you obtained from this research will be kept as confidential (private) as possible. However, it is not possible to guarantee confidentiality. Your personal information may be disclosed if required by law. Records related to your involvement in this research study will be stored in a locked file cabinet at the research site. Your records will be maintained in such a manner that it is not possible to link the research data to your identity. Only the investigators and their research staff have access to the research records. Information from the records will be entered into a computer database. For the computer records, your identity will be indicated by an identification number rather than by your name, and the information linking these identification numbers with your identity will be kept separate from the computer records. Any publication of this study will not use your name or identify you personally.

All HIV testing information will be handled in compliance with the state law on HIV-related confidential information. For visits conducted in person where HIV test results are provided, confidentiality will be maintained by providing the results in a private room. If an HIV test is done at the Day 90/Study Early Termination Visit, you will be given the opportunity to receive results in person, if available. If you decline and prefer to receive results by phone, your identity will first be confirmed before you are given the test results. After you receive the results, you will have the opportunity to have any questions answered, and post-test counseling will be provided.

***As part of this study, we are also requesting your authorization or permission to review your medical records to obtain past and current medical information from hospital and other medical facilities.***

We will obtain information concerning your diagnosis, age, past medical history, diagnostic procedures, and results of any tests that were already done as part of your standard medical evaluations at hospitals. We will use this information to determine whether you meet the conditions for participation in this study, to compare your earlier test results to the findings from this study and, if possible, to use your previous exam results in place of or in addition to some of the exams needed for this study.

The study staff may use your personal information to verify that you are not in any other research studies. This includes studies conducted by other researchers that study staff may know about. The results of any visits that you make to the Emergency Room because of issues related to the study will become part of your medical records at the hospital. Other information gathered during this study will only be kept at the research site as described above.

***Who will have access to identifiable information related to my participation in this research study?***

In addition to the investigators listed on the first page of this authorization (consent) form and their research staff, the following individuals and their representatives or designees will or may have access to identifiable information (which may include your identifiable medical record information) related to your participation in this research study:

- Research Conduct and Compliance Office for the research site
- The sponsors of this research study, the National Institutes of Health (NIH) and the Microbicide Trials Network (MTN)
- The Food and Drug Administration (FDA)
- The Office of Human Research Protection (OHRP)
- Other local, US and international regulatory authorities
- IPM, the organization supplying the study vaginal rings and CCTN, including study monitors
- Health Decisions (a research organization that monitors CCTN sponsored clinical trials for safety and data quality)
- Study staff
- Site Institutional Review Boards or Ethics Committees

***For how long will the investigators be permitted to use and disclose identifiable information related to my participation in this research study?***

The investigators may continue to use and disclose, for the purposes described above, identifiable information (which may include your identifiable medical information) related to your participation in this research study for a minimum of ***[SITE TO INSERT]*** years and for as long (indefinite) as it may take to complete this research study. Your audio recordings, notes and transcripts will be kept for at least two years after the vaginal ring is approved for marketing or two years after all developmental research on the vaginal ring is stopped.

***May I have access to my medical information that results from my participation in this research study?***

In accordance with the Notices of Privacy Practices document that you have been provided, you are permitted access to information (including information resulting from your participation in this research study) contained within your medical records, filed with your health care provider.

***Is my participation in this research study voluntary?***

Your participation in this research study, to include the use and disclosure of your identifiable information for the purposes described above, is completely voluntary.

(Note, however, that if you do not provide your consent for the use and disclosure of your identifiable information for the purposes described above, you will not be allowed, in general, to participate in the research study.) Whether or not you provide your consent for participation in this research study will have no effect on your current or future medical care provided by a hospital or affiliated health care provider or your current or future relationship with a health care insurance provider.

Your doctor may be involved as an investigator in this research study. As both your doctor and a research investigator, s/he is interested both in your medical care and the conduct of this research study. Before agreeing to participate in this research study, or at any time during your study participation, you may discuss your care with another doctor who is not associated with this research study. You are not under any obligation to participate in any research study offered by your doctor.

***[SITE TO INCLUDE/AMEND THE FOLLOWING]:***

**Following study participation in the study, you may be referred to other research studies.**

***What additional confidentiality (privacy) protections are provided by a federal Confidentiality Certificate?***

To help us protect your privacy, we have obtained a Certificate of Confidentiality from the National Institutes of Health. The researchers can use this Certificate to legally refuse to disclose any information that may identify you in any federal, state, or local civil, criminal, administrative, legislative, or other proceedings; for example, if there is a court subpoena. The researchers will use the Certificate to resist any demands for information that would identify you, except as explained below.

The Certificate cannot be used to resist a demand for information from personnel of the United States Government that is used for auditing or evaluation of federally-funded projects or for information that must be disclosed in order to meet the requirements of the federal Food and Drug Administration (FDA).

You should understand that a Certificate of Confidentiality does not prevent you or a member of your family from voluntarily releasing information about yourself or your involvement in this research. If an insurer, employer, or other person obtains your written consent to receive research information, then the researchers may not use the Certificate to withhold that information. Finally, you should also understand that this federal Certificate does not prevent study doctors from taking steps, including reporting to appropriate authorities, to prevent serious harm to yourself or others. If the study doctors learn that you or someone with whom you are involved is in serious danger or potential harm, they will need to inform, as required by state law, the appropriate agencies.

**CLINICALTRIALS.GOV**

A description of this clinical trial will be available on http://www.ClinicalTrials.gov, as required by U.S. Law. This web site will not include information that can identify you. At most, the web site will include a summary of the results. You can search this web site at any time.

**GETTING ANSWERS TO YOUR QUESTIONS OR CONCERNS ABOUT THE STUDY**

You can ask questions about this consent form or the study (before you decide to start the study, at any time during the study, or after completion of the study).  Questions may include:

- Who to contact in the case of a research-related injury or illness
- Payment or compensation for being in the study, if any
- Your responsibilities as a study subject
- Eligibility to participate in the research
- The study doctor’s or study site’s decision to exclude you from participation
- Results of tests and/or procedures
- Other questions, concerns, or complaints

***Contact the study doctor or study staff listed on the first page of this form with any questions, concerns or complaints.***

**GETTING ANSWERS TO YOUR QUESTIONS ABOUT YOUR RIGHTS AS A RESEARCH SUBJECT**

This study has been reviewed by an Institutional Review Board (IRB). This Committee reviewed this study to help ensure that your rights and welfare are protected and that this study is carried out in an ethical manner.

For questions about your rights as a research subject, contact:

- By mail:

Study Subject Adviser

Chesapeake IRB

6940 Columbia Gateway Drive, Suite 110

Columbia, MD 21046

- or call **toll free**:        877-992-4724
- or by **email**:              [adviser@chesapeakeirb.com](mailto:adviser@chesapeakeirb.com)

Please reference the following number when contacting the Study Subject Adviser: Pro00024525.

| **OPTIONAL CONSENT FOR LONG-TERM STORAGE AND FUTURE TESTING OF SPECIMENS**  There might be a small amount of blood, cervicovaginal fluid or cervical tissue left over after we have completed all study related testing. We would like to ask your permission to store these leftover samples and related health information for use in future studies, such as future research to fight HIV and other related diseases. This health information may include personal facts about you such as your race, ethnicity, sex, medical conditions and your age range.  If you agree, your samples and related health data will be stored safely and securely at facilities that are designed so that only approved researchers will have access to the samples. Some employees of the facilities will need to have access to your samples to store them and keep track of where they are, but these people will not have information that directly identifies you.  The type of testing planned for your leftover specimens is not yet known. However, samples may be used by the MTN Laboratory Center to complete additional quality assurance testing, ensuring that the tests work correctly and supply accurate data. No genetic testing on either a limited set or the full set of genes is planned for leftover specimens that are stored for the purposes of future research. It is important that you know that any future testing or studies planned for these specimens must be approved by an Institutional Review Board before they can be done.  **You can still enroll in this study if you decide not to have leftover samples stored for future studies. If you do not want the leftover samples stored, we will destroy them. You can withdraw your consent for the storage and future testing of specimens at any time by providing your request in writing to the principal investigator of this research study at the address listed on the first page of this form. However, researchers will not be able to destroy samples or information from research that is already underway.**  _______________   \| Initials and Date \| I **DO agree** to allow my biological specimens and health data to be stored and used in future research studies. \| \| --- \| --- \|   _______________   \| Initials and Date \| I **DO NOT** agree to allow my biological specimens and health data to be stored and used in future research studies. \| \| --- \| --- \| |
| --- | --- | --- | --- | --- |

**VOLUNTARY CONSENT:** All of the above has been explained to me and all of my current questions have been answered. I agree that the data collected during this study can be processed in a protected computerized system by a member of the study team. I understand that I am encouraged to ask questions about any aspect of this research study during the course of this study, and that such future questions will be answered by the researchers listed on the first page of this form. Any questions which I have about my rights as a research participant will be answered by the IRB office. By signing this form, I consent to participate in this research study and provide my authorization to share my medical records with the study team. A copy of this consent form will be given to me.

_________________________ _________________________

Participant’s Printed Name Participant’s Signature Date and Time

**CERTIFICATION OF INFORMED CONSENT:** I certify that I have explained the nature and purpose of this research study to the above-named individual, and I have discussed the potential benefits and possible risks of study participation. Any questions the individual has about this study have been answered, and we will always be available to address future questions as they arise. I further certify that no research component of this protocol was begun until after this consent form was signed.

________________________ ________________________ ____________

Study Staff Conducting Study Staff Signature Date and Time

Consent Discussion (print)
