# Supplementary material for: Phase 1 randomized trials to assess safety, pharmacokinetics, and vaginal bleeding associated with use of extended duration dapivirine and levonorgestrel vaginal rings
Source: PLoS One. 2024 Jun 5;19(6):e0304552. doi: 10.1371/journal.pone.0304552 (PMC11152307; doi:10.1371/journal.pone.0304552)
Supplement: S2 File — (PDF) [file pone.0304552.s004.pdf]

MTN-030/IPM 041

A Phase 1, Randomized, Double-Blind Pharmacokinetic and Safety Study of Dapivirine/Levonorgestrel Vaginal Rings

Microbicide Trials Network

Funding Agencies:
Division of AIDS, US National Institute of Allergy and Infectious Diseases
US *Eunice Kennedy Shriver* National Institute of
Child Health and Human Development
US National Institute of Mental Health
US National Institutes of Health

Grant Number:
5UM1 AI068633-07

DAIDS Protocol ID: 12037

IND Sponsor:
International Partnership for Microbicides

IND #: 126907

Protocol Chair:
Sharon L. Achilles, MD, PhD

Protocol Co-Chair:
Beatrice A. Chen, MD, MPH

Version 1.0

April 6, 2016

MTN-030/IPM 041

A Phase 1, Randomized, Double-Blind Pharmacokinetic and Safety Study of Dapivirine/Levonorgestrel Vaginal Rings

TABLE OF CONTENTS

LIST OF ABBREVIATIONS AND ACRONYMS vi
PROTOCOL TEAM ROSTER ix
INVESTIGATOR SIGNATURE FORM xvii
PROTOCOL SUMMARY xviii
1 KEY ROLES 1
1.1 Protocol Identification 1
1.2 Funding Agencies, Sponsor and Monitor Identification 1
1.3 Medical Officer 2
1.4 Clinical Laboratories 2
1.5 Data Center 2
1.6 Study Implementation 2
2 INTRODUCTION 3
2.1 Microbicides, Human Immunodeficiency Virus Prevention, and Contraception 3
2.2 Dapivirine 4
2.3 Levonorgestrel 6
2.4 Nonclinical Studies of Dapivirine 6
2.5 Clinical Studies of Dapivirine 8
2.6 Safety of Levonorgestrel 20
2.7 Nonclinical Studies of Levonorgestrel in Combination with Dapivirine 23
2.8 Rationale for Study Design and Features 25
3 OBJECTIVES 26
3.1 Primary Objectives 26
3.2 Secondary Objective 26
3.3 Exploratory Objectives 27
4 STUDY DESIGN 27
4.1 Identification of Study Design 27
4.2 Primary Endpoints: 27
4.3 Secondary Endpoint: 28
4.4 Exploratory Endpoints: 28
4.5 Description of Study Population 28
4.6 Time to Complete Accrual 28
4.7 Study Groups 28
4.8 Expected Duration of Participation 28
4.9 Sites 29
5 STUDY POPULATION 29
5.1 Selection of the Study Population 29
5.2 Inclusion Criteria 29
5.3 Exclusion Criteria 31
5.4 Co-enrollment Guidelines 33
6 STUDY PRODUCT 33

6.1 Regimen 33
6.2 Administration 34
6.3 Study Product Formulation 34
6.4 Supply and Accountability 35
6.5 VR Use Instructions 35
6.6 Concomitant Medications 36
6.7 Use of Intravaginal Medications/Products and Practices 36
7 STUDY PROCEDURES 37
7.1 Pre-screening 37
7.2 Visit 1 - Screening Visit 37
7.3 Visit 2 - Enrollment Visit (Day 0) 39
7.4 Follow-up Visits 40
7.5 Follow-up Procedures for Participants Who Permanently Discontinue Study Product 43
7.6 Interim Visits 45
7.7 Pharmacokinetics 45
7.8 Behavioral Assessments 46
7.9 Clinical Evaluations and Procedures 47
7.10 Laboratory Evaluations 47
7.11 Specimen Management 48
7.12 DAIDS Laboratory Oversight 49
7.13 Biohazard Containment 49
8 ASSESSMENT OF SAFETY 49
8.1 Safety Monitoring 49
8.2 Clinical Data and Safety Review 50
8.3 Adverse Events Definitions and Reporting Requirements 51
8.4 Adverse Event Reporting Requirements 53
8.5 Pregnancy and Pregnancy Outcomes 54
8.6 Regulatory Requirements 54
8.7 Social Harms Reporting 54
9 CLINICAL MANAGEMENT 54
9.1 Grading System 54
9.2 Dose Modification Instructions 55
9.3 General Criteria for Permanent Discontinuation of Study Product 55
9.4 Response to Adverse Events 55
9.5 Sexually Transmitted Infection/Reproductive Tract Infection 56
9.6 Management of Specific Genital Events 56
9.7 HIV-1 Infection 56
9.8 Pregnancy 57
9.9 Criteria for Early Termination of Study Participation 57
10 STATISTICAL CONSIDERATIONS 58
10.1 Overview and Summary of Design 58
10.2 Study Endpoints 58
10.3 Primary Study Hypotheses 58
10.4 Sample Size and Power Calculations 59
10.5 Participant Accrual, Follow-up, Retention, and Replacement 60

10.6 Randomization 60
10.7 Blinding 60
10.8 Data and Safety Monitoring and Analysis 61
11 DATA HANDLING AND RECORDKEEPING 62
11.1 Data Management Responsibilities 62
11.2 Source Documents and Access to Source Data/Documents 63
11.3 Quality Control and Quality Assurance 63
12 CLINICAL SITE MONITORING 63
13 HUMAN SUBJECTS PROTECTIONS 64
13.1 Institutional Review Boards/Ethics Committees 64
13.2 Protocol Registration 65
13.3 Study Coordination 65
13.4 Risk Benefit Statement 66
13.5 Informed Consent Process 68
13.6 Participant Confidentiality 69
13.7 Special Populations 70
13.8 Compensation 70
13.9 Communicable Disease Reporting 70
13.10 Access to HIV-related Care 71
13.11 Care for Participants Identified as HIV-Positive 71
13.12 Study Discontinuation 71
14 PUBLICATION POLICY 71
15 APPENDICES 72
APPENDIX I: SCHEDULE OF STUDY VISITS AND EVALUATIONS 72
APPENDIX II: ALGORITHM FOR HIV TESTING FOR SCREENING AND ENROLLED PARTICIPANTS 74
APPENDIX III: SAMPLE INFORMED CONSENT FORM (SCREENING, ENROLLMENT, LONG-TERM STORAGE AND FUTURE TESTING) 75

Table of Figures

Figure 1: Study Visit Schedule 19
Figure 2: Study Visit Schedule 37

Table 1:Treatment-Emergent Adverse Events (≥ 5% for Either Treatment Group) Across Completed Dapivirine Vaginal Gel 4759 Trials 14
Table 2: Treatment-Emergent Adverse Events (≥ 5% for either Treatment Group) Across Completed Dapivirine Vaginal Ring-004 Trials 16
Table 3: Pharmacokinetics of Dapivirine and Levonorgestrel in Plasma and Vaginal Fluid Following Vaginal Administration to Sheep 24
Table 4: Study Regimen 33
Table 5: Retrieval of VR 35
Table 6: Visit 1 - Screening Visit 38
Table 7: Visit 2 - Enrollment Visit (Day 0) 39
Table 8: Visits 3-5: Day 1, Day 2, Day 3 Study Follow-up Visits 40
Table 9: Visit 6 – Day 7 41
Table 10: Visit 7 – Day 14: PUEV/Early Termination Visit 42

Table 11: Visit 8 and 9 – Day 15 and 16 43
Table 12: PK Specimen Collection Schedule 45
Table 13: Analysis of PK Event Frequency 59
Table 14: Analysis of Safety Event Frequency 59
Table 15: Precision of Exact 2-sided 95% CIs for Observed Event Rates 59

MTN-030/IPM 041

A Phase 1, Randomized, Double-Blind Pharmacokinetic and Safety Study of Dapivirine/Levonorgestrel Vaginal Rings

LIST OF ABBREVIATIONS AND ACRONYMS

AE	adverse event
ALT	alanine transaminase
ART	antiretroviral therapy
ARV	antiretroviral
ASPIRE	A Study to Prevent Infection with a Ring for Extended Use
AST	aspartate aminotransferase
AUC	area under the curve
b.i.d.	*bis in die* (twice daily)
BRWG	Behavioral Research Working Group
BSWG	Biomedical Science Working Group
BV	bacterial vaginosis
CBC	complete blood count
CDC	Centers for Disease Control and Prevention
CFR	Code of Federal Regulations
C_{max}	maximum concentration
CRF	case report form
CROI	Conference on Retroviruses and Opportunistic Infections
CRMS	Clinical Research Management System
CRS	clinical research site
CT	*Chlamydia trachomatis,* chlamydia
CTA	Clinical Trial Agreement
CVF	cervicovaginal fluid
CWG	Community Working Group
CYP	cytochrome P450
DAERS	DAIDS Adverse Experience Reporting System
DAIDS	Division of AIDS
DAPY	di-amino-pyrimidine
DDU	dideoxyuridine
DLV	delavirdine
DMPA	depot medroxyprogesterone acetate
DNA	deoxyribonucleic acid
DPV	dapivirine
EAE	expedited adverse event
EC_{50}	median effective concentration
EFV	efavirenz
ENR	Enrollment
FDA	(US) Food and Drug Administration
FHCRC	Fred Hutchinson Cancer Research Center
FSH	follicle-stimulating hormone
g	grams
GC	*Neisseria gonorrhoeae,* gonorrhea
GCP	Good Clinical Practices
GMP	Good Manufacturing Practices
hCG	human chorionic gonadotropin
HEC	hydroxyethylcellulose
HHS	US Department of Health and Human Services

HIV	Human Immunodeficiency Virus
hu-PBL	human peripheral blood lymphocytes
IATA	International Air Transport Association
IB	Investigator's Brochure
ICF	informed consent forms
ICH	International Conference on Harmonisation
IND	Investigational New Drug
IoR	Investigator of Record
IPM	International Partnership for Microbicides
IRB	Institutional Review Board
IUD	intrauterine device
IUS	intrauterine system
kg	kilogram
KOH	potassium hydroxide
LC	MTN Laboratory Center
LDMS	Laboratory Data Management System
LLOQ	lower limit of quantification
LNG	levonorgestrel
LOC	MTN Leadership and Operations Center
μg	microgram
μM	micromole
m	meter
mg	milligram
mL	milliliter
mm	millimeter
MO	Medical Officer
MTD	maximum tolerated dose
MTN	Microbicide Trials Network
NAAT	nucleic acid amplification test
ng	nanogram
NIAID	National Institute of Allergy and Infectious Diseases
NICHD	*Eunice Kennedy Shriver* National Institute of Child Health and Human Development
NIH	National Institutes of Health
NIMH	National Institute of Mental Health
nM	nanomole
NNRTI	non-nucleoside reverse transcriptase inhibitor
NOAEL	no-observed-adverse-effect-level
NVP	nevirapine
OHRP	Office for Human Research Protections
PD	pharmacodynamics
PEP	post-exposure prophylaxis
pg	picogram
PK	pharmacokinetics
PoR	Pharmacist of Record
PPD	Pharmaceutical Product Development
PrEP	pre-exposure prophylaxis
PRO	Protocol Registration Office
PSP	Prevention Sciences Program
PSRT	Protocol Safety Review Team
PTID	participant identification
PUEV	product use end visit
PVI	penile-vaginal intercourse
RBA	relative binding affinity
RE	Regulatory Entity
RNA	ribonucleic acid

RSC	Regulatory Support Center
RT	reverse transcriptase
RTI	reproductive tract infection
SAE	serious adverse event
SCHARP	Statistical Center for HIV/AIDS Research & Prevention
SCID	severe combined immunodeficiency
SCR	Screening
SDMC	Statistical Data Management Center
SE	silicone elastomer
SHBG	sex hormone-binding globulin
SMC	Study Monitoring Committee
SMS	short message service
SOP	standard operating procedure
SSP	study specific procedures
STI	sexually transmitted infection
SUSARs	suspected, unexpected serious adverse reactions
TEAE	treatment-emergent adverse events
UA	urinalysis
UNAIDS	United Nations Programme on HIV/AIDS
UPMC	University of Pittsburgh Medical Center
USA	United States of America
USP	U.S. Pharmacopeial Convention
UTI	urinary tract infection
VIDD	Vaccine and Infectious Disease Division
VR	intravaginal ring
WHO	World Health Organization
w/w	weight/weight

MTN-030/IPM 041

A Phase 1, Randomized, Double-Blind Pharmacokinetic and Safety Study of Dapivirine/Levonorgestrel Vaginal Rings

PROTOCOL TEAM ROSTER

Protocol Chair

Sharon L. Achilles, MD, PhD, FACOG
Magee-Womens Hospital of UPMC
300 Halket Street
Pittsburgh, PA 15213 USA
Phone: 412-641-1403
Fax: 412-641-1133
Email: achisx@upmc.edu

Protocol Co-Chair

Beatrice A. Chen, MD, MPH
Magee-Womens Hospital of UPMC
300 Halket Street
Pittsburgh, PA 15213 USA
Phone: 412-641-1403
Fax: 412-641-1133
Email: chenba@upmc.edu

Site Investigators

Beatrice A. Chen, MD, MPH
Protocol Co-Chair/Site Investigator
Magee-Womens Hospital of UPMC
300 Halket Street
Pittsburgh, PA 15213 USA
Phone: 412-641-1403
Fax: 412-641-1133
Email: chenba@upmc.edu

Craig Hoesley, MD
Site Investigator
University of Alabama at Birmingham
1530 3rd Avenue South, VH 201
Birmingham, AL 35294 USA
Phone: 205-934-3365
Fax: 205-975-3232
Email: choesley@uab.edu

US National Institutes of Health (NIH)

Roberta Black, PhD
Microbicide Research Branch Chief
National Institute of Allergy and Infectious Diseases (NIAID), Division of AIDS (DAIDS)
5601 Fishers Lane, Room 8B62, MSC 9831
Rockville, MD 20852 USA
Phone: 301-496-8199
Fax: 301-402-3684
Email: rblack@niaid.nih.gov

Nahida Chakhtoura, MD, MsGH
Maternal and Pediatric Infectious Disease Branch
Eunice Kennedy Shriver National Institute of Child Health and Human Development (NICHD)
National Institutes of Health (NIH)
6100 Executive Blvd., Room 4B11
Rockville, MD 20852
Phone: 301-594-1968
Fax: 301-496-8678
Email: nahida.chakhtoura@nih.gov

Naana Cleland, MHCA
Health Specialist, Clinical Microbicide Research Branch (CMRB)
Prevention Sciences Program (PSP) DAIDS, NIAID
National Institutes of Health (NIH) - U.S. Department of Health and Human Services (HHS)
5601 Fishers Lane, Room 8B27, MSC 9831
Rockville, MD 20852 USA
Phone: 240-292-4779
Email: clelandn@niaid.nih.gov

Jeanna Piper, MD
DAIDS Senior Medical Officer
NIAID, DAIDS
5601 Fishers Lane, Room 8B68, MSC 9831
Rockville, MD 20852 USA
Phone: 240-292-4798
Fax: 301-402-3684
Email: piperj@niaid.nih.gov

Dianne M. Rausch, PhD
Director, Division of AIDS Research
NIMH
5601 Fishers Lane, Room 8D20, MSC 9831
Rockville, MD 20852 USA
Phone: 240-627-3874
Fax: 240-627-3467
Email: drausch@mail.nih.gov

MTN Leadership and Operations Center (LOC) - Pitt

Katherine Bunge, MD
Protocol Safety Physician
Magee-Womens Hospital of UPMC
300 Halket Street
Pittsburgh, PA 15213 USA
Phone: 412-641-3464
Fax: 412-641-1133
Email: kbunge@mail.magee.edu

Ellen Conser, MA
Protocol & Regulatory Specialist
Microbicide Trials Network
204 Craft Avenue
Pittsburgh, PA 15213 USA
Phone: 412-641-2282
Fax: 412-641-6170
Email: consere@mwri.magee.edu

Beth Galaska, MID
Protocol Development Manager
Microbicide Trials Network
204 Craft Avenue
Pittsburgh, PA 15213 USA
Phone: 412-641-5579
Fax: 412-641-6170
Email: galaskab@upmc.edu

Sharon Hillier, PhD
Co-Principal Investigator
Microbicide Trials Network
204 Craft Avenue
Pittsburgh, PA 15213 USA
Phone: 412-641-8933
Fax: 412-641-6170
Email: shillier@mail.magee.edu

Ken Ho, MD
Safety Physician
UPMC, Keystone Building, Suite 533
3520 Fifth Avenue
Pittsburgh, PA 15213 USA
Phone: 412-383-7178
Fax: 412-383-2900
Email: hok2@upmc.edu

Cindy Jacobson, PharmD
Director of Pharmacy Affairs
Microbicide Trials Network
204 Craft Avenue
Pittsburgh, PA 15213 USA
Phone: 412-641-8913
Fax: 412-641-6170
Email: cjacobson@mail.magee.edu

Ian McGowan, MBChB, MD, DPhil, FRCP
Co-Principal Investigator
Microbicide Trials Network
204 Craft Avenue
Pittsburgh, PA 15213 USA
Phone: 412-641-8999
Fax: 412-641-6170
Email: imcgowan@pitt.edu

Sharon A. Riddler, MD, MPH
Protocol Physician
UPMC, Keystone Building, Suite 510
3520 Fifth Avenue
Pittsburgh, PA 15213 USA
Phone: 412-383-1741 or 412-383-1675
Fax: 412-383-2900
Email: riddler@pitt.edu

Devika Singh, MD, MPH
Protocol Safety Physician
Box 359927, Dept. of Global Health, ICRC
325 Ninth Ave.
Seattle WA 98104 USA
Phone: 206-744-8311
Fax: 206-520-3831
Email: devika@mtnstopshiv.org

MTN Laboratory Center (LC)

May Beamer, BS
Laboratory Manager/Supervisor
Microbicide Trials Network
204 Craft Avenue
Pittsburgh, PA 15213 USA
Phone: 412-641-6026
Fax: 412-641-6170
Email: mbeamer@mwri.magee.edu

Charlene S. Dezzutti, PhD
LC Director
Microbicide Trials Network
204 Craft Avenue
Pittsburgh, PA 15213 USA
Phone: 412-641-3642
Fax: 412-641-6170
Email: dezzuttics@upmc.edu

Craig Hendrix, MD
Pharmacology LC Principal Investigator
Johns Hopkins University
600 North Wolfe Street, Harvey 502
Baltimore, MD 21287 USA
Phone: 410-955-9707
Fax: 410-955-9708
Email: cwhendrix@jhmi.edu

Samuel Poloyac, PharmD, PhD
Core Director
University of Pittsburgh, School of Pharmacy
Small Molecular Biomarker Core, 807 Salk Hall
3501 Terrace Street
Pittsburgh, PA 15261 USA
Phone: 412-624-4595
Email: poloyac@pitt.edu

Lorna Rabe, BS, M(ASCP)
Laboratory Manager/Supervisor
Microbicide Trials Network
204 Craft Avenue
Pittsburgh, PA 15213 USA
Phone: 412-641-6042
Fax: 412-641-6170
Email: lrabe@mwri.magee.edu

Jennifer Robinson, MD, MPH, FACOG
Pharmacology LC Representative
Johns Hopkins University
600 North Wolfe Street, Harvey 502
Baltimore, MD 21287 USA
Phone: 410-550-7202
Fax: 410-550-0196
Email: jrobin87@jhmi.edu

MTN LOC – FHI 360

Kat Calabrese, MPH
Clinical Research Manager
FHI 360
359 Blackwell St., Suite 200
Durham, NC 27701 USA
Phone: 919-544-7040 Ext. 11306
Fax: 919-544-7261
Email: krichards@fhi360.org

Lisa Levy, MPH
Sr. Clinical Research Manager
FHI 360
1825 Connecticut Avenue, NW
Washington, DC 20009 USA
Phone: 202-884-8480
Fax: 202-884-8844
Email: llevy@fhi360.org

Rhonda White, RH Ed
Community Program Manager
FHI 360
359 Blackwell St., Suite 200
Durham, NC 27701 USA
Phone: 919-544-7040, Ext. 11515
Fax: 919-544-0207
Email: rwhite@fhi360.org

MTN Statistical Data Management Center (SDMC)

Karen Liu, M.S.
Statistical Research Associate
VIDD – SCHARP
1100 Fairview Avenue North, M2-C200
PO Box 19024
Seattle, WA 98109-1024 USA
Phone: 206-667-2721
Fax: 206-667-4378
Email: cliu2@scharp.org

Melissa Peda, MPA
SDMC Project Manager
FHCRC - SCHARP
1100 Fairview Avenue North, E3-129
PO Box 19024
Seattle, WA 98109-1024 USA
Phone: 206-667-7672
Fax: 206-667-4812
Email: mapeda@scharp.org

Barbra Richardson, PhD
Faculty Statistician
FHCRC-SCHARP
1100 Fairview Ave. North, M2-C200
PO Box 19024
Seattle, WA 98109-1024 USA
Phone: 206-667-7788
Fax: 206-667-4812
Email: barbrar@uw.edu

MTN Working Groups

Barbara S. Mensch, PhD
Behavioral Research Working Group (BRWG) Representative
Population Council
1 Dag Hammarskjold Plaza
New York, NY 10017 USA
Phone: 212-339-0640
Fax: 212-755-6052
Email: bmensch@popcouncil.org

Barbara Shacklett, PhD
Biomedical Science Working Group (BSWG) Representative
UC Davis School of Medicine
3327 Tupper Hall, 1 Shields Ave.
Davis, CA 95616 USA
Phone: 530-752-6785
Fax: 530-752-8692
Email: blshacklett@ucdavis.edu

Renee Weinman, BS, CCRP
MTN Community Working Group (CWG) Representative
University of Pittsburgh
Falk Medical Bldg. 7th floor
3601 Fifth Avenue
Pittsburgh, Pa 15213
Phone: 412-647-0322
Email: weinmandr@upmc.edu

MTN-030/IPM 041

A Phase 1, Randomized, Double-Blind Pharmacokinetic and Safety Study of Dapivirine/Levonorgestrel Vaginal Rings

INVESTIGATOR SIGNATURE FORM

Version 1.0

April 6, 2016

A Study of the Microbicide Trials Network

Funded by:

Division of AIDS, US National Institute of Allergy and Infectious Diseases
US *Eunice Kennedy Shriver* National Institute of Child Health and Human Development
US National Institute of Mental Health
US National Institutes of Health

IND Holder:

International Partnership for Microbicides (IPM)

I, the Investigator of Record, agree to conduct this study in full accordance with the provisions of this protocol. I will comply with all requirements regarding the obligations of investigators as outlined in the Statement of Investigator (Form FDA 1572), which I have also signed. I agree to maintain all study documentation for at least two years following the date of marketing approval for the study product for the indication in which it was studied. If no marketing application is filed, or if the application is not approved, the records will be retained for two years after the investigation is discontinued and the US Food and Drug Administration is notified. Publication of the results of this study will be governed by MTN policies. Any presentation, abstract, or manuscript will be submitted to the MTN Manuscript Review Committee, DAIDS, IPM and other entities for review prior to submission, as required by the MTN Publication Policy.

I have read and understand the information in the Investigator's Brochure(s), including the potential risks and side effects of the products under investigation, and will ensure that all associates, colleagues, and employees assisting in the conduct of the study are informed about the obligations incurred by their contribution to the study.

____
Name of Investigator of Record

______________________________ ______________________________
Signature of Investigator of Record Date

MTN-030/IPM 041

A Phase 1, Randomized, Double-Blind Pharmacokinetic and Safety Study of Dapivirine/Levonorgestrel Vaginal Rings

PROTOCOL SUMMARY

**Short Title:**	PK and Safety Study of Vaginal Rings Containing Dapivirine and Levonorgestrel
**Clinical Phase:**	Phase 1
**IND Sponsor:**	IPM
**Protocol Chair:**	Sharon L. Achilles, MD, PhD, FACOG
**Protocol Co-Chair:**	Beatrice A. Chen, MD, MPH
**Sample Size:**	Approximately 36 participants
**Study Population:**	Healthy, HIV-uninfected females, 18-45 (inclusive) years old
**Study Sites:**	US sites selected by the MTN Executive Committee
**Study Design:**	Phase 1, three-arm, double-blind, multi-site, randomized (1:1:1) trial
**Study Duration:**	Accrual will require approximately 8-10 months. Each enrolled participant will be followed for approximately 16 days.
**Study Products:**	Three silicone elastomer intravaginal rings (VRs) containing the active ingredient dapivirine (DPV), or a combination of the active ingredients DPV and levonorgestrel (LNG), formulated with different dose strengths:<br>1. 200 mg of DPV (Ring-104)<br>2. 200 mg of DPV + 32 mg LNG (Ring-101)<br>3. 200 mg of DPV + 320 mg LNG (Ring-102)
**Study Regimen:**	Participants will be randomized to the study products in a 1:1:1 ratio. Participants will insert one VR to be used for a period of approximately 14 days

Figure 1: Study Visit Schedule

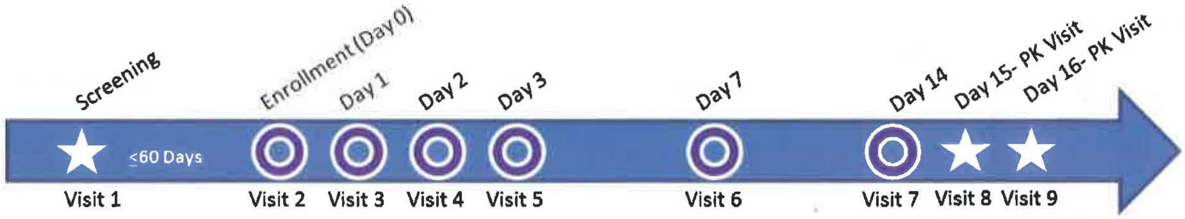

Primary Objectives:

Pharmacokinetics

- To characterize the local and systemic pharmacokinetics of one dapivirine vaginal ring formulation and two dapivirine-levonorgestrel vaginal ring formulations used continuously for 14 days

Safety

- To evaluate the safety of one dapivirine vaginal ring formulation and two dapivirine-levonorgestrel vaginal ring formulations used continuously for 14 days

Primary Endpoints:

Pharmacokinetics

- Dapivirine and levonorgestrel concentrations in blood
- Dapivirine and levonorgestrel concentrations in vaginal fluid

Safety

- Grade 2 or higher genitourinary adverse event as defined by the Division of AIDS (DAIDS) Table for Grading the Severity of Adult and Pediatric Adverse Events, Version 2.0, November 2014, and/or Addendum 1 (Female Genital [Dated November 2007] Grading Table for Use in Microbicide Studies)
- Grade 3 or higher adverse event as defined by the Division of AIDS (DAIDS) Table for Grading the Severity of Adult and Pediatric Adverse Events, Version 2.0, November 2014

Secondary Objective:

Bleeding

- To describe vaginal bleeding experienced during study participation

Secondary Endpoint:

Bleeding

- Self-reported vaginal bleeding

Exploratory Objectives:

Acceptability

- To assess the early acceptability of one dapivirine vaginal ring formulation and two dapivirine-levonorgestrel vaginal ring formulations

Adherence

- To evaluate participant adherence to one DPV vaginal ring formulation or two dapivirine-levonorgestrel vaginal ring formulations

Vaginal Microenvironment

- To describe the genital microenvironment in HIV-uninfected women during 14 days of continuous study product use

Exploratory Endpoints:

Acceptability

- Self-reported attitudes about ring attributes including single vs. dual-purpose indication and willingness to use this study product in the future.

Adherence

- Frequency of study vaginal ring removal/expulsions (voluntary and involuntary) and duration without the vaginal ring *in situ*
- Drug pharmacokinetic levels
- Residual drug levels in returned vaginal rings

Vaginal Microenvironment

- Changes in microflora

1 KEY ROLES

1.1 Protocol Identification

Protocol Title:	A Phase 1, Randomized, Double-Blind Pharmacokinetic and Safety Study of Dapivirine/Levonorgestrel Vaginal Rings
Protocol Number:	MTN-030/IPM 041
Short Title:	PK and Safety Study of Vaginal Rings Containing Dapivirine and Levonorgestrel
Date:	April 6, 2016

1.2 Funding Agencies, Sponsor and Monitor Identification

Funding Agencies:	US Division of AIDS (DAIDS)/National Institute of Allergy and Infectious Diseases (NIAID)<br>National Institutes of Health (NIH)<br>5601 Fishers Lane<br>Rockville, MD 20852 USA<br><br>US National Institute of Mental Health (NIMH)<br>6001 Executive Boulevard<br>Rockville, MD 20852 USA<br><br>US *Eunice Kennedy Shriver* National Institute of Child Health and Human Development (NICHD)<br>Maternal and Pediatric Infectious Diseases (MPID) Branch<br>6100 Executive Boulevard<br>Rockville, MD 20852 USA
IND Sponsor:	International Partnership for Microbicides (IPM)<br>8401 Colesville Rd., Suite 200<br>Silver Spring, MD 20910 USA
Monitor:	Pharmaceutical Product Development (PPD), Inc.<br>929 North Front St.<br>Wilmington, NC 28401-3331 USA

1.3 Medical Officer

Medical Officer: Jeanna Piper, MD
DAIDS/NIAID
5601 Fishers Lane, Room 8B68, MSC 9831
Rockville, MD 20852 USA

1.4 Clinical Laboratories

Laboratory Center: MTN Laboratory Center (LC)
204 Craft Avenue
Pittsburgh, PA 15213 USA

Pharmacology: MTN Pharmacology LC
600 N. Wolfe Street, Osler 527
Johns Hopkins University
Baltimore, MD 21287 USA

1.5 Data Center

Data Center: MTN Statistical Data and Management Center (SDMC)
Statistical Center for HIV/AIDS Research & Prevention (SCHARP)/Fred Hutchinson Cancer Research Center (FHCRC)
1100 Fairview Avenue N., LE-400
PO Box 19024
Seattle, WA 98109-1024 USA

1.6 Study Implementation

Study Implementation: MTN LOC - FHI 360
359 Blackwell Street, Suite 200
PO Box 21059
Durham, NC 27701 USA

2 INTRODUCTION

2.1 Microbicides, Human Immunodeficiency Virus Prevention, and Contraception

In 2014, 2 million people were newly infected and 1.2 million lost their lives to Human Immunodeficiency Virus (HIV)-related causes.[1] Every 60 seconds, a young woman is infected with HIV.[2] Given the high rates of HIV infection among women, female controlled prevention options remain a global priority. Women and girls continue to be affected disproportionately by HIV in sub-Saharan Africa, where women account for approximately 60% of people living with HIV. The ongoing development of safe and effective HIV prevention technologies that can be made easily accessible to developing countries remains a public health priority.

Unprotected heterosexual intercourse is currently the leading mode of HIV acquisition among women. Correct and consistent use of latex condoms is one proven method of preventing both pregnancy and HIV acquisition; however, condoms are widely regarded as an inadequate prevention option for women because many women are unable to negotiate condom use with their partners. So, the most widely available HIV prevention method requires the consent of the male partner. Thus, developing HIV prevention options that women can use independent of male partner consent remains a global concern. Vaginal microbicides, which are self-initiated and controlled, offer women a critically needed biomedical prevention tool that will complement existing HIV prevention strategies as well as future products being developed.

With successful proof-of-concept that antiretroviral (ARV)-based microbicides reduce the risk of HIV-1 acquisition,[3, 4] confirmatory work and further trials involving different ARV compounds, various formulations, and different dosing strategies are required to provide options to end users and to improve upon the level of product effectiveness.

Globally, nearly half of pregnancies (100+ million per year) are unintended.[5, 6] Many highly effective contraceptives have been available for decades. Low utilization rates and high discontinuation rates remain problems due to factors including inconvenience, cost, inaccessibility, and a constrained ability for women in many developing nations to fully participate in sexual and reproductive decision-making. For women, similar factors fuel both unintended pregnancies and the acquisition of HIV: lack of education, malnutrition, poverty and oppression of women.[7] Because 99% of pregnancy-related maternal deaths occur in developing nations,[5, 6, 8] the need for highly acceptable, effective, affordable, dual-purpose contraceptive and HIV prevention options seems clear.

For either a contraceptive or a microbicide to be effective, it is essential that it is used correctly and consistently, and is also acceptable to the user. In addition, a product used independently of sex could be more convenient for women and provide long-term protection during anticipated and unanticipated sexual intercourse. Higher adherence

to a product may translate into higher effectiveness of the product, whether to prevent pregnancy or HIV acquisition. It is likely that products that can be applied less frequently or products that can remain in situ for an extended duration will be more acceptable and will achieve better adherence. Vaginal rings (VRs) that need to be replaced monthly or less frequently may have benefits over dosage forms that need to be used more frequently.

Multiple clinical trials have evaluated the safety of dapivirine (DPV) in VRs,[9] aqueous gels,[10, 11] quick-dissolve vaginal films[10] and in an oral formulation[12, 13]. These clinical trials support the favorable safety profile and tolerability of DPV in general and specifically in vaginal delivery formulations. The safety and efficacy of the DPV-only 25 mg VR (Ring-004) replaced monthly were recently tested in the MTN-020 (ASPIRE) and the IPM 027 (The Ring Study), and results were reported in February 2016. Both trials demonstrated the safety of the VR and showed a protective effect against HIV.[14 15] In ASPIRE, women over the age of 25 were most protected against HIV. The DPV matrix VR proposed for use in MTN-030/IPM 041 (Ring-104) contains 200 mg of active drug.

MTN-030/IPM 041 is a collaborative project between the International Partnership for Microbicides (IPM) and the Microbicide Trials Network (MTN) to evaluate the pharmacokinetics (PK) and safety of DPV-LNG VRs of varying LNG dose strengths in a Phase 1 trial enrolling women aged 18-45.

2.2 Dapivirine

2.2.1 Description

DPV (also known as TMC-120), a non-nucleoside reverse-transcriptase inhibitor (NNRTI), is a substituted di-amino-pyrimidine (DAPY) derivative with potent antiviral activity against HIV-1. DPV is chemically described as 4-[[4-[(2,4,6-trimethylphenyl)amino]-2-pyrimidinyl]amino]benzonitrile.[16]

DPV was originally developed by Janssen Research and Development (formerly Tibotec Pharmaceuticals Ltd.), a subsidiary of Johnson & Johnson, as an oral ARV compound for treatment of HIV/AIDS and was tested in Phase 1 and 2 clinical trials in more than 200 participants.[17] DPV is also a promising topical microbicide candidate due to its proven *in vitro* and *in vivo* efficacy and favorable safety profile as well as its physical and chemical properties.[9, 11, 16, 18] DPV has potent activity against wild-type HIV-1 strains and strains harboring different resistance-inducing mutations.[9, 11, 16, 18] The ARV profile of DPV is superior to that of several other NNRTI drugs, including nevirapine (NVP), delavirdine (DLV), and efavirenz (EFV). Like other NNRTIs, *in vitro* tests have also shown that DPV is not active against HIV-2 and has little or no activity against common sexually transmitted infections (STI). Therefore it is not intended for use against HIV-2 or other STIs. DPV does not have any contraceptive properties.[19] Detailed information on DPV is available in the Dapivirine VR Investigator's Brochure (IB).[9]

In vitro metabolism studies showed the main metabolic pathways for DPV to be (slow) oxidation and glucuronidation.[9] Incubations of DPV with human liver microsomes in the presence of diagnostic cytochrome P450 (CYP) inhibitors and with *E. coli* expressing human CYP confirmed CYP3A4 as the main CYP form. Inhibition of CYP3A4 by other drugs had a major effect on the *in vitro* metabolism of DPV, with troleandomycin and ketoconazole inhibiting the overall metabolism by 82% and 66%, respectively, whereas gestodene (23%) was a less potent inhibitor.[9]

The effect of DPV on the expression of CYP enzymes has been determined *in vitro* in human hepatocytes, and it was shown that treatment with DPV at up to 100 ng/mL did not induce CYP1A2 or CYP3A4/5 activity in cultured human hepatocytes.[9] The inhibition of CYP isoenzymes by DPV was investigated *in vitro* in human microsomes using probe substrates selective for human CYP1A2, CYP2A6, CYP2B6, CYP2C9, CYP2C19, CYP2D6, CYP2E1 and CYP3A. CYP1A2 was most potently inhibited by DPV, followed by CYP2C9 and CYP3A. In these *in vitro* studies, systemic exposure to DPV observed in women using the DPV ring (Ring-004) was very low (C_{max} = 392 pg/mL and area under the curve $(AUC)_{0\text{-}24h}$ = 8.4 ng.h/mL) and unlikely to induce the metabolism, or result in significant inhibition of the metabolism, of co-administered drugs. MTN-030/IPM 041 will obtain PK data for both DPV and LNG to inform *in vivo* exposure to this combination of drugs and the potential for other drug-drug interactions to inform appropriate dosing.

IPM has investigated a wide range of dosage formulations for the development of topical microbicide products, including vaginal gels, rings, films, tablets and soft gel capsules. The vaginal gel was the initial dosage form chosen for a DPV-based microbicide because the majority of previous microbicides evaluated in clinical trials were also vaginal gels. Therefore, a wealth of information was available on this dosage form. However, the DPV silicone elastomer VR was prioritized over all other dosage forms for the following reasons:

- Clinical trials have demonstrated sustained delivery of high levels of DPV for up to 1 month;
- Since the VR is able to deliver drug for at least 1 month, the burden of user-dependent adherence is lower than for once daily products;
- Product acceptability studies and the experience gained from marketed VR products have established a high level of acceptance and adherence from women using VR with similar physical characteristics;
- The overall cost for the VR is relatively low;
- Minimal storage space is required for the VR when compared with once daily products.

A summary of the safety of DPV administered orally and vaginally as evaluated in clinical studies by IPM and Janssen Research and Development (formerly Tibotec Pharmaceuticals Ltd.) can be found below.

2.2.2 Mechanism of Action

DPV is an NNRTI; NNRTIs bind to the HIV reverse transcriptase (RT) enzyme thereby preventing viral replication and therefore the production of an infectious virus.

2.2.3 Strength of Study Product

Each of the three study VRs will contain 200 mg of DPV. IPM Ring-104 will contain 200 mg DPV; IPM Ring-101 will contain 200 mg DPV and 32 mg LNG; IPM Ring-102 will contain 200 mg DPV and 320 mg LNG.

2.3 Levonorgestrel

2.3.1 Description

LNG is a second-generation progestin (synthetic progestogen), with chemical name (17a)-(-)-13-ethyl-17-hydroxy-18,19,dinorpregna-4-en-20-yn-3-one.[20] LNG has been used extensively as an active ingredient in hormonal contraceptives including combined oral contraceptive pills, emergency contraceptive pills, intrauterine systems, and contraceptive subdermal implants. LNG has a well-established safety profile with no significant safety findings reported from post-marketing experience, and demonstrated efficacy in many contraceptive formulations. LNG is currently being investigated in other multi-purpose prevention technologies in development.

2.3.2 Mechanism of Action

LNG is a progestin that is thought to work primarily through cervical mucus thickening. LNG also decreases ovulation though does not completely inhibit ovulation in all women in approved effective contraceptives. Other possible mechanisms of action include suppression of midcycle gonadotropin peaks and effects on the endometrium and fallopian tubes.

2.3.3 Strength of Study Product

Each of the three study VRs will contain 200 mg of DPV. IPM Ring-104 will contain 200 mg DPV; IPM Ring-101 will contain 200 mg DPV and 32 mg LNG; IPM Ring-102 will contain 200 mg DPV and 320 mg LNG.

2.4 Nonclinical Studies of Dapivirine

2.4.1 *In vitro* Studies of Dapivirine

<u>Anti-HIV-1 Activity</u>

The antiviral activity of DPV against wild-type (wt) HIV-1, clinical isolates of HIV-1 (including subtype C virus), and a panel of NNRTI-resistant viruses has been

established using *in vitro* models. The 50% effective concentration (EC_{50}) values ranged from 0.3 ng/mL (0.9 nM) against laboratory isolates to <33 ng/mL (<100 nM) against HIV-1 isolates encoding one or more known NNRTI resistance mutations.[16, 19]

The anti-HIV activity of DPV was also confirmed in an ex vivo model of human cervical and colonic explant cultures and a humanized severe combined immunodeficient (hu-SCID) mouse model.[16, 19] *In vitro* testing showed DPV retained activity in the presence of semen; similar EC_{50} of 1.95 nM without and 1.7 nM with semen.[21] Pre-treatment of tissue with DPV for 2 or 24 hours inhibited HIV-1 infection when challenged with virus on Days 0, 2, 4 and 6 post drug removal. DPV was also able to inhibit virus dissemination by migratory cells for up to 6 days post drug removal at concentrations as low as 3.3 µg/mL (10 µM) following treatment for 2 or 24 hours. In addition, DPV 32.9 ng/mL100 µM was able to block transfer of free virus by migratory dendritic cells to indicator T-cells (EC_{50}= 0.03 ng/mL [0.1 nM]). Formulated DPV showed retention of activity by blocking HIV infection of ectocervical tissue at 10 µM and colonic tissue at 1 µM.[21]

Resistance

HIV-1 breakthrough in the presence of DPV was initially evaluated in studies in which cells were infected with wild-type HIV-1 laboratory strains at a high multiplicity of infection and in the presence of increasing concentrations of DPV. At DPV concentration of 40 nM, virus breakthrough occurred between 4 and 7 days; at 200 nM, breakthrough occurred between 7 and 10 days; and at 1 µM, virus breakthrough took up to 30 days to occur. In all cases, mutations were present. Virus that selected for the Y181C mutation was resistant to DPV. Subsequently, cells were infected with wild-type HIV-1 at low multiplicity of infection and were exposed to very low concentrations of DPV to mimic the extremely low systemic concentrations observed in the first clinical trial of one formulation of topical DPV (Gel-001).[9]

Population sequencing performed following prolonged exposure of HIV-1LAI-infected MT4 cells to low concentrations of DPV for a period of approximately 30 days identified several NNRTI resistance-associated mutations, including Y181C, at DPV constant low concentrations (10 nM and 100 nM), but not at constant very low concentrations (1 nM and 0.1 nM). However, both Y181C and V179I were detected when single viral genomes were analyzed by end-point dilution at 1 nM and 0.1 nM concentrations. The frequency of Y181C was 10-12% both at 1 nM and 0.1 nM.[9]

In further experiments using lower DPV concentrations, population sequencing identified the Y181C mutation only at concentrations ≥1 nM, not at lower concentrations. Analysis using more sensitive single genome sequencing indicated the presence of Y181C mutation after exposure to even lower (0.1 nM, and possibly 0.01 nM--approximately 10-fold lower than the EC_{50} for DPV) concentrations of DPV.[9]

Cross-resistance

In comparison with NVP, DLV, EFV and emivirine, DPV showed significantly better *in vitro* activity against laboratory and recombinant HIV strains resistant to one or more drugs of the same class. The EC_{50} was below 32.9 ng/mL (100 nM) for 80% of the strains compared with only 56% of the strains for EFV. When tested against 433 clinical isolates with phenotypic resistance to at least one of the NNRTIs NVP, DLV, EFV or DPV, DPV was able to inhibit 46% (202/433) of the samples including 41% (142/350) of the strains resistant to EFV. In contrast, only 10% (24/231) of the DPV-resistant strains were inhibited by EFV.[9]

Progesterone Receptor Binding

In an *in vitro* assay of progesterone receptor binding, DPV demonstrated the potential to bind the progesterone receptor. However, the relative binding affinity (RBA) compared to progesterone was ~0.1% - 0.2% and 3230 fold lower than that of LNG (RBA = 323%).[20] Therefore, DPV is unlikely to impede the progestogenic activity of LNG.

2.4.2 Condom Compatibility Studies of Dapivirine

Chemical compatibility studies with different DPV-containing gel formulations have been conducted on the following types of condoms:[9]

- Non-lubricated latex condoms (male condom);
- Silicone lubricated latex condoms (male and female condoms);
- Aqueous lubricated latex condoms (male condom);
- Polyurethane condoms with silicone lubricant (male and female condoms); and
- Nitrile condoms with silicone lubricant (female condom).

The results of condom compatibility testing indicate that DPV-containing vaginal gel formulations (0.05%) have no deleterious effects on the integrity of male or female condoms, as indicated by tensile condom properties tested pre- and post-treatment. Two clinical condom functionality studies (one with male condoms [IPM 029] and one with female condoms [IPM 033]) were conducted with a placebo VR (silicone elastomer ring containing no active ingredient). Results from both studies showed that the difference between the total clinical failure rate between condom use while using a VR and condom use while not using a VR was less than the pre-defined non-inferiority margins in both studies (3% for the male condom study and 8% for the female condom study). Condom use was safe and well tolerated during VR use.

2.5 Clinical Studies of Dapivirine

2.5.1 Clinical Studies of Dapivirine Vaginal Rings

To date, 27 Phase I and Phase I/II clinical trials of DPV with various formulations have been completed. These include eight trials of DPV vaginal rings in 469 participants (298

using DPV rings and 183 using placebo rings); eight trials of DPV vaginal gel in 774 participants (491 using DPV gel and 283 using placebo gel); and 11 trials of oral DPV including a total of 211 participants.

Additionally, two pivotal Phase III trials (IPM 027 [The Ring Study] and MTN-020 [ASPIRE]) evaluating long-term safety and efficacy of the 25 mg DPV vaginal Ring-004 (IND 108,743), in which the vaginal ring was replaced with a new vaginal ring after approximately 28 days of use, were recently completed, having enrolled a total of 4588 participants.

In March 2012, IPM 027, also known as The Ring Study, was initiated. IPM 027 was a randomized, double-blind, placebo-controlled efficacy and long-term safety study that enrolled 1959 healthy, HIV-uninfected women, ages 18-45. The study was conducted in South Africa and Uganda. Study participants used either the DPV VR or the placebo VR every four weeks over approximately two years. The main goals of The Ring Study were to evaluate the long-term safety and efficacy of the DPV VR for the prevention of HIV-1 as compared to a placebo VR, when used by healthy, HIV-negative women over a two-year period. Additional goals included measuring the incidence of curable STIs, HIV-2 and pregnancy; monitoring ring acceptability (how well women like using the ring) and adherence (if women use the ring as intended) as reported by the study participants; and tracking the development of any HIV-1 drug resistance in participants who became HIV positive during the study. A total of 1959 women (1762 in South Africa and 197 in Uganda) were randomized in a 2:1 ratio to receive either a DPV VR or a placebo VR. The median age at enrollment was 25 years; 91% were unmarried. At the data cut-off point, the total number of person years of follow-up was 2805, and 761 women had completed the two year follow-up period. Results were presented at CROI 2016.

MTN-020, A Study to Prevent Infection with a Ring for Extended Use (ASPIRE), was a Phase 3 clinical trial designed to assess the efficacy and safety of a ring containing 25 mg of DPV for the prevention of HIV-1 acquisition in women. The double-blind, randomized controlled trial was conducted in HIV-uninfected women between the ages of 18 and 45. A total of 2629 women from Malawi, South Africa, Uganda, and Zimbabwe enrolled in the trial. Participants replaced the ring monthly for a minimum of one year. MTN-020 aimed to determine the safety and efficacy of the DPV VR in preventing HIV-1 infection among healthy, sexually active, HIV-uninfected women when inserted vaginally once every 4 weeks. Additional goals of MTN-020 included the assessment of participant acceptability and adherence to the investigational product, HIV-1 drug resistance mutations among participants who acquired HIV-1 infection, and establishing steady state drug concentrations in the study population. Results were published in the New England Journal of Medicine and presented at CROI 2016.

Clinical Pharmacokinetics of Dapivirine

The highest daily dose of DPV delivered from a vaginal gel to date (Gel 4750, Gel 4789 and Gel 4759, approximately 1250 µg/day) is 280 times lower than the maximum

tolerated single dose for oral DPV (350 mg) and 480 times lower than the maximum tolerated multiple dose for oral DPV (300 mg twice daily for 14 days).

Following single and multiple oral doses of DPV, maximum plasma concentrations were generally reached 1 to 3 hours after dose intake. Participants were exposed to oral doses of DPV ranging from 50 mg to 1000 mg daily. At the maximum tolerated multiple dose of 300 mg b.i.d. for 14 days, C_{max} plasma concentration was 2286 ng/mL and $AUC_{0\text{-}12h}$ was 18247 ng.h/mL. These values are more than 3000 times and 1200 times higher, respectively, than the mean C_{max} (705 pg/mL to 716 pg/mL) and $AUC_{0\text{-}24h}$ (approximately 15 ng.h/mL) values following once daily application of Gel 4789 and Gel 4750 (both 0.05% DPV, 2.5 g) for 10 days, and more than 5800 times and 2100 times higher, respectively, than the maximum mean C_{max} (392 pg/mL) and $AUC_{0\text{-}24h}$ (8.4 ng.h/mL) values observed in women using DPV Ring-004 for 28 days. These data suggest a wide safety margin when comparing the highest report systemic exposures of orally administered and vaginally administered DPV.

Across all completed trials with various DPV gel formulations plasma concentrations of DPV were very low (≤ 2.33 ng/mL), and were therefore well below plasma concentrations observed at the maximum tolerated dose (MTD) following multiple oral doses (300 mg b.i.d. for 14 days; plasma C_{max} of 2286 ng/mL). In trials evaluating multiple concentrations of the same formulation, systemic exposure generally increased approximately proportionally with increasing dose.

The pharmacokinetic profile of DPV Ring-004 showed a rapid increase in plasma and vaginal fluid concentrations of DPV after ring insertion, resulting in maximum concentrations in plasma by Day 7 and in vaginal fluids between Day 1 and Day 14, after which concentrations decreased steadily over the remainder of the 28-day ring use period. When extending the vaginal ring use period to 56 days (8 weeks) and 84 days (12 weeks) (IPM 034), both plasma and vaginal fluids showed a linear decline in DPV concentrations with duration of ring use. Plasma DPV concentrations did not exceed 1 ng/mL, and were therefore well below concentrations at the MTD for multiple oral doses (300 mg b.i.d. for 14 days; plasma C_{max} of 2286 ng/mL).

Furthermore, data from post-use analysis of residual levels of DPV in Ring-004 (IPM 015, IPM 034) indicate that, on average, 4-5 mg of DPV were released over approximately one month of ring use and that mean residual ring levels of DPV after 84 days of continual use was 15 mg of DPV, suggesting a mean release of 10 mg over the 84 day period.

Vaginal fluid concentrations of DPV (IPM 020, Gel 4759 and 4789, 0.05%, 2.5 g) were markedly higher than plasma concentrations. Whereas the individual plasma concentrations did not exceed 2.33ng/mL, the highest individual vaginal fluid concentration was 9860 ng/mL after 2 weeks of daily use. In IPM 012 (Gel 4750 and 4789, 0.05%, 2.5 g) the highest concentration of DPV in vaginal fluids was observed in the area where the ring was placed, followed by the cervix, with the lowest concentrations near the introitus. When comparing vaginal fluid levels between vaginal

rings and vaginal gels C_{max} (79.9 µg/mL) and $AUC_{0\text{-}24h}$ (1035 µg.h/g) values in vaginal fluids after use of a single or up to three vaginal rings were approximately 3 fold lower than the corresponding values after single and multiple doses of Gel 4750 and Gel 4789 (C_{max} measured at the cervix was approximately 222 µg/mL on day 1 and $AUC_{0\text{-}24h}$ was 3250 µg.h/g).

Extending the period that a single DPV Ring-004 was used (IPM 034) to 56 days (8 weeks) and 84 days (12 weeks), resulted in vaginal fluid concentrations (collected at the cervix) declining gradually with the period of ring use: the mean concentration prior to ring removal was similar for rings inserted for 1 or 2 weeks (39.5 and 44.6 µg/g), and then declined to 20.1 µg/g at Week 4, 17.2 µg/g at Week 8, and 13.3 µg/g at Week 12. The lowest individual DPV vaginal fluid concentration observed after 84 days (12 weeks) prior to ring removal was 1138 ng/g, which was still 345 times above the level at which greater than 99% inhibition of integrated provirus was observed (IC_{99}; 3.3 ng/mL).

IPM 001 was a Phase I, crossover, open-label trial of Ring-001 (200 mg DPV) in 12 healthy, sexually abstinent, HIV-negative women, 18 to 50 years of age, conducted at a single site in Belgium. Each woman used a placebo silicone elastomer VR for 7 days, followed by 7 days of use of a VR containing 200 mg DPV. Samples of vaginal fluids from the introitus, cervix, and ring area were collected from all participants at 4 hours, 24 hours, and 7 days after insertion of the VR. At 4 hours and 24 hours post-insertion, mean DPV concentrations in vaginal fluids were higher in the ring area (4.7; 6.1 µg/mL) than at the introitus (0.7; 1.3 µg/mL) or cervix (2.3; 2.0 µg/mL). At 7 days post-insertion, mean concentrations at the introitus, cervix, and ring area were 2.9 µg/mL, 1.9 µg/mL, and 1.8 µg/mL, respectively. Tissue biopsies of the vagina (introitus and ring area) and cervix were collected immediately following removal of the VR, 7 days after insertion. Mean DPV concentrations were higher in the ring area (0.7 µg/g) than in the introitus or cervix (0.3 µg/g each). The lowest observed concentration of DPV in any tissue sample (28.0 ng/g, at the introitus) was over 30-fold greater than the reported EC_{90} for DPV *in vitro*. Plasma concentrations were measured from 4 hours through 7 days post-insertion. DPV was detectable, but concentrations were below the limit of quantification of the assay (< 50 pg/mL) for all samples. For Ring-001, only limited PK data are available, but these showed that plasma levels of DPV were consistently below the limit of quantification (LLOQ = 0.05 ng/mL), and levels in fluid and tissue were much lower than for Ring-004 (DPV levels in vaginal fluids were between 10 and 28 times higher, and in cervical tissue about five times higher, for Ring-004 than Ring-001).

In MTN-020, in the DPV group, DPV was detected in 82% of plasma samples at levels of greater than 95 pg/mL. Detection increased over the first year of use and remained relatively stable thereafter. In the subgroup of visits in which returned VRs were available, 84% contained less than 23.5 mg DPV, and DPV levels in plasma and in returned VRs were correlated. In general, for visits at which plasma DPV levels were less than 95 pg/mL, residual DPV levels in used VRs were similar to levels in unused rings, whereas residual DPV levels in used rings were lower for visits at which plasma DPV levels exceeded 95 pg/mL. However, a range of residual DPV levels was

observed, with low levels observed for some visits with low plasma DPV levels and high levels observed for some visits with plasma DPV levels of more than 95 pg/mL.[14]

Safety of Dapivirine

In a series of 11 oral DPV clinical trials, 211 participants were exposed to oral doses of DPV ranging from 50 mg to 1000 mg daily. The MTD established was 350 mg for a single dose and, for multiple doses 300 mg twice daily. No serious adverse drug reactions were reported and no deaths occurred during these trials. TEAEs reported in more than 2% of participants included headache, dizziness, nausea, diarrhea, fatigue, tremor, somnolence, flatulence and vomiting. Elevations in alanine aminotransferase (ALT) and aspartate aminotransferase (AST) (maximum severity grade 3) were observed and these increases were transient and did not result in permanent liver impairment. One HIV-infected participant had to be withdrawn from the trial because of increases in AST and ALT, which were assessed to be related to an acute concomitant hepatitis C infection. This event was the only serious adverse event (AE) that led to withdrawal from the trial across all trials with oral DPV. In IPM 027, product-related AEs included metrorrhagia, menometrorrhagia, pelvic discomfort/pain, suprapubic pain and application site pain. The rate of AEs, including product-related AEs, urogenital AEs, serious AEs and deaths, was similar between treatment arms. In MTN-020, the rate of adverse medical events was similar between study arms. There were no statistically significant differences in the frequency of the primary safety endpoints between the study arms or in other AEs commonly detected in the study population. Incident sexually transmitted infections occurred at a similar rate in the two study arms.

Across all completed clinical trials with vaginally administered DPV formulations in healthy participants, including the two recently-completed Phase III trials described above (MTN-020 and IPM 027), DPV was safe and well tolerated. No serious adverse drug reactions have been reported and none of the serious adverse events (SAEs) reported with a fatal outcome were attributed to investigational product (IP) use. No trials have been stopped or paused for safety reasons by an Independent Data Safety Monitoring Board.

Efficacy of Dapivirine

In IPM 027, a total of 133 post-randomization HIV-1 infections occurred: 77 among women assigned to the DPV VR (incidence 4.08 per 100 person-years) and 56 among women assigned to placebo VR (incidence 6.10 per 100 person-years). The DPV VR reduced the risk of HIV-1 infection by 30.7% (95% CI: 0.90-51.5%; p=0.0401) relative to placebo VR. A 37.5% (95% CI: 3.5-59.5%) reduction in HIV-1 infection was observed in a subgroup analysis of women older than 21 years.

In MTN-020, a total of 168 HIV-1 infections occurred: 71 among those assigned the DPV VR and 97 among those assigned the placebo VR (incidence 3.3 and 4.5 per 100 person-years, respectively). The DPV VR resulted in a 27% (95% CI: 1-46%, p=0.05) relative reduction in HIV-1 incidence overall, a 37% (95% CI: 12-56%, p=0.007)

reduction in an analysis defined early in the study, excluding data from two study sites with lower retention and adherence, and a 56% (95% CI: 31-71%, p<0.001) reduction in a post-hoc analysis among women older than 21 years of age. HIV-1 protection was not observed for women aged 18-21, and objective markers of adherence were lower in this subgroup compared to women older than 21. Finally, among those acquiring HIV-1, the detection of NNRTI mutations did not differ by study arm (8/68 assigned dapivirine and 10/96 assigned placebo, p=0.80). The frequency of ARV resistance was also similar between study arms.

Treatment Discontinuations with Vaginally Administered Dapivirine

Given the lower systemic exposure following vaginal administration of DPV and the lower potential for systemic toxicities it is not surprising that very few participants have required permanent discontinuation of the investigational product (IP). All TEAEs leading to permanent IP discontinuations have been due to non-serious AEs.

Only one participant using the DPV Ring-004 discontinued the trial due to a TEAE of generalized pruritus. In the IPM 001 trial (DPV Ring-001), no participants discontinued the trial due to TEAEs. In clinical trials of DPV vaginal gels four participants discontinued DPV gel due to non-serious AEs. These events included a grade 1 (mild) hypersensitivity reaction (reported by the investigator as an allergic response; symptoms and signs included vaginal burning, itching and erythema) and an event of worsening of a cervico-vaginal human papilloma virus infection in the DPV Gel 4759 group. Other events reported included grade 2 (moderate) vaginal itching, grade 2 vulvar irritation along with vaginal pruritus (in the same participant) and grade 1 (mild) inter-menstrual bleeding, both occurring in users of Gel 4789 (0.05%, 2.5 g).

Lack of Significant Local Toxicity with Vaginally Administered Dapivirine

Completed trials of DPV vaginal gels and DPV Ring-004 have generally indicated the absence of significant local toxicity. In all completed trials including vaginal Gel 4759 (0.05%, 2.5 g) the cumulative incidence of TEAEs was generally similar across DPV gel and placebo treatment arms apart from vulvovaginal pruritus that occurred at a frequency of 7.6% across DPV vaginal gel arms and 4.4% across placebo arms. Table 1 presents the cumulative incidence of AEs across placebo and Gel 4579 arms. All AE terms with a reported incidence of at least 5% in each contributing trial were included. AE terms describing genitourinary events of interest are highlighted in bolded italics.

Table 1:Treatment-Emergent Adverse Events (≥ 5% for Either Treatment Group) Across Completed Dapivirine Vaginal Gel 4759 Trials

MedDRA Preferred Term	IPM 014A		IPM 020*		Total	
	Dapivirine Gel 4759 (N=141)	Placebo (N=139)	Dapivirine Gel 4759 (N=43)	Placebo (N=42)	Dapivirine Gel 4759 (N=184)	Placebo (N=181)
	n (%)	n (%)	n (%)	n (%)	n (%)	n (%)
Participants with at least one TEAE	103 (73.0)	104 (74.8)	30 (69.8)	35 (83.3)	133 (72.3)	139 (76.8)
Intermenstrual bleeding	***39 (27.7)***	***31 (22.3)***	***1 (2.3)***	***4 (9.5)***	***40 (21.7)***	***35 (19.3)***
Vulvovaginal pruritus	***14 (9.9)***	***6 (4.3)***	***0 (0.0)***	***2 (4.8)***	***14 (7.6)***	***8 (4.4)***
Upper respiratory tract infection	12 (8.5)	9 (6.5)	1 (2.3)	3 (7.1)	13 (7.1)	12 (6.6)
Vaginitis, Bacterial	***6 (4.3)***	***8 (5.8)***	***7 (16.3)***	***5 (11.9)***	***13 (7.1)***	***13 (7.2)***
Headache	11 (7.8)	9 (6.5)	2 (4.7)	3 (7.1)	13 (7.1)	12 (6.6)
Vaginal candidiasis	***5 (3.5)***	***9 (6.5)***	***4 (9.3)***	***0 (0.0)***	***9 (4.9)***	***9 (5.0)***
Gonorrhea	***7 (5.0)***	***5 (3.6)***	***0 (0.0)***	***1 (2.4)***	***7 (3.8)***	***6 (3.3)***
Gynecological chlamydia infection	***7 (5.0)***	***7 (5.0)***	***0 (0.0)***	***0 (0.0)***	***7 (3.8)***	***7 (3.9)***
Urinary tract infection	4 (2.8)	4 (2.9)	2 (4.7)	2 (4.8)	6 (3.3)	6 (3.3)
Abdominal pain, lower	3 (2.1)	4 (2.9)	2 (4.7)	0 (0.0)	5 (2.7)	4 (2.2)
Oligomenorrhoea	***5 (3.5)***	***10 (7.2)***	***0 (0.0)***	***0 (0.0)***	***5 (2.7)***	***10 (5.5)***
Abdominal pain	2 (1.4)	0 (0.0)	1 (2.3)	2 (4.8)	3 (1.6)	2 (1.1)
Gastroenteritis	3 (2.1)	7 (5.0)	0 (0.0)	0 (0.0)	3 (1.6)	7 (3.9)
Nasopharyngitis	1 (0.7)	1 (0.7)	2 (4.7)	2 (4.8)	3 (1.6)	3 (1.7)
Vaginal discharge	***3 (2.1)***	***4 (2.9)***	***0 (0.0)***	***3 (7.1)***	***3 (1.6)***	***7 (3.9)***
Cough	1 (0.7)	0 (0.0)	2 (4.7)	0 (0.0)	3 (1.6)	0 (0.0)
Hypersensitivity	0 (0.0)	0 (0.0)	2 (4.7)	0 (0.0)	2 (1.1)	0 (0.0)
Muscle spasms	1 (0.7)	0 (0.0)	1 (2.3)	2 (4.8)	2 (1.1)	2 (1.1)
Dysmenorrhoea	***0 (0.0)***	***2 (1.4)***	***2 (4.7)***	***0 (0.0)***	***2 (1.1)***	***2 (1.1)***
Pharyngolaryngeal pain	0 (0.0)	0 (0.0)	2 (4.7)	0 (0.0)	2 (1.1)	0 (0.0)
Diarrhea	1 (0.7)	1 (0.7)	0 (0.0)	4 (9.5)	1 (0.5)	5 (2.8)
Adnexa uteri pain	***0 (0.0)***	***0 (0.0)***	***1 (2.3)***	***2 (4.8)***	***1 (0.5)***	***2 (1.1)***
Cervix erythema	***0 (0.0)***	***0 (0.0)***	***1 (2.3)***	***2 (4.8)***	***1 (0.5)***	***2 (1.1)***
Erythema	0 (0.0)	1 (0.7)	1 (2.3)	2 (4.8)	1 (0.5)	3 (1.7)
Vomiting	0 (0.0)	1 (0.7)	0 (0.0)	3 (7.1)	0 (0.0)	4 (2.2)
Hemoglobin, decreased	0 (0.0)	0 (0.0)	0 (0.0)	2 (4.8)	0 (0.0)	2 (1.1)
Dysuria	0 (0.0)	1 (0.7)	0 (0.0)	3 (7.1)	0 (0.0)	4 (2.2)
Dyspareunia	***0 (0.0)***	***0 (0.0)***	***0 (0.0)***	***2 (4.8)***	***0 (0.0)***	***2 (1.1)***
Vulvovaginal discomfort	***0 (0.0)***	***2 (1.4)***	***0 (0.0)***	***2 (4.8)***	***0 (0.0)***	***4 (2.2)***

* In order to compare results from IPM 020 with those obtained for IPM 014A, the Gel 4789 arm from IPM 020 is not included in this table.

In all completed trials with the DPV Ring-004 the cumulative incidence of TEAEs were generally similar (or lower in some cases) in the DPV ring compared to the placebo ring arms. Metrorrhagia (29.7% vs 24.4%), headache (15.1% vs 11.9%), nausea (5.0% vs 1.0%) and vulvovaginal discomfort (2.7 vs 1.3%) were reported more frequently in users of the DPV vaginal ring than the placebo ring respectively. Table 2 presents the cumulative incidence of AEs across placebo and Ring-004 arms. All AE terms with a reported incidence of at least 5% in each contributing trial were included. AE terms describing genitourinary events of interest are highlighted in bolded italics.

There were no SAEs in the IPM 001 trial (DPV Ring-001). TEAE profiles were similar during the placebo ring and DPV VR phases of the trial; 75% (9/12) of women experienced TEAEs during each phase. All TEAEs were classified as Grade 1 (mild) or Grade 2 (moderate) in severity per DAIDS grading. Vaginal hemorrhage occurred in 6 (50%) participants, 2 (17%) during the placebo phase and 5 (42%) during the active phase. The median duration of bleeding was 7 days. All cases of vaginal hemorrhage were assessed by the investigator as Grade 1 and doubtfully related to the VR. No trend was observed between the occurrence of vaginal hemorrhage and VR insertion/removal or tissue biopsy, and could have been caused by breakthrough bleeding associated with oral contraceptive use. Other events that occurred in a greater percentage of participants using DPV Ring-001 were fatigue (3/12 [25%] versus 1/12 [8%]), abdominal discomfort (2/12 [17%] versus 1/12 [8%]), and genital pruritus (2/12 [17%] versus 0/12 [0%]). All cases of abdominal discomfort, fatigue, genital discharge, genital pruritus, and vaginal discharge, and 2 of 3 (67%) cases of headache, were assessed as possibly related to the VR.

Table 2: Treatment-Emergent Adverse Events (≥ 5% for either Treatment Group) Across Completed Dapivirine Vaginal Ring-004 Trials

MedDRA Preferred Term*	IPM 013		IPM 015		IPM 024		IPM 028**	IPM 034**	Total	
	Dapivirine (N=36)	Placebo (N=12)	Dapivirine (N=140)	Placebo (N=140)	Dapivirine (N=8)	Placebo (N=8)	Dapivirine (N=35)	Dapivirine (N=40)	Dapivirine (N=259)	Placebo (N=160)
	n (%)	n (%)	n (%)	n (%)	n (%)	n (%)	n (%)	n (%)	n (%)	n (%)
Participants with report of any treatment emergent adverse event	32 (88.9)	11 (91.7)	114 (81.4)	121 (86.4)	7 (87.5)	8 (100.0)	31 (88.6)	25 (62.5)	209 (80.7)	140 (87.5)
Metrorrhagia	***21 (58.3)***	***9 (75.0)***	***26 (18.6)***	***27 (19.3)***	***4 (50.0)***	***3 (37.5)***	***13 (37.1)***	***13 (32.5)***	***77 (29.7)***	***39 (24.4)***
Headache	14 (38.9)	6 (50.0)	7 (5.0)	10 (7.1)	4 (50.0)	3 (37.5)	11 (31.4)	3 (7.5)	39 (15.1)	19 (11.9)
Gynecological chlamydia infection	***0 (0.0)***	***0 (0.0)***	***22 (15.7)***	***22 (15.7)***	***0 (0.0)***	***0 (0.0)***	***0 (0.0)***	***0 (0.0)***	***22 (8.5)***	***22 (13.4)***
Vaginal candidiasis	***1 (2.8)***	***0 (0.0)***	***20 (14.3)***	***12 (8.6)***	***0 (0.0)***	***0 (0.0)***	***0 (0.0)***	***0 (0.0)***	***21 (8.1)***	***12 (7.5)***
Urinary tract infection	***0 (0.0)***	***0 (0.0)***	***18 (12.9)***	***14 (10.0)***	***0 (0.0)***	***0 (0.0)***	***0 (0.0)***	***0 (0.0)***	***18 (6.9)***	***14 (8.8)***
Vaginal discharge	***3 (8.3)***	***2 (16.7)***	***10 (7.1)***	***7 (5.0)***	***0 (0.0)***	***1 (12.5)***	***1 (2.9)***	***3 (7.5)***	***17 (6.6)***	***10 (6.3)***
Upper respiratory tract infection	0 (0.0)	0 (0.0)	15 (10.7)	16 (11.4)	0 (0.0)	0 (0.0)	0 (0.0)	0 (0.0)	15 (5.8)	16 (10.0)
Abdominal pain, lower	4 (11.1)	4 (33.3)	2 (1.4)	3 (2.1)	0 (0.0)	1 (12.5)	4 (11.4)	4 (10.0)	14 (5.4)	8 (5.0)
Nasopharyngitis	1 (2.8)	1 (8.3)	1 (0.7)	1 (0.7)	2 (25.0)	1 (12.5)	5 (14.3)	5 (12.5)	14 (5.4)	3 (1.9)
Nausea	5 (13.9)	1 (8.3)	0 (0.0)	1 (0.7)	0 (0.0)	0 (0.0)	6 (17.1)	2 (5.0)	13 (5.0)	2 (1.3)
Vulvovaginal pruritus	***2 (5.6)***	***0 (0.0)***	***7 (5.0)***	***6 (4.3)***	***1 (12.5)***	***1 (12.5)***	***1 (2.9)***	***0 (0.0)***	***11 (4.2)***	***7 (4.4)***
Asymptomatic bacteriuria	0 (0.0)	0 (0.0)	11 (7.9)	7 (5.0)	0 (0.0)	0 (0.0)	0 (0.0)	0 (0.0)	11 (4.2)	7 (4.4)
Vaginitis bacterial	***0 (0.0)***	***0 (0.0)***	***10 (7.1)***	***13 (9.3)***	***0 (0.0)***	***1 (12.5)***	***0 (0.0)***	***1 (2.5)***	***11 (4.2)***	***14 (8.8)***
Abdominal pain	4 (11.1)	5 (41.7)	1 (0.7)	3 (2.1)	1 (12.5)	1 (12.5)	3 (8.6)	0 (0.0)	9 (3.5)	9 (5.6)
Back pain	2 (5.6)	0 (0.0)	4 (2.9)	5 (3.6)	0 (0.0)	2 (25.0)	1 (2.9)	1 (2.5)	8 (3.1)	7 (4.4)
Oligomenorrhoea	***0 (0.0)***	***0 (0.0)***	***8 (5.7)***	***2 (1.4)***	***0 (0.0)***	***0 (0.0)***	***0 (0.0)***	***0 (0.0)***	***8 (3.1)***	***2 (1.3)***

* MedDRA Version 10.0 was used for IPM 013, IPM 015 and IPM 024. MedDRA Version 15.0 was used for IPM 028 and IPM 034.

** There were no placebo arms in the IPM 028 and 034 trials. For the IPM 028 trial, TEAEs from the DPV ring only arm (Treatment A) are presented. 36 women were enrolled in IPM 028 of which 35 received the DPV vaginal ring in Treatment A.

Table 2: **Treatment-Emergent Adverse Events (≥ 5% for either Treatment Group) across Completed Dapivirine Vaginal Ring-004 Trials (continued)**

MedDRA Preferred Term*	IPM 013		IPM 015		IPM 024		IPM 028**	IPM 034**	Total	
	Dapivirine (N=36)	Placebo (N=12)	Dapivirine (N=140)	Placebo (N=140)	Dapivirine (N=8)	Placebo (N=8)	Dapivirine (N=35)	Dapivirine (N=40)	Dapivirine (N=259)	Placebo (N=160)
	n (%)	n (%)	n (%)	n (%)	n (%)	n (%)	n (%)	n (%)	n (%)	n (%)
Gonorrhea	***0 (0.0)***	***0 (0.0)***	***7 (5.0)***	***10 (7.1)***	***0 (0.0)***	***0 (0.0)***	***0 (0.0)***	***0 (0.0)***	***7 (2.7)***	***10 (6.3)***
Vulvovaginal discomfort	***4 (11.1)***	***1 (8.3)***	***0 (0.0)***	***1 (0.7)***	***3 (37.5)***	***0 (0.0)***	***0 (0.0)***	***0 (0.0)***	***7 (2.7)***	***2 (1.3)***
Diarrhea	0 (0.0)	2 (16.7)	2 (1.4)	0 (0.0)	0 (0.0)	2 (25.0)	4 (11.4)	0 (0.0)	6 (2.3)	4 (2.5)
Oropharyngeal pain	0 (0.0)	0 (0.0)	0 (0.0)	0 (0.0)	0 (0.0)	0 (0.0)	3 (8.6)	3 (7.5)	6 (2.3)	0 (0.0)
Neck pain	3 (8.3)	0 (0.0)	1 (0.7)	0 (0.0)	0 (0.0)	1 (12.5)	2 (5.7)	0 (0.0)	6 (2.3)	1 (0.6)
Influenza-like illness	3 (8.3)	0 (0.0)	0 (0.0)	0 (0.0)	0 (0.0)	2 (25.0)	1 (2.9)	1 (2.5)	5 (1.9)	2 (1.3)
Dyspepsia	4 (11.1)	1 (8.3)	1 (0.7)	4 (2.9)	0 (0.0)	1 (12.5)	0 (0.0)	0 (0.0)	5 (1.9)	6 (3.8)
Urogenital trichomoniasis	***0 (0.0)***	***0 (0.0)***	***5 (3.6)***	***8 (5.7)***	***0 (0.0)***	***0 (0.0)***	***0 (0.0)***	***0 (0.0)***	***5 (1.9)***	***8 (5.0)***
Dysuria	2 (5.6)	1 (8.3)	1 (0.7)	0 (0.0)	1 (12.5)	0 (0.0)	0 (0.0)	0 (0.0)	4 (1.5)	1 (0.6)
Migraine	1 (2.8)	0 (0.0)	0 (0.0)	0 (0.0)	0 (0.0)	1 (12.5)	2 (5.7)	1 (2.5)	4 (1.5)	1 (0.6)
Abdominal distension	2 (5.6)	2 (16.7)	0 (0.0)	0 (0.0)	0 (0.0)	0 (0.0)	2 (5.7)	0 (0.0)	4 (1.5)	2 (1.3)
Arthralgia	0 (0.0)	1 (8.3)	2 (1.4)	0 (0.0)	0 (0.0)	0 (0.0)	1 (2.9)	1 (2.5)	4 (1.5)	1 (0.6)
Dysmenorrhoea	***0 (0.0)***	***1 (8.3)***	***4 (2.9)***	***3 (2.1)***	***0 (0.0)***	***0 (0.0)***	***0 (0.0)***	***0 (0.0)***	***4 (1.5)***	***4 (2.5)***
Pharyngolaryngeal pain	3 (8.3)	3 (25.0)	0 (0.0)	1 (0.7)	1 (12.5)	0 (0.0)	0 (0.0)	0 (0.0)	4 (1.5)	4 (2.5)
Dermatitis contact	1 (2.8)	0 (0.0)	0 (0.0)	0 (0.0)	0 (0.0)	0 (0.0)	2 (5.7)	0 (0.0)	3 (1.2)	0 (0.0)
Gastroenteritis	1 (2.8)	1 (8.3)	1 (0.7)	8 (5.7)	0 (0.0)	0 (0.0)	1 (2.9)	0 (0.0)	3 (1.2)	9 (5.6)
Dry skin	0 (0.0)	0 (0.0)	0 (0.0)	0 (0.0)	0 (0.0)	0 (0.0)	2 (5.7)	1 (2.5)	3 (1.2)	0 (0.0)
Rash	2 (5.6)	0 (0.0)	0 (0.0)	0 (0.0)	0 (0.0)	1 (12.5)	0 (0.0)	1 (2.5)	3 (1.2)	1 (0.6)
Vomiting	1 (2.8)	0 (0.0)	0 (0.0)	0 (0.0)	0 (0.0)	0 (0.0)	2 (5.7)	0 (0.0)	3 (1.2)	0 (0.0)

* MedDRA Version 10.0 was used for IPM 013, IPM 015 and IPM 024. MedDRA Version 15.0 was used for IPM 028 and IPM 034.

** There were no placebo arms in the IPM 028 and 034 trials. For the IPM 028 trial, TEAEs from the DPV ring only arm (Treatment A) are presented. 36 women were enrolled in IPM 028 of which 35 received the DPV vaginal ring in Treatment A.

Table 2: Treatment-Emergent Adverse Events (≥ 5% for either Treatment Group) across Completed Dapivirine Vaginal Ring-004 Trials (continued)

MedDRA Preferred Term*	IPM 013 Dapivirine (N=36)	IPM 013 Placebo (N=12)	IPM 015 Dapivirine (N=140)	IPM 015 Placebo (N=140)	IPM 024 Dapivirine (N=8)	IPM 024 Placebo (N=8)	IPM 028** Dapivirine (N=35)	IPM 034** Dapivirine (N=40)	Total Dapivirine (N=259)	Total Placebo (N=160)
	n (%)	n (%)	n (%)	n (%)	n (%)	n (%)	n (%)	n (%)	n (%)	n (%)
Catheter site pain	1 (2.8)	0 (0.0)	0 (0.0)	0 (0.0)	0 (0.0)	0 (0.0)	2 (5.7)	0 (0.0)	3 (1.2)	0 (0.0)
Fatigue	1 (2.8)	2 (16.7)	0 (0.0)	1 (0.7)	0 (0.0)	0 (0.0)	2 (5.7)	0 (0.0)	3 (1.2)	3 (1.9)
Pyrexia	0 (0.0)	0 (0.0)	1 (0.7)	0 (0.0%)	1 (12.5)	1 (12.5)	1 (2.9)	0 (0.0)	3 (1.2)	1 (0.6)
Sinusitis	0 (0.0)	0 (0.0)	1 (0.7)	0 (0.0%)	0 (0.0)	0 (0.0)	2 (5.7)	0 (0.0)	3 (1.2)	0 (0.0)
Vessel puncture site hematoma	0 (0.0)	0 (0.0)	0 (0.0)	0 (0.0)	0 (0.0)	0 (0.0)	2 (5.7)	0 (0.0)	2 (0.8)	0 (0.0)
Asthenia	1 (2.8)	0 (0.0)	0 (0.0)	0 (0.0)	0 (0.0)	0 (0.0)	1 (2.9)	0 (0.0)	2 (0.8)	0 (0.0)
Acne	1 (2.8)	1 (8.3)	0 (0.0)	0 (0.0)	1 (12.5)	0 (0.0)	0 (0.0)	0 (0.0)	2 (0.8)	1 (0.6)
Pre-syncope	2 (5.6)	0 (0.0)	0 (0.0)	0 (0.0)	0 (0.0)	0 (0.0)	0 (0.0)	0 (0.0)	2 (0.8)	0 (0.0)
Procedural pain	0 (0.0)	0 (0.0)	0 (0.0)	0 (0.0)	0 (0.0)	0 (0.0)	0 (0.0)	2 (5.0)	2 (0.8)	0 (0.0)
Somnolence	2 (5.6)	0 (0.0)	0 (0.0)	0 (0.0)	0 (0.0)	0 (0.0)	0 (0.0)	0 (0.0)	2 (0.8)	0 (0.0)
Vaginal lesion	***0 (0.0)***	***0 (0.0)***	***1 (0.7)***	***0 (0.0)***	***1 (12.5)***	***0 (0.0)***	***0 (0.0)***	***0 (0.0)***	***2 (0.8)***	***0 (0.0)***
Vulvovaginal candidiasis	***0 (0.0)***	***0 (0.0)***	***0 (0.0)***	***0 (0.0)***	***0 (0.0)***	***0 (0.0)***	***2 (5.7)***	***0 (0.0)***	***2 (0.8)***	***0 (0.0)***
Breast pain	0 (0.0)	0 (0.0)	0 (0.0)	0 (0.0)	1 (12.5)	0 (0.0)	0 (0.0)	0 (0.0)	1 (0.4)	0 (0.0)
Nasal congestion	0 (0.0)	0 (0.0)	0 (0.0)	0 (0.0)	1 (12.5)	0 (0.0)	0 (0.0)	0 (0.0)	1 (0.4)	0 (0.0)
Seborrhoeic dermatitis	0 (0.0)	0 (0.0)	0 (0.0)	0 (0.0)	0 (0.0)	0 (0.0)	1 (2.9)	0 (0.0)	1 (0.4)	0 (0.0)
Vaginal hemorrhage	***0 (0.0)***	***0 (0.0)***	***0 (0.0)***	***2 (1.4)***	***1 (12.5)***	***0 (0.0)***	***0 (0.0)***	***0 (0.0)***	***1 (0.4)***	***2 (1.3)***
Abdominal pain, upper	0 (0.0)	1 (8.3)	0 (0.0)	0 (0.0)	0 (0.0)	0 (0.0)	0 (0.0)	1 (2.5)	1 (0.4)	1 (0.6)
Ecchymosis	0 (0.0)	0 (0.0)	0 (0.0)	0 (0.0)	1 (12.5)	0 (0.0)	0 (0.0)	0 (0.0)	1 (0.4)	0 (0.0)
Pruritus	0 (0.0)	0 (0.0)	1 (0.7)	0 (0.0)	0 (0.0)	1 (12.5)	0 (0.0)	0 (0.0)	1 (0.4)	1 (0.6)

* MedDRA Version 10.0 was used for IPM 013, IPM 015 and IPM 024. MedDRA Version 15.0 was used for IPM 028 and IPM 034.

** There were no placebo arms in the IPM 028 and 034 trials. For the IPM 028 trial, TEAEs from the DPV ring only arm (Treatment A) are presented. 36 women were enrolled in IPM 028 of which 35 received the DPV vaginal ring in Treatment A.

Table 2: Treatment-Emergent Adverse Events (≥ 5% for either Treatment Group) across Completed Dapivirine Vaginal Ring-004 Trials (continued)

MedDRA Preferred Term*	IPM 013		IPM 015		IPM 024		IPM 028**	IPM 034**	Total	
	Dapivirine (N=36)	Placebo (N=12)	Dapivirine (N=140)	Placebo (N=140)	Dapivirine (N=8)	Placebo (N=8)	Dapivirine (N=35)	Dapivirine (N=40)	Dapivirine (N=259)	Placebo (N=160)
	n (%)	n (%)	n (%)	n (%)	n (%)	n (%)	n (%)	n (%)	n (%)	n (%)
Uterine cervical laceration	***0 (0.0)***	***0 (0.0)***	***0 (0.0)***	***0 (0.0)***	***1 (12.5)***	***0 (0.0)***	***0 (0.0)***	***0 (0.0)***	***1 (0.4)***	***0 (0.0)***
Lymphadenopathy	0 (0.0)	1 (8.3)	0 (0.0)	0 (0.0)	0 (0.0)	0 (0.0)	0 (0.0)	0 (0.0)	0 (0.0)	1 (0.6)
Chlamydial infection	***0 (0.0)***	***1 (8.3)***	***0 (0.0)***	***0 (0.0)***	***0 (0.0)***	***0 (0.0)***	***0 (0.0)***	***0 (0.0)***	***0 (0.0)***	***1 (0.6)***
Dizziness, postural	0 (0.0)	0 (0.0)	0 (0.0)	0 (0.0)	0 (0.0)	1 (12.5)	0 (0.0)	0 (0.0)	0 (0.0)	1 (0.6)
Gastroenteritis, bacterial	0 (0.0)	0 (0.0)	0 (0.0)	0 (0.0)	0 (0.0)	1 (12.5)	0 (0.0)	0 (0.0)	0 (0.0)	1 (0.6)
Hyperventilation	0 (0.0)	0 (0.0)	0 (0.0)	0 (0.0)	0 (0.0)	1 (12.5)	0 (0.0)	0 (0.0)	0 (0.0)	1 (0.6)
Libido, decreased	0 (0.0)	1 (8.3)	0 (0.0)	0 (0.0)	0 (0.0)	0 (0.0)	0 (0.0)	0 (0.0)	0 (0.0)	1 (0.6)
Motion sickness	0 (0.0)	1 (8.3)	0 (0.0)	0 (0.0)	0 (0.0)	0 (0.0)	0 (0.0)	0 (0.0)	0 (0.0)	1 (0.6)
Thermal burn	0 (0.0)	0 (0.0)	0 (0.0)	2 (1.4)	0 (0.0)	1 (12.5)	0 (0.0)	0 (0.0)	0 (0.0)	3 (1.9)

* MedDRA Version 10.0 was used for IPM 013, IPM 015 and IPM 024. MedDRA Version 15.0 was used for IPM 028 and IPM 034.

** There were no placebo arms in the IPM 028 and 034 trials. For the IPM 028 trial, TEAEs from the DPV ring only arm (Treatment A) are presented. 36 women were enrolled in IPM 028 of which 35 received the DPV vaginal ring in Treatment A.

Safety of Extended Use of the Dapivirine Ring-004

Extended use of a single 25 mg DPV Ring-004 (IPM 034), for a period of up to 84 days, was considered generally safe and well tolerated. Only one SAE was reported in a participant who experienced a motor vehicle accident and sustained several thoracic vertebra fractures. No non-serious AEs led to the investigator taking action to permanently discontinue use of the DPV vaginal ring in any participant. The vast majority of AEs were of mild or moderate intensity. Apart from intermenstrual bleeding, the TEAEs reported most frequently (by at least 5.0% of participants using the DPV rings) were nasopharyngitis, lower abdominal pain, headache, vaginal discharge, oropharyngeal pain, nausea, and procedural pain. The only AEs reported in the Reproductive and Breast Disorders System Order Class were grade 1 (mild) vaginal discharge and intermenstrual bleeding.[20]

Conclusion

Considering the lack of significant local and systemic toxicities observed in ongoing and completed trials with vaginally administered DPV and the wide safety margins when comparing systemic exposure from orally administered to vaginally administered DPV, the aforementioned data support a wide safety margin for daily dosing of vaginally administered DPV. The 200 mg DPV ring, which represents an 8-fold increase in drug load when compared to DPV Ring-004, is not anticipated to deliver more DPV than has been demonstrated to be safe and well tolerated in studies with oral DPV. Therefore the proposed loading dose of 200 mg of DPV is unlikely to result in significantly greater local or systemic toxicity than Ring-004 given the 200 mg DPV VR's estimated local and systemic exposure.

2.6 Safety of Levonorgestrel

Levonorgestrel (LNG), a synthetic progestin, has been approved for use in contraceptive products for more than three decades.[20] Jadelle® and Mirena® are long-acting contraceptives that deliver LNG via subdermal implants or intrauterine systems (IUS) respectively approved for contraceptive use whereas Plan-B® is an oral product approved for emergency contraception.

The approved total dosage for a complete regimen of Plan B One-Step® is a single oral dose of 1.5 mg levonorgestrel, whereas the Mirena® IUS is loaded with 52 mg of LNG that is slowly released into the endometrium and is approved for use over 5 years. The Jadelle® implant system is comprised of two contraceptive rods, each loaded with 75 mg of LNG that are placed subdermally and release LNG slowly for up to 5 years of use.

Intrauterine Device

For the LNG IUS, the initial release rate of LNG is 20 µg/day; this rate declines by about 50% after 5 years. A stable plasma level of LNG of 150-200 pg/mL occurs after the first few weeks of use. Levonorgestrel plasma concentrations after 12, 24 and 60 months were approximately 180 ± 66 pg/mL, 192 ± 140 pg/mL and 159 ± 59 pg/mL, respectively.

Subdermal Implant

For the LNG implant, the release rate is estimated to be 100 µg/day at 1 month, declining to about 40 µg/day at 12 months, and stabilizing at a rate of approximately 30 µg/day from 24 months onwards. Maximum plasma concentrations are reached within 2-3 days with the mean ± standard deviation being 772 ± 140 pg/mL at 2 days. After the initial phase, LNG concentrations decline to 435 ± 172 pg/mL at one month, 357 ± 155 pg/mL at 6 months and 280 ± 123 pg/mL at 3 years.

The safety profile for these products is well established and post-marketing experience has not identified any significant safety concerns over a wide dosing range. Given the interspecies comparison of levonorgestrel C_{max} and mean ring release rate and the *in vitro* release rates, it appears that the systemic exposure from the proposed 320 mg loading dose is likely to fall within safety margins established by currently approved products.

Although the safety profile for vaginally administered LNG is less established compared to oral, subdermal, or intrauterine dosing, AEs reported with vaginal delivery of LNG include many AEs also observed during use of LNG subdermal implants.[20]

Vaginal Rings

A large WHO-sponsored trial evaluating a Silastic 382, core design vaginal ring containing 5 mg LNG (20 µg/day release rate) enrolled 1,005 women. The ring was intended to be used continuously for 90 days. The most commonly reported AEs were menstrual disturbances (breakthrough bleeding, prolonged or heavy periods), vaginal discharge, vaginal infection, and vaginitis. Approximately 17.2% of women discontinued the trial early due to menstrual disturbances. There appeared to be no significant trend in the bleeding patterns over one year of continuous use. Users with the worst bleeding patterns tended to discontinue first during the clinical trial and were influenced by their more recent experience of vaginal bleeding irregularities. Although the number of bleeding days was increased in this study, total blood loss decreased after 12 months of use, and hemoglobin levels increased after 6 and 12 months.

CONRAD is currently implementing the A13-128 trial, a *Phase I One-Month Safety, Pharmacokinetic, Pharmacodynamic, and Acceptability Study of Intravaginal Rings Releasing Tenofovir and Levonorgestrel or Tenofovir Alone*. The trial will enroll 100 women from 2 sites in the United States, Eastern Virginia Medical School, Norfolk, VA,

and Profamilia, Santo Domingo, Dominican Republic. Participants are randomized 2:2:1, in the following fashion: tenofovir (10 mg/d) -only ring (n=40), tenofovir/LNG ring (n=40), Placebo ring (n=20). The active vaginal rings are anticipated to release either 8-10 mg of tenofovir only per day, or 8-10 mg of tenofovir and 20 µg of LNG per day. The primary objectives of the trial are genital and systemic safety. The secondary objective of the trial is tenofovir and levonogestrel pharmacokinetics. Results are anticipated Q1 2016.

Safety Summary

In Jadelle® clinical trials where calculated mean *in vivo* release rates of LNG were 100 ug/day at one month declining to approximately 40 ug/day at 12 months, discontinuation rates at one year were 4.5 per 100 women for irregular bleeding. For most women, menstrual irregularities tended to diminish with prolonged use and despite changes in menstrual bleeding patterns, mean hemoglobin levels among Jadelle® users remained unchanged or increased. Experience among users of Norplant® (a subdermal LNG contraceptive system consisting of six capsules) has shown that only in rare cases did menstrual bleeding result in marked decreases in hemoglobin concentration.

The most common adverse reactions (in >5% users) for Mirena®, Jadelle® and Plan B® are similar and include uterine/vaginal bleeding alterations (including amenorrhea, menorrhagia and intermenstrual bleeding), abdominal/pelvic pain, headache/migraine, acne, depressed/altered mood, breast tenderness/pain, vaginal discharge and nausea. Other rare and potentially more serious AEs associated with continued LNG use that have been reported are ectopic pregnancy, ovarian cysts, thrombosis, and idiopathic intracranial hypertension (particularly in obese participants).

Some users of progestin-only oral contraceptives experience a slight deterioration in glucose tolerance, with increases in plasma insulin; however, the effect of levonorgestrel-containing implants on carbohydrate metabolism appears to be minimal. In a Norplant® post marketing surveillance study, there was no significant difference in the development of diabetes mellitus among users of Norplant® compared to women who were using IUDs or who had been sterilized.

A two-year longitudinal study undertaken by the WHO (1999) compared 177 users of Norplant® with a similar number of copper IUD users. Lipid changes were greatest three months after implant insertion, with a slow reversal of these trends during the next 19 months. The report concluded that lipid changes induced by Norplant® would probably not affect the risk of atherosclerotic disease in women who use this contraceptive method. In the WHO trial with a LNG vaginal ring (20 µg daily), no significant differences were observed in lipid/lipoprotein values or in glucose tolerance between the baseline and post removal assessment.

The only consistent change in liver function in users of Jadelle® has been a small increase in total bilirubin, with all mean values remaining within the normal range.

Assessment of kidney function for Jadelle® included an evaluation of blood uric acid, urea nitrogen, sodium, potassium, calcium, and inorganic phosphorous. There were no indications of compromised kidney function.

Some evidence was reported of a minor decrease in thyroxin and triiodothyronine levels in Jadelle® users but this was not accompanied by changes in free thyroxin.

In general, there have been no significant findings from laboratory safety evaluations with LNG products.

Preclinical Studies of Levonorgestrel and LNG-DPV Rings

During manufacture of addition cure silicone elastomer (SE) VRs, LNG is prone to irreversibly react or bind under certain drug, formulation, or processing conditions, unfavorably preventing controlled release as well as recovery of any LNG from VRs. Specifically, *in vitro* release data from testing of VRs containing various loadings of DPV and LNG, LNG-only VRs, as well as additional studies conducted using non-VR samples, showed that the LNG binding phenomenon is observed with addition cure SEs but not condensation cure SEs; the extent of binding depends upon the type of addition cure SE; micronised LNG displays significantly greater binding than non-micronised LNG; and the extent of binding correlates with increased mixing time, cure time, and cure temperature.[22]

2.7 Nonclinical Studies of Levonorgestrel in Combination with Dapivirine

2.7.1 Dapivirine Activity

LNG had no effect on the activity of DPV against a laboratory adapted strain of HIV-1 in an *in vitro* model of cellular infectivity, suggesting that it is unlikely to affect the efficacy of DPV.[20]

2.7.2 Nonclinical Pharmacokinetics

A pharmacokinetic study in sheep was performed in which DPV-only and DPV-LNG combination rings were inserted vaginally for up to 15 days. Vaginal fluid and plasma concentrations were evaluated. Systemic exposure to DPV showed little change with increasing ring load of DPV (Table 3). For rings containing both DPV and LNG, C_{max} was generally similar to values for rings containing DPV alone, but AUC values were higher, although the increase in AUC showed no relationship with the LNG load. In vaginal fluid, C_{max} and AUC values for DPV were higher for rings containing 200 or 530 mg DPV alone than the ring containing 75 mg DPV alone, and higher again for rings that also contained LNG, although the increase showed no relationship with the LNG load. C_{max} and AUC values for LNG in plasma and vaginal fluid increased with increasing ring load of LNG, but the increase was less than proportional to the increase in load.[20] LNG is reported to be ~55% bound to plasma proteins, a substrate for CYP3A4 metabolism and an inhibitor of CYP2B6.

Table 3: Pharmacokinetics of Dapivirine and Levonorgestrel in Plasma and Vaginal Fluid Following Vaginal Administration to Sheep

Ring Load (mg)		Plasma			Vaginal Fluid		
DPV	LNG	C_{max} (pg/mL)	$AUC_{0\text{-}last}$ (ng.h/mL)	T_{max} (h)	C_{max} (ng/mL)	$AUC_{0\text{-}last}$ (ng.h/mL)	T_{max} (h)
**Dapivirine**							
75	-	90.4	25.2	72	1800	34700	6
200	-	103	26.8	12	4000	213000	183
530	-	101	24.6	12	2230	108000	3.5
200	32	122	38.1	24	12500	529000	1
200	120	99.2	30.7	24	12500	615000	1
200	800	102	30.4	6	7780	345000	1
**Levonorgestrel**							
200	32	89.9	21.5	1	7180	42000	1
200	120	210	48.1	2	23400	148000	1
200	800	425	98.5	1	66400	765000	1

In vitro release data for vaginal rings 101, 102, and 104, containing 200 mg DPV with and without LNG, provide a very conservative (i.e. high over-estimate) assessment of the peak daily drug delivery of <8 mg in IPA:water and ~1.5mg in Na-acetate buffer with 2% solutol (Day 1 release). Similar conservative estimates of LNG release from VRs containing 200 mg DPV in combination with 32 mg (Ring-101) and 320 mg (Ring-102) of LNG indicate peak daily drug delivery of ~125 µg and ~400 µg respectively (Day 1 in Na-acetate buffer with 2% solutol).

Since neither DPV nor LNG have demonstrated any toxicity via the vaginal route, there is no basis on which to expect an interaction between the drugs in the combination product that would exacerbate the toxicity of either agent. Local levels of DPV in the vaginal vault may exceed the *in vitro* IC_{50} values for DPV. Competition of dapivirine for type A and B human progesterone receptor is not expected to interfere with progesterone even though dapivirine was found to bind to the recombinant progesterone receptors with a relative binding affinity (compared to progesterone at 100%) of only ~0.1% - 0.2%. LNG is reported to have a relative binding affinity at progesterone receptors of 323%. In light of LNG's approximately 3230-fold higher affinity for progesterone receptors than DPV, it seems highly unlikely that the roughly 2 - 65 fold difference in the vaginal concentrations will result in significant inhibition of LNG binding to local progesterone receptors in vaginal tissues. *In vitro* assessment of LNG's ability to inhibit the anti-HIV activity of DPV indicated that there was no effect of LNG up to the highest soluble concentrations.

Assessed together, these data suggest that it is unlikely that there will be any pharmacodynamic interactions between the two active ingredients that would compromise the efficacy of either. Data on the effects of each drug on cytochrome P450 enzymes also suggest that pharmacokinetic interactions are unlikely. In conclusion, the safety margins established in DPV toxicity studies and the absence of local toxicity following vaginal administration of DPV and LNG to rabbits, along with the established clinical safety of both DPV and LNG, support the use of DPV-LNG VRs in clinical trials.[23]

2.7.3 Nonclinical Toxicity

In rabbits, daily doses of 0.375 mg/mL LNG (2 mL and 0.4 mL dose volume) administered for 10 days resulted in no findings of vaginal irritation or any other local or systemic toxicity that would indicate LNG was not suitable for vaginal administration.

2.8 Rationale for Study Design and Features

2.8.1 Rationale for Inclusion of SMS to Collect Bleeding Data

A controlled trial in which 230 English- and Spanish-speaking women in the New York City area (ages 16-45) were randomized 1:1 to use either daily SMS or paper diaries to report on bleeding experienced during the 90 days after insertion of one of two IUD types (copper or LNG; participants' choice) found that those reporting bleeding via SMS provided more complete data than users of paper diaries. The text group reported a median of 82 days [interquartile range (IQR) 40-89] and the paper group reported a median of 36 days (IQR 0-88) ($p \leq .001$). The number of responses received gradually decreased with time, but was always higher in the text group. Women with higher levels of education did well regardless of modality, while response rates to SMS were greater among women with a high school education or less ($p < .01$).[24]

2.8.2 Study Design

The design of MTN-030/IPM 041, a clinical study of DPV and DPV-LNG VRs in women, will provide data on the PK profile of DPV + LNG when DPV is administered alone, and when combined with LNG, in a VR formulation. This two week study will provide data to inform future clinical trials designed for 90 days of VR use. It is important to note that the dapivirine exposure from the release of the 200 mg DPV VR (Ring-104) is anticipated to fall within pre-established preclinical and clinical safety margins for which vaginally administered data exist. Although it is anticipated that the levels will not exceed those previously identified as safe, close monitoring will be performed over this brief period of planned product use to respond rapidly to any participant safety concerns. Given that this is the first time this VR is being used, study product use will be brief and safety and PK monitoring frequent. It should also be noted that the 200 mg DPV silicone matrix rings planned for this study (Rings 101, 102 and 104) have slightly different physical characteristics than the 25 mg DPV silicone matrix ring (Ring 004) as

follows, respectively: color (white to off-white vs. off-white); weight (7.8 g vs. 8 g); outer diameter (57.1mm vs. 56mm); and cross-sectional diameter (7.9 mm vs. 7.7 mm).[20] MTN-030/IPM 041 will evaluate DPV and LNG levels in both blood and cervicovaginal fluid (CVF) during 14 days of continuous use. PK data will allow for determination of the concentration-time profiles using pooled data across all participants. The study design includes frequent collection of corresponding blood and CVF samples following insertion of a DPV or DPV + LNG VR to allow for detection of burst release. PK parameters of DPV and LNG will be calculated for blood and vaginal fluid. It is important to note that the goal of this study is not to show a comparative difference in safety, but to characterize what AEs are experienced; for this reason a control, or placebo arm, was not included.

Results from this study may support future, more complex study designs that include assessments of markers of contraceptive efficacy and acceptability of LNG when combined with an antiretroviral such as DPV in a VR.

2.8.3 Study Hypotheses

- Blood and CVF DPV and LNG levels will be measureable in all women randomized to DPV and LNG VRs
- Continuous exposure to DPV, or DPV and LNG, via VR for 14 days will be safe

3 OBJECTIVES

3.1 Primary Objectives

Pharmacokinetics

- To characterize the local and systemic pharmacokinetics of one dapivirine vaginal ring formulation and two dapivirine-levonorgestrel vaginal ring formulations used continuously for 14 days

Safety

- To evaluate the safety of one dapivirine vaginal ring formulation and two dapivirine-levonorgestrel vaginal ring formulations used continuously for 14 days

3.2 Secondary Objective

Bleeding

- To describe vaginal bleeding experienced during study participation

3.3 Exploratory Objectives

Acceptability

- To assess the early acceptability of one dapivirine vaginal ring formulation and two dapivirine-levonorgestrel vaginal ring formulations

Adherence

- To evaluate participant adherence to one DPV vaginal ring formulation or two dapivirine-levonorgestrel vaginal ring formulations

Vaginal Microenvironment

- To describe the genital microenvironment in HIV-uninfected women during 14 days of continuous study product use

4 STUDY DESIGN

4.1 Identification of Study Design

MTN-030/IPM 041 is a Phase 1, three-arm, multi-site, double-blind, randomized trial of three silicone elastomer intravaginal rings containing the active ingredient DPV; or a combination of the active ingredients DPV and LNG, formulated with different dose strengths of LNG. The study VR is inserted and worn continuously for a total of approximately 14 days by healthy, HIV-uninfected women age 18-45 (inclusive).

4.2 Primary Endpoints:

Pharmacokinetics

- Dapivirine and levonorgestrel concentrations in blood
- Dapivirine and levonorgestrel concentrations in vaginal fluid

Safety

- Grade 2 or higher genitourinary adverse event as defined by the Division of AIDS (DAIDS) Table for Grading the Severity of Adult and Pediatric Adverse Events, Version 2.0, November 2014, and/or Addendum 1 (Female Genital [Dated November 2007] Grading Table for Use in Microbicide Studies)
- Grade 3 or higher adverse event as defined by the Division of AIDS (DAIDS) Table for Grading the Severity of Adult and Pediatric Adverse Events, Version 2.0, November 2014

4.3 Secondary Endpoint:

Bleeding

- Self-reported vaginal bleeding

4.4 Exploratory Endpoints:

Acceptability

- Self-reported attitudes about ring attributes including single vs. dual-purpose indication and willingness to use this study product in the future.

Adherence

- Frequency of study vaginal ring removal/expulsions (voluntary and involuntary) and duration without the vaginal ring *in situ*
- Drug pharmacokinetic levels
- Residual drug levels in returned vaginal rings

Vaginal Microenvironment

- Changes in microflora

4.5 Description of Study Population

The study population will be healthy, HIV-uninfected women who meet the criteria outlined in Section 5.2 and 5.3.

4.6 Time to Complete Accrual

Accrual is expected to be complete in approximately 8-10 months.

4.7 Study Groups

Approximately 36 females will be randomized in a 1:1:1 ratio to one of the following study groups:

- 200 mg of DPV
- 200 mg of DPV + 32 mg LNG
- 200 mg of DPV + 320 mg LNG

4.8 Expected Duration of Participation

The expected trial duration for each enrolled participant is approximately 16 days.

4.9 Sites

Sites selected by the MTN Executive Committee.

5 STUDY POPULATION

5.1 Selection of the Study Population

The inclusion and exclusion criteria in Sections 5.2 and 5.3 will be utilized to ensure the appropriate selection of study participants.

5.1.1 Recruitment

Participants will be recruited from a variety of sources across sites, including gynecological offices and community-based locations. In addition, participants may be referred to the study from other local research projects and other health and social service providers. Recruitment materials and the site recruitment plan will be approved by site Institutional Review Boards (IRBs) prior to use. Advice regarding these materials will be sought from site community representatives before they are submitted to the IRB for review.

5.1.2 Retention

Once a participant is enrolled/randomized in MTN-030/IPM 041, the study site will make every effort to retain the participants in follow-up to minimize possible bias associated with loss-to-follow-up. An average retention rate of 95% will be targeted at each site. All study sites will be responsible for developing and implementing local standard operating procedures (SOPs) to achieve this. Engaging peer educators/advocates as well as other organizations in retention messaging or other strategies may be used to facilitate MTN-030/IPM 041 retention.

5.2 Inclusion Criteria

Women must meet all of the following criteria to be eligible for inclusion in the study:

1) Age 18 through 45 years (inclusive) at Screening, verified per site SOPs

2) Able and willing to provide written informed consent to be screened for and enrolled in MTN-030/IPM 041

3) Able and willing to provide adequate locator information, as defined in site SOP

4) Able to communicate in spoken and written English

5) Available for all visits and able and willing to comply with all study procedural requirements, including short message service (SMS) requirements

6) Willing to abstain from receptive intercourse (vaginal, oral and finger stimulation) for 24 hours preceding the Enrollment Visit and for the duration of study participation

7) Per participant report, using an effective, non-hormonal method of contraception at Enrollment, and intending to continue the use of an effective, non-hormonal method for the duration of study participation

   Note: MTN-030/IPM 041 defines effective non-hormonal contraception as sterilized (self or partner), non-hormonal (e.g., copper) intrauterine device (IUD) inserted at least 28 days prior to Enrollment, engages in sex exclusively with women, and/or sexually abstinent for the past 90 days and plans to remain abstinent for the duration of study participation

8) In general good health as determined by the Investigator of Record (IoR)/designee at Screening and Enrollment

9) HIV-uninfected based on testing performed at Screening and Enrollment (per protocol algorithm in Appendix II)

10) Regular menstrual cycles of approximately 21 to 35 days duration

11) Intact uterus with at least one ovary

12) Per participant report at Screening and Enrollment, states a willingness to refrain from inserting any <u>non-study</u> vaginal products or objects into the vagina including, but not limited to tampons, spermicides, female condoms, diaphragms, contraceptive VRs, vaginal medications, menstrual cups, cervical caps (or any other vaginally applied barrier method), vaginal douches, lubricants and moisturizers, sex toys (vibrators, dildos, etc.), for 24 hours prior to enrollment and for the duration of study participation.

13) Women over the age of 21 (inclusive) must have documentation of a satisfactory Pap within the past 3 years prior to Enrollment consistent with Grade 0 according to the Female Genital Grading Table for Use in Microbicide Studies Addendum 1 (Dated November 2007) to the DAIDS Table for Grading Adult and Pediatric Adverse Events, Version 2.0, November 2014 or satisfactory evaluation with no treatment required of Grade 1 or higher Pap result

14) At Screening and Enrollment, agrees not to participate in other research studies involving drugs, medical devices, vaginal products, or vaccines after the Screening Visit and for the duration of study participation

5.3 Exclusion Criteria

Women who meet any of the following criteria will be excluded from the study:

1) Body mass index greater than 35 kg/m^2 at Screening

2) Pregnant at Screening or Enrollment or plans to become pregnant during the study period

   Note: A documented negative pregnancy test performed by study staff is required for inclusion; however a self-reported pregnancy is adequate for exclusion from the study.

3) Diagnosed with a urinary tract infection (UTI) or reproductive tract infection (RTI) at Screening or Enrollment

   Otherwise eligible participants diagnosed with UTI/RTI during screening will be offered treatment. If treatment is complete and symptoms have resolved within the 60 day screening window, eligible participants may be enrolled.

4) Diagnosed with an acute sexually transmitted infection requiring treatment per current Centers for Disease Control and Prevention (CDC) guidelines (http://www.cdc.gov/std/treatment/) at Screening or Enrollment such as gonorrhea, chlamydia, trichomonas, pelvic inflammatory disease, and/or syphilis

   Note: Genital warts requiring treatment and frequent reoccurrence of HSV are considered exclusionary; however, infrequent HSV outbreaks are not. Genital warts requiring treatment are defined as those that cause undue burden or discomfort to the participant, including bulky size, unacceptable appearance, or physical discomfort. See MTN-030/IPM 041 SSP Manual for additional information.

5) Has a clinically apparent Grade 2 or higher pelvic examination finding (observed by study staff) at Screening or Enrollment, as per the DAIDS Table for Grading the Severity of Adult and Pediatric Adverse Events, Version 2.0, November 2014, and/or Addendum 1 (Female Genital [Dated November 2007] Grading Table for Use in Microbicide Studies)

   Note: Cervical bleeding associated with speculum insertion and/or specimen collection judged to be within the range of normal according to the clinical judgment of the Investigator of Record (IoR)/designee is considered expected non-menstrual bleeding and is not exclusionary.

   Note: Otherwise eligible participants with exclusionary pelvic examination findings may be enrolled/randomized after the findings have improved to a non-exclusionary severity grading or resolved within 60 days of providing informed consent for screening.

6) Participant report and/or clinical evidence of any of the following:

   a) Known adverse reaction to any of the study products (ever)
   b) Chronic and/or recurrent vaginal candidiasis
   c) Has a contraindication to progestin-only contraceptive method as defined by a category 3 or 4 CDC *U.S. Medical Eligibility Criteria for Contraceptive Use, 2010*[25] condition
   d) Use of hormonal contraception, including hormonal IUD within the 28 days prior to Enrollment
   e) Current chronic use or planned chronic use of antibiotics, corticosteroids, or strong CYP3A inhibitors and inducers
   f) Depot medroxyprogesterone acetate (DMPA) use in the 6 months prior to Enrollment
   g) Non-therapeutic injection drug use in the 12 months prior to Enrollment
   h) Post-exposure prophylaxis (PEP) for HIV exposure within the 6 months prior to Enrollment
   i) Pre-exposure prophylaxis (PrEP) for HIV prevention within the 6 months prior to Enrollment
   j) Last pregnancy outcome less than 90 days prior to Enrollment
   k) Gynecologic or genital procedure (e.g., tubal ligation, dilation and curettage, piercing) 60 days or less prior to Enrollment

      Note: Colposcopy and cervical biopsies for evaluation of an abnormal Pap test as well as IUD insertion/removal are not exclusionary.

   l) Currently breastfeeding or planning to breastfeed during the course of the study
   m) Participation in any other research study involving drugs, medical devices, vaginal products, or vaccines, in the 60 days prior to Enrollment

7) Has any of the following Grade 1 or higher laboratory abnormalities at Screening Visit:
   a) AST or ALT*
   b) Creatinine*
   c) Hemoglobin*

   Note: Otherwise eligible participants with an exclusionary laboratory result may be re-tested and may be enrolled/randomized after the findings have improved to a non-exclusionary severity grading or resolved within 60 days of providing informed consent for screening. Results of safety laboratory testing performed at the Enrollment Visit are expected to be received after the Enrollment Visit, and thus will not be exclusionary. Abnormal results will be noted as pre-existing conditions, and may result in product discontinuation, per IoR discretion as per Section 9.3 of the protocol

8) Has any other condition that, in the opinion of the IoR/designee, would preclude informed consent, make study participation unsafe, complicate the interpretation of

study outcome data, or otherwise interfere with achieving the study objectives including any significant uncontrolled active or chronic medical condition.

*DAIDS Table for Grading the Severity of Adult and Pediatric Adverse Events Version 2.0, November, 2014 and/or Addendum 1 (Female Genital [Dated November 2007] Grading Table for Use in Microbicide Studies)

5.4 Co-enrollment Guidelines

As indicated in Section 5.2 and 5.3, participants must not take part in other research studies involving drugs, medical devices, vaginal products, or vaccines after the Screening Visit and while taking part in MTN-030/IPM 041 unless approved by the Protocol Safety Review Team (PSRT) and Protocol Chair. Participation in the following types of studies may be allowed at the discretion of the IoR/designee after consultation with the Protocol Chair and PSRT:

- Participants may take part in MTN ancillary studies.

Should any participant report concurrent participation in contraindicated studies after enrolling in MTN-030/IPM 041, the IoR/designee will consult the PSRT regarding ongoing product use and other potential safety considerations associated with co-enrollment.

6 STUDY PRODUCT

6.1 Regimen

Each participant will be randomized in a double-blind fashion to one of three study regimens:

Table 4: Study Regimen

Regimen	N	Ring Description
A	12	DPV VR, containing 200 mg DPV (Ring-104)
B	12	DPV-LNG VR, containing 200 mg of DPV + 32 mg LNG (Ring-101)
C	12	DPV-LNG VR, containing 200 mg of DPV + 320 mg LNG (Ring-102)

Each participant will receive a VR containing either 200 mg DPV, 200 mg DPV + 32 mg LNG, or 200 mg DPV + 320 mg LNG. Participants will be randomized in a 1:1:1 ratio. The VR should be worn for approximately 14 consecutive days +/-1 day. The ring will be removed by the participant (or clinician/designee, if necessary) at the Product Use End Visit (PUEV)/Early Termination Visit. The participant will be followed for approximately 2 days following VR removal.

6.2 Administration

At the Enrollment Visit, the VR will be inserted by the participant (or clinician/designee, if necessary). Participants will be given detailed instructions in the clinic on proper VR insertion and removal procedures. Additional details on administration procedures in the event of expulsion or loss and cleaning will be provided to the participant.

Additional details regarding VR administration will be provided in the MTN-030/IPM 041 Study Specific Procedures (SSP) Manual.

6.3 Study Product Formulation

The rings are designed to provide sustained release of drug(s) over a 90-day period. For this first-in-human trial, the rings will only be worn for 14 days +/-1 day.

6.3.1 Dapivirine VR

The DPV silicone elastomer vaginal matrix ring (Ring-104) is a white flexible ring containing 200 mg of DPV dispersed in a platinum-cured DDU-4320 silicone matrix. The dimensions of the ring are 57.1 mm (outer diameter) and 7.9 mm (cross sectional diameter). The DPV silicone elastomer VR is designed to provide sustained release of DPV over a minimum of 90 days.

6.3.2 Dapivirine-Levonorgestrel VRs

The dapivirine-levonorgestrel silicone elastomer vaginal matrix ring is a white flexible ring containing 200 mg of DPV and either 32 mg (Ring-101) or 320 mg (Ring-102) of LNG dispersed in a platinum-cured DDU-4320 silicone matrix. The dimensions of the ring are 57.1 mm (outer diameter) and 7.9 mm (cross sectional diameter). The silicone elastomer VR is designed to provide sustained release of DPV and LNG over a minimum of 90 days.

6.3.3 VR Storage and Dispensing

The recommended storage condition for VRs containing LNG is 2-8°C. Due to the blinded design, all of the VRs will require storage at 2-8°C. Study VRs will be dispensed only to enrolled study participants or clinic staff on behalf of the participant, upon receipt of a written prescription from an authorized prescriber. Dispensation of one VR will take place on the day of enrollment. Provisions for the dispensation of additional VRs will be at the discretion of the IoR, in consultation with the PSRT.

6.4 Supply and Accountability

6.4.1 Supply

IPM (Silver Spring, MD) will oversee the manufacture of all of the study VRs and analysis/release of the rings under Good Manufacturing Practices (GMP).

6.4.2 Accountability

Each Clinical Research Site (CRS) Pharmacist of Record (PoR) is required to maintain a complete record of all study VRs received. The procedures to be followed are provided in the MTN-030/IPM 041 Pharmacy Study Product Management Procedures Manual.

The clinic staff will document all VRs provided to the participants. The clinic staff will also document when the ring is returned/removed. Any VRs not returned must also be documented by the clinic.

6.4.3 Retrieval of Used Study Product

Study participants will be instructed to return for VR removal at the PUEV/Early Termination Visit. In the event that the participant has removed the VR and it is not returned at the PUEV/Early Termination Visit, site staff members will make every effort to encourage participants to return the VR as soon as possible (optimally within 5 working days). Attempts by study staff to retrieve the VR from the participant must be documented. If the VR is not returned within the time frames outlined below, the MTN-030/IPM 041 PSRT must be notified.

When product use is permanently discontinued for HIV infection or pregnancy, the VR must be retrieved (optimally within 24 hours) and returned to the clinic (see table below). Additional VR retrieval specifications in response to discontinuations for other reasons, or IoR instruction, can be found in Table 5. Study product retrieval should occur within the specified timeframe. Attempts should be made by study staff to contact the participant and retrieve the VR as soon as possible when not returned as expected.

Table 5: Retrieval of VR

	Retrieve Study Product
Permanent discontinuation due to potential HIV infection or pregnancy	Within 24 hours
Permanent discontinuation for any other reason or IoR discretion	Within 5 working days

6.5 VR Use Instructions

Participants will receive VR use instructions at the Enrollment Visit and at additional follow-up visits, as needed. Site staff will counsel participants on VR use, including

instruction to refrain from removing the ring (except as directed) and instructions for re-insertion in case of accidental ring expulsion, etc. Additional details will be provided in the MTN-030/IPM 041 SSP Manual. Participants will also be counseled on the use of non-study intravaginal products and other devices as described in Section 6.7.

6.6 Concomitant Medications

Enrolled study participants may use concomitant medications during study participation with the exception of medications and products listed as prohibited. All concomitant medications reported throughout the course of the study will be recorded in the study database. All prescription medications, over-the-counter preparations, vitamins, nutritional supplements, and herbal preparations will be recorded as concomitant medications.

Prohibited Medications

Several concomitant medications/practices will not be permitted. Participants are prohibited from using certain CYP3A inhibitors and inducers. These medications are not recommended because LNG is a CYP3A substrate. It is important to note that single dose oral fluconazole for the treatment of vaginal fungal infections is permitted.

There are potential drug-drug interactions between LNG and antibiotics and corticosteroids, therefore chronic antibiotic and corticosteroid use is prohibited.

A listing of the specific prohibited agents as well as drugs permitted for short-term use (i.e., STI treatment) are provided in the MTN-030/IPM 041 SSP Manual available at www.mtnstopshiv.org. See Section 9.3 for additional information.

6.7 Use of Intravaginal Medications/Products and Practices

All participants will be counseled to avoid the use of non-study intravaginal products and other devices. Other devices include, but are not limited to, spermicides, female condoms, diaphragms, contraceptive intravaginal rings, vaginal medications, menstrual cups, cervical caps, douches, lubricants, and sex toys (e.g., vibrators, dildos, etc.) for the 24 hours preceding the Enrollment Visit and for the duration of study participation. Use of these products will be captured in the study database. Participants who report use of these products during study product use periods will be counseled regarding the use of alternative methods and study staff should reference Section 9.3 for permanent discontinuation guidelines. Tampon use is prohibited for the 24 hours preceding the Enrollment Visit and for the duration of study participation. Participants are expected to be sexually abstinent i.e., no receptive intercourse (vaginal, oral and finger stimulation) for the 24 hours preceding the Enrollment Visit and for the duration of study participation.

7 STUDY PROCEDURES

An overview of the study visit and evaluations schedule is provided in Appendix I. Presented in this section is additional information on visit-specific study procedures. Detailed instructions to guide and standardize procedures across sites as well as to specify the visit windows are provided in the MTN-030/IPM 041 SSP Manual available at www.mtnstopshiv.org.

Figure 2: Study Visit Schedule

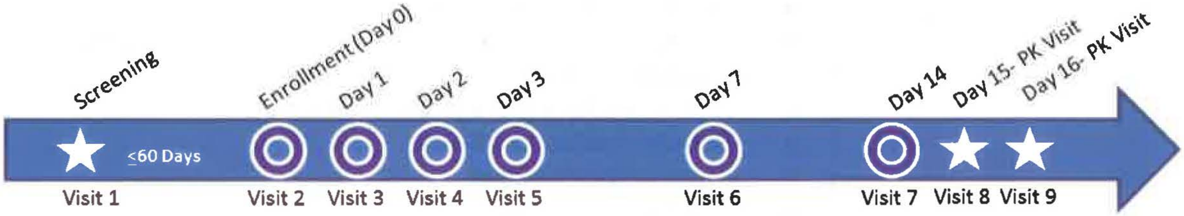

7.1 Pre-screening

As part of participant outreach and recruitment strategies, study staff may pre-screen potential study participants on-site, off-site or over the telephone. During these interactions, study staff may explain the study to potential participants and ascertain elements of presumptive eligibility, to be confirmed at an on-site screening visit. Process information (e.g., number of potential participants contacted, number presumptively eligible) may be recorded and stored at the study site in the absence of written informed consent from potential participants, provided the information is collected in such a manner that it cannot be linked to participant identifiers. At each site, procedures and documentation will comply with local IRB requirements.

7.2 Visit 1 - Screening Visit

A Screening Visit may take place up to 60 days prior to the Enrollment Visit (Day 0). Multiple visits may be conducted to complete all required screening procedures, if necessary. Written informed participant consent for Screening/Enrollment will be obtained at the Screening Visit before any screening procedures are initiated. For participants who do not meet the eligibility criteria, screening will be discontinued once ineligibility is determined.

NOTE: Participants who fail their first screening attempt may be re-screened one time.

Table 6: Visit 1 - Screening Visit

Visit 1 - Screening Visit		
**Component**		**Procedure/Analysis**
**Administrative and Regulatory**		• Obtain Informed consent<br>• Assign a unique Participant Identification (PTID) number<br>• Assess eligibility<br>• Demographic information<br>• Collect locator information<br>• Provide reimbursement<br>• Schedule next visit/contact*
~~**Behavioral/Counseling**~~		• HIV pre- and post-test counseling<br>• HIV/STI risk reduction counseling
**Clinical**		• Medical eligibility information (including exclusionary medical conditions and medications)<br>• Collect medical and menstrual history<br>• Concomitant medications<br>• Physical examination<br>• Pelvic examination<br>• Treatment for RTI, UTI, or STIs*<br>• Disclosure of available test results
**Laboratory**	**Urine**	• hCG<br>• Urine dipstick/culture*
	**Blood**	• ~~HIV-1 testing~~<br>• Serum creatinine<br>• Complete blood count (CBC) with platelets and differential<br>• AST/ALT<br>• Syphilis serology
	**Pelvic Samples**	• NAAT for *Neisseria gonorrhoeae* (GC)/ *Chlamydia trachomatis* (CT)<br>• Test for Trichomonas<br>• Herpes lesion testing*<br>• Pap test^<br>• Saline/KOH wet mount with pH for candidiasis and/or bacterial vaginosis (BV)*
**Study Product Supply**		• Provide condoms*

*If indicated ^ if indicated (if participant [over age 21] is unable to provide documentation of a satisfactory Pap test within 3 years prior to enrollment)

7.3 Visit 2 - Enrollment Visit (Day 0)

The participant's menstrual cycle must be considered when scheduling Visit 2 - Enrollment (Day 0). Ideally, no bleeding occurs during the 14 days of product use.

Table 7: Visit 2 - Enrollment Visit (Day 0)

Visit 2 - Enrollment Visit (Day 0)		
**Component**		**Procedure/Analysis**
**Administrative and Regulatory**		• Assess and confirm eligibility<br>• Review/update locator information<br>• Randomization<br>• Provide reimbursement<br>• Schedule next visit/contact*
**Behavioral/Counseling**		• HIV pre- and post-test counseling<br>• HIV/STI risk reduction counseling<br>• Protocol adherence counseling<br>• Behavioral assessment
**Clinical**		• Medical eligibility information (including exclusionary medical conditions and medications)<br>• Review/update medical and menstrual history<br>• Review/update concomitant medications<br>• Physical examination<br>• Pelvic examination<br>• Digital examination by clinician to check VR placement<br>• Treatment for reproductive tract infection (RTI)/urinary tract infection (UTI), or STIs*<br>• Disclosure of available test results
**Laboratory**	**Urine**	• hCG<br>• Urine dipstick/culture*
	**Blood**	• HIV-1 testing<br>• Serum creatinine<br>• Complete blood count (CBC) with platelets and differential<br>• AST/ALT<br>• Plasma archive<br>• DPV levels∞<br>• LNG levels∞<br>• Sex hormone-binding globulin (SHBG) and albumin<br>• Serum progesterone and estradiol
	**Pelvic Samples**	• Test for Trichomonas*<br>• NAAT for GC/CT*<br>• Saline/KOH wet mount with pH for candidiasis and/or BV*<br>• Herpes lesion testing*<br>• Vaginal Gram stain<br>• CVF DPV levels∞<br>• CVF LNG levels∞
**Study Product Supply**		• Provision of one study VR and VR use instructions<br>• Insertion of the provided study VR

* If indicated, ∞=See Table 12 for additional details on sample collection

7.4 Follow-up Visits

7.4.1 Visits 3-5: Day 1, Day 2, Day 3

Table 8: Visits 3-5: Day 1, Day 2, Day 3 Study Follow-up Visits

Visits 3-5: Day 1, Day 2, Day 3 Study Follow-up Visits		
**Component**		**Procedure/Analysis**
**Administrative and Regulatory**		• Review/update locator information<br>• Provide reimbursement<br>• Schedule next visit/contact
**Behavioral/Counseling**		• HIV pre- and post-test counseling*<br>• HIV/STI risk reduction counseling*<br>• Protocol adherence counseling*<br>• Collect product use information
**Clinical**		• Review/update medical and menstrual history<br>• Review/update concomitant medications<br>• Modified physical examination*<br>• Pelvic examination*(Day 3 mandatory)<br>• Treatment for RTI, UTI, or STIs*<br>• Disclosure of available test results<br>• Collect AEs
**Laboratory**	**Urine**	• hCG*
	**Blood**	• HIV-1 testing*<br>• Serum creatinine*<br>• CBC with platelets and differential*<br>• Syphilis serology*<br>• DPV levels∞<br>• LNG levels∞
	**Pelvic Samples**	• NAAT for GC/CT*<br>• Test for Trichomonas*<br>• Saline/KOH wet mount with pH for candidiasis and/or BV*<br>• Herpes lesion testing*<br>• Vaginal Gram stain* (Day 3 mandatory)<br>• CVF DPV levels∞<br>• CVF LNG levels∞

* If indicated, ∞=See Table 12 for additional details on sample collection

7.4.2 Visit 6 – Day 7

Table 9: Visit 6 – Day 7

Visit 6 – Day 7		
**Component**		**Procedure/Analysis**
**Administrative and Regulatory**		• Review/update locator information<br>• Provide reimbursement<br>• Schedule next visit/contact
**Behavioral/Counseling**		• HIV pre- and post-test counseling*<br>• HIV/STI risk reduction counseling*<br>• Protocol adherence counseling<br>• Collect product use information
**Clinical**		• Review/update medical and menstrual history<br>• Review/update concomitant medications<br>• Modified physical examination*<br>• Pelvic examination<br>• Treatment for RTI, UTI, or STIs*<br>• Disclosure of available test results<br>• Collect AEs
**Laboratory**	**Urine**	• hCG*
	**Blood**	• HIV-1 testing*<br>• Serum creatinine*<br>• CBC with platelets and differential*<br>• Syphilis serology*<br>• DPV levels∞<br>• LNG levels∞
	**Pelvic Samples**	• Vaginal Gram stain*<br>• NAAT for GC/CT*<br>• Test for Trichomonas*<br>• Saline/KOH wet mount with pH for candidiasis and/or BV*<br>• Herpes lesion testing*<br>• CVF DPV levels∞<br>• CVF LNG levels∞

* If indicated, ∞=See Table 12 for additional details on sample collection

7.4.3 Visit 7 – Day 14: PUEV/Early Termination Visit Ring Removal

Table 10: Visit 7 – Day 14: PUEV/Early Termination Visit

Visit 7 – Day 14: PUEV/Early Termination Visit		
**Component**		**Procedure/Analysis**
**Administrative and Regulatory**		• Review/update locator information<br>• Provide reimbursement<br>• Schedule next visit/contact
**Behavioral/Counseling**		• HIV pre- and post-test counseling<br>• HIV/STI risk reduction counseling<br>• Protocol adherence counseling<br>• Collect product use information<br>• Behavioral assessment
**Clinical**		• Review/update medical and menstrual history<br>• Review/update concomitant medications<br>• Modified physical examination<br>• Pelvic examination<br>• Treatment for RTI, UTI, or STIs*<br>• Disclosure of available test results<br>• Collect AEs
**Laboratory**	**Urine**	• hCG*
	**Blood**	• HIV-1 testing<br>• CBC with platelets and differential<br>• AST/ALT<br>• Serum creatinine<br>• Syphilis serology*<br>• Sex hormone-binding globulin (SHBG) and albumin<br>• DPV levels∞<br>• LNG levels∞
	**Pelvic Samples**	• NAAT for GC/CT*<br>• Test for Trichomonas*<br>• Saline/KOH wet mount with pH for candidiasis and/or BV*<br>• Herpes lesion testing*<br>• Vaginal Gram stain<br>• CVF DPV levels∞<br>• CVF LNG levels∞
**Study Product Supply**		• Removal and collection of study VR<br>• Provide condoms*

* If indicated, ∞=See Table 12 for additional details on sample collection

7.4.4 Visit 8 and 9 – Day 15 and 16

Table 11: Visit 8 and 9 – Day 15 and 16

Visit 8 and 9 – Day 15 and 16		
**Component**		**Procedure/Analysis**
**Administrative and Regulatory**		• Review/update locator information<br>• Provide reimbursement<br>• Schedule next visit/contact* (Required at Day 15 Visit)
**Behavioral/Counseling**		• HIV pre- and post-test counseling*<br>• HIV/STI risk reduction counseling*<br>• Protocol adherence counseling*
**Clinical**		• Pelvic examination*<br>• Review/update medical and menstrual history<br>• Review/update concomitant medications<br>• Treatment for RTI, UTI, or STIs*<br>• Disclosure of available test results<br>• Collect AEs
**Laboratory**	**Urine**	• hCG* (Required at Day 16 Visit)
	**Blood**	• DPV levels∞<br>• LNG levels∞<br>• AST/ALT*<br>• HIV-1 testing*<br>• Serum creatinine*<br>• CBC with platelets and differential*<br>• Syphilis serology*
	**Pelvic Samples**	• NAAT for GC/CT*<br>• Test for Trichomonas*<br>• Saline/KOH wet mount with pH for candidiasis and/or BV*<br>• Vaginal Gram stain*<br>• Herpes lesion testing*<br>• CVF DPV levels∞<br>• CVF LNG levels∞

* If indicated, ∞=See Table 12 for additional details on sample collection

7.5 Follow-up Procedures for Participants Who Permanently Discontinue Study Product

7.5.1 Participants Who Become Infected with HIV-1

If a participant tests positive for HIV-1 after randomization at the Enrollment Visit, she will be referred to local care and treatment services and may return to the research clinic for additional counseling and other support services, as needed. Continued study participation would be of no added benefit, thus follow-up visits will be discontinued, study product use will cease, and the participant will be considered terminated from the study. Participants who become infected with HIV after randomization may be offered additional laboratory testing (such as HIV RNA and HIV drug resistance testing), as

clinically indicated per discussions between IoR and LC. Please reference the MTN-030/IPM 041 SSP Manual for additional details (www.mtnstopshiv.org).

7.5.2 Participants Who Become Pregnant

If a participant becomes pregnant, she will be referred to local health care services and may return to the research clinic for additional counseling, as needed. Continued study participation would be of no added benefit to the participant, thus follow-up visits and procedures will be discontinued and the participant will be considered terminated from the study. A participant who is pregnant at study termination will continue to be followed until the pregnancy outcome is ascertained, see Section 9.8 for additional details. For additional details regarding obtaining pregnancy outcome, please reference the MTN-030/IPM 041 SSP Manual (www.mtnstopshiv.org).

Participants who become pregnant while on study product may be offered enrollment in MTN-016 (www.mtnstopshiv.org), provided their study site is taking part in MTN-016.

7.5.3 Participants Who Permanently Discontinue Study Product for Other Reasons

In the event of permanent discontinuation of study product use, participants will be asked to complete an interim visit where all of the study procedures scheduled to occur at Visit 7-Day 14-PUEV/Early Termination Visit will be conducted. These participants will then be asked to continue the visit schedule with modified procedures, as described below.

Protocol-specified procedures will continue except the following:

- Pelvic exams*
- Collection of blood for safety assessments*
- Collection of samples for PK and PD
- Behavioral assessments
- Product use data collection
- Protocol-required counseling will be modified

*Unless required for AE follow-up

Note: The MTN-030/IPM 041 Management Team, in consultation with the MTN Pharmacology Core, may provide alternative guidance to the site regarding a modified study visit schedule, in an effort to ensure that PK samples are collected at the appropriate time points and/or omitted if the collection of samples would not be anticipated to yield analyzable data. Participants' duration of use and timing of study product permanent discontinuation will be factored into a modified schedule. See MTN-030/IPM 041 SSP Manual for additional details.

Site investigators may, after consultation with the PSRT and MTN-030/IPM 041 Management Team, decide to discontinue study follow-up visits and procedures. However, participants who permanently discontinue study product use due to an AE must continue to be followed until the resolution or stabilization of the AE is documented.

7.6 Interim Visits

Interim visits may be performed at any time during the study and any procedures may be conducted. All interim contacts and visits will be documented in participants' study records. If a participant misses a visit (e.g., presents to the clinic outside of the visit window), she can return for an interim visit to have the specimens collected. See MTN-030/IPM 041 SSP Manual for additional details.

7.7 Pharmacokinetics

The entire MTN-030/IPM 041 cohort will provide blood and CVF for PK at Visits 2-9.

Detailed instructions are provided in the MTN-030/IPM 041 SSP Manual available at http://www.mtnstopshiv.org.

Table 12: PK Specimen Collection Schedule

Visit	Specimens Collected for PK (Blood and CVF)
Visit 2: Enrollment (Day 0)	• Blood for DPV level (Baseline & Hours 1, 2, 4, 6)<br>• Blood for LNG level (Baseline & Hours 1, 2, 4, 6)<br>• CVF for DPV level (Baseline & Hours 1, 2, 4, 6)<br>• CVF for LNG level (Baseline & Hours 1, 2, 4, 6)
Visits 3-5: Day 1, Day 2, Day 3 Study Visits	• Blood for DPV level<br>• Blood for LNG level<br>• CVF for DPV level<br>• CVF for LNG level
Visit 6: Day 7	• Blood for DPV level<br>• Blood for LNG level<br>• CVF for DPV level<br>• CVF for LNG level
Visit 7: Day 14	• Blood for DPV level (Prior to ring removal and at Hour 6)<br>• Blood for LNG level (Prior to ring removal and at Hour 6)<br>• CVF for DPV level (Prior to ring removal and at Hour 6)<br>• CVF for LNG level (Prior to ring removal and at Hour 6)
Visit 8: Day 15<br>Visit 9: Day 16	• Blood for DPV level<br>• Blood for LNG level<br>• CVF for DPV level<br>• CVF for LNG level

7.8 Behavioral Assessments

Behavioral Assessment

The behavioral measures used in this protocol will focus on assessing participants' experiences with inserting, wearing and removing the ring. At baseline, initial acceptability of the VRs will be assessed. The baseline behavioral assessment may also assess participants' prior clinical trial experience, including experience with VR trials as well as questions on prior contraceptive use. Adherence to the protocol requirements over 14 days of continuous use will be assessed. A follow-up behavioral assessment will assess participants' experiences with the trial, including acceptability of and adherence to the ring.

Vaginal Bleeding and Adherence Assessment

Daily short message service (SMS) will be employed as a measure to monitor vaginal bleeding and product adherence.

Participants will also be asked to reply to questions regarding ring adherence at every study visit during the study product use period. Questions regarding bleeding will be asked at each follow-up visit.

Data on self-reports of ring use and vaginal bleeding sent through SMS and captured via CRFs will be made available to the site counselor and/or clinician for comparison via one-on-one data convergence interviews with study participants. A Data Convergence Interview will be conducted on Visit 6: Day 7, Visit 7: Day 14, and Visit 9: Day 16 (bleeding only), to assess any discrepancies between ring adherence and vaginal bleeding data collected via SMS and on the CRFs. The counselor/clinician will review the information with the participant to elicit information about possible discrepancies. The counselor's/clinician's approach will be non-judgmental, reminding the participant that, regardless of her level of ring use or vaginal bleeding, she will not be disqualified from the study. This analysis of data on the same topic emanating from different sources is generally referred to as research triangulation.[26] This process will allow for clarification of discrepancies between data sources, and will be more informative than any single data source taken alone. All the independent data sources will be available to allow for analysis of different estimates of ring adherence and vaginal bleeding.

In the rare case in which a participant attends the visit but does not stay to speak with a counselor, we will analyze the available SMS and CRF data.

The available SMS and CRF data, along with the counselor's/clinician's converged assessment of the most likely ring adherence and vaginal bleeding, will constitute the summary database on ring adherence and vaginal bleeding.

7.9 Clinical Evaluations and Procedures

Physical exams will include the following assessments:

- General appearance
- Weight
- Vital signs
    - Temperature
    - Pulse
    - Blood pressure
    - Respirations
- Height**
- Waist measurement**
- Lymph nodes*
- Abdomen*
- Neck*
- Heart*
- Lungs*
- Extremities*
- Skin*
- Neurological*

**may be omitted after the Screening Visit
*may be omitted after the Enrollment Visit

Additional clinical assessments may be performed at the discretion of the examining clinician in response to symptoms or illnesses present at the time of the examination.

Pelvic Examination and Specimen Collection
Pelvic examinations will be conducted per guidelines for naked eye inspection described in the WHO/CONRAD Manual for Standardization of Colposcopy for the Evaluation of Vaginal Products, Update 2004, available at http://www.conrad.org/publications-13.html.

The required sequence of procedures and specimen collection performed during pelvic exams will be specified in the MTN-030/IPM 041 SSP Manual.

7.10 Laboratory Evaluations

Local Laboratory

- Urine
    - Urine hCG
    - Dipstick UA and/or urine culture

- Blood
  - Serum creatinine
  - AST/ALT
  - Complete blood count with platelets and differential
  - HIV-1 testing
  - Syphilis serology
  - Sex hormone-binding globulin (SHBG) and albumin
  - Serum progesterone and estradiol

- Pelvic
  - Trichomonas test
  - Pap test
  - Saline/KOH wet mount with pH for candidiasis and/or BV
  - NAAT for GC/CT
  - Herpes lesion testing

Network Laboratory Center (LC)

- Blood
  - DPV levels
  - LNG levels
  - Confirmation HIV-1 testing for seroconversion
  - HIV-1 resistance tests for confirmed seroconverters
  - Plasma archive

- Pelvic
  - Cervicovaginal fluid for DPV and LNG levels
  - Gram stain of vaginal smear

IPM Designated Laboratory

- Study Product
  - Used study VR residual drug level assessment

7.11 Specimen Management

Each study site will adhere to the standards of good clinical laboratory practice (https://www.niaid.nih.gov/LabsAndResources/resources/DAIDSClinRsrch/Documents/gclp.pdf), in accordance with current DAIDS Laboratory Requirements, MTN-030/IPM 041 Study Specific Procedures Manual (http://www.mtnstopshiv.org/studies) and site standard operating procedures for proper collection, processing, labeling, transport, and storage of specimens to standardize procedures. Specimen collection, testing, and storage at the site laboratories will be documented when applicable using the Laboratory Data Management System (LDMS). In cases where laboratory results are not available due to administrative or laboratory error, sites are permitted to re-draw specimens. Further, as part of quality control, researchers may need to look at short

pieces of non-coding repetitive DNA sequence (3-7 base pairs) from blood in the event of sample mix-up. This test will only let researchers know the number of times this short segment is repeated and not specific genes or specific sequences of base pairs. This sequence element does not contain any information about genes, and therefore researchers will not be able to identify if participants are predisposed to specific diseases or any other genetic information based on this information. This test will be an important tool for distinguishing whether two samples collected at the same or different time points are likely from the same person. The test will only be used as part of a sample investigation with the knowledge of the site in situations where a known or suspected sample mix-up has occurred. No genetic testing (limited or genome-wide) is planned on leftover samples that are stored for the purposes of future research.

7.12 DAIDS Laboratory Oversight

All laboratories participating in DAIDS Sponsored and/or Funded Laboratories in Clinical Trials will adhere to the DAIDS Laboratory Policy. (http://www.niaid.nih.gov/labsandresources/resources/daidsclinrsrch/documents/labpolicy.pdf)

7.13 Biohazard Containment

As the acquisition of HIV and other bloodborne pathogens can occur through contact with contaminated needles, blood, and blood products, appropriate blood and secretion precautions will be employed by all personnel in the drawing of blood and shipping and handling of all specimens for this study as recommended by the CDC and National Institutes of Health (NIH). All biological specimens will be transported using packaging mandated by Code of Federal Regulations (CFR) 42 Part 72. All dangerous goods materials, including diagnostic specimens and infectious substances, must be transported according to instructions detailed in the International Air Transport Association (IATA) Dangerous Goods Regulations. Biohazardous waste will be contained according to institutional, transportation/carrier, and all other applicable regulations.

8 ASSESSMENT OF SAFETY

8.1 Safety Monitoring

Site IoRs/designees are responsible for continuous close safety monitoring of all study participants, and for alerting the Protocol Team if unexpected concerns arise. A sub-group of the Protocol Team, including the Protocol Co-Chairs, DAIDS Medical Officer, Protocol Safety Physician(s), and IPM Safety Physician(s) will serve as the Protocol Safety Review Team (PSRT). The MTN SDMC prepares routine AE and clinical data reports for review by the PSRT, which meets via conference call approximately once

per month or as needed throughout the period of study implementation to review safety data, discuss product use management, and address any potential safety concerns.

8.2 Clinical Data and Safety Review

A multi-tiered safety review process will be followed for the duration of this study. The study site investigators are responsible for the initial evaluation and reporting of safety information at the participant level and for alerting the PSRT if unexpected concerns arise. Participant safety is also monitored at the Network level through a series of routine reviews conducted by the SDMC, the PSRT and study sponsors. Additional reviews may be conducted at each of these levels as dictated by the occurrence of certain events.

MTN SDMC staff will review incoming safety data on an ongoing basis. Events identified as questionable, inconsistent, or unexplained will be queried for verification. AE reports submitted in an expedited manner to the DAIDS Safety Office will be forwarded to the DAIDS Medical Officer and SDMC Clinical Affairs staff, and the IPM safety physician for review.

The PSRT will meet approximately every month, or as needed, via conference call to review clinical data reports generated by the MTN SDMC. The content, format and frequency of the clinical data reports will be agreed upon by the PSRT and the SDMC in advance of study implementation. In addition to the routine safety data reviews, the PSRT will convene on an ad hoc basis to make decisions regarding the handling of any significant safety concerns. If necessary, experts external to the MTN representing expertise in the fields of microbicides, biostatistics, HIV acquisition and medical ethics may be invited to join the PSRT safety review. A recommendation to pause or stop the trial may be made by the PSRT at this time or at any such time that the team agrees that an unacceptable type and/or frequency of AEs has been observed.

The Study Monitoring Committee (SMC) will review participant safety data as part of their regular reviews (see Section 10.8.1), since no Data and Safety Monitoring Board oversight is planned for MTN-030/IPM 041. The SMC may recommend that the study proceed as designed, proceed with design modifications, or be discontinued. Members of the SMC will be independent investigators with no interest (financial or otherwise) in the outcomes of this study. If at any time a decision is made to discontinue enrollment and/or study product use in all participants, IPM will notify the FDA and the Site IoR will notify the responsible IRB expeditiously.

In addition to the safety monitoring done by the PSRT, the MTN SMC will conduct interim reviews of study progress, including rates of participant accrual, retention, completion of primary and main secondary endpoint assessments, and study or lab issues. These reviews will take place approximately every 4-6 months, or as needed. At the time of these reviews, or at any other time, the SMC may recommend that the study proceed as designed, proceed with design modifications, or be discontinued.

8.3 Adverse Events Definitions and Reporting Requirements

8.3.1 Adverse Events

An AE is defined as any untoward medical occurrence in a clinical research participant administered an investigational product and which does not necessarily have a causal relationship with the investigational product. As such, an AE can be an unfavorable or unintended sign (including an abnormal laboratory finding, for example), symptom or disease temporally associated with the use of an investigational product, whether or not considered related to the product. This definition is applied to all study groups, and is applied to all groups beginning at the time of enrollment (i.e., once a participant is randomized) through the termination visit. The term "investigational product" for this study refers to all study products.

Study participants will be provided instructions for contacting the study site to report any untoward medical occurrences they may experience. In cases of potentially life-threatening events, participants will be instructed to seek immediate emergency care. Where feasible and medically appropriate, participants will be encouraged to seek evaluation where a study clinician is based, and to request that the clinician be contacted upon their arrival. With appropriate permission of the participant, whenever possible, records from all non-study medical providers related to untoward medical occurrences will be obtained and required data elements will be captured in the study database. All participants reporting an untoward medical occurrence will be followed clinically until the occurrence resolves (returns to baseline) or stabilizes.

Study site staff will document in source documents and in the study database all AEs reported by or observed in enrolled study participants, regardless of severity and presumed relationship to study product. AEs will be graded per the DAIDS Table for Grading the Severity of Adult and Pediatric Adverse Events, Version 2.0, November 2014, and Addendum 1 (Female Genital [Dated November 2007]) Grading Table for Use in Microbicide Studies.

In cases where a genital AE is covered in multiple tables, the Female Genital Grading Table for Use in Microbicide Studies will be the grading scale utilized.

Please note:

- Asymptomatic BV and asymptomatic candidiasis will not be reportable AEs;
- Fetal losses (e.g., spontaneous abortions, spontaneous fetal deaths, stillbirths) will not be reported as AEs;
- Untoward maternal conditions that either result in or result from fetal losses are reported as reproductive system AEs;
- Changes in genital bleeding will be collected as data, but will not be considered an AE unless deemed to be an SAE.

8.3.2 Serious Adverse Events

SAEs will be defined as in the Manual for Expedited Reporting of Adverse Events to DAIDS (Version 2.0, January 2010), as AEs occurring at any dose that:

- Result in death
- Are life-threatening
- Result in persistent or significant disability/incapacity
- Are congenital anomalies/birth defects
- Require inpatient hospitalization or prolongation of existing hospitalization
  Note: Per ICH SAE definition, hospitalization itself is not an AE, but is an outcome of the event. Thus, hospitalization in the absence of an AE is not regarded as an AE, and is not subject to expedited reporting. The following are examples of hospitalization that are not considered to be AEs:
    - Protocol-specified admission (e.g., for procedure required by study protocol)
    - Admission for treatment of target disease of the study, or for pre-existing condition (unless it is a worsening or increase in frequency of hospital admissions as judged by the clinical investigator)
    - Diagnostic admission (e.g., for a work-up of an existing condition such as persistent pretreatment lab abnormality)
    - Administrative admission (e.g., for annual physical)
    - Social admission (e.g., placement for lack of place to sleep)
    - Elective admission (e.g., for elective surgery)

Important medical events that may not result in death, be life-threatening, or require hospitalization may be considered a serious adverse drug experience when, based upon appropriate medical judgment, they may jeopardize the patient or subject and may require medical or surgical intervention to prevent one of the outcomes listed above.

8.3.3 Adverse Event Relationship to Study Product

Relatedness is an assessment made by a study clinician of whether or not the event is related to the study agent. Degrees of relatedness will be categorized according to current DAIDS-approved guidelines. Per the Manual for Expedited Reporting of Adverse Events to DAIDS (Version 2.0, January 2010), the relationship categories that will be used for this study are:

- *Related:* There is a reasonable possibility that the AE may be related to the study agent(s)
- *Not Related:* There is not a reasonable possibility that the AE is related to the study agent(s)

8.4 Adverse Event Reporting Requirements

8.4.1 Expedited Adverse Event Reporting to DAIDS

Requirements, definitions and methods for expedited reporting of adverse events are outlined in Version 2.0 of the DAIDS EAE Manual, which is available on the DAIDS RSC website at http://rsc.tech-res.com/safetyandpharmacovigilance.

The DAIDS Adverse Experience Reporting System (DAERS), an internet-based reporting system, must be used for EAE reporting to DAIDS. In the event of system outages or technical difficulties, EAEs may be submitted using the DAIDS EAE Form. This form is available on the DAIDS RSC website at http://rsc.tech-res.com/safetyandpharmacovigilance/.

For questions about DAERS, please contact NIAID CRMS Support at CRMSSupport@niaid.nih.gov. Please note that site queries may also be sent from within the DAERS application itself.

For questions about expedited reporting, please contact the DAIDS RSC Safety Office at (DAIDSRSCSafetyOffice@tech-res.com).

8.4.2 Reporting Requirements for this Study

- The SAE Reporting Category, as defined in Version 2.0 of the DAIDS EAE Manual, will be used for this study
- The study agents for which expedited reporting is required are the DPV VR and the DPV-LNG VRs

8.4.3 Grading Severity of Events

The grading of severity of events and the reporting period will be the same as for all AEs, as described in Section 8.3.1. The most current Division of AIDS Table for Grading Adult and Pediatric Adverse Events, Version 2.0, November 2014 and the Female Genital Grading Table for Use in Microbicide Studies (Addendum 1 to the DAIDS Table for Grading the Severity of Adult and Pediatric Adverse Events, Version 1.0, November 2007), will be used and are available on the RSC website at http://rsc.tech-res.com/safetyandpharmacovigilance/gradingtables.aspx.

8.4.4 Expedited AE Reporting Period

The expedited AE reporting period for this study begins at enrollment and continues through the participant's termination from the study.

After the protocol-defined AE reporting period, unless otherwise noted, only suspected, unexpected serious adverse reactions (SUSARs) as defined in Version 2.0 of the EAE

Manual will be reported to DAIDS if the study staff become aware of the events on a passive basis (from publicly available information).

8.5 Pregnancy and Pregnancy Outcomes

Pregnant women are excluded from this study.

A participant who is pregnant after enrollment will continue to be followed until the pregnancy outcome is ascertained; see Section 9.8 for additional details. Pregnancy outcomes will not be expeditiously reported to IPM or the DAIDS Medical Officer (MO) unless there is an associated AE in the pregnant participant that meets expedited reporting criteria or the pregnancy results in a congenital anomaly meeting the Manual for Expedited Reporting of EAEs to DAIDS (Version 2.0, January 2010) guidelines for expedited reporting.

8.6 Regulatory Requirements

Information on all reported AEs will be included in reports to the FDA and other applicable government and regulatory authorities. Site IoRs/designees will submit AE and any relevant safety information in accordance with local regulatory requirements.

8.7 Social Harms Reporting

Although study sites make every effort to protect participant privacy and confidentiality, it is possible that participants' involvement in the study could become known to others and that social harms may result. Social harms that are judged by the IoR/designee to be serious or unexpected will be reported to the PSRT and responsible site IRBs according to their individual requirements.

9 CLINICAL MANAGEMENT

Guidelines for clinical management and permanent discontinuation of study product are outlined in this section. In general, the IoR/designee has the discretion to discontinue study product use at any time if s/he feels that continued product use would be harmful to the participant or interfere with treatment deemed clinically necessary. Unless otherwise specified below, the IoR/designee must immediately notify the PSRT of permanent discontinuation of study product. The IoR/designee will document all permanent discontinuations on applicable CRFs.

9.1 Grading System

AE severity grading is described in Section 8.4.3.

9.2 Dose Modification Instructions

No dose modifications will be undertaken in this study.

9.3 General Criteria for Permanent Discontinuation of Study Product

Participants will be permanently discontinued from VR product use by the IoR/designee for any of the following reasons:

- Acquisition of HIV-1 infection; such participants will not resume product use at any time. The study VR must be discontinued immediately upon recognition of the first reactive rapid HIV test.
- Allergic reaction to the VR
- Pregnancy
- Breastfeeding
- Reported use of PEP for HIV exposure
- Reported use of PrEP for HIV prevention
- Non-therapeutic injection drug use
- Study VR has been out of vagina for more than 3 consecutive days
- Participant reports the use of prohibited medications as listed in Section 6.6 and further clarified in the MTN-030/IPM 041 SSP Manual.
- Participant is unable or unwilling to comply with required study procedures, or otherwise might be put at undue risk to their safety and well-being by continuing product use (e.g., changes in safety laboratories between Screening and Enrollment) according to the judgment of the IoR/designee.

9.4 Response to Adverse Events

Grade 1 or 2
In general, a participant who develops a Grade 1 or 2 AE not specifically addressed below, regardless of relationship to study product, may continue product use.

Grade 3
Participants who develop a Grade 3 AE not specifically addressed below, judged by the IoR/designee to be not related to study product, may continue product use.

If a participant develops a Grade 3 AE not specifically addressed below and the AE is judged by the IoR/designee to be related to study product, the IoR/designee must permanently discontinue study product use.

Grade 4
Participants who develop a Grade 4 AE (regardless of relationship to study product) not specifically addressed below, must have the study product permanently discontinued.

9.5 Sexually Transmitted Infection/Reproductive Tract Infection

The IoR/designee must manage STI/RTI per current CDC guidelines, available at http://www.cdc.gov/std/treatment/.

VR use need not be held in the event of an STI/RTI requiring treatment, unless other permanent discontinuation guidelines described below apply. Should the IoR/designee determine that a permanent discontinuation is warranted due to an STI or RTI, consultation with the PSRT is required.

9.6 Management of Specific Genital Events

If a suspected finding is reported by the participant between scheduled visits, an interim visit may be scheduled at the discretion of the site investigator. Management of genital events observed at scheduled or interim visits will be in accordance with the following:

Superficial epithelial disruption or localized erythema or edema: area of less than 50% of vulvar surface or combined vaginal and cervical surface

- Continue study VR use (at study clinician's discretion)
- Perform naked eye evaluation
- Re-evaluate by speculum examination in approximately 3-5 days
- If condition worsens or does not resolve at that time, permanently discontinue study VR use

Deep epithelial disruption (ulceration) or generalized erythema or severe edema: area of more than 50% of vulvar surface or combined vaginal and cervical surface affected by erythema or severe edema

- Permanently discontinue study VR use

Unexpected genital bleeding

- Continue study VR use (at study clinician's discretion)
- Perform naked eye evaluation
- If determined to be due to deep epithelial disruption, refer to guidelines above; otherwise continue study VR use

Genital petechia(e) and genital ecchymosis

- Continue study VR use (at study clinician's discretion)
- Perform naked eye evaluation
- No further evaluation or treatment is required

9.7 HIV-1 Infection

Participants who test positive for HIV-1 must have study product permanently discontinued by the IoR/designee. A participant who is confirmed to be HIV-1 positive

during the course of the study will have study product discontinued, all follow-up visits will be discontinued and the participant will be considered terminated from the study, as per Section 7.5.1. Guidance regarding management and referral for participants confirmed to be HIV-positive is located in Section 13.11.

9.8 Pregnancy

Pregnancy testing will be performed at designated study visits and participants will be encouraged to report all signs or symptoms of pregnancy to study staff. The IoR/designee will counsel any participant who becomes pregnant regarding possible risks to the fetus according to site SOPs. The IoR/designee also will refer the participant to all applicable services; however, sites will not be responsible for paying for pregnancy-related care.

A participant who becomes pregnant during the course of the study will have study product discontinued and will be terminated from the study, as per Section 7.5.2. A participant who is pregnant at study termination will continue to be followed until the pregnancy outcome is ascertained (or, in consultation with the PSRT, it is determined that the pregnancy outcome cannot be ascertained). Pregnancy outcomes will be reported on relevant CRFs; outcomes meeting criteria for EAE reporting also will be reported on EAE forms.

A participant who becomes pregnant during the course of study participation may be offered participation in MTN-016, HIV Prevention Agent Pregnancy Exposure Registry: EMBRACE Study, at sites participating in MTN-016. This registry study captures pregnancy outcomes as well as infant health information, (including growth), to evaluate the safety and teratogenic risks of microbicide and oral PrEP exposure in pregnancy. In the event that a study site is not taking part in MTN-016, participants may be contacted to collect the outcome of pregnancies up to one year after the birth of the infant.

9.9 Criteria for Early Termination of Study Participation

Participants may voluntarily withdraw from the study for any reason at any time. The IoR/designee also may withdraw participants from the study to protect their safety and/or if they are unwilling or unable to comply with required study procedures, after consultation with the PSRT. Participants may also be withdrawn if IPM, NIAID, MTN, government or regulatory authorities, including the FDA and Office for Human Research Protections (OHRP), or site IRBs terminate the study prior to its planned end date. Every reasonable effort is made to complete a final evaluation of participants who withdraw or are withdrawn from the study prior to completing follow-up. Study staff members will record the reason(s) for all withdrawals in participants' study records.

10 STATISTICAL CONSIDERATIONS

10.1 Overview and Summary of Design

This is a Phase 1, double-blind, three-arm, multi-site PK and safety study of three silicone elastomer vaginal matrix rings: a VR containing 200 mg DPV, a VR containing 200 mg DPV + 32 mg LNG, and a VR containing 200 mg DPV + 320 mg LNG. A total of approximately 36 healthy, HIV-uninfected females will be enrolled and randomized 1:1:1 (12 per arm) to use a study VR continuously for 14 days.

10.2 Study Endpoints

Consistent with the primary study objective to assess the PK of the study VRs worn continuously for 14 days, the following endpoints will be assessed:

- Dapivirine and levonorgestrel concentrations in blood
- Dapivirine and levonorgestrel concentrations in vaginal fluid

Consistent with the primary study objective to assess safety of the study VRs worn continuously for 14 days, the primary safety endpoints are the proportion of women with the following:

- Grade 2 or higher genitourinary adverse event as defined by the Division of AIDS (DAIDS) Table for Grading the Severity of Adult and Pediatric Adverse Events, Version 2.0, November 2014, and/or Addendum 1 (Female Genital [Dated November 2007] Grading Table for Use in Microbicide Studies)
- Grade 3 or higher adverse event as defined by the Division of AIDS (DAIDS) Table for Grading the Severity of Adult and Pediatric Adverse Events, Version 2.0, November 2014

Consistent with the secondary study objective to describe vaginal bleeding experienced during study participation, the following endpoint will be assessed:

- Self-reported vaginal bleeding

10.3 Primary Study Hypotheses

- Blood and CVF DPV and LNG levels will be measureable in all women randomized to DPV and LNG VRs
- Continuous exposure to DPV, or DPV and LNG, via VR for 14 days will be safe

10.4 Sample Size and Power Calculations

10.4.1 Primary Endpoints

The proposed total sample size is approximately N=36 women randomized into 3 arms in a 1:1:1 ratio giving 12 women per group. This sample size is based upon the size of similar Phase 1 studies of vaginal microbicide products.

As a means to characterize the statistical properties of this study Table 13 below presents the probability of observing ten or more, eleven or more, or twelve women with detectable PK levels among the 12 women in each arm given a true event rate. For example, if the true rate of detection among women using a ring is 99% then the probability we will see 11 or more women with detectable PK levels is 99%.

Table 13: Analysis of PK Event Frequency

"True" Event Rate (PK Detectable)	P (≥10 events | n=12)	P (≥11 events | n=12)	P (12 events | n=12)
75%	0.39	0.16	0.03
90%	0.89	0.66	0.28
99%	1.00	0.99	0.89

Table 14 below presents the probability of observing zero, one or more and two or more safety events among the 12 women in each arm given a true event rate. For example, if the true rate of a safety event among women using a ring is 15% then the probability we will see 1 or more women with this event is 86%.

Table 14: Analysis of Safety Event Frequency

"True" Event Rate (Safety Event)	P (0 events | n=12)	P (≥1 events | n=12)	P (≥2 events | n=12)
1%	0.89	0.11	<0.01
5%	0.54	0.46	0.12
15%	0.14	0.86	0.56

An alternative way of describing the statistical properties of the study design is in terms of the 95% confidence interval (95% CI) for the true rate based on the observed data. Table 15 below shows the exact 2-sided 95% CIs for the probability of an event based on a particular observed rate. If none of the 12 participants in an arm experience a safety event, the 95% exact 2-sided upper confidence bound for the true rate of such events in a particular arm of the study is 26%. Similarly if all of the 12 participants in an arm have detectable PK, the 95% exact 2-sided lower confidence bound for the true rate of PK detection is 74%.

Table 15: Precision of Exact 2-sided 95% CIs for Observed Event Rates

Observed Event Rate	Exact 2-sided 95% CI (n=12)
12/12 (100%)	74%, 100%

10/12 (83%)	52%, 98%
8/12 (67%)	35%, 90%
6/12 (50%)	21%, 79%
4/12 (33%)	10%, 65%
2/12 (17%)	2%, 48%
0/12 (0%)	0%, 26%

10.4.2 Secondary Endpoint

The statistical properties of the study design for the secondary outcome of vaginal bleeding are similar to those presented above for the primary endpoints. Specifically Table 14 presents the probability of observing zero, one or more and two or more vaginal bleeding events among the 12 women in each arm given a true event rate and Table 15 shows the exact 2-sided 95% CIs for the probability of vaginal bleeding based on a particular observed rate.

10.5 Participant Accrual, Follow-up, Retention, and Replacement

Based on previous studies of vaginal products with similar eligibility requirements, the accrual of 36 eligible participants will take approximately 8-10 months. Each participant will be followed for 16 consecutive days. Each site will target retention of 95% of enrolled participants over the study period. Women lost to follow-up and/or without study product for more than 3 days will be replaced in order to obtain complete data on PK. Details on participant replacement are outlined in the MTN-030/IPM 041 SSP Manual.

10.6 Randomization

Participants will be randomized in a 1:1:1 ratio to the three arms of the study. Study arm randomization will be stratified by site to ensure balanced assignment of products at each site. The randomization scheme, including enrollment of replacement participants, will be generated and maintained by the MTN SDMC.

10.7 Blinding

Study staff and participants will be blinded to the treatment assignments of all study participants. All VRs will be individually packaged and labeled. Multiple codes will be utilized to conceal and protect randomization assignments and the identity of the content of the ring.

Blinding will be maintained until all data are entered into the study database, all study endpoint data and other data included in the final analysis have been cleaned and verified, and the data are ready for final analysis. This will be explained to participants as part of the study.

There are no circumstances under which it is expected that unblinding will be necessary for the provision of medical treatment or to otherwise protect the safety of study participants. As described in Section 9, in the event that an IoR/designee is concerned that a participant might be put at undue risk by continuing product use, the Investigator may discontinue study product use by this participant; however, knowledge of the specific product to which the participant was assigned should not be necessary to guide further follow-up and/or treatment. If an IoR/designee feels that specific product knowledge is necessary to protect participant safety, the IoR/designee will notify the PSRT to consider and rule upon the request.

10.8 Data and Safety Monitoring and Analysis

10.8.1 Study Monitoring Committee

Data and Safety Monitoring Board oversight is not planned for this study. The MTN SMC will conduct interim reviews of study progress, including rates of participant accrual, retention, completion of primary and main secondary endpoint assessments, study or lab issues, and a closed safety data report to voting SMC members. These reviews will take place approximately every 4-6 months, or as needed. At the time of this review, or at any other time, the SMC may recommend that the study proceed as designed, proceed with design modifications, or be discontinued. For further information regarding the SMC, please reference the MTN Manual of Operational Procedures (www.mtnstopshiv.org).

10.8.2 Primary Analysis

When the use of descriptive statistics to assess group characteristics or differences is required, the following methods will be used: for categorical variables, the number and percent in each category; for continuous variables, the mean, median, standard deviation, quartiles and range (minimum, maximum). Within-treatment group assessment of the change from the baseline measurement to a follow-up measurement will be analyzed using McNemar's test (for categorical response variables) or the paired t-test or Wilcoxon signed-ranks test (for continuous variables).

To assess the adequacy of the randomization, participants in each of the three arms will be compared for baseline characteristics including demographics and laboratory measurements using descriptive statistics. Due to the small sample size, formal comparisons will not be done.

PK Endpoints
The proportion of women with detectable drug levels in each arm and the measured drug concentration levels will be summarized using descriptive statistics and graphics.

Safety Endpoints

All visits in which participants have been exposed to the study products will be included in the primary analysis of safety. Secondary intent to treat analyses may also be performed. To assess genitourinary safety, the number and percentages of participants experiencing each safety endpoint (see section 10.2) will be tabulated by study arm as well as the total number of safety endpoints experienced in each arm. Each participant will contribute once in each category (i.e., only for the highest severity AE for each participant) for the calculation of event rates. Exact binomial confidence intervals will be calculated for each safety endpoint for each arm.

10.8.3 Secondary Analyses

All visits in which participants have been exposed to the study products will be included in the secondary analysis of vaginal bleeding. Secondary intent to treat analyses may also be performed. To assess reported vaginal bleeding, the number and percentages of participants experiencing this endpoint will be tabulated by study arm as well as the total number of cases of vaginal bleeding, the total number of person years of follow-up, and the incidence rate of vaginal bleeding by study arm.

10.8.4 Missing Data

In any situation with missing data, appropriate secondary analyses will be performed to adjust for variables that may be related to the missingness mechanism. If missing data rates are higher than 10%, covariates that are related to missingness in likelihood-based regression models will be included. A sensitivity analyses to assess the potential impact of the missing data will also be performed. These analyses will include imputing the data under the most extreme scenarios of information missingness, such as assuming everyone missing has an extreme value of the missing variable, and less informative imputation approaches.

11 DATA HANDLING AND RECORDKEEPING

11.1 Data Management Responsibilities

Data collection tools will be developed by the MTN SDMC and Population Council (SMS) in conjunction with the protocol team. Quality control reports and queries routinely will be generated and distributed by the SDMC to the study sites for verification and resolution. As part of the study activation process, each study site must identify all CRFs to be used as source documents. Data are transferred to the MTN SDMC, entered, and cleaned using a data management system.

11.2 Source Documents and Access to Source Data/Documents

All study sites will maintain source data/documents in accordance with current DAIDS policies.
(http://www.niaid.nih.gov/labsandresources/resources/daidsclinrsrch/Pages/Default.aspx)

Each IoR/designee will maintain, and store securely, complete, accurate and current study records throughout the study. In accordance with U.S. regulations regarding testing investigational products, the IoR/designee will maintain all study documentation for at least two years following the date of marketing approval for the study product being tested for the indication in which they were studied. If no marketing application is filed, or if the application is not approved, the records will be retained for two years after the investigation is discontinued and the US FDA is notified.

Study records must be maintained on site for the entire period of study implementation. Thereafter, instructions for record storage will be provided by DAIDS. No study records may be moved to an off-site location or destroyed prior to receiving approval from DAIDS.

11.3 Quality Control and Quality Assurance

All study sites will conduct quality control and quality assurance procedures in accordance with current DAIDS policies.
(http://www.niaid.nih.gov/labsandresources/resources/daidsclinrsrch/documents/qmppolicy.pdf)

12 CLINICAL SITE MONITORING

Study monitoring will be carried out by Pharmaceutical Product Development, Inc. (PPD) (Wilmington, NC) in accordance with current DAIDS policies. On-site study monitoring will be performed in accordance with current DAIDS policies. Study monitors will visit the site to do the following:

- Review informed consent forms, procedures, and documentation
- Assess compliance with the study protocol, Good Clinical Practices (GCP) guidelines, and applicable regulatory requirements (US and non-US), including US CFR Title 45 Part 46 and Title 21 Parts 50, 56, and 312
- Perform source document verification to ensure the accuracy and completeness of study data
- Verify proper collection and storage of biological specimens

- Verify proper storage, dispensing, and accountability of investigational study products
- Assess implementation and documentation of internal site quality management procedures

The IoR/designee will allow study monitors to inspect study facilities and documentation (e.g., informed consent forms, clinic and laboratory records, other source documents, CRFs), as well as observe the performance of study procedures. The IoR/designee also will allow inspection of all study-related documentation by authorized representatives of the MTN Coordinating Leadership and Operations Center (LOC), SDMC, LC, IPM, NIAID, FDA, OHRP, IRBs and local and US regulatory authorities. A site visit log will be maintained at the study site to document all visits.

13 HUMAN SUBJECTS PROTECTIONS

Site investigators will make efforts to minimize risks to participants. Participants and study staff members will take part in a thorough informed consent process. Before beginning the study, the IoR/designee will have obtained IRB approval and the protocol will have been submitted to the FDA. The IoR/designee will permit audits by the NIH, IPM, the FDA, OHRP, MTN LOC, IRBs, SDMC, and other local and US regulatory authorities or any of their appointed agents.

13.1 Institutional Review Boards/Ethics Committees

Each participating institution is responsible for assuring that this protocol, the associated site-specific informed consent forms, and study-related documents (such as participant education and recruitment materials) are reviewed by an IRB responsible for oversight of research conducted at the study sites. Any amendments to the protocol must be approved by the responsible IRBs prior to implementation.

Subsequent to the initial review and approval, the responsible IRBs must review the study at least annually. Each IoR/designee will make safety and progress reports to the IRBs at least annually and within three months after study termination or completion. These reports will include the total number of participants enrolled in the study, the number of participants who completed the study, all changes in the research activity, and all unanticipated problems involving risks to human subjects or others. In addition, the results of all SMC reviews of the study will be provided to the IRBs. Study sites will submit documentation of continuing review to the DAIDS Protocol Registration Office in accordance with the DAIDS Protocol Registration Policy and Procedures Manual.

13.2 Protocol Registration

Prior to implementation of this protocol, and any subsequent full version amendments, each site must have the protocol and the protocol informed consent form(s) approved, as appropriate, by their local institutional review board (IRB)/ethics committee (EC) and any other applicable regulatory entity (RE). Upon receiving final approval, sites will submit all required protocol registration documents to the DAIDS Protocol Registration Office (DAIDS PRO) at the Regulatory Support Center (RSC). The DAIDS PRO will review the submitted protocol registration packet to ensure that all of the required documents have been received.

Site-specific informed consent forms (ICFs) will be reviewed and approved by the DAIDS PRO and sites will receive an Initial Registration Notification from the DAIDS PRO that indicates successful completion of the protocol registration process. A copy of the Initial Registration Notification should be retained in the site's regulatory files.

Upon receiving final IRB/EC and any other applicable RE approval(s) for an amendment, sites should implement the amendment immediately. Sites are required to submit an amendment registration packet to the DAIDS PRO at the RSC. The DAIDS PRO will review the submitted protocol registration packet to ensure that all the required documents have been received. Site-specific ICF(s) will not be reviewed and approved by the DAIDS PRO and sites will receive an Amendment Registration Notification when the DAIDS PRO receives a complete registration packet. A copy of the Amendment Registration Notification should be retained in the site's regulatory files.

For additional information on the protocol registration process and specific documents required for initial and amendment registrations, refer to the current version of the DAIDS Protocol Registration Manual.

13.3 Study Coordination

IPM holds the Investigational New Drug (IND) application for this study. Copies of all regulatory documents submitted to this IND by IPM are forwarded to DAIDS. Assignment of all sponsor responsibilities for this study will be specified in a Clinical Trial Agreement (CTA) executed by NIAID and IPM.

Study implementation will also be guided by a common study-specific procedures manual (SSP) that provides further instructions and operational guidance on conducting study visits; data and forms processing; specimen collection, processing, and shipping; AE assessment, management and reporting; dispensing study products and documenting product accountability; and other study operations. Standardized study-specific training will be provided to all sites by the MTN LOC, SDMC, LC and other designated members of the Protocol Team.

Close coordination between protocol team members is necessary to track study progress, respond to queries about proper study implementation, and address other issues in a timely manner. The PSRT will address issues related to study eligibility and AE management and reporting as needed to assure consistent case management, documentation, and information-sharing across sites. Rates of accrual, adherence, follow-up, and AE incidence will be monitored closely by the team as well as the SMC.

13.4 Risk Benefit Statement

13.4.1 Risks

General
It is not expected that this trial will expose human subjects to unreasonable risk.

Pelvic examination and procedures may cause mild discomfort, pressure and/or vaginal bleeding or spotting. Phlebotomy may lead to discomfort, feelings of dizziness or faintness, bruising, swelling and/or infection. Disclosure of HIV and STI status may cause worry, sadness or depression. Disclosure of HIV-positive status has been associated with depression, suicidal ideation, and denial as well as social isolation. Trained counselors will be available to help participants deal with these feelings. Participation in clinical research includes the risks of loss of confidentiality and discomfort with the personal nature of questions when discussing sexual behaviors.

Participants at sites where local regulatory authorities require partner notification in response to diagnosed STI or HIV infection could have problems in their relationships with their sexual partners. Participants also could have problems in their partner relationships associated with use of study product and abstinence requirements.

Use of the study VR may lead to vaginal symptoms, including irritation, increased discharge, and discomfort (including with vaginal intercourse if it were to occur). It is possible that a participant may have an allergic reaction to the study product. Symptoms of an allergic reaction include rash or other skin irritation, itching, joint pain, or difficulty in breathing.

Based on AEs reported among female participants in previous studies, DPV VRs may be associated with:

- Intermenstrual bleeding
- Vaginal discharge
- Vaginal candidiasis
- Bacterial vaginosis
- Urinary tract infection

The most common adverse reactions reported in clinical trials of Plan B® were (>10%):

- Heavier menstrual bleeding
- Nausea

- Lower abdominal pain
- Fatigue
- Headache
- Dizziness

The most common adverse reactions reported in clinical trials of an LNG-releasing subcutaneous implant system (Jadelle®) include (>10%):

- Headache
- Nervousness
- Dizziness
- Nausea
- Changes in menstrual bleeding
- Cervicitis
- Vaginal discharge
- Genital pruritus
- Pelvic pain
- Breast pain
- Weight increase
- Vaginal fungal infection
- Acne

The most common adverse reactions reported in clinical trials of an LNG-releasing subcutaneous implant system (Norplant®) include (>10%):

- Many bleeding days or prolonged bleeding
- Spotting
- Amenorrhea
- Irregular (onsets of) bleeding
- Frequent bleeding onsets
- Scanty bleeding

The most common adverse reactions (in >5% users) for Mirena®, Jadelle® and Plan B® are similar and include uterine/vaginal bleeding alterations (including amenorrhea, menorrhagia and intermenstrual bleeding), abdominal/pelvic pain, headache/migraine, acne, depressed/altered mood, breast tenderness/pain, vaginal discharge and nausea. Other rare, and potentially more serious, AEs associated with continued LNG use that have been reported are ectopic pregnancy, ovarian cysts, thrombosis, and idiopathic intracranial hypertension (particularly in obese participants).

Although study sites make every effort to protect participant privacy and confidentiality, it is possible that participants' involvement in the study could become known to others, and that social harms may result (i.e., because participants could become known as HIV-positive or at "high risk" for HIV infection). For example, participants could be treated unfairly or discriminated against, or could have problems being accepted by their families, communities, and/or employer(s).

13.4.2 Benefits

Participants will receive HIV/STI risk reduction counseling, HIV and STI testing, physical examination, pelvic examination, and routine laboratory testing. Participants will be provided with STI treatment in accordance with CDC guidelines. For other medical conditions identified as part of the study screening and/or follow-up procedures, participants will be referred to other sources of care available in their community. Some participants may have the opportunity to access expedient treatment and decreased morbidity due to early diagnosis and treatment of abnormalities identified during tests, examinations and referrals.

Participants and others may benefit in the future from information learned from this study. Specifically, information learned in this study may lead to the development of safe and effective interventions to prevent HIV transmission and unplanned pregnancy. Information learned in this study may also help to understand issues important for broader implementation of the DPV ring. Participants may also appreciate the opportunity to contribute to the field of HIV prevention research.

13.5 Informed Consent Process

Written informed consent will be obtained from each study participant prior to screening. Written informed consent also will be obtained for long-term specimen storage and possible future testing. Consent for long-term specimen storage is not required for study participation. In obtaining and documenting informed consent, the IoR and their designees will comply with applicable local and US regulatory requirements and will adhere to GCP and to the ethical principles that have their origin in the Declaration of Helsinki. Study staff must document the informed consent process in accordance with the Requirements for Source Documentation in DAIDS Funded and/or Sponsored Clinical Trials (http://rsc.tech-res.com/policiesandregulations/). Participants will be provided with copies of the informed consent forms if they are willing to receive them.

In addition to informed consent forms, the Protocol Team will work with study staff and community representatives to develop appropriate materials about the study and a standardized approach to the informed consent process to be implemented at all study sites, which will be detailed in the study-specific procedures manual.

The informed consent process will cover all elements of informed consent required by research regulations. In addition, the process specifically will address the following topics of importance to this study:

- The unknown safety and unproven efficacy of the study product
- Randomization and the importance of participants in all of the study groups to the success of the study
- The importance of adherence to the study visit and procedures schedule
- The potential medical risks of study participation (and what to do if such risks are experienced)
- The potential social harms associated with study participation (and what to do if such harms are experienced)
- The real yet limited benefits of study participation
- The distinction between research and clinical care
- The right to withdraw from the study at any time

13.6 Participant Confidentiality

All study procedures will be conducted in private, and every effort will be made to protect participant privacy and confidentiality to the extent possible. Each study site will implement confidentiality protections that reflect the local study implementation plan and the input of study staff and community representatives to identify potential confidentiality issues and strategies to address them. In addition to local considerations, the protections described below will be implemented at all sites.

All study-related information will be stored securely. All participant information will be stored in locked areas with access limited to study staff. All laboratory specimens, study data collection, and administrative forms will be identified by coded number only to maintain participant confidentiality. All local databases will be secured with password protected access systems. Forms, lists, logbooks, appointment books, and any other listings that link participants' ID numbers to identifying information will be stored in a locked file in an area with limited access. After receiving appropriate approval, all study documents/data will be properly disposed of, including the proper destruction and/or deletion of paper files, electronic study data, and electronic documents. Participants' study information will not be released without their written permission, except as necessary for review, monitoring, and/or auditing by the following:

- Representatives of the US Federal Government, including the US FDA, OHRP, NIH, and/or contractors of the NIH
- Representatives of IPM
- Representatives of the MTN LOC, SDMC, and/or LC
- Study staff
- Site IRBs

MTN has obtained a Certificate of Confidentiality from the US Department of Health and Human Services that is applicable for this study. This Certificate protects study staff from being compelled to disclose study-related information by any US Federal, State or local civil, criminal, administrative, legislative or other proceedings. It thus serves to protect the identity and privacy of study participants.

13.7 Special Populations

13.7.1 Pregnant Females

Females who test positive for pregnancy at the Screening or Enrollment Visit will not be eligible to participate in this study. Should a woman test positive for pregnancy after Enrollment, a product discontinuation will be implemented. Follow-up will be completed and data collected per Section 7.5.2. During the informed consent process, women will be informed that the study VR is not proven to be an effective method of contraception and the effects of the study VR on a developing human fetus are unknown.

13.7.2 Children

The NIH has mandated that children be included in research trials when appropriate. This study meets "Justifications for Exclusion" criteria for younger children as set forth by the NIH. Specifically, "insufficient data are available in adults to judge potential risk in children" and "children should not be the initial group to be involved in research studies." This study does not plan to enroll children under 18 years old.

13.8 Compensation

Pending IRB approval, participants will be compensated for their time and for their effort contributed to this study, and/or be reimbursed for travel to study visits. Site specific compensation amounts will be specified in the study informed consent forms of each individual site.

13.9 Communicable Disease Reporting

Study staff will comply with local requirements to report communicable diseases including HIV-1 identified among study participants to health authorities. Participants will be made aware of reporting requirements during the informed consent process.

13.10 Access to HIV-related Care

HIV test-related counseling will be provided to all potential study participants who consent to undergo HIV-1 screening to determine their eligibility for this study, and to all enrolled participants at each follow-up HIV-1 testing time point. Testing will be performed in accordance with the algorithm in Appendix II. Counseling will be provided in accordance with standard HIV counseling policies and methods at each site and additionally will emphasize the unknown efficacy of the study products in preventing HIV-1 infection. In accordance with the policies of the NIH, participants must receive their HIV-1 test results to take part in this study.

13.11 Care for Participants Identified as HIV-Positive

An individual who has been identified as infected with HIV-1 will be managed or referred for management according to the local standard of care. Should a woman test positive for HIV after the Enrollment Visit, follow-up procedures will be performed as per Section 7.5.1. Please refer to Section 9.7 for further details.

13.12 Study Discontinuation

This study may be discontinued at any time by NIAID, the MTN, IPM, the US FDA, OHRP, other government or regulatory authorities, or site IRBs.

14 PUBLICATION POLICY

DAIDS/NIAID and MTN policies and a CTA between NIAID and IPM will govern publication of the results of this study.

15 APPENDICES

APPENDIX I: SCHEDULE OF STUDY VISITS AND EVALUATIONS

		Visit 1 SCR	Visit 2 ENR (Day 0)	Visit 3-5 (Days 1, 2, 3)	Visit 6 (Day 7)	Visit 7 (Day 14)	Visit 8 & 9 (Days 15 and 16)
**ADMINISTRATIVE AND REGULATORY**							
	Obtain Informed consent(s)	X					
	Assign a unique Participant Identification (PTID) number	X					
	Assess and/or confirm eligibility	X	X				
	Demographic information	X					
	Collect/review/update locator information	X	X	X	X	X	X
	Randomization		X				
	Provide Reimbursement	X	X	X	X	X	X
	Schedule next visit/contact	*	*	X	X	X	* Day 15 mandatory
**BEHAVIORAL**							
	HIV pre- and post-test counseling	X	X	*	*	X	*
	HIV/STI risk reduction counseling	X	X	*	*	X	*
	Protocol adherence counseling		X	*	X	X	*
	Collect product use information			X	X	X	
	Behavioral assessment		X			X	
**CLINICAL**							
	Medical eligibility information (including exclusionary medical conditions and medications)	X	X				
	Medical/menstrual history	X	X	X	X	X	X
	Concomitant medications	X	X	X	X	X	X
	Full/Modified physical examination	X	X	▲*	▲*	▲	
	Pelvic examination	X	X	* Day 3 mandatory	X	X	*
	Digital examination by clinician to check VR placement		X				
	Treatment for RTI, UTI, or STIs	*	*	*	*	*	*
	Disclosure of available test results	X	X	X	X	X	X
	Collect AEs			X	X	X	X
**LABORATORY**							
URINE	hCG	X	X	*	*	*	* Day 16 mandatory
URINE	Urine dipstick/culture	*	*				
BLOOD	HIV-1 testing	X	X	*	*	X	*
BLOOD	Plasma archive		X				
BLOOD	Serum creatinine	X	X	*	*	X	*
BLOOD	CBC with platelets and differential	X	X	*	*	X	*
BLOOD	AST/ALT	X	X			X	*
BLOOD	Syphilis serology	X		*	*	*	*
BLOOD	DPV levels		X∞	X∞	X∞	X∞	X∞
BLOOD	LNG levels		X∞	X∞	X∞	X∞	X∞
BLOOD	Sex hormone-binding globulin (SHBG) and albumin		X			X	
BLOOD	Serum progesterone and estradiol		X				

		Visit 1 SCR	Visit 2 ENR (Day 0)	Visit 3-5 (Days 1, 2, 3)	Visit 6 (Day 7)	Visit 7 (Day 14)	Visit 8 & 9 (Days 15 and 16)
PELVIC	NAAT for GC/CT	X	*	*	*	*	*
	Trichomonas test	X	*	*	*	*	*
	Herpes lesion testing	*	*	*	*	*	*
	Pap test	^					
	Saline/ KOH wet mount with pH for candidiasis and/or BV	*	*	*	*	*	*
	Vaginal Gram stain		X	* Day 3 mandatory	*	X	*
	CVF DPV levels		X∞	X∞	X∞	X∞	X∞
	CVF LNG levels		X∞	X∞	X∞	X∞	X∞
**STUDY PRODUCT / SUPPLIES**							
Provision of one study VR and VR use instructions			X				
Insertion of the provided VR			X				
Removal and collection of study VR						X	
Provide condoms		*				*	

* = if indicated; ^ = if indicated (if participant [over age 21] is unable to provide documentation of a satisfactory Pap test within 3 years prior to Enrollment); ∞ = See Table 12 for additional details on sample collection; ▲= Modified

APPENDIX II: ALGORITHM FOR HIV TESTING FOR SCREENING AND ENROLLED PARTICIPANTS

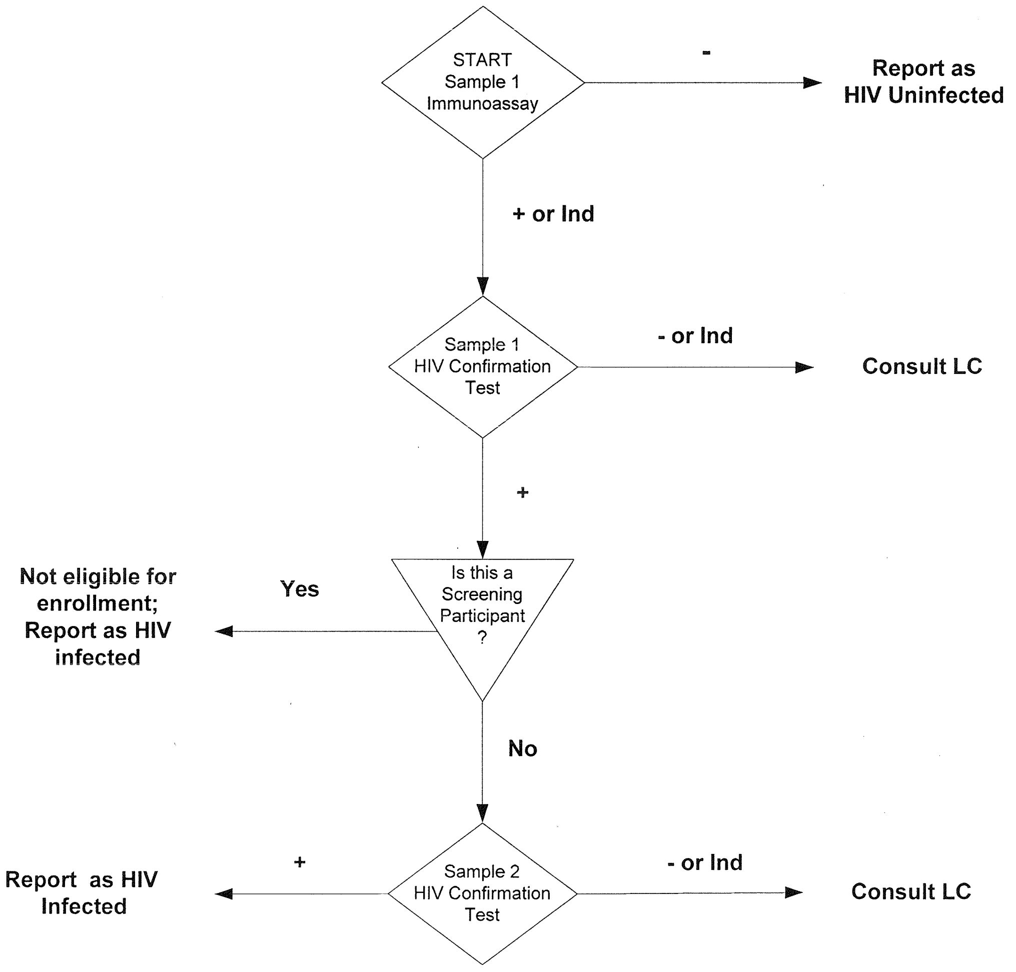

Ind: Indeterminate test results
LC: Laboratory Center

APPENDIX III: SAMPLE INFORMED CONSENT FORM (SCREENING, ENROLLMENT, LONG-TERM STORAGE AND FUTURE TESTING)

SAMPLE INFORMED CONSENT FORM
DIVISION OF AIDS, NIAID, NIH

MTN-030/IPM 041

Version 1.0

April 6, 2016

A Phase 1, Randomized, Double-Blind Pharmacokinetic and Safety Study of Dapivirine/Levonorgestrel Vaginal Rings

PRINCIPAL INVESTIGATOR: [Sites to insert]
PHONE: [Sites to insert]
SHORT TITLE: PK and Safety Study of Vaginal Rings Containing Dapivirine and Levonorgestrel

INFORMED CONSENT

You are being asked to take part in this research study because you are:

- female
- HIV-negative
- healthy
- have regular menstrual cycles
- between the ages of 18 and 45 years old

Approximately 36 women who agree to be sexually abstinent for the duration of their study participation will participate at two study sites in the United States. This study is sponsored by the US National Institutes of Health (NIH) and conducted by the Microbicide Trials Network (MTN). The study products in this clinical trial include one vaginal ring (VR) containing the anti-HIV medication, dapivirine (DPV), and two VRs containing a combination of DPV and different doses of an FDA-approved contraceptive hormone called levonorgestrel (LNG). If you agree to take part in this study, one VR will be placed in your vagina to be worn continuously for 14 days (about 2 weeks). The VR is made out of flexible plastic. You will be asked to leave the ring in place for approximately 14 continuous days and attend clinic visits while the ring is in place and for two days after the ring is removed. The study rings are supplied by the International Partnership for Microbicides, a not-for-profit research organization. At this site, the person in charge of this study is ***[INSERT NAME OF PRINCIPAL INVESTIGATOR]***.

Before you decide if you want to join this study, we want you to learn more about it. This consent form gives you information about the study. Study staff will talk with you and

answer any questions you may have. Once you have read this form and understand the study and its requirements, you can decide if you want to join. If you do decide to take part in the trial, you will sign your name on this form. A copy of this form will be offered to you. Signing this consent form does not mean you will be able to join the study. You must first complete the screening tests and exams to see if you are eligible.

It is important to know that your participation in this research is your decision and taking part in this study is completely voluntary (see Your Rights as a Research Participant/Volunteer for more information).

WHAT IS THE PURPOSE OF THIS STUDY?

Two drugs are being used in this study. The drugs are dapivirine (DPV) and levonorgestrel (LNG). The main purpose of this research study is to find out how these drugs enter and exit the body when a ring containing a combination of these drugs is inserted into the vagina and left in place for approximately 14 days (about 2 weeks). Another purpose of this study is to find out if the VR is safe and well-tolerated. This study will provide important information about the DPV and DPV-LNG VRs when used by women of childbearing age. This study will also provide important information on the best dose of these medications when delivered in a VR, and help develop VRs in the future, including combination VRs that can also be used to prevent pregnancy.

STUDY PRODUCTS

Dapivirine

DPV VRs have been previously tested and found to be generally safe and well-tolerated. DPV was recently tested in two large studies to see whether it can help to prevent the spread of HIV. It was shown to be safe and helps to prevent HIV acquisition. Study staff can provide you with additional information about these studies if you are interested in learning more. HIV is the virus that causes AIDS. DPV works by preventing HIV from making copies of itself, which stops the spread of HIV in the body.

Levonorgestrel

Many contraceptives such as pills and VRs contain progestin hormones like LNG. The hormone is intended to keep women from releasing an egg so they do not get pregnant. It also thickens the mucus of the cervix (the tissue that attaches the vagina to the uterus) to prevent sperm from reaching an egg. Currently there is one approved VR available in the United States (called NuvaRing®), which contains a different progestin hormone, etonogestrel, as well as an estrogen hormone, ethinyl estradiol. The new VRs being tested in this study will be used for 14 days and the only hormone they contain is a progestin hormone, LNG.

While DPV has been tested before in humans, this is the first time a VR containing both DPV and LNG has been studied in humans. Only a small amount of clinical data exists on VRs containing 200 mg DPV without LNG. VRs containing 200 mg of DPV are not

expected to deliver more DPV throughout the body than has been shown to be safe and well-tolerated in earlier studies in which DPV was taken orally. Therefore, the dose of DPV for use in this study is unlikely to result in significantly greater side effects than VRs with a smaller amount of DPV. Close monitoring will be performed over the relatively short period of planned product use during this study to respond quickly to any safety concerns.

Researchers are continuing to study DPV to learn more about how it works in humans to protect against HIV infection. There are only two known effective ways to prevent sexually transmitted HIV in women: condom use during sex and/or the use of pre-exposure prophylaxis (PrEP). PrEP is a new HIV prevention method where people who do not have HIV take an oral tablet to reduce their risk of becoming infected. Study staff can provide you with additional information about PrEP if you are interested in learning more.

Researchers also do not yet know if the LNG prevents pregnancy when combined with DPV in a VR. Therefore, it will be important that you use another form of contraception that is non-hormonal. You will also need to remain sexually abstinent for the duration of your study participation.

STUDY GROUPS

Approximately 36 eligible participants will be randomized equally to one of three VR study groups:

- A ring containing DPV
- A combination ring containing DPV and a low dose strength of LNG
- A combination ring containing DPV and a high dose strength of LNG

Approximately 12 participants will be assigned to each of the three study groups,. This means that approximately two-thirds of participants will receive a VR containing both DPV and LNG. One third will receive a ring with DPV only. Participants will be assigned to a group by random chance (like throwing dice). Neither you nor the study staff will know which group you are in until the study is completed. Each participant will insert one ring into her vagina and be asked not to remove it for approximately 14 days.

All three study groups are important. No matter which study group you are in, you must remember that we do not know if the drugs contained within these rings will work to protect you against pregnancy or from getting HIV.

WHAT WILL HAPPEN DURING THE STUDY VISITS?

Screening Procedures:

The study includes a total of nine (9) clinic visits. All visits will take place at this clinic.

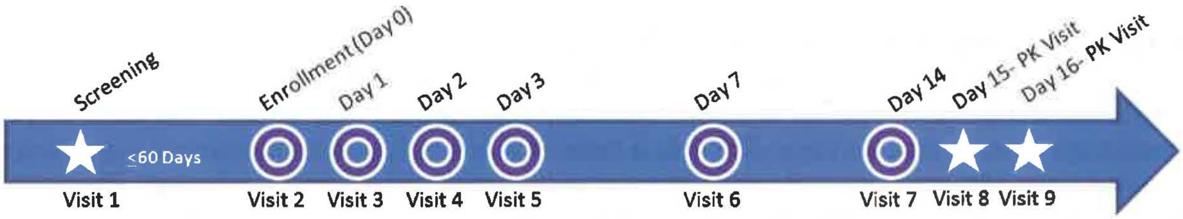

Screening Visit

The procedures done today will take about ***[SITES TO INSERT TIME]***.

- You will answer questions to confirm that you are able to join the study.
- Study staff will ask you about where you live and other questions about you, your medical health (including what medications you are taking), menstrual history, and your understanding of the study requirements. They may also ask to view your medical records, with your permission.
- You will be asked to provide study staff with your contact information (i.e., where you live and how we can contact you).
- Study staff will:
  - Test your urine for pregnancy
    - If you are pregnant you cannot join this study.
    - Study staff will talk with you about ways to avoid becoming pregnant.
    - You will answer questions about whether you are using an effective, non-hormonal method of contraception and intend to use this method for the entire time that you are in this study.
    - If needed, study staff will provide and/or refer you to obtain an acceptable contraceptive method for use during your participation in the study. Acceptable methods include:
      - Sterilization: You or your partner has been sterilized (tubal ligation, vasectomy, etc.)
      - Non-hormonal (for example, copper) intrauterine devices (IUDs) inserted at least 28 days (4 weeks) prior to enrollment
      - You engage in sex exclusively with women
      - Sexually abstinent for the past 90 days
  - Perform a physical examination
  - Perform a pelvic examination:
    - The study clinician will use a speculum, a plastic or metal instrument inserted in the vagina. Study staff will ask if you are experiencing

symptoms of an infection. They will check your vagina and cervix for signs of infection and other problems.
  - A small amount of vaginal fluid will be collected via swab(s), like a Q-tip. These swabs will be used to test for sexually transmitted infections and diseases (commonly known as STIs or STDs) and other problems.
  - If you are older than 21, the study staff may also collect samples from your cervix for a "Pap test" or "Pap smear". Study staff will inform you of the results of your Pap test. It takes about ***[SITES TO INSERT AMOUNT OF TIME]*** before Pap test results are ready. If you are 21 years of age or older and have a written report confirming a normal Pap test in the past 3 years, or if you had an abnormal Pap test but had follow-up indicating no treatment was required, you will not need to have a Pap test taken at this screening visit. The results of your Pap test may affect whether or not you can join the study.
- Take a blood sample ***[SITES TO INSERT AMOUNT]***:
  - To test the health of your blood, liver and kidneys.
  - To test for infections that typically are passed through sex, including HIV
    - You will be told your test results as soon as they are available. You will talk with the study staff about the meaning of your results, how you feel about them, and learn about ways to prevent HIV and other STIs. Sometimes HIV tests are not clearly positive, but also not clearly negative. In that case, we will do more tests until we are sure of your status. To participate in the study you must receive the results of your HIV test. If the test shows you have HIV, you cannot join the study. We will refer you to available sources of medical care and other services you may need. The study staff will tell you about other studies you may be eligible for, if any.
- If needed, give you treatment or refer you for treatment of STIs or other urinary or reproductive tract infections.
- Inform you about other services, if needed.
- Provide you with the results of your tests, when available. It is expected that all of your results will be available by ***[SITES TO SPECIFY TIMEFRAME]***.
- Give you male condoms, if you need them.
- Reimburse you for your visit.
- Talk with you about the requirements of the study, including the importance of completing clinic visits, and study activities and procedures according to the study schedule.
- Schedule your next visit to enroll in the study, if you are willing and eligible.

If you decide not to join the study, blood and other samples collected at this visit will not be kept or used for any tests other than those listed above.

If you decide to enroll in the study, you will be asked to receive and reply to text messages about your study product use and/or menstrual bleeding or spotting daily <u>for the duration of your participation</u>. Your answers will be kept private.

For the duration of your study participation, you will also be asked to abstain from sexual practices, tampon use and other non-study products for specified periods of time prior to your clinic visits. See stated length of time highlighted below:

Activity:	*For How Long?*
• Penile-vaginal intercourse<br>• Receptive oral intercourse<br>• Finger stimulation<br>• Inserting any objects into your vagina, including:<br>o Sex toys<br>o Female condoms<br>o Diaphragms<br>o Menstrual cups<br>o Cervical caps or any other vaginal barrier method<br>• Vaginal products, including:<br>o Spermicides<br>o Lubricants<br>o Contraceptive VRs<br>o Douches<br>o Vaginal medications<br>o Vaginal moisturizers<br>o Tampons	• For the duration of study participation beginning 24 hours before the enrollment visit

Enrollment Visit:

The Enrollment Visit is when you join the study. This visit will take about ***[SITES TO INSERT TIME]***. In addition to the procedures listed below, it is possible that study staff may need to perform additional tests if medically necessary (for example, you report having symptoms of a urinary, genital, or other infection and/or other issues).

The following procedures are specific to the Enrollment Visit, which will take place up to 60 days (approximately 8 ½ weeks) after your Screening Visit:

- You will answer questions to confirm you are able to join the study
- You will update study staff with your contact information (i.e., where you live and how we can contact you)
- Study staff will:
  - Talk with you about the requirements of the study and how to follow them, including restrictions on sexual practices. **If you do not think you can abstain from sex and tampon use for the approximate 2-week study duration then you should not join this study.** Sex for this study is defined as penile-vaginal intercourse, receptive oral intercourse, and finger stimulation.
  - Ask you questions about vaginal practices that may affect how your body absorbs the study drugs.
  - Ask you questions about your thoughts on the study product.

    - A staff member may ask you these questions. It is important that you know that you will answer these questions in private and your responses will be kept confidential.
  - Talk with you about STIs, HIV, HIV/STI testing, and ways to avoid HIV and other infections passed through sex.
  - Discuss any health or medical problems you may have had in the past, are currently experiencing or that have occurred since your last visit (including what medications you are taking)
  - Ask you about any menstrual periods or spotting you may have had since your last visit.
- You will also:
  - Have a physical examination
  - Have your urine tested for pregnancy
  - Have blood samples taken [SITES TO INSERT AMOUNT] at several time points including before you receive the study VR and at one, two, four, and six hours after you receive the study VR. An intravenous cannula (IV tube) may be placed for up to 6 hours after you receive the study VR for the blood draws.
    - The blood samples will be collected:
      - For research purposes, to test for sex hormone (progesterone, estradiol) and a hormone-related protein (sex hormone binding globulin [SHBG]) and albumin testing
      - For HIV testing
      - For research purposes, to learn how DPV and LNG enter and exit the body over time.
    - We will also collect blood
      - In case there is a question about your lab results in the future
      - To test the health of your blood, liver and kidneys.
  - Have a pelvic examination
    - The study clinician will then use a speculum to check your vagina and cervix for signs of problems due to the ring or infection. They will also take samples to test for bacteria and organisms in the vagina, if necessary
    - Study staff will ask if you are experiencing symptoms of an infection
    - A small amount of vaginal fluid will be collected via swab and at one, two, four, and six hours after you receive the study VR. Swabs will also be collected to test for STIs and for research purposes, including to see how much DPV or DPV-LNG is present in your blood once you start using the VR. The same tests will be done when these samples are collected at future visits.
  - Receive and insert the study ring. Study staff may help you insert the VR if you cannot do it on your own. All participants will have an examination to ensure the ring is inserted correctly. You will be asked to keep the VR in place and not remove it between visits, until Visit 7 (approximately 14 days later). Study staff will show you how to take the ring out in case you need to

do so. Study staff will talk with you about what to do if you have any problems or symptoms while using the ring.

- Receive treatment or be referred for treatment issues that the study staff may find
- Receive test results, if available
- Talk with study staff about any of your questions. Study staff will talk with you if you encounter any problems or symptoms while undergoing any of the procedures listed above.
- Be reimbursed for your visit
- Schedule your next visit, if applicable

Visits 3-5:

- Visit 3 will take place approximately 1 day after your Enrollment Visit, and will take between ***[SITES TO SPECIFY TIMEFRAME]*** to complete.
- Visit 4 will take place approximately 2 days after your Enrollment Visit, and will take between ***[SITES TO SPECIFY TIMEFRAME]*** to complete.
- Visit 5 will take place approximately 3 days after your Enrollment Visit, and will take between ***[SITES TO SPECIFY TIMEFRAME]*** to complete.

In addition to the procedures listed below, it is possible that study staff may need to perform additional tests if medically necessary (for example, you report having symptoms of a urinary, genital, or other infection and/or other issues).

At each visit (except where noted otherwise), you will:

- Update study staff with your contact information
- Provide study staff information about your study product use
- Discuss any health or medical problems you may be currently experiencing or that have occurred since your last visit (including what medications you are taking)
- Be asked about any menstrual periods or spotting you may have had since your last visit.
- Talk with study staff about any problems that you may be experiencing as a result of wearing the VR or procedures performed during your last visit
    - As needed, study staff will speak with you again and answer your questions about the requirements of the study and wearing the VR, including keeping the VR in place and not removing it between visits

At each visit (except where noted otherwise), study staff will:

- Take a blood sample ***[SITES TO INSERT AMOUNT]*** for research purposes, to learn how DPV and LNG enter and exit the body over time
- Collect vaginal fluid samples via a swab
- Give you any available test results
- Reimburse you for your visit

- Schedule your next visit.

Visits 6 and 7:

- Visit 6 will take place after you have worn the VR for about 1 week (approximately 7 days after your Enrollment visit). It will take between *[SITES TO SPECIFY TIMEFRAME]* to complete.
- Visit 7 will take place after you have worn the VR for about 2 weeks (approximately one week after Visit 6). It will take between *[SITES TO SPECIFY TIMEFRAME]* to complete.

In addition to the procedures listed below, it is possible that study staff may need to perform additional tests if medically necessary (for example, you report having symptoms of a urinary, genital, or other infection and/or other issues).

At each visit (except where noted otherwise), you will:

- Update study staff with your contact information
- Provide study staff information about your study product use
- Discuss any health or medical problems you may be currently experiencing or that have occurred since your last visit (including what medications you are taking)
- Be asked about any menstrual periods or spotting you may have had since your last visit

At each visit (except where noted otherwise), study staff will:

- Talk with you about any problems you may be experiencing while wearing the VR or resulting from procedures performed during your last visit
- Speak with you about the requirements of the study
- Perform a modified physical exam (Visit 7 only)
- Ask you questions about your thoughts on the study product. (Visit 7)
- Talk with you about STIs, HIV, HIV/STI testing, and ways to avoid HIV and other infections passed through sex (Visit 7)
- Perform a pelvic examination.
  - Study staff will ask if you are experiencing symptoms of an infection
  - The study clinician will use a speculum. They will check your vagina and cervix for signs of problems due to the ring or infection. They will also take samples to test for bacteria and organisms in the vagina, if necessary.
  - Study staff will collect vaginal fluid samples via a swab. It is important to note that at Visit 7 fluid will be collected at two time points: before study VR removal and six hours after removal.
- Remove the VR if not previously collected (Visit 7)
- Take blood samples [SITES TO INSERT AMOUNT]
  - At Visit 6 and Visit 7 for research purposes, to learn how DPV and LNG enter and exit the body over time. It is important to note that at Visit 7 blood will be

collected at two time points: before study VR removal and six hours after removal (an intravenous cannula [IV tube] may be placed).
    - To test the health of your blood, liver and kidneys (Visit 7)
    - To test for sex hormone binding globulin and albumin (Visit 7)
    - To test for HIV (Visit 7)
- Give you any available test results
- Give you male condoms, if you need them (Visit 7)
- Reimburse you for your visit
- Schedule your next visit

Visits 8 and 9 (Days 15 and 16)

- Visit 8 will take place approximately one day after your vaginal ring has been removed. It will take between *[SITES TO SPECIFY TIMEFRAME]* to complete.
- Visit 9 will take place approximately two days after your vaginal ring has been removed. It will take between *[SITES TO SPECIFY TIMEFRAME]* to complete.

In addition to the procedures listed below, it is possible that study staff may need to perform additional tests if medically necessary (for example, you report having symptoms of a urinary, genital, or other infection and/or other issues).

At each visit (except where noted otherwise), you will:
- Update study staff with your contact information
- Discuss any health or medical problems you may be currently experiencing or that have occurred since your last visit (including what medications you are taking)
- Be asked about any menstrual periods or spotting you may have had since your last visit

Study staff will:
- Talk with you about any problems you may be experiencing
- Speak with you about the requirements of the study if you need a reminder.
- Collect vaginal fluid via a swab for research purposes, to learn how DPV and LNG enter and exit the body over time.
- Have your urine tested for pregnancy (Required at Visit 9)
- Take a blood sample ***[SITES TO INSERT AMOUNT]*** for research purposes, to learn how DPV and LNG enter and exit the body over time.
- Give you any available test results
- Reimburse you for your visit
- Schedule next visit/contact (Required at Visit 8)

It is important that you remember that at any time during the study, study staff can answer any questions you may have about the procedures mentioned above.

Additional Visits and Procedures

It may be necessary for you to have additional visit(s) and/or provide additional samples if any of the above procedures need to be repeated due to one or more of the following:

- Issues with sample processing, testing or shipping
- If you are experiencing any symptoms or changes in your physical condition
- If tests or procedures were missed or not conducted.

Additional testing may be performed as part of quality control.

RISKS AND/OR DISCOMFORTS

Risks of Blood Draws

Whenever your blood is drawn, you may have:

- Discomfort
- Feelings of dizziness or faintness
- Bruising, swelling and/or infection

Risks of Pelvic Exams

During pelvic exams and cervical and vaginal fluid collection you may feel discomfort or pressure in your vagina, genital area and/or pelvis. You may also have vaginal bleeding or spotting, which should stop shortly after the examination.

Risks of Study Ring

The study VR can cause some side effects, such as an allergic reaction. Signs of an allergic reaction include, but are not limited to: rash or other skin irritation, itching, joint pain, or difficulty in breathing.

We do not yet know all the side effects of the study VR. Some but not all women who used the ring in other studies have had:

- Vaginal discharge
- Vaginal irritation
- Vaginal discomfort

Risks of Dapivirine

Based on side effects reported among women in previous studies, DPV VRs may be associated with:

- Intermenstrual bleeding
- Vaginal discharge
- Vaginal candidiasis
- Bacterial vaginosis
- Urinary tract infection

Risks of Levonorgestrel

LNG has been approved for use in contraceptive products for more than three decades.

The most common adverse reactions reported in clinical trials of Plan B® were:

- Heavier menstrual bleeding
- Nausea
- Lower abdominal pain
- Fatigue
- Headache
- Dizziness

The most common adverse reactions reported in clinical trials of an LNG-releasing implant (Jadelle®) were:

- Headache
- Nervousness
- Dizziness
- Nausea
- Changes in menstrual bleeding
- Cervicitis (inflammation of the cervix)
- Vaginal infection
- Vaginal itching
- Vaginal discharge
- Pelvic pain
- Breast pain
- Weight gain
- Acne

The most common adverse reactions reported in clinical trials of a different LNG-releasing implant (Norplant®) were:

- Prolonged, frequent, or irregular bleeding
- Lack of vaginal bleeding (amenorrhea)
- Infrequent bleeding or spotting

The most common adverse reactions (in >5% users) for Mirena®, Jadelle® and Plan B® are similar and include uterine/vaginal bleeding alterations (including amenorrhea [abnormal absence of menstrual bleeding], menorrhagia [abnormally heavy menstrual bleeding] and intermenstrual bleeding [vaginal bleeding occurring between a woman's monthly menstrual periods]), abdominal/pelvic pain, headache/migraine, acne, depressed/altered mood, breast tenderness/pain, vaginal discharge and nausea. Other rare, and potentially more serious, adverse reactions associated with continued LNG use that have been reported are ectopic pregnancy (a pregnancy outside the uterus), ovarian cysts (fluid-filled sac within the ovary), thrombosis (blood clots), and idiopathic intracranial hypertension (increased pressure around the brain [particularly in obese participants]).

Other Possible Risks
You may become embarrassed and/or worried when discussing your sexual practices, ways to protect against HIV and other STIs, and your test results. You may be worried while waiting for your test results. If you have HIV or other infections, learning this could make you worried. Trained study counselors will help you deal with any feelings or questions you have.

We will make every effort to protect your privacy and confidentiality during the study visits. Your visits will take place in private. However, it is possible that your involvement in the study could become known to others, and that social harms may result (i.e., because participants could become known as HIV-positive or at "high risk" for HIV infection). For example, participants could be treated unfairly or discriminated against, or could have problems being accepted by their families, communities, and/or employer(s). Finding out your HIV or STI status could cause depression, suicidal thoughts and/or problems between you and your partner. If you have any problems, study counselors will talk with you and/or your partner to try to help resolve them.

Sexual Practices, Pregnancy, and Breastfeeding
LNG is widely used in different types of hormonal birth control; however, it is not known whether the study VRs containing LNG can prevent pregnancy. LNG is not recommended for use by women who are pregnant or may be pregnant. We do not know what effect DPV has on pregnancy, including the effect of DPV on the fetuses of women who use the VR when pregnant, or the babies of women who use the VR when breastfeeding. Because of this, anyone who is pregnant or breastfeeding may not join this study. Participants who join the study must agree to use an acceptable method of contraception (see Screening Visit for details). Participants who join this study must also agree to be sexually abstinent starting 24 hours prior to the Enrollment Visit and for the duration of study participation. Participants who join this study will have pregnancy tests while in the study.

If you become pregnant at any time during the study, study staff will refer you to available medical care and other services you may need. The study does not pay for this care. You will not receive (or you will stop using) the study VR and you will exit the study. The outcome of your pregnancy is important to study staff; therefore, your pregnancy will be followed until the results of your pregnancy are known. We may contact you to find out about the health of your pregnancy. We may also contact you about a study that collects information about pregnancy and babies up to one year old.

If You Become Infected with HIV
A 25 mg DPV VR was recently shown in two large studies to be safe and to help to prevent HIV. Your participation in this study will not cause HIV infection. The study drugs do not cause HIV. However, there is always a chance that through sexual activity or other activities that you may become HIV-positive. In the unlikely event that you become HIV-positive, study staff will give you counseling and refer you for medical care

and other available services. Tests may be performed to see if you have HIV drug resistance. This will allow doctors to know what HIV drugs would be best for the treatment of your type of HIV. If the HIV tests indicate you may be infected with HIV, you will stop using the VR. If HIV infection is confirmed, you will stop your participation in this study.

BENEFITS

No one knows if the study VRs will prevent pregnancy. As mentioned previously, a dapivirine only ring (25 mg) has been shown to be safe and effective in preventing HIV, but we do not yet know if this VR with 200 mg and levonorgestrel prevents against HIV or pregnancy. Though you may not experience any direct benefit from participation in this study, information learned from this study may help in the development of ways to prevent unwanted pregnancy and the spread of HIV in the future. You will receive HIV/STI risk reduction counseling, HIV and STI testing, physical examinations, pelvic examinations, and routine laboratory testing, including tests to check the overall health of your liver and kidneys.

This study cannot provide you with general medical care, but study staff will refer you to other available sources of care.

You will be counseled and tested for HIV and STIs. If you are infected with HIV, you will be referred for medical care, counseling, and other services available to you. Medical care for HIV infection will not be part of this study. If you have an STI diagnosed, you will receive medicine or a referral, if needed. You can bring your partner here for counseling and referral for testing and treatment for STIs, if needed.

NEW INFORMATION

You will be told of any new information learned during this study that might affect your willingness to stay in the study. For example, if information becomes available that shows that the VR may be causing bad effects, you will be told about this. You will also be told when the study results are available, and how to learn about them. Additionally, you will be told of any new information about other effective HIV-prevention products as they become available.

WHY YOU MAY BE WITHDRAWN FROM THE STUDY WITHOUT YOUR CONSENT

You may be removed from the study early without your permission if:

- The study is cancelled by the US FDA, US NIH, International Partnership for Microbicides (the nonprofit organization that supplies the VRs), the US Office for Human Research Protections (OHRP), the MTN, the local government or regulatory agency, or the Institutional Review Board. An Institutional Review Board is a committee that watches over the safety and rights of research participants
- The Study Monitoring Committee recommends that the study be stopped early. The Study Monitoring Committee reviews the progress of the study and the kinds of effects that people report while they are participating in the study
- You are found to be infected with HIV

- You become pregnant
- Study staff decide that using the VR would be harmful to you, for example, if you have a bad reaction to the study ring
- Other reasons that may prevent you from completing the study successfully, such as inability to consistently keep appointments

If study staff ask you to stop using the VR, you will be asked to complete an interim visit during which time the procedures highlighted to occur at Visit 7 will be completed. Thereafter, you will continue your regular clinic visit schedule with modified procedures, unless otherwise informed by study staff.

In the event that you are removed from or choose to leave this study, you will be asked to return your VR and complete a final evaluation. If you do not have the VR with you at the time of your contact with staff, staff members will make every effort to assist you in returning the ring as soon as possible. ***[SITE TO SPECIFY ALLOWANCES FOR SPECIAL CIRCUMSTANCES]***

COSTS TO YOU

[SITE TO COMPLETE ACCORDING TO SITE CAPACITY]: There is no cost to you for study related visits, the VR, physical/pelvic examinations, laboratory tests or other procedures. Treatments available to you from the study site for HIV/STIs may be given to you free of charge or you will be referred for available treatment for the duration of the study.

REIMBURSEMENT

[*SITE TO INSERT INFORMATION ABOUT LOCAL REIMBURSEMENT*]: You will receive ***[SITE TO INSERT AMOUNT $XX]*** for your time, effort, and travel to and from the clinic at each scheduled visit. You will receive ***[SITE TO INSERT AMOUNT $XX]*** for responding to text messages. You may receive ***[SITE TO INSERT AMOUNT $XX]*** for any visits which occur in between your normally scheduled visits.

CONFIDENTIALITY

Efforts will be made to keep your information confidential. However, it is not possible to guarantee confidentiality. Your personal information may be disclosed if required by law. The study staff may use your personal information to verify that you are not in any other research studies. This includes studies conducted by other researchers that study staff may know about. Any publication of this study will not use your name or identify you personally.

Your records may be reviewed by:

- Representatives of the US Federal Government, including the US FDA, US OHRP, NIH and/or contractors of NIH, and other local and US regulatory authorities
- Representatives of the International Partnership for Microbicides, including study monitors

- PPD (a contract research organization that monitors clinical trials for safety and data quality)
- Site Institutional Review Boards or Ethics Committees
- Study staff

[SITE TO INCLUDE/AMEND THE FOLLOWING]:
Following study participation in the study, you may be referred to other research studies.

[SITE TO INCLUDE/AMEND THE FOLLOWING]:
[LOCAL/STATE/NATIONAL] regulations require study staff to report the names of people who test positive for HIV and other STIs to the ***[LOCAL HEALTH AUTHORITY]***. Outreach workers from the ***[LOCAL HEALTH AUTHORITY]*** may then contact you about informing your partners, since they also should be tested. If you do not want to inform your partners yourself, the outreach workers will contact them, according to the confidentiality guidelines of the ***[HEALTH AUTHORITY]***.

The researchers will do everything they can to protect your privacy. In addition to the efforts of the study staff to help keep your personal information private, we have obtained a Certificate of Confidentiality from the US Federal Government. This Certificate protects study staff from being forced to tell people who are not connected with this study, such as the court system, about your participation or information you give for study purposes. However, if the study staff learns of possible child abuse and/or neglect or a risk of harm to you or others, they will be required to tell the proper authorities. This Certificate does not prevent you from releasing information about yourself and your participation in the study.

RESEARCH-RELATED INJURY
[SITE TO SPECIFY INSTITUTIONAL POLICY]: It is unlikely that you will be injured as a result of study participation. If you are injured, the ***[INSTITUTION]*** will give you immediate necessary treatment for your injuries. You ***[WILL/WILL NOT]*** have to pay for this treatment. You will be told where you can receive additional treatment for your injuries. The U.S. NIH does not have a mechanism to pay money or give other forms of compensation for research related injuries. You do not give up any legal rights by signing this consent form.

CLINICALTRIALS.GOV
A description of this clinical trial will be available on **http://www.ClinicalTrials.gov**. This website will not include information that can identify you. At most, the website will include a summary of the results. You can search this website at any time.

YOUR RIGHTS AS A RESEARCH PARTICIPANT/VOLUNTEER
[SITE TO SPECIFY INSTITUTIONAL POLICY]: Taking part in this study is completely voluntary. You may choose not to take part in this study or leave this study at any time.

If you choose not to participate or to leave the study, you will not lose the benefit of services to which you would otherwise be entitled at this clinic. If you want the results of the study after the study is over, let the study staff members know.

PROBLEMS OR QUESTIONS

If you ever have any questions about the study, or if you have a research-related injury, you should contact ***[INSERT NAME OF THE INVESTIGATOR OR OTHER STUDY STAFF]*** at ***[INSERT TELEPHONE NUMBER AND/OR PHYSICAL ADDRESS]***.

If you have questions about your rights as a research participant, you should contact ***[INSERT NAME OR TITLE OF PERSON ON THE IRB/EC OR OTHER ORGANIZATION APPROPRIATE FOR THE SITE]*** at ***[INSERT PHYSICAL ADDRESS AND TELEPHONE NUMBER]***.

CONSENT FOR LONG-TERM STORAGE AND FUTURE TESTING OF SPECIMENS

There might be a small amount of blood or vaginal fluid left over after we have done all of the study related testing. We would like to ask your permission to store these leftover samples and related health information for use in future studies, such as future research to fight HIV and other related diseases. This health information may include personal facts about you such as your race, ethnicity, sex, medical conditions and your age range. If you agree, your samples and related health data will be stored safely and securely at facilities that are designed so that only approved researchers will have access to the samples. Some employees of the facilities will need to have access to your samples to store them and keep track of where they are, but these people will not have information that directly identifies you. You can still enroll in this study if you decide not to have leftover samples stored for future studies. If you do not want the leftover samples stored, we will destroy them. The type of testing planned for your leftover specimens is not yet known. However, samples may be used by the MTN Laboratory Center to complete additional quality assurance testing, ensuring that the tests work correctly and supply accurate data. No genetic testing on either a limited set or the full set of genes is planned for leftover specimens that are stored for the purposes of future research. It is important that you know that any future testing or studies planned for these specimens must be approved by an Institutional Review Board before they can be done. You can withdraw your consent for the storage and future testing of specimens at any time by providing your request in writing to the person in charge of this study. However, researchers will not be able to destroy samples or information from research that is already underway.

____________<br>Initials and Date	I DO agree to allow my biological specimens and health data to be stored and used in future research studies.
____________<br>Initials and Date	I DO NOT agree to allow my biological specimens and health data to be stored and used in future research studies.

SIGNATURES- VOLUNTARY CONSENT

[INSERT SIGNATURE BLOCKS AS REQUIRED BY THE LOCAL IRB/EC]: If you have read this consent form, or had it read and explained to you, and you understand the information, and you voluntarily agree to the study, please sign your name or make your mark below.

______________________	______________________	______________
Participant Name (print)	Participant Signature/Mark	Date
______________________	______________________	______________
Study Staff Conducting Consent Discussion (print)	Study Staff Signature	Date
______________________	______________________	______________
Witness Name (print)	Witness Signature	Date

Reference List

1. WHO HIV/AIDS Factsheet (updated July 2015) accessed 20 November 2015. http://www.who.int/mediacentre/factsheets/fs360/en/. 2015.

2. UNAIDS report on the global AIDS epidemic-2013. UNAIDS Joint United Nations Programme on HIV/AIDS, 2013

3. Baeten JM, Donnell D, Ndase P et al. Antiretroviral prophylaxis for HIV prevention in heterosexual men and women. N Engl J Med 2012;367(5):399-410.

4. Grant RM, Lama JR, Anderson PL et al. Preexposure chemoprophylaxis for HIV prevention in men who have sex with men. N Engl J Med 2010;363(27):2587-2599.

5. Kott A. Rates of Unintended Pregnancy Remain High In Developing Regions. International perspectives on sexual and reproductive health 2011;37.

6. Singh EJ. Effect of oral contraceptives and IUD's on the copper in human cervical mucus. Obstet Gynecol 1975;45(3):328-330.

7. CONRAD. Phase 1 one-month safety, pharmacokinetic, pharmacodynamic, and acceptability study of intravaginal rings releasing tenonfovir and levonorgestrel or tenofovir alone. Arlington, VA, USA: CONRAD, 2014

8. Singh S, Wulf D, Hussain R, Bankole A, Sedgh G. Abortion worldwide: a decade of uneven progress. Guttmacher Institute; 2009.

9. International Partnership for Microbicides (IPM). Investigator's Brochure: Dapivirine Vaginal Ring. Version 9.0. 12-3-2014.

10. FAME-02: A Phase I trial To assess safety, PK, and PD of gel and film formulations of dapivirine. Conference on Retroviruses and Opportunistic Infections 2014; 14 Mar 3; International Antiviral Society-USA; 2014.

11. International Partnership for Microbicides (IPM). Investigator's Brochure: Dapivirine Vaginal Gel. Version 7.0. 8-28-2013.

12. TMC-120 (R147681), a next generation NNRTI has potent in vitro activity against NNRTI-resistant HIV variants (Abstract 304). 8th Conference on Retroviruses & Opportunistic Infections (CROI); 01 Feb 4; 2015.

13. TMC120, a new non-nucleoside reverse transcriptase inhibitor, is a potent antiretroviral in treatment naive, HIV-1 infected subjects (Abstract 13). 8th Conference on Retroviruses and Opportunistic Infections (CROI); 01 Feb 4; 2015.

14. Baeten JM, Palanee-Phillips T, Brown ER et al. Use of a vaginal ring containing dapivirine for HIV-1 prevention in women. The New England Journal of Medicine. In press.

15. Safety and efficacy of dapivirine vaginal ring for HIV-1 prevention in African women. 2016 Conference on Retroviruses and Opportunistic Infections (CROI); 16 Feb 22; 2016.

16. Fletcher P, Harman S, Azijn H et al. Inhibition of human immunodeficiency virus type 1 infection by the candidate microbicide dapivirine, a nonnucleoside reverse transcriptase inhibitor. Antimicrob Agents Chemother 2009;53(2):487-495.

17. Nel AM, Coplan P, Smythe SC et al. Pharmacokinetic assessment of dapivirine vaginal microbicide gel in healthy, HIV-negative women. AIDS Res Hum Retroviruses 2010;26(11):1181-1190.

18. Di FS, Van RJ, Giannini G et al. Inhibition of vaginal transmission of HIV-1 in hu-SCID mice by the non-nucleoside reverse transcriptase inhibitor TMC120 in a gel formulation. AIDS 2003;17(11):1597-1604.

19. Anderson RM, Swinton J, Garnett GP. Potential impact of low efficacy HIV-1 vaccines in populations with high rates of infection. Proc Biol Sci 1995;261(1361):147-151.

20. International Partnership for Microbicides (IPM). Investigator's Brochure: Dapivirine-Levonorgestrel Vaginal Ring (Version 1.0 Final). 11-16-2015.

21. Dezzutti CS, Yandura S, Wang L et al. Pharmacodynamic Activity of Dapivirine and Maraviroc Single Entity and Combination Topical Gels for HIV-1 Prevention. Pharm Res 2015;32(11):3768-3781.

22. Malcolm K, Murphy D, McCoy C et al. Evaluation of the factors contributing to levonorgestrel binding in addition cure silicone elastomer vaginal rings. AIDS Research and Human Retroviruses 30[S1], A139. 10-30-2014. 1-6-2016.

23. Holt J. Dose Rationale (Long Version): Dapivirine-Levonorgestrel Vaginal Ring, Investigator's Brochure, Version 1.0 Final. Galaska B, editor. 1-5-2016. 1-5-2016.

24. Nippita S, Oviedo J, Velasco M, Westhoff C, Davis A, Castano P. A randomized controlled trial of daily text messages versus monthly paper diaries to collect bleeding data after intrauterine device insertion. Contraception 2015;92(6):578-584.

25. Centers for Disease Control and Prevention (US). United States Medical Eligibility Criteria (US MEC) for Contraceptive Use, 2010. 2014. Atlanta, GA, USA, Centers for Disease Control and Prevention (US). 8-4-2015.

26. Pool R, Montgomery CM, Morar NS et al. A mixed methods and triangulation model for increasing the accuracy of adherence and sexual behaviour data: the Microbicides Development Programme. PLoS One 2010;5(7):e11600.
