# Supplementary material for: Phase 1 randomized trials to assess safety, pharmacokinetics, and vaginal bleeding associated with use of extended duration dapivirine and levonorgestrel vaginal rings
Source: PLoS One. 2024 Jun 5;19(6):e0304552. doi: 10.1371/journal.pone.0304552 (PMC11152307; doi:10.1371/journal.pone.0304552)
Supplement: Peer review history

# Peer review: Decision Letter 0

**DOI:** 10.1371/journal.pone.0304552.r001

**Authors:** Renee Ridzon

10 Dec 2023

PONE-D-23-17718Phase 1 studies to assess safety, pharmacokinetics, and vaginal bleeding associated with use of extended duration dapivirine and levonorgestrel vaginal ringsPLOS ONE

Dear Dr. Achilles,

Thank you for submitting your manuscript to PLOS ONE. After careful consideration, we feel that it has merit but does not fully meet PLOS ONE’s publication criteria as it currently stands. Therefore, we invite you to submit a revised version of the manuscript that addresses the points raised during the review process.

Please submit your revised manuscript by Jan 24 2024 11:59PM. If you will need more time than this to complete your revisions, please reply to this message or contact the journal office at plosone@plos.org. When you're ready to submit your revision, log on to https://www.editorialmanager.com/pone/ and select the 'Submissions Needing Revision' folder to locate your manuscript file.

Please include the following items when submitting your revised manuscript:

- A rebuttal letter that responds to each point raised by the academic editor and reviewer(s). You should upload this letter as a separate file labeled 'Response to Reviewers'.
- A marked-up copy of your manuscript that highlights changes made to the original version. You should upload this as a separate file labeled 'Revised Manuscript with Track Changes'.
- An unmarked version of your revised paper without tracked changes. You should upload this as a separate file labeled 'Manuscript'.

If you would like to make changes to your financial disclosure, please include your updated statement in your cover letter. Guidelines for resubmitting your figure files are available below the reviewer comments at the end of this letter.

If applicable, we recommend that you deposit your laboratory protocols in protocols.io to enhance the reproducibility of your results. Protocols.io assigns your protocol its own identifier (DOI) so that it can be cited independently in the future. For instructions see: https://journals.plos.org/plosone/s/submission-guidelines#loc-laboratory-protocols. Additionally, PLOS ONE offers an option for publishing peer-reviewed Lab Protocol articles, which describe protocols hosted on protocols.io. Read more information on sharing protocols at https://plos.org/protocols?utm_medium=editorial-email&utm_source=authorletters&utm_campaign=protocols.

We look forward to receiving your revised manuscript.

Kind regards,

Renee Ridzon

Academic Editor

PLOS ONE

Journal Requirements:

When submitting your revision, we need you to address these additional requirements.

1. Please ensure that your manuscript meets PLOS ONE's style requirements, including those for file naming. The PLOS ONE style templates can be found at

https://journals.plos.org/plosone/s/file?id=wjVg/PLOSOne_formatting_sample_main_body.pdf and

https://journals.plos.org/plosone/s/file?id=ba62/PLOSOne_formatting_sample_title_authors_affiliations.pdf

2. Thank you for stating the following in the Competing Interests section:

"The following authors have read the journal's policy and have competing interests:  Sharon L. Achilles has received consulting fees from Mayne Pharma and Merck and has received research funding from The National Institutes of Health, the US Food and Drug Administration, the Pennsylvania Department of Health, Society of Family Planning Research Fund, Estetra SRL (an affiliate company of Mithra Pharmaceuticals), EvoFem, and Merck, all of which were managed by Magee-Womens Research Institute. Barbra A. Richardson has received payment from Gilead Sciences for DSMB membership. Brid Devlin and John Steytler were full-time salaried employees of the International Partnership for Microbicides (IPM), a non-profit company registered in the United States of America, at the time the work was performed. Craig W. Hendrix is an Inventor on patent relating to vaginal microbicides and the founder of a microbicide development company, both unrelated to this study product and both managed by Johns Hopkins University. Beatrice A. Chen has served on a Merck & Co. advisory board and has received research grants from Sebela, Mylan, and Medicines360, all of which were managed by Magee-Womens Research Institute. NIH employees (Diana L. Blithe, Jill Brown, and Jeanna M. Piper) contributed to the study design, manuscript development and the decision to publish as well as providing safety oversight during study conduct but had no role in data collection and analysis.

All other authors have declared that no competing interests exist."

Please confirm that this does not alter your adherence to all PLOS ONE policies on sharing data and materials, by including the following statement: "This does not alter our adherence to  PLOS ONE policies on sharing data and materials.” (as detailed online in our guide for authors http://journals.plos.org/plosone/s/competing-interests).  If there are restrictions on sharing of data and/or materials, please state these. Please note that we cannot proceed with consideration of your article until this information has been declared.

Please include your updated Competing Interests statement in your cover letter; we will change the online submission form on your behalf.

3. Please amend either the title on the online submission form (via Edit Submission) or the title in the manuscript so that they are identical

4. In your Data Availability statement, you have not specified where the minimal data set underlying the results described in your manuscript can be found. PLOS defines a study's minimal data set as the underlying data used to reach the conclusions drawn in the manuscript and any additional data required to replicate the reported study findings in their entirety. All PLOS journals require that the minimal data set be made fully available. For more information about our data policy, please see http://journals.plos.org/plosone/s/data-availability.

Upon re-submitting your revised manuscript, please upload your study’s minimal underlying data set as either Supporting Information files or to a stable, public repository and include the relevant URLs, DOIs, or accession numbers within your revised cover letter. For a list of acceptable repositories, please see http://journals.plos.org/plosone/s/data-availability#loc-recommended-repositories. Any potentially identifying patient information must be fully anonymized.

Important: If there are ethical or legal restrictions to sharing your data publicly, please explain these restrictions in detail. Please see our guidelines for more information on what we consider unacceptable restrictions to publicly sharing data: http://journals.plos.org/plosone/s/data-availability#loc-unacceptable-data-access-restrictions. Note that it is not acceptable for the authors to be the sole named individuals responsible for ensuring data access.

We will update your Data Availability statement to reflect the information you provide in your cover letter.

5. Please include your full ethics statement in the ‘Methods’ section of your manuscript file. In your statement, please include the full name of the IRB or ethics committee who approved or waived your study, as well as whether or not you obtained informed written or verbal consent. If consent was waived for your study, please include this information in your statement as well.

Additional Editor Comments:

This is a well written, clear manuscript with important findings form 2 well conducted studies. Apologies in the delay in return as it was extremely hard to get reviewers for this and I think this may be a widespread problem that may be in part due to the pandemic. Please see below for additional minor comments

Abstract

Lines 47, 48-it is not clear which of the 2 groups being compared have the Cmax and AUC measurements that are listed on each of these lines. Are these the values for both groups in each comparison. Please clarify.

Background

Line 59-As written, implies that unintended pregnancy is a health risk. Is this the intention of this statement. Is unintended pregnancy an unintended consequence?

Line 91-will reader know what is intended with the term “single action”?

Line 92-Increased accessibility to what?

Study Design

Line 134-was the exclusion of sexually transmitted infection requiring treatment any infection? Past? Current? Chronic?

Results

Line 298- the text states that for MTN-044/IPM 053/CCN019, the median tmax was 21 days and 28 days for the 2 arms. As this is prior to removal of the it seems that 7 day difference in time to tmax is long. Is this unusual? If so what could account for this?

Line 302, 321, 345, 363-Nadir is a noun. Does it have a past tense form of nadired? Please check and if this is not a correct term, please reword.

Line 351, 360-what do the asterisks in 79.8ng*d/mL and 0.37µg*d/mL indicate?

Line 364-return should be returned

Line 376-stated that there was no difference in bleeding and both continuous and cyclic users, 89% experienced no bleeding to light bleeding. Was there a difference in when the bleeding occurred in these groups. For the cyclic group was bleeding related to when the ring was removed?

Line 411-It is stated that DPV rings used in past trials contained 25m of drug whereas the rings used in these studies contained 200mg. Presumably this is because the rings are designed to be used for longer periods of time. It might be helpful to specifically state this in the manuscript somewhere to help the reader understand why the amount of DPV in the rings is different.

Line 487-stated “genital tract DPV concentrations were not sustained following ring removal and may be fundamental to HIV prevention”. It might be helpful to remind the reader that in the 2 DPV ring studies that showed efficacy, the monthly rings were used continuously without a break between the removal of one ring and reinsertion of a new ring.

[Note: HTML markup is below. Please do not edit.]

Reviewers' comments:

Reviewer's Responses to Questions

**Comments to the Author**

1. Is the manuscript technically sound, and do the data support the conclusions?

The manuscript must describe a technically sound piece of scientific research with data that supports the conclusions. Experiments must have been conducted rigorously, with appropriate controls, replication, and sample sizes. The conclusions must be drawn appropriately based on the data presented. 

Reviewer #1: Yes

Reviewer #2: Yes

**********

2. Has the statistical analysis been performed appropriately and rigorously? 

Reviewer #1: Yes

Reviewer #2: No

**********

3. Have the authors made all data underlying the findings in their manuscript fully available?

The PLOS Data policy requires authors to make all data underlying the findings described in their manuscript fully available without restriction, with rare exception (please refer to the Data Availability Statement in the manuscript PDF file). The data should be provided as part of the manuscript or its supporting information, or deposited to a public repository. For example, in addition to summary statistics, the data points behind means, medians and variance measures should be available. If there are restrictions on publicly sharing data—e.g. participant privacy or use of data from a third party—those must be specified.

Reviewer #1: Yes

Reviewer #2: No

**********

4. Is the manuscript presented in an intelligible fashion and written in standard English?

PLOS ONE does not copyedit accepted manuscripts, so the language in submitted articles must be clear, correct, and unambiguous. Any typographical or grammatical errors should be corrected at revision, so please note any specific errors here.

Reviewer #1: Yes

Reviewer #2: Yes

**********

5. Review Comments to the Author

Please use the space provided to explain your answers to the questions above. You may also include additional comments for the author, including concerns about dual publication, research ethics, or publication ethics. (Please upload your review as an attachment if it exceeds 20,000 characters)

Reviewer #1: ABSTRACT: MTN030/IPM 041 compared DPV/LNG to DPV for 14 days of use. MTN-044/IPM 053/CCN019 compared continuous or cyclic use of DPV/LNG over 90 days. Higher Cmax of DPV with DPV LNG IVR compared to DPV IVR, despite having the same concentration of DPV in each IVR. Would add that the LNG plasma concentrations are comparable with other efficacious SYSTEMICALLY delivered LNG-based contraceptives.

BACKGROUND: I think lines 85 – 88 can be removed from the introduction as this is more PD related.

METHODS: For the MTN-030/IPM 041 study – Lines 144 – 145, please clarify if the IP was inserted at a particular time in the menstrual cycle as this would potentially impact vaginal bleeding endpoint.

For the MTN-044/IPM 053/CCN 019 study, lines 158 – 159, I’m assuming that the IP was inserted at the end of menses, but please clarify if the IP insertion was timed to the menstrual cycle. Line 166 – I’m assuming these are ectocervical tissue biopsies, but please clarify.

RESULTS: Minor detail line 227 – it says 25 participants enrolled IN Pittsburgh. Should this be at Pittsburgh site? Table 2 and Table 3 are well presented. I understand from the methods how vaginal bleeding was recorded in the 90 day study (daily diary entry), would you also include how vaginal bleeding was captured in the 14 day study, since the results do go in to the incidence of vaginal bleeding in the 14-day study. I’m assuming that this was just by participant report at visits, but would you clarify. That is really great that 89% of women had no bleeding to light bleeding in the 90-day study – I would point to Table 4 in lines 376 – 377 where that result is presented.

DISCUSSION: DPV PK data is well summarized. I’m assuming that because the DPV plasma concentrations are higher with the 90 day IVRs, the assumption is that they would have comparable efficacy to the ASPIRE study DPV 28-day IVR. I’m familiar with the Cherala LNG paper (ref 46), but what is the reference for the statement on lines 418 – 419 that the threshold of 225 pg/mL using MASS SPECTROMETRY- based methods? The Cherala paper talks about the Norplant, RIA thresholds for LNG. I disagree with lines 421 – 423 – the “threshold” concentrations for LNG are for systemically delivered LNG, so the median serum concentrations of 240 pg/mL and 210 pg/mL at days 30 and 60 respectively are likely near the minimal threshold for contraceptive efficacy for systemically delivered LNG, but the “threshold” for vaginally delivered LNG is not known. I would include this caveat, because lines 421 – 423, as written may make some readers think that cyclic use would result in contraceptive failure, and this is clearly not known. I think you can work in the next paragraph (lines 424 – 430) to clarify this. I’m not really understanding how lines 428 – 430 fit in to the argument. I think you should potentially add to the discussion – why do you think the DPV Cmax was higher with the DPV/LNG IVR? Is this just statistically significant but perhaps not clinically significant.

Overall these two studies are very important data to add to the MPT literature!

Reviewer #2: Two phase 1 randomized clinical trials were conducted which aimed to evaluate safety, pharmacokinetics, and vaginal bleeding associated with the use of vaginal rings. One trial compared 14-day DPV to 14-day DPV/LNG while the other compared 90-day DPV/LNG continuous to 90-day DPV/LNG cyclic. No statistical difference was observed in the proportion of participants with grade 2 or higher genitourinary AEs in the DPV/LNG ring arm compared to the DPV ring arm. No statistically significant differences were observed in the proportion of participants with grade ≥2 genitourinary AEs in the continuous use compared with the cyclic use arm, and no statistically significant differences were observed in the proportion of participants with grade ≥3 AEs in the continuous use compared with the cyclic use.

Major revision:

Provide a comprehensive statistical analysis section, listing and describing the use of all statistical methods from which p-values were estimated.

Minor revisions:

1- Line 207: Provide a reference and note the software for calculating AUC using the trapezoidal method.

2- Indicate the date range subjects were enrolled in the studies.

3- Cite the statistical software used for the analysis.

**********

6. PLOS authors have the option to publish the peer review history of their article (what does this mean?). If published, this will include your full peer review and any attached files.

If you choose “no”, your identity will remain anonymous but your review may still be made public.

**Do you want your identity to be public for this peer review?** For information about this choice, including consent withdrawal, please see our Privacy Policy.

Reviewer #1: **Yes: **Andrea Thurman MD

Reviewer #2: No

**********

[NOTE: If reviewer comments were submitted as an attachment file, they will be attached to this email and accessible via the submission site. Please log into your account, locate the manuscript record, and check for the action link "View Attachments". If this link does not appear, there are no attachment files.]

While revising your submission, please upload your figure files to the Preflight Analysis and Conversion Engine (PACE) digital diagnostic tool, https://pacev2.apexcovantage.com/. PACE helps ensure that figures meet PLOS requirements. To use PACE, you must first register as a user. Registration is free. Then, login and navigate to the UPLOAD tab, where you will find detailed instructions on how to use the tool. If you encounter any issues or have any questions when using PACE, please email PLOS at figures@plos.org. Please note that Supporting Information files do not need this step.

# Author comment: Author response to Decision Letter 0

**DOI:** 10.1371/journal.pone.0304552.r002

9 May 2024

Responses to Editor Comments

• Comment 1: Abstract

Lines 47, 48-it is not clear which of the 2 groups being compared have the Cmax and AUC measurements that are listed on each of these lines. Are these the values for both groups in each comparison. Please clarify.

Response 1: Please note that there are no Cmax or AUC values listed, rather for both Cmax and for AUC, the numbers that follow are p-values for the comparisons demonstrating no difference between the respective groups. No change was made to the document.

• Comment 2: Background

Line 59-As written, implies that unintended pregnancy is a health risk. Is this the intention of this statement. Is unintended pregnancy an unintended consequence?

Response 2: Thank you for this comment. Yes, we intended the statement as written since unintended pregnancy is both an unintended consequence and it poses additional health risk in addition to the baseline increased health risk associated with pregnancy in general. There is increased morbidity and mortality among those with unintended pregnancy compared to those with planned pregnancies, likely related to multifactorial causes notably including differences in access to care that likely differentially limit access to reliable contraception as well as to prenatal care. No change was made to the document.

• Comment 3: Line 91-will reader know what is intended with the term “single action”?

Response 3: We agree that ‘single action’ was vague and we have adjusted the wording to provide better clarity. New wording to lines 95-98: “The development of a ring containing DPV for HIV prevention and levonorgestrel (LNG) for contraception, both with adequate loading doses to support extended release for 3-months continuous use, allows for use of a single ring with less frequent ring replacements compared to simultaneous use of monthly contraceptive and HIV preventative rings.”

• Comment 4: Line 92-Increased accessibility to what?

Response 4: Thank you for requesting this clarification. As demonstrated with long-acting contraceptives whereby access to contraception increased by decreasing the frequency with which one needs to interact with healthcare systems that are often fraught with barriers particularly for those with the fewest resources, long-acting MPTs would be anticipated to provide increased accessibility to sexual and reproductive health protection. We have added to the statement on lines 99-101 as follows: “…extended duration MPT rings replaced quarterly may further reduce user and provider burden, thereby increasing accessibility to important preventative sexual and reproductive health options and improving adherence.”

• Comment 5: Study Design

Line 134-was the exclusion of sexually transmitted infection requiring treatment any infection? Past? Current? Chronic?

Response 5: We have provided clarification in the text. New wording: “…current sexually transmitted infection requiring treatment…”

• Comment 6: Results

Line 298- the text states that for MTN-044/IPM 053/CCN019, the median tmax was 21 days and 28 days for the 2 arms. As this is prior to removal of the it seems that 7 day difference in time to tmax is long. Is this unusual? If so what could account for this?

Response 6: This apparent difference is neither statistically (based upon formal statistical testing p value >0.05) nor clinically significant. We judge this apparent difference not to be clinically significant because the antiviral effect of dapivirine, like antiretrovirals generally, is driven by the minimum effective concentration, not maximum (peak) concentrations. Further, the plasma DPV concentration is indirectly reflecting temporal changes in the more relevant site of action of cervicovaginal tissue and is not itself (plasma) determinant of PrEP efficacy, though some very useful associations have been described.

In the case of dapivirine one-month rings, high levels of adherence and protection were demonstrated with plasma DPV concentrations achieving >95 pg/mL within 48 hours. In both MTN-030 and MTN-044, the lower quartile of the 48hr DPV plasma concentrations exceed 95 pg/mL. The time to or absolute value of Cmax is of little import for DPV efficacy. It is useful as a PK parameter to describe performance of the formulation, but not efficacy. 

Statistically, the apparent 7-day difference in median Tmax for the two arms is, to some degree, an artifact of the method for calculating medians and the discrete visit days at that point in the study, at least 14 days apart (study visits are days 0, 2, 14, 28, 30, 44, 58, 60, 74, 90, 91, 92, and a final visit on day 93-94.) The actual difference could be trivial or up to 7 days. There is no way to tell using the median, though the interquartile range provides evidence supporting no important difference. 

The median calculation will always either fall upon a visit day or the midpoint between two visits. In the case of the cyclic arm, the day 28 median indicates that the middle value (50th percentile) among all Tmax values falls on the day 28 visit. For the continuous use arm, the day 21 median indicates that the midpoint value among all Tmax observations falls between the day 14 and day 28 visit (and can only indicate the midpoint, not some other number influenced by a specific proportion at or below the day 14 visit or at or above day 28 visit. So, it is possible that as few as one Tmax value could be the difference in a day 21 vs. day 28 median. It could also be more.

The lower quartile is also helpful here because at the day 14 visit, they are the same in both arms. The upper quartile is the only value that is different than one visit, with 66 days (falling between day 60 and day 74 visits) and 44 days falling on the day 44 visit. Overall, these distributions are largely overlapping, and the apparent difference is not statistically significant. No change was made to the document in the Results—please see answer to Reviewer 1, Comment 13 for related addition to the Discussion.

• Comment 7: Line 302, 321, 345, 363-Nadir is a noun. Does it have a past tense form of nadired? Please check and if this is not a correct term, please reword.

Response 7: Thank you, we have corrected this in each instance with the following replacement sentence structure: “…nadir median CVF DPV concentrations were…”

• Comment 8: Line 351, 360-what do the asterisks in 79.8ng*d/mL and 0.37µg*d/mL indicate?

Response 8: The asterisks (*) represent multiplication symbol as we have seen most commonly in other published literature. In this case the units of the calculated AUC are concentration (ng/mL and µg/mL here) times time in days (d). If the journal has a preferred symbol to represent multiplication, please advise. No change was made to the document.

• Comment 9: Line 364-return should be returned

Response 9: Respectfully, we posit that the sentence is grammatically correct as written. The past participle (‘had returned’) is used here since the observation is that the moment of return of the LNG concentrations must have occurred in the past relative to the time of the sampling. No change was made to the document.

• Comment 10: Line 376-stated that there was no difference in bleeding and both continuous and cyclic users, 89% experienced no bleeding to light bleeding. Was there a difference in when the bleeding occurred in these groups. For the cyclic group was bleeding related to when the ring was removed?

Response 10: Thank you for this question that prompted us to look a bit further at the data. Our predetermined analysis of bleeding was on number of bleeding days and bleeding incidence, which we reported and demonstrates no difference between the continuous vs cyclic use groups. We have now opted to include additional reporting on a pattern seen in the data on windows of less bleeding in the cyclic users compared to the continuous users following each cycle of ring removal. The following changes were made to lines 412-418: “In both continuous and cyclic users, 89% experienced no bleeding to light bleeding (Table 4). Although there were no significant differences in the number of bleeding episodes or bleeding incidence rates for continuous compared to cyclic use of the ring, cyclic users experienced less or a trend toward less bleeding in the days following each ring reinsertion compared to continuous users: On days 34-37, 9/11 (82%) of continuous users and 4/12 (33%) of cyclic users reported any bleeding (p=0.04) and on days 64-67, 6/9 (67%) of continuous users and 5/12 (42%) of cyclic users reported any bleeding (p=0.39).”

• Comment 11: Line 411-It is stated that DPV rings used in past trials contained 25m of drug whereas the rings used in these studies contained 200mg. Presumably this is because the rings are designed to be used for longer periods of time. It might be helpful to specifically state this in the manuscript somewhere to help the reader understand why the amount of DPV in the rings is different.

Response 11: Yes, the increased drug load in the vaginal ring (200mg compared to 25mg) is to support the longer, 3-month duration of use. We have made this more explicit in the background. Changes to lines 88-90 were made as follows: “More recently, a DPV vaginal ring containing 200mg DPV designed for extended use (3 month) has been evaluated for pharmacokinetics and safety…”

• Comment 12: Line 487-stated “genital tract DPV concentrations were not sustained following ring removal and may be fundamental to HIV prevention”. It might be helpful to remind the reader that in the 2 DPV ring studies that showed efficacy, the monthly rings were used continuously without a break between the removal of one ring and reinsertion of a new ring.

Response 12: Agree with this addition to add clarity. The following addition was made to lines 539-540: “were not sustained following ring removal and may be fundamental to HIV prevention as has been demonstrated with continuous use of the monthly 25mg DPV vaginal ring…”

Responses to Reviewer 1

• Comment 1: ABSTRACT: MTN030/IPM 041 compared DPV/LNG to DPV for 14 days of use. MTN-044/IPM 053/CCN019 compared continuous or cyclic use of DPV/LNG over 90 days. Higher Cmax of DPV with DPV LNG IVR compared to DPV IVR, despite having the same concentration of DPV in each IVR. Would add that the LNG plasma concentrations are comparable with other efficacious SYSTEMICALLY delivered LNG-based contraceptives.

Response 1: Since there are no topically delivered LNG-based contraceptives with demonstrated efficacy, we view the addition of the word ‘SYSTEMICALLY’ to not add additional value and may distract from the intended emphasis on levonorgestrel. Thus, our preference is to leave the sentence unchanged. No change was made to the document.

• Comment 2: BACKGROUND: I think lines 85 – 88 can be removed from the introduction as this is more PD related.

Response 2: Agree that these sentences can be removed. These sentences were both deleted entirely, and the paragraph starting on line 95 now begins: “The development of a ring containing DPV for HIV prevention and levonorgestrel (LNG) for contraception, both with adequate loading doses to support extended release for 3-months continuous use, …”

• Comment 3: METHODS: For the MTN-030/IPM 041 study – Lines 144 – 145, please clarify if the IP was inserted at a particular time in the menstrual cycle as this would potentially impact vaginal bleeding endpoint.

Response 3: We have added a clarifying sentence to the methods to address the timing of enrollment and IP insertion relative to menstrual cycle. The following addition was made to lines 153-156: “In both trials, the enrollment visit was scheduled with consideration of the menstrual cycle with ideally no anticipated bleeding during the 14 days of study product use in MTN-030/IPM 041 and no anticipated bleeding during the first 3 days of study product use in MTN-044/IPM 053/CCN019.”

• Comment 4: For the MTN-044/IPM 053/CCN 019 study, lines 158 – 159, I’m assuming that the IP was inserted at the end of menses, but please clarify if the IP insertion was timed to the menstrual cycle.

Response 4: See response to Reviewer 1, Comment 3. No additional change was made to the document.

• Comment 5: Line 166 – I’m assuming these are ectocervical tissue biopsies, but please clarify.

Response 5: Yes, the cervical biopsies were of ectocervical tissue. The following addition was made to line 184: “Ectocervical tissue biopsies were collected…”

• Comment 6: RESULTS: Minor detail line 227 – it says 25 participants enrolled IN Pittsburgh. Should this be at Pittsburgh site?

Response 6: Both the original and the alternatively suggested wording are technically and grammatically correct. The following changes were made to lines 237 and 251 respectively: “Twenty-four participants enrolled at the Birmingham (n=12) and Pittsburgh sites.” And “Twenty-five participants enrolled at the Pittsburgh site.”

• Comment 7: Table 2 and Table 3 are well presented. I understand from the methods how vaginal bleeding was recorded in the 90 day study (daily diary entry), would you also include how vaginal bleeding was captured in the 14 day study, since the results do go in to the incidence of vaginal bleeding in the 14-day study. I’m assuming that this was just by participant report at visits, but would you clarify.

Response 7: Thank you for demonstrating that the methods for assessment of vaginal bleeding were not clearly articulated. To correct this, we have made the following addition to lines 132-141: “Methods for assessing safety, local and systemic drug concentrations, and vaginal bleeding were the same in both trials as follows unless otherwise noted: Safety was evaluated…Participants answered a daily questionnaire by short message service (SMS) that included reporting of vaginal bleeding as follows: none, light (used no protection, toilet paper, or panty liner only), moderate (used pad or tampon), or heavy (leaked through pad or tampon).”

• Comment 8: That is really great that 89% of women had no bleeding to light bleeding in the 90-day study – I would point to Table 4 in lines 376 – 377 where that result is presented.

Response 8: We have added an additional reference to Table 4 as follows on line 413: “In both continuous and cyclic users, 89% experienced no bleeding to light bleeding (Table 4).”

• Comment 9: DISCUSSION: DPV PK data is well summarized. I’m assuming that because the DPV plasma concentrations are higher with the 90 day IVRs, the assumption is that they would have comparable efficacy to the ASPIRE study DPV 28-day IVR.

Response 9: Yes, correct, this hypothesis would need to be tested in further trials. As articulated in the discussion, in the ASPIRE clinical trial evaluation of the 25mg DPV ring, plasma concentrations >95pg/mL correlated with adherence and with reduction in HIV acquisition. Thus, we thought it important to point out in the discussion that the plasma concentrations observed in these early phase trials of a 90-day DPV ring were consistently above the ASPIRE threshold. No change was made to the document.

• Comment 10: I’m familiar with the Cherala LNG paper (ref 46), but what is the reference for the statement on lines 418 – 419 that the threshold of 225 pg/mL using MASS SPECTROMETRY- based methods?

Response 10: We have added a couple of relevant references here and we have revised the language to best reflect the literature assessment. The following revision was made to line 463: “…however it can be conservatively surmised to be above approximately 225pg/mL as measured….”

• Comment 11: The Cherala paper talks about the Norplant, RIA thresholds for LNG. I disagree with lines 421 – 423 – the “threshold” concentrations for LNG are for systemically delivered LNG, so the median serum concentrations of 240 pg/mL and 210 pg/mL at days 30 and 60 respectively are likely near the minimal threshold for contraceptive efficacy for systemically delivered LNG, but the “threshold” for vaginally delivered LNG is not known. I would include this caveat, because lines 421 – 423, as written may make some readers think that cyclic use would result in contraceptive failure, and this is clearly not known. I think you can work in the next paragraph (lines 424 – 430) to clarify this.

Response 11: While we ag

# Editor report: Decision Letter 1

**DOI:** 10.1371/journal.pone.0304552.r003

**Authors:** Renee Ridzon

15 May 2024

Phase 1 randomized trials to assess safety, pharmacokinetics, and vaginal bleeding associated with use of extended duration dapivirine and levonorgestrel vaginal rings

PONE-D-23-17718R1

Dear Dr. Achilles,

We’re pleased to inform you that your manuscript has been judged scientifically suitable for publication and will be formally accepted for publication once it meets all outstanding technical requirements.

Within one week, you’ll receive an e-mail detailing the required amendments. When these have been addressed, you’ll receive a formal acceptance letter and your manuscript will be scheduled for publication.

An invoice will be generated when your article is formally accepted. Please note, if your institution has a publishing partnership with PLOS and your article meets the relevant criteria, all or part of your publication costs will be covered. Please make sure your user information is up-to-date by logging into Editorial Manager at Editorial Manager® and clicking the ‘Update My Information' link at the top of the page. If you have any questions relating to publication charges, please contact our Author Billing department directly at authorbilling@plos.org.

If your institution or institutions have a press office, please notify them about your upcoming paper to help maximize its impact. If they’ll be preparing press materials, please inform our press team as soon as possible -- no later than 48 hours after receiving the formal acceptance. Your manuscript will remain under strict press embargo until 2 pm Eastern Time on the date of publication. For more information, please contact onepress@plos.org.

Kind regards,

Renee Ridzon

Academic Editor

PLOS ONE

Additional Editor Comments (optional):

Reviewers' comments:

# Editor report: Acceptance letter

**DOI:** 10.1371/journal.pone.0304552.r004

**Authors:** Renee Ridzon

27 May 2024

PONE-D-23-17718R1 

PLOS ONE

Dear Dr. Achilles, 

I'm pleased to inform you that your manuscript has been deemed suitable for publication in PLOS ONE. Congratulations! Your manuscript is now being handed over to our production team.

At this stage, our production department will prepare your paper for publication. This includes ensuring the following:

* All references, tables, and figures are properly cited

* All relevant supporting information is included in the manuscript submission,

* There are no issues that prevent the paper from being properly typeset

If revisions are needed, the production department will contact you directly to resolve them. If no revisions are needed, you will receive an email when the publication date has been set. At this time, we do not offer pre-publication proofs to authors during production of the accepted work. Please keep in mind that we are working through a large volume of accepted articles, so please give us a few weeks to review your paper and let you know the next and final steps. 

Lastly, if your institution or institutions have a press office, please let them know about your upcoming paper now to help maximize its impact. If they'll be preparing press materials, please inform our press team within the next 48 hours. Your manuscript will remain under strict press embargo until 2 pm Eastern Time on the date of publication. For more information, please contact onepress@plos.org.

If we can help with anything else, please email us at customercare@plos.org.

Thank you for submitting your work to PLOS ONE and supporting open access. 

Kind regards, 

PLOS ONE Editorial Office Staff

on behalf of

Dr. Renee Ridzon 

Academic Editor

PLOS ONE
